# Association of Early Interventions With Birth Outcomes and Child Linear Growth in Low-Income and Middle-Income Countries: Bayesian Network Meta-analyses of Randomized Clinical Trials

**DOI:** 10.1001/jamanetworkopen.2019.7871

**Published:** 2019-07-26

**Authors:** Jay J. H. Park, Mei Lan Fang, Ofir Harari, Louis Dron, Ellie G. Siden, Reham Majzoub, Virginia Jeziorska, Kristian Thorlund, Edward J. Mills, Zulfiqar A. Bhutta

**Affiliations:** 1 Experimental Medicine, Department of Medicine, Faculty of Medicine, University of British Columbia, Vancouver, British Columbia, Canada; 2 MTEK Sciences, Vancouver, British Columbia, Canada; 3 School of Nursing and Health Sciences, University of Dundee, Dundee, United Kingdom; 4 Department of Health Research Methodology, Evidence, and Impact, McMaster University, Hamilton, Ontario, Canada; 5 Center of Excellence in Women and Child Health, The Aga Khan University, Karachi, Pakistan; 6 Centre for Global Child Health, The Hospital for Sick Children, Toronto, Ontario, Canada

## Abstract

**Importance:**

The first 1000 days of life represent a critical window for child development. Pregnancy, exclusive breastfeeding (EBF) period (0-6 months), and complementary feeding (CF) period (6-24 months) have different growth requirements, so separate considerations for intervention strategies are needed. No synthesis to date has attempted to quantify the associations of interventions under multiple domains of micronutrient and balanced energy protein and food supplements, deworming, maternal education, water sanitation, and hygiene across these 3 life periods with birth and growth outcomes.

**Objective:**

To determine the magnitude of association of interventions with birth and growth outcomes based on randomized clinical trials (RCTs) conducted in low-income and middle-income countries (LMICs) using Bayesian network meta-analyses.

**Data Sources:**

MEDLINE, Embase, and Cochrane databases were searched from their inception up to August 14, 2018.

**Study Selection:**

Included were LMIC-based RCTs of interventions provided to pregnant women, infants (0-6 months), and children (6-24 months).

**Data Extraction and Synthesis:**

Two independent reviewers used a standardized data extraction and quality assessment form. Random-effects network meta-analyses were performed for each life period. Effect sizes are reported as odds ratios (ORs) and mean differences (MeanDiffs) for dichotomous and continuous outcomes, with 95% credible intervals (CrIs). This study calculated probabilities of interventions being superior to standard of care by at least a minimal clinically important difference.

**Main Outcomes and Measures:**

The study compared ORs on preterm birth and MeanDiffs on birth weight for pregnancy, length for age (LAZ) for EBF, and height for age (HAZ) for CF.

**Results:**

Among 302 061 participants in 169 randomized clinical trials, the network meta-analyses found several nutritional interventions that demonstrated greater association with improved birth and growth outcomes compared with standard of care. For instance, compared with standard of care, maternal supplements of multiple micronutrients showed reduced odds for preterm birth (OR, 0.54; 95% CrI, 0.27-0.97) and improved mean birth weight (MeanDiff, 0.08 kg; 95% CrI, 0.00-0.17 kg) but not LAZ during EBF (MeanDiff, −0.02; 95% CrI, −0.18 to 0.14). Supplementing infants and children with multiple micronutrients showed improved LAZ (MeanDiff, 0.20; 95% CrI, 0.03-0.35) and HAZ (MeanDiff, 0.14; 95% CrI, 0.02-0.25). The study found that pregnancy interventions generally had higher probabilities of a minimal clinically importance difference than the interventions for the EBF or CF in the first 1000 days of life.

**Conclusions and Relevance:**

These analyses highlight the importance of intervening early for child development, during pregnancy if possible. Results of this study suggest that there is a need to combine interventions from multiple domains and test for their effectiveness as a package.

## Key Points

**Question:**

Which interventions under the domains of nutrition, deworming, maternal education, and water, sanitation, and hygiene can improve birth and linear growth outcomes during the first 1000 days of life in low-income and middle-income countries (LMICs)?

**Findings:**

This study used Bayesian network meta-analyses of 169 randomized clinical trials including 302 061 participants and showed that several nutritional interventions demonstrating greater associations with improved outcomes compared with standard of care, while other domains generally did not. Interventions provided to pregnant women generally demonstrated greater associations with improved outcomes than interventions provided to infants and children at later periods.

**Meaning:**

The study findings suggest that it is important to intervene early for child development in LMICs, during pregnancy if possible, and combine interventions from multiple domains and test for their effectiveness.

## Teaser

This study used Bayesian network meta-analyses to assess the magnitude of association of interventions provided to pregnant women, infants, and young children with child development birth and growth outcomes based on randomized clinical trials conducted in low-income and middle-income countries (LMICs).

## Introduction

Between 1990 and 2015, remarkable progress was made toward the Millennium Development Goals of reducing childhood morbidity and mortality.^1^ However, childhood stunting, defined as a height for age (HAZ) score of less than 2 SDs below the World Health Organization (WHO) Child Growth Standards median,^2^ continues to be a critical public health issue, particularly in low-income and middle-income countries (LMICs).^3,4,5^ In 2017, it was estimated that 22.2% of all children under the age of 5 years had stunted development, with most of the burden shouldered by Asian (55%) and African (39%) countries.^6^

Childhood stunting can have immediate and long-lasting negative consequences on both physical growth and neurodevelopment.^3^ Immediate consequences include decreased survival during infancy and greater childhood susceptibility to frequent infections.^4,7,8,9^ Further developmental deficits can include sensory, motor, cognitive, language, socioemotional, cultural, and behavioral impairments. These health and developmental issues can hamper personal, educational, and professional attainment.^4,7,8,10,11^

The first 1000 days of life, a period spanning from conception to a child’s second birthday, are critical for child development.^8,12,13^ This window also represents a key opportunity for stunting intervention delivery because stunting frequently begins during this time.^13^ The first 1000 days of life are often further divided into the following 3 key periods: pregnancy, the exclusive breastfeeding (EBF) period (0-6 months of age), and the complementary feeding (CF) period (6-24 months of age).^14^ Each of these periods deserves separate consideration with regard to the delivery of stunting interventions because growth determinants and requirements between them differ greatly.^14^

Over the last decade, several review articles of clinical trials or observational studies have rigorously investigated the consequences of interventions addressing stunting. However, all of these focused on a single class of interventions or investigated within a single early-life period (eTable 1 in the Supplement).^12^ Similarly, comprehensive reviews have been limited to summarizing key findings of more focused review articles.^11,12^ No synthesis to date has attempted to summarize and quantify the effectiveness of multiple classes of interventions across the 3 key early-life periods of pregnancy, EBF, and CF.

Network meta-analysis is an extension of conventional pairwise meta-analysis that allows for the comparison of interventions that have not been compared directly in head-to-head randomized clinical trials (RCTs).^15^ This article uses a systematic review and network meta-analysis approach to investigate the magnitude of association of interventions with reduced adverse birth outcomes and improved linear growth in LMICs. We applied this approach separately to each of the 3 life periods to determine the consequences of interventions classified as either micronutrients, balanced energy protein or food supplements, deworming, maternal education, or water, sanitation, and hygiene (WASH) interventions. In this article, we report the magnitude of associations of the above interventions with birth outcomes and linear growth outcomes in the 3 key life periods across the first 1000 days of life.

## Methods

We conducted this study according to the Preferred Reporting Items for Systematic Reviews and Meta-analyses (PRISMA) extension to network meta-analysis^16^ and report this study according to the *JAMA* users’ guides on network meta-analysis.^17^ The systematic reviews and NMAs conducted for this study were registered in PROSPERO.^18,19,20^

### Search Strategy

**Figure 1.**
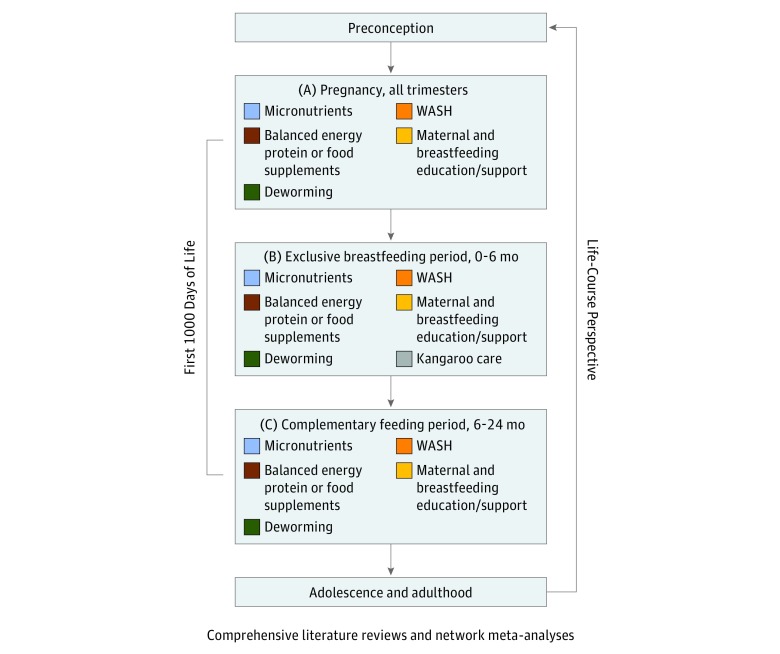
Life-Course Conceptual Framework for Linear Growth Interventions The first 1000 days of life were separated into 3 periods because the growth determinants and requirements for these periods differ greatly. The conceptual framework is informed by the ecological systems theory by Bronfenbrenner.^21^ Interventions during pregnancy and childhood will have health and social implications in adolescence and in adulthood. This will in turn alter the health and social outcomes of the next generation of society. This study was guided by comprehensive literature reviews and network meta-analyses and by the concepts in the figure. WASH indicates water, sanitation, and hygiene.

We aimed to capture all relevant interventions that alter early childhood growth outcomes. We developed a life-course conceptual framework to guide the study, specifically to determine the appropriate intervention domains at each life period (Figure 1). We identified relevant LMIC-based trials by scanning prior systematic reviews and global health policy guidelines. This step formulated the trial eligibility criteria in the form of PICOS (population, interventions, comparisons, outcomes, and study design) summarized in Table 1.

**Table 1.** PICOS Criteria for Trial Selection

Criterion	Pregnancy, All Trimesters	Exclusive Breastfeeding Period, 0-6 mo	Complementary Feeding Period, 6-24 mo
Population	Pregnant women living in LMICs	Newborn infants aged 0-6 mo living in LMICs	Children aged 6-24 mo living in LMICs
Interventions	Micronutrient and calcium supplementation to mother Balanced energy protein (ie, food) supplementation to mother Deworming Maternal education Any WASH intervention	Micronutrient and calcium supplementation to mother or to infant Balanced energy protein supplementation to mother Food supplementation or fortification to infant Deworming Maternal and breastfeeding education/support Any WASH intervention Kangaroo care^a^	Micronutrient and calcium supplementation to children Food supplementation to children Deworming Maternal education/support Any WASH intervention
Comparisons	Placebo Standard of care (if applicable) No intervention Any of the interventions listed above as monotherapy or in combination that can be used for indirect comparison		
Outcomes	At least 1 of the following 3 outcomes: Preterm birth (<37 gestational wk) Low birth weight (<2500 g)^a^ Birth weight (continuous)	At least 1 of the following 4 outcomes: LAZ *z* score Proportion of stunted (LAZ less than −2 SDs)^a^ Height/length^a^ Head circumference^a^	At least 1 of the following 2 outcomes: HAZ *z* score Proportion of stunted (HAZ less than −2 SDs)^a^
Study design	Randomized clinical trial		

Abbreviations: HAZ, height for age; LAZ, length for age; LMICs, low-income and middle-income countries; PICOS, population, interventions, comparisons, outcomes, and study design; WASH, water, sanitation, and hygiene.

^a^ The details on the analyses and results of kangaroo care and other interventions’ magnitude of associations with low birth weight, stunting, height/length, and head circumference will be published elsewhere. To maintain external validity and prevent inflation of findings, trials representing subpopulations, such as infants born to HIV-positive mothers and infants born preterm, with low birth weight, with severe acute malnutrition, or with preexisting health conditions, were excluded for all 3 life-period analyses.

For each life period, we implemented 2-way sensitivity searches whereby we first hand-searched systematic reviews and trials highlighted in recent global health guidelines and key maternal, newborn, and child health (MNCH) articles (eFigure 1 in the Supplement). We followed this with a comprehensive search of interventions for all life periods published from database inception up to August 14, 2018. We searched the MEDLINE, Embase, and Cochrane Central Register of Controlled Trials databases, as well performing hand searches of review article bibliographies. Full search terms and details of the electronic searches are provided in the eAppendix and eTables 2 through 7 in the Supplement. The full texts of identified English-language articles were assessed independently by a paired group of 4 reviewers (J.J.H.P., M.L.F., R.M., and V.J.) using a standardized data extraction and quality assessment form. Any disagreements were settled by a fifth reviewer (K.T.).

### Study Selection, Data Extraction, and Outcome Measures

Trials related to micronutrient supplementation, balanced energy protein or food supplementation, deworming, maternal education/support that included breastfeeding strategies and psychosocial support, WASH, and kangaroo care were included for data extraction if they reported preterm birth (<37 gestational weeks), birth weight (continuous), and/or low birth weight (<2500 g) outcomes for pregnancy; length for age (LAZ), proportion stunted (LAZ less than −2 SDs), height/length, and/or head circumference for the EBF period; or HAZ and/or proportion stunted (HAZ less than −2 SDs) for the CF period. Trials that did not meet all the PICOS criteria were excluded. This review article presents the details and the results on the preterm birth, birth weight, LAZ, and HAZ; the details and the results of kangaroo care and other classes of interventions’ magnitude of associations with low birth weight, stunting, height/length, and head circumference will be published elsewhere.

Two reviewers (J.J.H.P. and M.L.F.) independently extracted the data into a standardized spreadsheet (Excel; Microsoft) and categorized results corresponding to the PICOS criteria for each of the systematic reviews. Cross-checking for consistency was conducted by other reviewers (L.D. and K.T.). For each eligible article, we extracted study characteristics (eg, title, first author, year of publication, country, intervention type, and intervention arm), participant information (eg, mother’s age, gestational age, sex of child, and intervention duration), and outcome measures (eg, preterm birth, birth weight, and LAZ/HAZ). In addition, 2 reviewers (J.J.H.P. and E.G.S.) assessed risk of bias in the included RCTs using the Cochrane Collaboration’s tool for assessing risk of bias.^22^

### Statistical Analysis

We applied Bayesian evidence synthesis models, as recommended by the National Institute for Health and Care Excellence in their Technical Support Document 2 (NICE TSD2).^23^ For pregnancy, the magnitudes of associations of interventions are measured using odds ratios (ORs) with associated 95% credible intervals (95% CrIs) for preterm birth and mean differences (MeanDiffs) with associated 95% CrIs for birth weight. For EBF and CF life periods, the interventions’ magnitudes of associations with LAZ and HAZ are measured using MeanDiffs with associated 95% CrIs.

To allow for comparisons of interventions across multiple life periods, we calculated the posterior probability for achieving a minimally clinically importance difference (MCID) for preterm birth and birth weight (pregnancy), LAZ (EBF), and HAZ (CF) outcomes. Based on the recommendations of clinical experts, we determined the MCID threshold of 15% for preterm birth and 0.15 standardized MeanDiff improvement (ie, improvement of 0.15 SD) for continuous outcomes for the primary analysis. The MCID for birth weight outcome was calculated using the WHO Child Growth Standards (1 SD equals 474 g).^24^ Accordingly, we defined the MCID threshold for birth weight as follows: 474 g ×0.15 = 71.1 g. As sensitivity analyses, we considered the MCID thresholds of 10% and 20% for preterm birth and 0.10 and 0.20 standardized MeanDiff improvement for LAZ, HAZ, and mean birth weight (ie, 47.4 g and 94.8 g, respectively). These MCID thresholds have previously been used in the sample size calculations of several MNCH trials.^25,26,27,28,29,30,31,32,33,34^

Because we anticipated heterogeneity between different trials, we used random-effects models for our NMAs. We used empirically informative priors for the heterogeneity variance, as suggested by Rhodes et al^35^ for LAZ/HAZ and by Turner et al^36^ for preterm birth outcome, to stabilize the estimation of heterogeneity in the face of the low number of trials per comparison in the network. We used noninformative priors for mean birth weight outcome. We considered different random-effects model options with or without baseline adjustments or meta-regression based on baseline characteristics (eg, age of children and mothers). Our model selection was informed by the deviance information criterion (DIC) and the deviance-leverage plots that could help identify outliers or lack of model fit. The final model selection was done according to recommendations of the NICE TSD2.^23^

Across the 3 early-life periods, our primary analyses included both cluster and individually randomized RCTs. Within the cluster trials included in our NMA, a mean value of intracluster correlation coefficient (ICC) of 0.0505 was reported,^25,26,27,29,30,31,37,38,39,40,41,42,43,44^ so we assumed a conservative value of 0.05 to adjust for the clustering consequences of the cluster trials in our analyses. The ICC was used to inflate variance accordingly for the continuous outcome and to down-adjust the sample sizes and the number of cases for the dichotomous outcome, as recommended by Uhlmann et al.^45^ We conducted sensitivity analyses for each outcome by excluding cluster RCTs. We conducted NMAs for each life period in a software program (R; The R Project for Statistical Computing) using the R2WinBUGS 14 package.^46,47^ Full details of our statistical approaches, including inconsistency, are provided in the eAppendix in the Supplement.

## Results

Among 302 061 participants comprised of 169 randomized clinical trials, the network meta-analyses found several nutritional interventions that demonstrated greater association with improved birth and growth outcomes compared with standard of care. For instance, compared with standard of care, maternal supplements of multiple micronutrients (MMN) showed reduced odds for preterm birth (OR, 0.54; 95% CrI, 0.27-0.97) and improved mean birth weight (MeanDiff, 0.08 kg; 95% CrI, 0.00-0.17 kg) but not LAZ during EBF (MeanDiff, −0.02; 95% CrI, −0.18 to 0.14). Supplementing infants and children with MMN showed improved LAZ (MeanDiff, 0.20; 95% CrI, 0.03-0.35) and HAZ (MeanDiff, 0.14; 95% CrI, 0.02-0.25). The study found that pregnancy interventions generally had higher MCID probabilities than the interventions for the EBFor CF in the first 1000 days of life.

Our systematic search yielded publications that included 302 061 participants, of which 205 867 were pregnant women, 32 320 were mother-infant dyads, and 63 874 were children aged 6 to 12 months. The systematic search of databases and hand searching of bibliographies of published review articles yielded 22 738 abstracts (eFigure 2 in the Supplement). Of these abstracts, 1072 studies underwent full-text review, resulting in 235 articles reporting on 169 trials (87 for pregnancy, 27 for EBF, and 68 for CF) that met our inclusion criteria. The participants were randomized to 461 arms. This included 29 cluster RCTs that randomized 4207 clusters (182 421 participants) to 88 intervention arms. A list of the final included studies (eTables 8-10 in the Supplement), the excluded studies (eTables 11-13 in the Supplement), the associated trial characteristics (eTables 14, 15, and 16 in the Supplement), and participant characteristics (eTables 17-19 in the Supplement) can be found online. Studies were located predominantly in the following geographic regions: South East Asia (n = 89), Africa (n = 72), and South America (n = 27). In all 3 life periods, micronutrient supplementation (n = 133 of 169) was the most common intervention domain investigated (76 of 87 for pregnancy, 10 of 27 for EBF, and 47 of 68 for CF). A bias assessment was completed for all included trials (eTable 20 in the Supplement). For all domains except “Blinding of participants and personnel (performance bias),” more than 90% of studies had either low or unclear bias.

### Interventions for Women During Pregnancy

**Figure 2.**
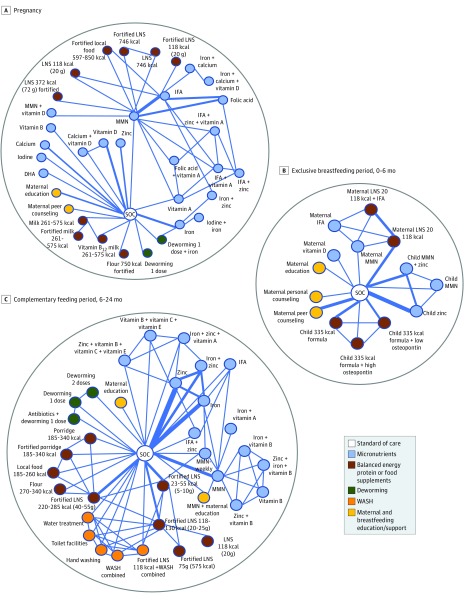
Overall Network Diagrams of Interventions for Pregnancy, Exclusive Breastfeeding Period, and Complementary Feeding Period Life Stages A-C, Each node represents an intervention, with each line representing a direct comparison between interventions. The line width represents the numbers of trials with the relevant comparison. DHA indicates docosahexaenoic acid; IFA, iron + folic acid; LNS, lipid-based nutrient supplements; MMN, multiple micronutrients; SOC, standard of care; and WASH, water, sanitation, and hygiene.

The network of evidence diagram for pregnancy interventions is shown in Figure 2A. We summarize the results of several key interventions for this life period in Table 2, and the full results of our analysis are shown in eFigures 3A, 3B, 5A, and 5B in the Supplement. The model diagnostics are provided in eFigures 3C, 3D, 3E, and 3F in the Supplement. Compared with standard of care, MMN supplements demonstrated reduced odds of preterm birth. No important differences in preterm birth were observed for the other interventions.

**Table 2.** Random-Effects Network Meta-analysis Results of Selected Key Interventions Across the 3 Life Periods^a^

Outcome	Pregnancy		Exclusive Breastfeeding Period, LAZ, MeanDiff	Complementary Feeding Period, HAZ, MeanDiff
	Preterm Birth, OR (95% CI)	Birth Weight, MeanDiff (95% CI), kg		
**Micronutrients vs Standard of Care**				
MMN (maternal)	0.54 (0.27 to 0.97)^b^	0.08 (0.00 to 0.17)^b^	−0.02 (−0.18 to 0.14)^b^	NA
MMN (child)	NA	NA	0.20 (0.03 to 0.35)	0.14 (0.02 to 0.25)
Iron + folic acid	0.59 (0.30 to 1.07)^b^	0.05 (−0.04 to 0.13)^b^	0.05 (−0.15 to 0.23)^b^	0.18 (0.05 to 0.30)
Zinc	0.53 (0.28 to 0.93)^b^	0.12 (0.06 to 0.18)^b^	0.12 (−0.03 to 0.24)	−0.02 (−0.08 to 0.04)
Iron + calcium	0.16 (0.03 to 0.87)^b^	0.15 (0.02 to 0.28)^b^	NA	NA
Iron + zinc	0.56 (0.28 to 1.05)^b^	0.08 (0.00 to 0.17)^b^	NA	−0.04 (−0.13 to 0.05)
Iron	0.55 (0.31 to 0.90)^b^	0.09 (0.03 to 0.15)^b^	NA	0.05 (−0.04 to 0.15)
Folic acid	0.61 (0.30 to 1.13)^b^	0.05 (−0.03 to 0.14)^b^	NA	NA
Calcium	0.76 (0.56 to 0.98)^b^	0.03 (−0.01 to 0.07)^b^	NA	NA
**Balanced Energy Protein or Food Supplements vs Standard of Care**				
LNS 118 kcal (20 g)	0.58 (0.27 to 1.14)^b^	0.11 (0.02 to 0.21)^b^	NA	−0.04 (−0.19 to 0.11)
Fortified LNS 118-130 kcal (20-25 g)	0.56 (0.25 to 1.16)^b^	0.09 (−0.02 to 0.20)^b^	0.08 (−0.13 to 0.29)^b^	−0.03 (−0.11 to 0.04)
Fortified LNS 220-285 kcal (40-50 g)	NA	NA	NA	0.01 (−0.09 to 0.07)
Fortified LNS 372 kcal (72 g)	0.64 (0.29 to 1.32)^b^	0.10 (−0.02 to 0.21)^b^	NA	NA
LNS 746 kcal	0.24 (0.04 to 1.09)^b^	NA	NA	NA
Flour 270-340 kcal	NA	NA	NA	0.05 (−0.08 to 0.19)
Fortified flour 750 kcal	0.25 (0.03 to 1.66)^b^	0.05 (−0.07 to 0.18)^b^	NA	NA
Local food 185-260 kcal	NA	NA	NA	0.04 (−0.10 to 0.18)
Fortified local food 597-850 kcal	NA	0.08 (−0.03 to 0.18)^b^	NA	NA
Formula 335 kcal	NA	NA	0.05 (−0.20 to 0.30)	NA
**Deworming, WASH Interventions, and Maternal Education vs Standard of Care**				
Deworming 1 dose	0.85 (0.21 to 3.23)^b^	0.02 (−0.02 to 0.07)^b^	NA	−0.01 (−0.13 to 0.10)
Maternal education	NA	0.04 (−0.12 to 0.20)^b^	0.05 (−0.12 to 0.23)^b^	−0.10 (−0.26 to 0.03)^b^
WASH	NA	NA	NA	−0.06 (−0.19 to 0.06)
WASH + fortified LNS 118 kcal (20 g)	NA	NA	NA	0.02 (−0.10 to 0.14)

Abbreviations: HAZ, height for age; LAZ, length for age; LNS, lipid-based nutrient supplements; MeanDiffs, mean differences; MMN, multiple micronutrients; NA, not available; OR, odds ratio; WASH, water, sanitation, and hygiene.

^a^ Each cell represents the estimated comparative result (ORs or MeanDiffs and their respective 95% CIs) vs standard of care from the primary analysis that included both cluster and noncluster randomized clinical trials.

^b^ Interventions that were provided to mothers and those cells without this superscript letter indicate interventions that were provided to children.

For mean birth weight, the following resulted in modest improvements relative to standard of care: iron, iron plus calcium, calcium plus vitamin D, zinc, iron plus folic acid (IFA) plus vitamin A, MMN, a single dose of deworming plus iron, and 118 kcal (20 g) of lipid-based nutrient supplementation (LNS 118kcal). No important differences were observed for the remaining interventions for birth weight.

### Interventions for Mothers and Infants During EBF (0-6 Months)

The network diagram of evidence for the EBF is shown in Figure 2B. The results of our NMA for key interventions for mothers and infants during EBF life period are listed in Table 2, and the full results of our analysis are shown in eFigures 3G and 4C in the Supplement. The model diagnostic of the EBF NMA is shown in eFigures 3H and 3I in the Supplement. Compared with standard of care, supplementing mothers with MMN did not show improvements for LAZ, but supplementing infants directly with MMN showed improvements in LAZ. No other interventions led to improvements.

### Interventions for Children During CF Period (6-24 Months)

The network for the CF is shown in Figure 2C. The results regarding the key interventions for this life period are summarized in Table 2 (with the full results shown in eFigure 3J and eFigure 4D in the Supplement). The model diagnostic of the NMA for the CF is shown in eFigures 3K and 3L in the Supplement. Micronutrient supplementations of IFA and MMN showed increased associations with improvements in HAZ. Food supplements, deworming, maternal education, and WASH did not show a positive association with HAZ.

### Assessment of Intervention Domains Across the First 1000 Days of Life

**Figure 3.**
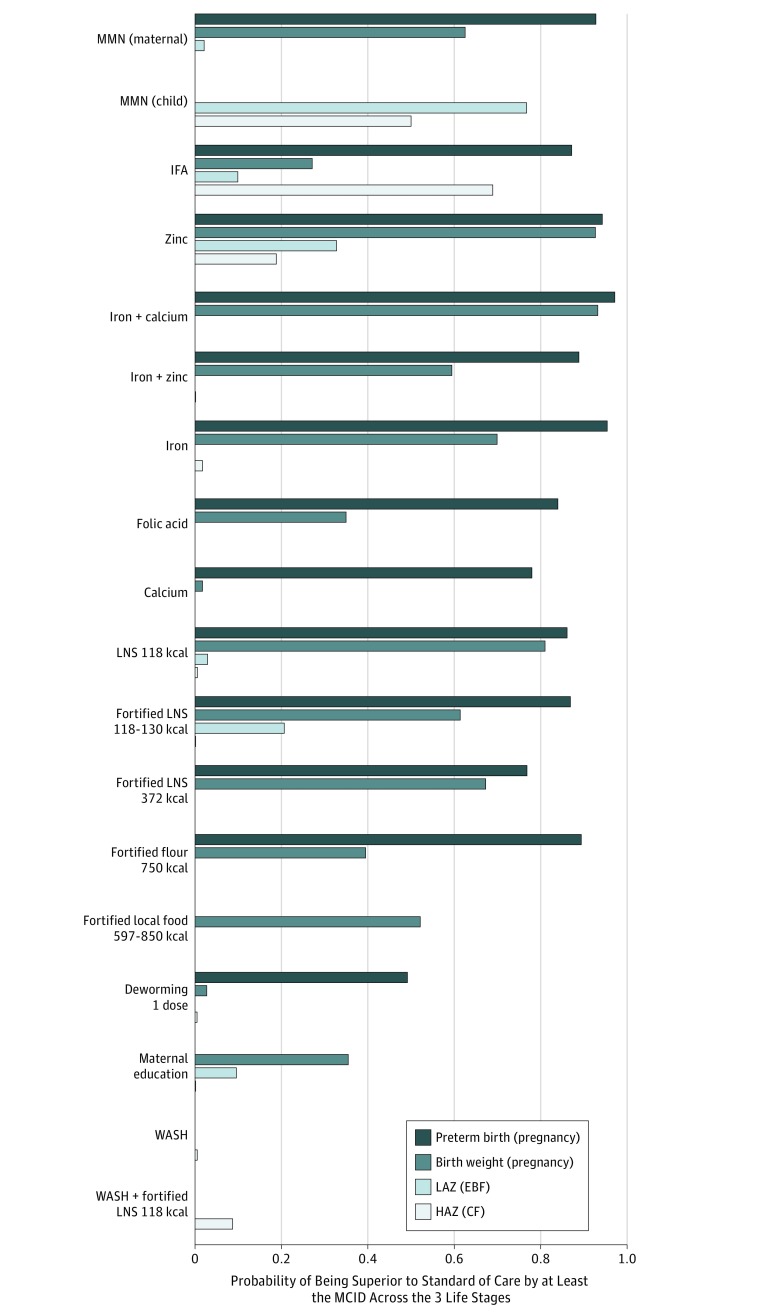
Probability of Key Interventions Under the Domains of Micronutrients, Balanced Energy Protein and Food Supplements, Deworming, and Maternal Education, With Water, Sanitation, and Hygiene (WASH) Being Superior to Standard of Care by at Least the Minimal Clinically Importance Difference (MCID) Across the 3 Life Stages Shown is the probability of interventions being considered superior by an MCID vs standard of care (SOC). The MCID threshold is defined in the Statistical Analysis subsection of the Methods section. These selected interventions had comparative effect sizes (vs SOC) of a promising magnitude. Full MCID probability results are provided in the eAppendix in the Supplement. CF indicates complementary feeding period (6-24 months); EBF, exclusive breastfeeding period (0-6 months); HAZ, height for age; IFA, iron + folic acid; LAZ, length for age; LNS, lipid-based nutrient supplements; and MMN, multiple micronutrients.

Posterior probabilities of key interventions being superior to standard of care by at least the predefined MCID for preterm birth, mean birth weight, LAZ, and HAZ are shown in Figure 3. The full MCID results are available online (eFigure 5A-D and eFigure 6A-D in the Supplement). Among the micronutrient supplements domain, several maternal micronutrient supplements during pregnancy life period demonstrated high probabilities of achieving an MCID for preterm birth, and fewer interventions demonstrated high probabilities of achieving an MCID for birth weight. For both outcomes of preterm birth and mean birth weight, maternal supplements of zinc and iron plus calcium showed high MCID probabilities. Other supplements, such as MMN, IFA, iron plus zinc, iron, and folic acid, showed inconsistent associations.

Similar to micronutrients, several balanced energy and protein supplements for pregnant women demonstrated higher probabilities of achieving an MCID consequence for preterm birth than mean birth weight, but their MCID probabilities were generally lower than those of the micronutrients. Maternal supplements of unfortified LNS 118 kcal had higher MCID probabilities for preterm birth and mean birth weight than fortified LNS 118-130 kcal, fortified LNS 372 kcal, and fortified flour 750 kcal. Fortified local food 597-850 kcal showed higher MCID probability for preterm birth, but its MCID probability for mean birth weight was low. Deworming and maternal education interventions showed low MCID probabilities.

After birth, interventions under all domains generally did not show high MCID probabilities for either LAZ or HAZ. During the EBF period, maternal MMN and IFA supplements showed low MCID probabilities for LAZ. Provision of MMN directly to infants during this life period showed a slightly higher MCID probability. For the CF stage, IFA and MMN showed above-average MCID probabilities for HAZ. All other interventions were associated with low probabilities for this life period.

### Sensitivity Analyses

For all of the 3 life periods, the results of the sensitivity analyses based on only individual RCTs were generally similar to the primary analyses. The results from these sensitivity analyses can be found in eFigure 4A to D and eFigure 6A to D in the Supplement. Because fewer studies were available, the 95% CrIs for many comparisons became wider. However, the magnitudes and directions of associations remained stable. For instance, the micronutrient supplements that demonstrated reduced ORs in the primary analysis (eg, MMN, iron, calcium, zinc, and iron plus calcium) also showed similar trends in the sensitivity analysis but with wider 95% CrIs.

Moreover, the sensitivity analyses with varying MCID thresholds generally showed similar trends as the primary analyses. When a lower threshold was used for the analyses, the MCID probabilities became larger for all interventions (eFigures 7A, 7C, 7E, and 7G in the Supplement), and the MCID probabilities became lower with higher MCID thresholds (eFigures 7B, 7D, 7F, and 7H in the Supplement). Across different life periods, the pregnancy interventions still showed higher MCID probabilities with either lower and higher MCID thresholds than the interventions of the later life periods.

## Discussion

In this study, we sought to comprehensively acquire, assess, and synthesize evidence to better understand the magnitude of associations between a range of interventions on birth and linear growth outcomes for pregnant women, infants, and children living in LMICs. We conducted NMAs (one for each early-life period) to evaluate the magnitude of associations with preventive interventions under several domains, including micronutrients, balanced energy and protein and food supplements, maternal education, deworming, and WASH. We found several interventions that could improve birth outcomes and linear growth outcomes for infants and children living in LMICs. The MCID probabilities of interventions under the nutritional (micronutrient and food supplements) domains were generally greater than those of other domains. We also found that the magnitude of associations with interventions varies between different life periods. For instance, maternal supplementation of MMN during pregnancy was shown to be superior to standard of care, but provision of maternal MMN during the EBF period was not shown to be statistically superior to standard of care. Larger MCID probabilities were demonstrated for pregnancy compared with the life periods after birth, highlighting the importance of intervening early during the fetal growth.

Our results are largely comparable to previously published review articles in terms of the precision of effect size estimates and trends of the associations.^11,12,48^ However, in our analysis on preterm birth, we found an OR of 0.54 (95% CrI, 0.27-0.97) for MMN compared with standard of care. This appears to disagree with the findings of a recently published Cochrane review on MMN that found a relative risk of 0.95 (95% CI, 0.90-1.01) for preterm birth.^48^ However, in that Cochrane review, a pairwise comparison of MMN was made with “iron with or without folic acid,”^48^^(p8)^ whereas our NMA compared MMN against IFA and iron alone separately. Our network analysis showed similar associations with preterm birth as the Cochrane review, with MMN showing an OR of 0.90 (95% CrI, 0.78-1.01) compared with IFA and an OR of 1.01 (95% CrI, 0.54-1.82) compared with iron.

Some of the differences between our analyses and the previously published review articles can be attributed to the differences in PICOS criteria used to guide the study selection (eTable 1 in the Supplement). For instance, in our analyses for the CF period, we excluded trials with children older than 24 months because the nutritional needs of these older children are considerably different than those of the children in the CF period. Some of the other review articles also included studies that were not RCTs (eg, quasi-randomized and nonrandomized studies). As well, we adjusted for the clustering consequences of the cluster trials in our analyses, while other review articles that included cluster trials did not always report details on ICC adjustment.

### Strengths and Limitations

A key strength of our review article is the use of a life-course perspective that enables critical insights into the current evidence base, generating a better understanding of the consequences of these interventions across key stages of the early-life trajectory. For instance, growth rates can vary substantially across the early-life periods, with such variability being heavily influenced by biochemical, genetic, and pathophysiological processes alongside external, environmental factors.^49^ Indeed, there is some evidence that infants who are low birth weight or premature may experience a robust velocity of growth if appropriate management of nutrition is maintained.^50^ Therefore, following the associations of stunting interventions across these 3 key stages is crucial to determining the optimum timing for intervention implementation. In particular, our review article appears to indicate that earlier interventions are more often associated with greater benefits.

Limitations include the lack of repeated trials of some interventions or domains and the lack of long-term clinical trials across the different life periods from early trimester of pregnancy to the end of the CF period. Few studies evaluated interventions across all 3 life periods^26,30,43,51,52,53,54,55^ or across 2 early-life periods.^42,56,57,58,59,60^ Consequently, the long-term association of interventions with improved birth and linear growth could not be accounted for in our analysis. Further to this point was the substantial heterogeneity observed in the interventions themselves, as well as the duration of the interventions and the timing of outcome assessments, particularly during the EBF and CF.

In relation to the scope of the current evidence, the evidence base was limited pertaining to maternal education, deworming, and WASH. A potential explanation could be the lack of focus on such interventions because there are a number of associated design and resource challenges. These include difficulties creating standardized interventions, heterogeneity in measuring baseline maternal education and other socioeconomic status levels, as well as cost and human resource limitations. The small number of studies reporting on these interventions may have had a role in the less convincing results for these outcomes.

We opted to focus on micro-level interventions after reviewing the bibliographies of existing systematic reviews (eTable 1 in the Supplement) and key MNCH articles^12,61^ because the RCT evidence base for macro-level approaches (eg, family planning and vocational training) was limited. However, we recognize the need for more investigation into these approaches to gain a better understanding of the social processes that contribute to linear growth faltering.^5^

In addition, few interventions have been assessed across 2 or more life periods, and there is a paucity of information on the consequences of combining interventions. While MMN is a combination of nutritional interventions, the component interventions potentially driving the results are, as yet, unknown. More trial research is needed to investigate the long-term and additive consequences of individual components of interventions.

Generally, RCTs are known for being a costly, time and resource–intensive approach for determining treatment effectiveness. Most trials herein used a conventional trial approach with a fixed sample size design, where the assessment of effectiveness occurred only after the number of participants recruited reached the calculated sample size target. However, it is important to point out that the degree of clinical equipoise that could justify the initial trial design decreases as data accumulate during the trial. Applying a conventional trial approach to investigate several treatments across extended periods is often not efficient. While more clinical trials are needed to improve the quality of evidence, future trials will also require more efficient clinical trial designs. To achieve the 40% reduction target set forth by the World Health Assembly^62^ to reduce the number of children with stunted growth younger than 5 years by 2025, it is necessary and important to ensure that our assessment of interventions is comprehensive and appropriate for different settings.

## Conclusions

The findings of our study highlight the importance of intervening early to improve birth outcomes and counter childhood stunting. Our findings suggest that nutritional interventions, micronutrients, and food supplements generally showed greater associations with improved outcomes than interventions from other domains. Despite the numerous clinical trials that have already been conducted, more research targeting less explored areas, such as maternal education and WASH, appears to be needed. We believe that additional research that combines multiple intervention domains will also prove valuable for critical issues in global child development.

## Notes

Supplement. **eTable 1.** Existing Reviews on Interventions for First 1,000 Days of Life: Pregnancy, Exclusive Breastfeeding Period, and Complementary Feeding Period **eTable 2.** Search Strategy for Pregnancy: Cochrane Central Register of Controlled Trials (CENTRAL) **eTable 3.** Search Strategy for Pregnancy: Embase **eTable 4.** Search Strategy for Pregnancy: Medline **eTable 5.** Search Strategy for Children Life Stages: CENTRAL **eTable 6.** Search Strategy for Children Life Stages: Embase **eTable 7.** Search Strategy for Children Life Stages: Medline **eTable 8.** List of Included Studies for Pregnancy Life Stage **eTable 9.** List of Included Studies for Exclusive Breastfeeding Life Stage (0-6m) **eTable 10.** List of Included Studies for Complementary Feeding Life Stage (6-24m) **eTable 11.** List of Excluded Studies for Pregnancy Life Stage With Reasons **eTable 12.** List of Excluded Studies for Exclusive Breastfeeding Stage With Reasons **eTable 13.** List of Excluded Studies for Complementary Feeding Stage With Reasons **eTable 14.** Trial Characteristics for Pregnancy Life Stage **eTable 15.** Trial Characteristics for Principal Systematic Review, Exclusive Breastfeeding Life Stage **eTable 16.** Trial Characteristics for Principal Systematic Literature Review, Complementary Feeding Stage **eTable 17.** Patient Characteristics for Pregnancy Life Stage Review **eTable 18.** Patient Characteristics for Exclusive Breastfeeding Life Stage Review **eTable 19.** Patient Characteristics for Complementary Feeding Life Stage Review **eTable 20.** Risk of Bias Assessment for the Included Studies **eTable 21.** MCMC Summary for Preterm Birth NMA **eTable 22.** MCMC Summary for Mean Birthweight NMA **eTable 23.** MCMC Summary for Length-for-Age (LAZ) NMA **eTable 24.** MCMC Summary for Height-for-Age (HAZ) NMA **eAppendix.** Supplemental Appendix **eFigure 1.** An Overview of the Literature Review Strategy **eFigure 2.** Study Flow Diagram **eFigure 3A.** Preterm Birth Forest Plot (Odds Ratio) for Pregnancy, Primary Analysis (Cluster and Non-Cluster Trials) **eFigure 3B.** Birthweight Forest Plot (Mean Difference) for Pregnancy, Primary Analysis (Cluster and Non-Cluster Trials) **eFigure 3C.** LAZ Forest Plot (Mean Difference) for Exclusive Breastfeeding Period, Primary Analysis (Cluster and Non-Cluster Trials) **eFigure 3D.** HAZ Forest Plot (Mean Difference) for Complementary Feeding Period, Primary Analysis (Cluster and Non-Cluster Trials) **eFigure 3E.** Leverage Plot of Mean Birthweight NMA (Pregnancy), Primary Analysis (Cluster and Non-Cluster Trials) **eFigure 3F.** Consistency Check of Mean Birthweight NMA (Pregnancy), Primary Analysis (Cluster and Non-Cluster Trials) **eFigure 3G.** LAZ Forest Plot (Mean Difference) for Exclusive Breastfeeding Period, Primary Analysis (Cluster and Non-Cluster Trials) **eFigure 3H.** Leverage Plot of LAZ NMA (Exclusive Breastfeeding Period), Primary Analysis (Cluster and Non-Cluster Trials) **eFigure 3I.** Consistency Check of LAZ NMA (Exclusive Breastfeeding Period), Primary Analysis (Cluster and Non-Cluster Trials) **eFigure 3J.** HAZ Forest Plot (Mean Difference) for Complementary Feeding Period, Primary Analysis (Cluster and Non-Cluster Trials) **eFigure 3K.** Leverage Plot of HAZ NMA (Complementary Feeding Period), Primary Analysis (Cluster and Non-Cluster Trials) **eFigure 3L.** Consistency Check of HAZ NMA (Complementary Feeding Period), Primary Analysis (Cluster and Non-Cluster Trials) **eFigure 4A.** Preterm Birth Forest Plot (Odds Ratio) for Pregnancy, Sensitivity Analysis (Non-Cluster Trials Only) **eFigure 4B.** Birthweight Forest Plot (Mean Difference) for Pregnancy, Sensitivity Analysis (Non-Cluster Trials Only) **eFigure 4C.** LAZ Forest Plot (Mean Difference) for Exclusive Breastfeeding Period, Sensitivity Analysis (Non-Cluster Trials Only) **eFigure 4D.** HAZ Forest Plot (Mean Difference) for Complementary Feeding Period, Sensitivity Analysis (Non-Cluster Trials Only) **eFigure 5A.** Probability of Being Superior to SOC by at Least the MCID for Pregnancy, Preterm Birth, Primary Analysis (Cluster And Non-Cluster Trials) **eFigure 5B.** Probability of Being Superior to SOC by at Least the MCID for Pregnancy, Mean Birthweight, Primary Analysis (Cluster And Non-Cluster Trials) **eFigure 5C.** Probability of Being Superior to SOC by at Least the MCID for EBF, LAZ, Primary Analysis (Cluster And Non-Cluster Trials) **eFigure 5D.** Probability of Being Superior to SOC by at Least the MCID for CFP, HAZ, Primary Analysis (Cluster And Non-Cluster Trials) **eFigure 6A.** Probability of Being Superior to SOC by at Least the MCID for Pregnancy, Preterm Birth, Sensitivity Analysis (Non-Cluster Trials Only) **eFigure 6B.** Probability of Being Superior to SOC by at Least the MCID for Pregnancy, Mean Birthweight, Sensitivity Analysis (Non-Cluster Trials Only) **eFigure 6C.** Probability of Being Superior to SOC by at Least the MCID for EBF, LAZ, Sensitivity Analysis (Non-Cluster Trials Only) **eFigure 6D.** Probability of Being Superior to SOC by at Least the MCID for CFP, HAZ, Sensitivity Analysis (Non-Cluster Trials Only) **eFigure 7A.** Probability of Being Superior to SOC by at Least the Lower MCID, the Preterm Birth NMA With Cluster and Non-Cluster Trials **eFigure 7B.** Probability of Being Superior to SOC by at Least the Higher MCID, the Preterm Birth NMA With Cluster and Non-Cluster Trials **eFigure 7C.** Probability of Being Superior to SOC by at Least the Lower MCID, the Mean Birthweight NMA With Cluster and Non-Cluster Trials **eFigure 7D.** Probability of Being Superior to SOC by at Least the Higher MCID, the Mean Birthweight NMA With Cluster and Non-Cluster Trials **eFigure 7E.** Probability of Being Superior to SOC by at Least the Lower MCID, the LAZ NMA With Cluster and Non-Cluster Trials **eFigure 7F.** Probability of Being Superior to SOC by at Least the Higher MCID, the LAZ NMA With Cluster and Non-Cluster Trials **eFigure 7G.** Probability of Being Superior to SOC by at Least the Lower MCID, the HAZ NMA With Cluster and Non-Cluster Trials **eFigure 7H.** Probability of Being Superior to Soc by at Least the Higher MCID, the HAZ NMA With Cluster and Non-Cluster Trials
