# Supplementary material for: Association of Early Interventions With Birth Outcomes and Child Linear Growth in Low-Income and Middle-Income Countries: Bayesian Network Meta-analyses of Randomized Clinical Trials
Source: JAMA Netw Open. 2019 Jul 26;2(7):e197871. doi: 10.1001/jamanetworkopen.2019.7871 (PMC6661710; doi:10.1001/jamanetworkopen.2019.7871)
Supplement: Supplement. — eTable 1. Existing Reviews on Interventions for First 1,000 Days of Life: Pregnancy, Exclusive Breastfeeding Period, and Complementary Feeding Period eTable 2. Search Strategy for Pregnancy: Cochrane Central Register of Controlled Trials (CENTRAL) eTable 3. Search Strategy for Pregnancy: Embase eTable 4. Search Strategy for Pregnancy: Medline eTable 5. Search Strategy for Children Life Stages: CENTRAL eTable 6. Search Strategy for Children Life Stages: Embase eTable 7. Search Strategy for Children Life Stages: Medline eTable 8. List of Included Studies for Pregnancy Life Stage eTable 9. List of Included Studies for Exclusive Breastfeeding Life Stage (0-6m) eTable 10. List of Included Studies for Complementary Feeding Life Stage (6-24m) eTable 11. List of Excluded Studies for Pregnancy Life Stage With Reasons eTable 12. List of Excluded Studies for Exclusive Breastfeeding Stage With Reasons eTable 13. List of Excluded Studies for Complementary Feeding Stage With Reasons eTable 14. Trial Characteristics for Pregnancy Life Stage eTable 15. Trial Characteristics for Principal Systematic Review, Exclusive Breastfeeding Life Stage eTable 16. Trial Characteristics for Principal Systematic Literature Review, Complementary Feeding Stage eTable 17. Patient Characteristics for Pregnancy Life Stage Review eTable 18. Patient Characteristics for Exclusive Breastfeeding Life Stage Review eTable 19. Patient Characteristics for Complementary Feeding Life Stage Review eTable 20. Risk of Bias Assessment for the Included Studies eTable 21. MCMC Summary for Preterm Birth NMA eTable 22. MCMC Summary for Mean Birthweight NMA eTable 23. MCMC Summary for Length-for-Age (LAZ) NMA eTable 24. MCMC Summary for Height-for-Age (HAZ) NMA eAppendix. Supplemental Appendix eFigure 1. An Overview of the Literature Review Strategy eFigure 2. Study Flow Diagram eFigure 3A. Preterm Birth Forest Plot (Odds Ratio) for Pregnancy, Primary Analysis (Cluster and Non-Cluster Trials) eFigure 3B. Birthweight Fore [file jamanetwopen-2-e197871-s001.pdf]

Supplementary Online Content

Park JJH, Fang ML, Harari O, et al. Association of early interventions with birth outcomes and child linear growth in low-income and middle-income countries: Bayesian network meta-analyses of randomized clinical trials. *JAMA Netw Open*. 2019:2(7):e197871. doi:10.1001/jamanetworkopen.2019.7871

eTable 1. Existing Reviews on Interventions for First 1,000 Days of Life: Pregnancy, Exclusive Breastfeeding Period, and Complementary Feeding Period
eTable 2. Search Strategy for Pregnancy: Cochrane Central Register of Controlled Trials (CENTRAL)
eTable 3. Search Strategy for Pregnancy: Embase
eTable 4. Search Strategy for Pregnancy: Medline
eTable 5. Search Strategy for Children Life Stages: CENTRAL
eTable 6. Search Strategy for Children Life Stages: Embase
eTable 7. Search Strategy for Children Life Stages: Medline
eTable 8. List of Included Studies for Pregnancy Life Stage
eTable 9. List of Included Studies for Exclusive Breastfeeding Life Stage (0-6m)
eTable 10. List of Included Studies for Complementary Feeding Life Stage (6-24m)
eTable 11. List of Excluded Studies for Pregnancy Life Stage With Reasons
eTable 12. List of Excluded Studies for Exclusive Breastfeeding Stage With Reasons
eTable 13. List of Excluded Studies for Complementary Feeding Stage With Reasons
eTable 14. Trial Characteristics for Pregnancy Life Stage
eTable 15. Trial Characteristics for Principal Systematic Review, Exclusive Breastfeeding Life Stage
eTable 16. Trial Characteristics for Principal Systematic Literature Review, Complementary Feeding Stage
eTable 17. Patient Characteristics for Pregnancy Life Stage Review
eTable 18. Patient Characteristics for Exclusive Breastfeeding Life Stage Review
eTable 19. Patient Characteristics for Complementary Feeding Life Stage Review
eTable 20. Risk of Bias Assessment for the Included Studies
eTable 21. MCMC Summary for Preterm Birth NMA
eTable 22. MCMC Summary for Mean Birthweight NMA
eTable 23. MCMC Summary for Length-for-Age (LAZ) NMA
eTable 24. MCMC Summary for Height-for-Age (HAZ) NMA
eAppendix. Supplemental Appendix
eFigure 1. An Overview of the Literature Review Strategy
eFigure 2. Study Flow Diagram
eFigure 3A. Preterm Birth Forest Plot (Odds Ratio) for Pregnancy, Primary Analysis (Cluster and Non-Cluster Trials)
eFigure 3B. Birthweight Forest Plot (Mean Difference) for Pregnancy, Primary Analysis (Cluster and Non-Cluster Trials)
eFigure 3C. LAZ Forest Plot (Mean Difference) for Exclusive Breastfeeding Period, Primary Analysis (Cluster and Non-Cluster Trials)
eFigure 3D. HAZ Forest Plot (Mean Difference) for Complementary Feeding Period, Primary Analysis (Cluster and Non-Cluster Trials)
eFigure 3E. Leverage Plot of Mean Birthweight NMA (Pregnancy), Primary Analysis (Cluster and Non-Cluster Trials)
eFigure 3F. Consistency Check of Mean Birthweight NMA (Pregnancy), Primary Analysis (Cluster and Non-Cluster Trials)
eFigure 3G. LAZ Forest Plot (Mean Difference) for Exclusive Breastfeeding Period, Primary Analysis (Cluster and Non-Cluster Trials)
eFigure 3H. Leverage Plot of LAZ NMA (Exclusive Breastfeeding Period), Primary Analysis (Cluster and Non-Cluster Trials)
eFigure 3I. Consistency Check of LAZ NMA (Exclusive Breastfeeding Period), Primary Analysis (Cluster and Non-Cluster Trials)
eFigure 3J. HAZ Forest Plot (Mean Difference) for Complementary Feeding Period, Primary Analysis (Cluster and Non-Cluster Trials)
eFigure 3K. Leverage Plot of HAZ NMA (Complementary Feeding Period), Primary Analysis (Cluster and Non-Cluster Trials)
eFigure 3L. Consistency Check of HAZ NMA (Complementary Feeding Period), Primary Analysis (cluster and Non-Cluster Trials)
eFigure 4A. Preterm Birth Forest Plot (Odds Ratio) for Pregnancy, Sensitivity Analysis (Non-Cluster Trials Only)
eFigure 4B. Birthweight Forest Plot (Mean Difference) for Pregnancy, Sensitivity Analysis (Non-Cluster Trials Only)
eFigure 4C. LAZ Forest Plot (Mean Difference) for Exclusive Breastfeeding Period, Sensitivity Analysis (Non-Cluster Trials Only)
eFigure 4D. HAZ Forest Plot (Mean Difference) for Complementary Feeding Period, Sensitivity Analysis (Non-Cluster Trials Only)
eFigure 5A. Probability of Being Superior to SOC by at Least the MCID for Pregnancy, Preterm Birth, Primary Analysis (Cluster And Non-Cluster Trials)
eFigure 5B. Probability of Being Superior to SOC by at Least the MCID for Pregnancy, Mean Birthweight, Primary Analysis (Cluster And Non-Cluster Trials)
eFigure 5C. Probability of Being Superior to SOC by at Least the MCID for EBF, LAZ, Primary Analysis (Cluster And Non-Cluster Trials)
eFigure 5D. Probability of Being Superior to SOC by at Least the MCID for CFP, HAZ, Primary Analysis (Cluster And Non-Cluster Trials)
eFigure 6A. Probability of Being Superior to SOC by at Least the MCID for Pregnancy, Preterm Birth, Sensitivity Analysis (Non-Cluster Trials Only)
eFigure 6B. Probability of Being Superior to SOC by at Least the MCID for Pregnancy, Mean Birthweight, Sensitivity Analysis (Non-Cluster Trials Only)
eFigure 6C. Probability of Being Superior to SOC by at Least the MCID for EBF, LAZ, Sensitivity Analysis (Non-Cluster Trials Only)
eFigure 6D. Probability of Being Superior to SOC by at Least the MCID for CFP, HAZ, Sensitivity Analysis (Non-Cluster Trials Only)
eFigure 7A. Probability of Being Superior to SOC by at Least the Lower MCID, the Preterm Birth NMA With Cluster and Non-Cluster Trials
eFigure 7B. Probability of Being Superior to SOC by at Least the Higher MCID, the Preterm Birth NMA With Cluster and Non-Cluster Trials
eFigure 7C. Probability of Being Superior to SOC by at Least the Lower MCID, the Mean Birthweight NMA With Cluster and Non-Cluster Trials
eFigure 7D. Probability of Being Superior to SOC by at Least the Higher MCID, the Mean Birthweight NMA With Cluster and Non-Cluster Trials
eFigure 7E. Probability of Being Superior to SOC by at Least the Lower MCID, the LAZ NMA With Cluster and Non-Cluster Trials
eFigure 7F. Probability of Being Superior to SOC by at Least the Higher MCID, the LAZ NMA With Cluster and Non-Cluster Trials
eFigure 7G. Probability of Being Superior to SOC by at Least the Lower MCID, the HAZ NMA With Cluster and Non-Cluster Trials
eFigure 7H. Probability of Being Superior to Soc by at Least the Higher MCID, the HAZ NMA With Cluster and Non-Cluster Trials

This supplementary material has been provided by the authors to give readers additional information about their work.

© 2019 Park JJH et al. *JAMA Network Open*.

eTable 1. Existing reviews on interventions for first 1,000 days of life: Pregnancy, exclusive breastfeeding period, and complementary feeding period

Review ID	Title	Interventions	No of studies	Types of studies included
**Pregnancy**				
Imdad 2011[1]	Effect of balanced protein energy supplementation during pregnancy on birth outcomes	Balanced protein energy supplements	11	RCTs and quasi-RCTs
Imdad 2012[2]	Maternal Nutrition and Birth Outcomes: Effect of Balanced Protein-Energy Supplementation	Balanced protein energy supplements	16	RCTs and quasi-RCTs
Liberato 2013[3]	Effects of protein energy supplementation during pregnancy on fetal growth: a review of the literature focusing on contextual factors	Balanced protein energy supplements	20	RCTs, quasi-RCTs, and observational study
Stevens 2015[4]	The effect of balanced protein energy supplementation in undernourished pregnant women and child physical growth in low- and middle-income countries: a systematic review and meta-analysis	Balanced protein energy supplements	7	RCTs, quasi-RCTs, and observational study
Buppasiri 2015[5]	Calcium supplementation (other than for preventing or treating hypertension) for improving pregnancy and infant outcomes	Calcium	25	RCTs and cluster-RCTs
Hofmeyr 2014[6]	Calcium supplementation during pregnancy for preventing hypertensive disorders and related problems	Calcium	13	RCTs
Bassani 2013[7]	Financial incentives and coverage of child health interventions: a systematic review and meta-analysis	Conditional cash transfer	25	Non peer-reviewed institutional reports, RCTs, and observational studies
Glassman 2013[8]*	Impact of Conditional Cash Transfers on Maternal and Newborn Health	Conditional cash transfer	24	Cochrane reviews, systematic reviews, and other papers
Salam 2015[9]	Effect of administration of antihelminthics for soil-transmitted helminths during pregnancy	Deworming	4	RCTs
Lassi 2013[10]	Folic acid supplementation during pregnancy for maternal health and pregnancy outcomes	Folic acid	31	RCTs and cluster-RCTs
Yang 2011[11]*	Review of fortified food and beverage products for pregnant and lactating women and their impact on nutritional status	Fortified products	14	RCT, quasi-RCT
Bratton 2015[12]	Maternal Influenza Immunization and Birth Outcomes of Stillbirth and Spontaneous Abortion: A Systematic Review and Meta-analysis	Influenza vaccine	7	Observational and cross-sectional studies
Nunes 2016[13]	The Effects of Influenza Vaccination during Pregnancy on Birth Outcomes: A Systematic Review and Meta-Analysis	Influenza vaccine	18	RCTs and observational studies
Pena-Rosas 2015[14]	Effects and safety of preventive oral iron or iron+folic acid supplementation for women during pregnancy	Iron; Iron + folic acid	49	RCTs and quasi-RCTs
Suchdev 2015[15]	Multiple micronutrient powders for home (point-of-use) fortification of foods in pregnant women (Review)	Multiple micronutrient powders	2	RCTs
Haider 2017[16]	Multiple-micronutrient supplementation for women during pregnancy	Multiple micronutrient supplements	19	RCTs

© 2019 Park JJH et al. *JAMA Network Open*.

Review ID	Title	Interventions	No of studies	Types of studies included
Imhoff-Kunsch 2012[17]	Effect of n-3 Long-chain Polyunsaturated Fatty Acid Intake during Pregnancy on Maternal, Infant, and Child Health Outcomes: A Systematic Review	N-3 long chain polyunsaturated fatty acid	15	RCT
Thorne-Lyman 2012A[18]	Vitamin A and carotenoids during pregnancy and maternal, neonatal and infant health outcomes: a systematic review and meta-analysis	Vitamin A	17	RCTs
De-Regil 2016[19]	Vitamin D supplementation for women during pregnancy	Vitamin D	15	RCTs and quasi-RCTs
Perez-Lopez 2015[20]	Effect of vitamin D supplementation during pregnancy on maternal and neonatal outcomes: a systematic review and meta-analysis of randomized controlled trials	Vitamin D	13	RCTs
Thorne-Lyman 2012B[21]	Vitamin D during pregnancy and maternal, neonatal and infant health outcomes: a systematic review and meta-analysis	Vitamin D	5	RCTs
Ota 2015[22]	Zinc supplementation for improving pregnancy and infant outcome	Zinc	21	RCTs
**Exclusive breastfeeding life stage**				
Kramer 2012[23]	Optimal duration of exclusive breastfeeding (Review).	Exclusive breastfeeding vs complementary food introduction at 4 months	23	RCTs
Lumbiganon 2016[24]	Antenatal breastfeeding education for increasing breastfeeding duration.	Breastfeeding education for increasing breastfeeding duration	24	RCTs
Haroon 2013[25]	Breastfeeding promotion interventions and breastfeeding practices: a systematic review.	Breastfeeding education or support	110	RCTs and quasi-experimental studies
Balogun 2016[26]	Interventions for promoting the initiation of breastfeeding.	Breastfeeding education, support groups	28	RCTs
Giugliani 2015[27]	Effect of breastfeeding promotion interventions on child growth: a systematic review and meta-analysis.	Breastfeeding promoting interventions	35	RCTs
Abe 2016[28]	Supplementation with multiple micronutrients for breastfeeding women for improving outcomes for the mother and baby.	Micronutrients mothers	2	RCTs
Ndikom 2014[29]	Extra fluids for breastfeeding mothers for increasing milk production.	Forced fluids	1	RCTs
Martin 2016[30]	Review of Infant Feeding: Key Features of Breast Milk and Infant Formula.	Infant nutrition	6	RCTs
Fleith 2005[31]	Dietary PUFA for Preterm and Term Infants: Review of Clinical Studies	Infant nutrition	28	RCTs
Conde-Agudelo 2016[32]	Kangaroo mother care to reduce morbidity and mortality in low birthweight infants.	Kangaroo care	21	RCTs
Moore 2016[33]	Early skin-to-skin contact for mothers and their healthy newborn infants.	Kangaroo care	46	RCTs
Delgado-Noguera 2015[34]	Supplementation with long chain polyunsaturated fatty acids (LCPUFA) to breastfeeding mothers for improving child growth and development.	Long chain polyunsaturated fatty acids supplements	8	RCTs

© 2019 Park JJH et al. *JAMA Network Open*.

Review ID	Title	Interventions	No of studies	Types of studies included
Thiele 2013[35]	Maternal vitamin D supplementation to meet the needs of the breastfed infant: a systematic review.	Vitamin D supplements	3	RCTs
Becker 2016[36]	Methods of milk expression for lactating women.	Methods of lactation	41	RCTs
**Complementary feeding period**				
Dangour 2013[37]	Interventions to improve water quality and supply, sanitation and hygiene practices, and their effects on the nutritional status of children (Review)	WASH	14	RCTs, cluster-RCTs, quasi- and non-randomised trials, controlled cohort or cross-sectional studies and historically controlled studies
Darlow 2016[38]	Vitamin A supplementation to prevent mortality and short- and long-term morbidity in very low birth weight infants (Review)	Micronutrient: Vitamin A	10	RCTs
Das 2013[39]	Micronutrient fortification of food and its impact on woman and child health: a systematic review	Micronutrients	201	RCTs, quasi-experimental and before-after studies.
De-Regil 2011[40]	Intermittent iron supplementation for improving nutrition and development in children under 12 years of age (Review)	Micronutrient: Iron (intermittent)	33	RCTs and quasi-RCTs with either individual or cluster randomisation
De-Regil 2013[41]	Home fortification of foods with multiple micronutrient powders for health and nutrition in children under two years of age (Review)	Home fortification	8	RCTs or quasi-RCTs
Devakumar 2016[42]	Maternal antenatal multiple micronutrient supplementation for long-term health benefits in children: a systematic review and meta-analysis.	Micronutrient: MMS	9	RCTs, cluster-RCTs
Gaffey 2013[43]	Dietary management of childhood diarrhea in low- and middle-income countries: a systematic review.	Diet for diarrhea management	29	RCTs
Gough 2014[44]	The impact of antibiotics on growth in children in low and middle income countries: systematic review and meta-analysis of randomised controlled trials	Antibiotics	10	RCTs
Imdad 2011[45]	Effect of preventive zinc supplementation on linear growth in children under 5 years of age in developing countries: a meta-analysis of studies for input to the lives saved tool	Micronutrient: Zinc	36	RCTs
Imdad 2017[46]	Vitamin A supplementation for preventing morbidity and mortality in children from six months to five years of age (Review)	Micronutrient: Vitamin A	45	RCTs, Cluster-RCTs
Kristjansson 2015[47]	Food supplementation for improving the physical and psychosocial health of socio-economically disadvantaged children aged three months to five years	Food supplementation	26	RCTs and studies with historical controls
Lassi 2013[48]	Impact of complementary food and education on complementary food on growth and morbidity of children less than 2 years of age in developing countries: a systematic review	Complementary foods	16	RCTs, nonrandomized trials
Matsungo	Lipid-based nutrient supplements and linear growth in	Lipid	7	RCTs

© 2019 Park JJH et al. *JAMA Network Open*.

Review ID	Title	Interventions	No of studies	Types of studies included
2017[49]	children under 2 years: a review	supplements		
Mayo-Wilson 2014[50]	Zinc supplementation for preventing mortality, morbidity, and growth failure in children aged 6 months to 12 years of age (Review)	Micronutrient: Zinc	80	RCTs
Pasricha 2013[51]	Effect of daily iron supplementation on health in children aged 4-23 months: a systematic review and meta-analysis of randomised controlled trials.	Micronutrient: Iron	35	RCTs
Petry 2016[52]	The Effect of Low Dose Iron and Zinc Intake on Child Micronutrient Status and Development during the First 1000 Days of Life: A Systematic Review and Meta-Analysis.	Micronutrient: Iron + zinc	90	RCTs or quasi-RCTs
Salam 2013[53]	Effectiveness of micronutrient powders (MNP) in women and children	Micronutrient: Micronutrient powders	17	RCTs
Sguaseero 2012[54]	Community-based supplementary feeding for promoting the growth of children under five years of age in low and middle income countries (Review)	Community-based supplementary feeding	8	RCTs
Taylor-Robinson 2015[55]	Deworming drugs for soil-transmitted intestinal worms in children: effects on nutritional indicators, haemoglobin and school performance (Review)	Deworming	45	RCTs or quasi-RCTs

© 2019 Park JJH et al. *JAMA Network Open*.

Literature search strategy

eTable 2. Search strategy for pregnancy: Cochrane Central Register of Controlled Trials (CENTRAL)

Executed on August 14, 2018

No.	Terms	Hits	Comments
1	exp pregnancy/	18514	Population
2	exp Pregnancy Trimesters/ or exp Pregnancy Trimester, First/ or exp Pregnancy Trimester, Second/ or exp Pregnancy Trimester, Third/	1531	
3	exp Pregnant women/	149	
4	(Pregnant women or pregnant woman or pregnan* or gravid or obstetric or antenatal or antepartum or gestation).ti,ab.	28189	
5	or/1-4	34830	
6	Developing Countries/	702	LMIC terms
7	(Africa or Asia or Caribbean or West Indies or South America or Latin America or Central America).hw,ti,ab,cp.	7824	
8	(Afghanistan or Albania or Algeria or Angola or Antigua or Barbuda or Argentina or Armenia or Armenian or Aruba or Azerbaijan or Bahrain or Bangladesh or Barbados or Benin or Byelarus or Byelorussian or Belarus or Belorussian or Belorussia or Belize or Bhutan or Bolivia or Bosnia or Herzegovina or Hercegovina or Botswana or Brasil or Brazil or Bulgaria or Burkina Faso or Burkina Fasso or Upper Volta or Burundi or Urundi or Cambodia or Khmer Republic or Kampuchea or Cameroon or Cameroons or Cameron or Camerons or Cape Verde or Central African Republic or Chad or Chile or China or Colombia or Comoros or Comoro Islands or Comores or Mayotte or Congo or Zaire or Costa Rica or Cote d'Ivoire or Ivory Coast or Croatia or Cuba or Cyprus or Czechoslovakia or Czech Republic or Slovakia or Slovak Republic or Djibouti or French Somaliland or Dominica or Dominican Republic or East Timor or East Timur or Timor Leste or Ecuador or Egypt or United Arab Republic or El Salvador or Eritrea or Estonia or Ethiopia or Fiji or Gabon or Gabonese Republic or Gambia or Gaza or Georgia Republic or Georgian Republic or Ghana or Gold Coast or Greece or Grenada or Guatemala or Guinea or Guam or Guiana or Guyana or Haiti or Honduras or Hungary or India or Maldives or Indonesia or Iran or Iraq or Isle of Man or Jamaica or Jordan or Kazakhstan or Kazakh or Kenya or Kiribati or Korea or Kosovo or Kyrgyzstan or Kirghizia or Kyrgyz Republic or Kirghiz or Kirgizstan or Lao PDR or Laos or Latvia or Lebanon or Lesotho or Basutoland or Liberia or Libya or Lithuania or Macedonia or Madagascar or Malagasy Republic or Malaysia or Malaya or Malay or Sabah or Sarawak or Malawi or Nyasaland or Mali or Malta or Marshall Islands or Mauritania or Mauritius or Agalega Islands or Mexico or Micronesia or Middle East or Moldova or Moldovia or Moldovian or Mongolia or Montenegro or Morocco or Ifni or Mozambique or Myanmar or Myanma or Burma or Namibia or Nepal or Netherlands Antilles or New Caledonia or Nicaragua or Niger or Nigeria or Northern Mariana Islands or Oman or Muscat or Pakistan or Palau or Palestine or Panama or Paraguay or Peru or Philippines or Philipines or Phillipines or Phillippines or Poland or Portugal or Puerto Rico or Romania or Rumania or Roumania or Russia or Russian or Rwanda or Ruanda or Saint Kitts or St Kitts or Nevis or Saint Lucia or St Lucia or Saint Vincent or St Vincent or Grenadines or Samoa or Samoan Islands or Navigator Island or Navigator Islands or Sao Tome or Saudi Arabia or Senegal or Serbia or Montenegro or Seychelles or Sierra Leone or Slovenia or Sri Lanka or Ceylon or Solomon Islands or Somalia or South Africa or Sudan	76644	

© 2019 Park JJH et al. *JAMA Network Open*.

No.	Terms	Hits	Comments
	or Suriname or Surinam or Swaziland or Syria or Tajikistan or Tadzhikistan or Tadjikistan or Tadzhik or Tanzania or Thailand or Togo or Togolese Republic or Tonga or Trinidad or Tobago or Tunisia or Turkey or Turkmenistan or Turkmen or Uganda or Ukraine or Uruguay or USSR or Soviet Union or Union of Soviet Socialist Republics or Uzbekistan or Uzbek or Vanuatu or New Hebrides or Venezuela or Vietnam or Viet Nam or West Bank or Yemen or Yugoslavia or Zambia or Zimbabwe or Rhodesia).hw,ti,ab,cp.		
9	((developing or less* developed or under developed or underdeveloped or middle income or low* income or underserved or under served or deprived or poor*) adj (countr* or nation? or population? or world)).ti,ab.	4060	
10	((developing or less* developed or under developed or underdeveloped or middle income or low* income) adj (economy or economies)).ti,ab.	15	
11	(low* adj (gdp or gnp or gross domestic or gross national)).ti,ab.	39	
12	(low adj3 middle adj3 countr*).ti,ab.	528	
13	(lmic or lmics or third world or lami countr*).ti,ab.	167	
14	transitional countr*.ti,ab.	3	
15	or/6-14	81242	
16	5 and 15	5925	**Population + LMIC terms**
17	exp Micronutrients/	18026	Micronutrient and calcium supplements
18	(Vitamin* or provitamin* or mineral* or trace element* or provitamin* or vitamin*A or vitamin*B or vitamin*B1 or vitamin*B2 or vitamin*B6 or vitamin*B12 or niacin or vitamin*C or vitamin*D or vitamin*E or folic acid or iron or IFA or zinc or copper or selenium or iodine or calcium or MMN or multi*vitamin* or multiple micronutrient or magnesium).mp.	61133	
19	exp Dietary Supplements/	9748	Food supplements
20	(fortified food or food fortified or fortified, food or dietary product* or dietary food* or specialized food or foods, specialized or dietary supplement* or supplement, dietary or supplements, dietary or food supplement* or supplement, food or supplements, food or lipid based supplement or lipid supplement or soy based supplement or soy supplement or balanced energy protein supplement).mp.	11316	
21	exp Anthelmintics/	1387	Deworming
22	(Albendazole or mebendazole or praziquantel or ivermectin or benzimidazole or praziquantel).mp.	1388	
23	exp Prenatal education/	26	Education
24	(Maternal education or mother education or education, maternal or education, mother).mp.	226	
25	exp Sanitation/	346	WASH
26	(waste water management or drinking water or sanitation or sewage disposal or septic tank or latrine* or toilet* or hygiene or WASH or chlorine tablet* or hand washing or water store or potty or soap or detergent).mp.	9931	
27	or/17-26	84144	**Interventions combined**
28	16 and 27	1382	**Population and interventions combined**
29	limit 28 to english	1281	Limit to English only

© 2019 Park JJH et al. *JAMA Network Open*.

eTable 3. Search strategy for pregnancy: Embase

Executed on August 14, 2018

No.	Terms	Hits	Comments
1	exp pregnancy/	686717	Population
2	exp Pregnancy Trimester/ or exp Pregnancy Trimester, First/ or exp Pregnancy Trimester, Second/ or exp Pregnancy Trimester, Third/	63191	
3	exp Pregnant women/	59622	
4	(Pregnant women or pregnant woman or pregnan* or gravid or obstetric or antenatal or antepartum or gestation).ti,ab.	673630	
5	or/1-4	958906	
6	Developing Country.sh.	88669	LMIC terms
7	(Africa or Asia or Caribbean or West Indies or South America or Latin America or Central America).hw,ti,ab,cp.	301639	
8	(Afghanistan or Albania or Algeria or Angola or Antigua or Barbuda or Argentina or Armenia or Armenian or Aruba or Azerbaijan or Bahrain or Bangladesh or Barbados or Benin or Byelarus or Byelorussian or Belarus or Belorussian or Belorussia or Belize or Bhutan or Bolivia or Bosnia or Herzegovina or Hercegovina or Botswana or Brasil or Brazil or Bulgaria or Burkina Faso or Burkina Fasso or Upper Volta or Burundi or Urundi or Cambodia or Khmer Republic or Kampuchea or Cameroon or Cameroons or Cameron or Camerons or Cape Verde or Central African Republic or Chad or Chile or China or Colombia or Comoros or Comoro Islands or Comores or Mayotte or Congo or Zaire or Costa Rica or Cote d'Ivoire or Ivory Coast or Croatia or Cuba or Cyprus or Czechoslovakia or Czech Republic or Slovakia or Slovak Republic or Djibouti or French Somaliland or Dominica or Dominican Republic or East Timor or East Timur or Timor Leste or Ecuador or Egypt or United Arab Republic or El Salvador or Eritrea or Estonia or Ethiopia or Fiji or Gabon or Gabonese Republic or Gambia or Gaza or Georgia Republic or Georgian Republic or Ghana or Gold Coast or Greece or Grenada or Guatemala or Guinea or Guam or Guiana or Guyana or Haiti or Honduras or Hungary or India or Maldives or Indonesia or Iran or Iraq or Isle of Man or Jamaica or Jordan or Kazakhstan or Kazakh or Kenya or Kiribati or Korea or Kosovo or Kyrgyzstan or Kirghizia or Kyrgyz Republic or Kirghiz or Kirgizstan or Lao PDR or Laos or Latvia or Lebanon or Lesotho or Basutoland or Liberia or Libya or Lithuania or Macedonia or Madagascar or Malagasy Republic or Malaysia or Malaya or Malay or Sabah or Sarawak or Malawi or Nyasaland or Mali or Malta or Marshall Islands or Mauritania or Mauritius or Agalega Islands or Mexico or Micronesia or Middle East or Moldova or Moldovia or Moldovian or Mongolia or Montenegro or Morocco or Ifni or Mozambique or Myanmar or Myanma or Burma or Namibia or Nepal or Netherlands Antilles or New Caledonia or Nicaragua or Niger or Nigeria or Northern Mariana Islands or Oman or Muscat or Pakistan or Palau or Palestine or Panama or Paraguay or Peru or Philippines or Philipines or Phillipines or Phillippines or Poland or Portugal or Puerto Rico or Romania or Rumania or Roumania or Russia or Russian or Rwanda or Ruanda or Saint Kitts or St Kitts or Nevis or Saint Lucia or St Lucia or Saint Vincent or St Vincent or Grenadines or Samoa or Samoan Islands or	3765601	

© 2019 Park JJH et al. *JAMA Network Open*.

No.	Terms	Hits	Comments
	Navigator Island or Navigator Islands or Sao Tome or Saudi Arabia or Senegal or Serbia or Montenegro or Seychelles or Sierra Leone or Slovenia or Sri Lanka or Ceylon or Solomon Islands or Somalia or South Africa or Sudan or Suriname or Surinam or Swaziland or Syria or Tajikistan or Tadzhikistan or Tadjikistan or Tadzhik or Tanzania or Thailand or Togo or Togolese Republic or Tonga or Trinidad or Tobago or Tunisia or Turkey or Turkmenistan or Turkmen or Uganda or Ukraine or Uruguay or USSR or Soviet Union or Union of Soviet Socialist Republics or Uzbekistan or Uzbek or Vanuatu or New Hebrides or Venezuela or Vietnam or Viet Nam or West Bank or Yemen or Yugoslavia or Zambia or Zimbabwe or Rhodesia).hw,ti,ab,cp.		
9	((developing or less* developed or under developed or underdeveloped or middle income or low* income or underserved or under served or deprived or poor*) adj (countr* or nation? or population? or world)).ti,ab.	101323	
10	((developing or less* developed or under developed or underdeveloped or middle income or low* income) adj (economy or economies)).ti,ab.	541	
11	(low* adj (gdp or gnp or gross domestic or gross national)).ti,ab.	309	
12	(low adj3 middle adj3 countr*).ti,ab.	11270	
13	(lmic or lmics or third world or lami countr*).ti,ab.	6366	
14	transitional countr*.ti,ab.	199	
15	or/6-14	3958832	
16	5 and 15	161216	**Population and LMIC terms combined**
17	exp Micronutrients/	35349	Micronutrient and calcium supplements
18	(Vitamin* or provitamin* or mineral* or trace element* or provitamin* or vitamin*A or vitamin*B or vitamin*B1 or vitamin*B2 or vitamin*B6 or vitamin*B12 or niacin or vitamin*C or vitamin*D or vitamin*E or folic acid or iron or IFA or zinc or copper or selenium or iodine or calcium or MMN or multi*vitamin* or multiple micronutrient or magnesium).mp.	1901525	
19	exp Dietary Supplements/	5340	Food supplements
20	(fortified food or food fortified or fortified, food or dietary product* or dietary food* or specialized food or foods, specialized or dietary supplement* or supplement, dietary or supplements, dietary or food supplement* or supplement, food or supplements, food or lipid based supplement or lipid supplement or soy based supplement or soy supplement or balanced energy protein supplement).mp.	29584	
21	exp Anthelmintics/	116693	Deworming
22	(Albendazole or mebendazole or praziquantel or ivermectin or benzimidazole or praziquantel).mp.	46516	
23	exp Prenatal education/	332	Education
24	(Maternal education or mother education or education, maternal or education, mother).mp.	4664	
25	exp Sanitation/	396291	WASH
26	(waste water management or drinking water or sanitation or sewage disposal or septic tank or latrine* or toilet* or hygiene or WASH or	320752	

© 2019 Park JJH et al. *JAMA Network Open*.

No.	Terms	Hits	Comments
	chlorine tablet* or hand washing or water store or potty or soap or detergent).mp.		
27	or/17-26	2588619	**Interventions combined**
28	randomized controlled trial.tw.	73033	RCT filter
29	controlled clinical trial.tw.	15579	
30	randomized.ti.	167263	
31	randomized.ab.	571858	
32	placebo.ab.	259821	
33	placebo.ti.	44157	
34	placebo$.tw.	269336	
35	Clinical Trials as Topic/	63385	
36	randomly.ab.	371280	
37	randomly.ti.	1557	
38	trial.ti.	243423	
39	Clinical Trial/	967438	
40	Randomized Controlled Trial/	489746	
41	controlled clinical trial/	455586	
42	multicenter study/	177036	
43	Phase 3 clinical trial/	32667	
44	Phase 4 clinical trial/	2841	
45	exp RANDOMIZATION/	77284	
46	Single Blind Procedure/	30519	
47	Double Blind Procedure/	146899	
48	Crossover Procedure/	54524	
49	PLACEBO/	320337	
50	randomi?ed controlled trial$.tw.	175450	
51	rct.tw.	27432	
52	(random$ adj2 allocat$).tw.	35973	
53	single blind$.tw.	20682	
54	double blind$.tw.	186640	
55	((treble or triple) adj blind$).tw.	775	
56	or/28-55	2098711	Combined RCT terms
57	Case Study/	52371	Case report, letter, historical article, and other irrelvant publications
58	case report.tw.	357037	
59	abstract report/ or letter/	1044306	
60	Conference proceeding.pt.	0	
61	Conference abstract.pt.	2905562	
62	Editorial.pt.	556791	
63	Letter.pt.	1004205	
64	Note.pt.	703121	
65	or/57-64	5576079	Combined case report, letter, historical article, and

© 2019 Park JJH et al. *JAMA Network Open*.

No.	Terms	Hits	Comments
			other irrelvant publications
66	56 not 65	1605554	Excluding case report, letter, historical article, and other irrelvant publications
67	Humans/	13728469	
68	Animals/	1688264	
69	68 not 67	1327415	Animal studies
70	66 not 69	1565435	Final study design term
71	16 and 27 and 70	2085	Population, interventions and study design combined
72	limit 71 to english language	1789	Limit to English

© 2019 Park JJH et al. *JAMA Network Open*.

eTable 4. Search strategy for pregnancy: MEDLINE

Executed on August 14, 2018

No.	Terms	Hits	Comments
1	exp pregnancy/	827751	Population
2	exp Pregnancy Trimester/ or exp Pregnancy Trimester, First/ or exp Pregnancy Trimester, Second/ or exp Pregnancy Trimester, Third/	37442	
3	exp Pregnant women/	6536	
4	(Pregnant women or pregnant woman or pregnan* or gravid or obstetric or antenatal or antepartum or gestation).ti,ab.	524881	
5	or/1-4	957311	
6	Developing Countries.sh,kf.	80303	LMIC terms
7	(Africa or Asia or Caribbean or West Indies or South America or Latin America or Central America).hw,kf,ti,ab,cp.	233543	
8	(Afghanistan or Albania or Algeria or Angola or Antigua or Barbuda or Argentina or Armenia or Armenian or Aruba or Azerbaijan or Bahrain or Bangladesh or Barbados or Benin or Byelarus or Byelorussian or Belarus or Belorussian or Belorussia or Belize or Bhutan or Bolivia or Bosnia or Herzegovina or Hercegovina or Botswana or Brasil or Brazil or Bulgaria or Burkina Faso or Burkina Fasso or Upper Volta or Burundi or Urundi or Cambodia or Khmer Republic or Kampuchea or Cameroon or Cameroons or Cameron or Camerons or Cape Verde or Central African Republic or Chad or Chile or China or Colombia or Comoros or Comoro Islands or Comores or Mayotte or Congo or Zaire or Costa Rica or Cote d'Ivoire or Ivory Coast or Croatia or Cuba or Cyprus or Czechoslovakia or Czech Republic or Slovakia or Slovak Republic or Djibouti or French Somaliland or Dominica or Dominican Republic or East Timor or East Timur or Timor Leste or Ecuador or Egypt or United Arab Republic or El Salvador or Eritrea or Estonia or Ethiopia or Fiji or Gabon or Gabonese Republic or Gambia or Gaza or Georgia Republic or Georgian Republic or Ghana or Gold Coast or Greece or Grenada or Guatemala or Guinea or Guam or Guiana or Guyana or Haiti or Honduras or Hungary or India or Maldives or Indonesia or Iran or Iraq or Isle of Man or Jamaica or Jordan or Kazakhstan or Kazakh or Kenya or Kiribati or Korea or Kosovo or Kyrgyzstan or Kirghizia or Kyrgyz Republic or Kirghiz or Kirgizstan or Lao PDR or Laos or Latvia or Lebanon or Lesotho or Basutoland or Liberia or Libya or Lithuania or Macedonia or Madagascar or Malagasy Republic or Malaysia or Malaya or Malay or Sabah or Sarawak or Malawi or Nyasaland or Mali or Malta or Marshall Islands or Mauritania or Mauritius or Agalega Islands or Mexico or Micronesia or Middle East or Moldova or Moldovia or Moldovian or Mongolia or Montenegro or Morocco or Ifni or Mozambique or Myanmar or Myanma or Burma or Namibia or Nepal or Netherlands Antilles or New Caledonia or Nicaragua or Niger or Nigeria or Northern Mariana Islands or Oman or Muscat or Pakistan or Palau or Palestine or Panama or Paraguay or Peru or Philippines or Philipines or Phillipines or Phillippines or Poland or Portugal or Puerto Rico or Romania or Rumania or Roumania or Russia or Russian or Rwanda or Ruanda or Saint Kitts or St Kitts or Nevis or Saint Lucia or St Lucia or Saint Vincent or St Vincent or Grenadines or Samoa or Samoan Islands or Navigator Island or Navigator Islands or Sao Tome or Saudi Arabia or Senegal or Serbia or Montenegro or Seychelles or Sierra Leone or Slovenia or Sri Lanka or Ceylon or Solomon Islands or Somalia or	3245968	

© 2019 Park JJH et al. *JAMA Network Open*.

No.	Terms	Hits	Comments
	South Africa or Sudan or Suriname or Surinam or Swaziland or Syria or Tajikistan or Tadzhikistan or Tadjikistan or Tadzhik or Tanzania or Thailand or Togo or Togolese Republic or Tonga or Trinidad or Tobago or Tunisia or Turkey or Turkmenistan or Turkmen or Uganda or Ukraine or Uruguay or USSR or Soviet Union or Union of Soviet Socialist Republics or Uzbekistan or Uzbek or Vanuatu or New Hebrides or Venezuela or Vietnam or Viet Nam or West Bank or Yemen or Yugoslavia or Zambia or Zimbabwe or Rhodesia).hw,kf,ti,ab,cp.		
9	((developing or less* developed or under developed or underdeveloped or middle income or low* income or underserved or under served or deprived or poor*) adj (countr* or nation? or population? or world)).ti,ab.	79580	
10	((developing or less* developed or under developed or underdeveloped or middle income or low* income) adj (economy or economies)).ti,ab.	407	
11	(low* adj (gdp or gnp or gross domestic or gross national)).ti,ab.	212	
12	(lmic or lmics or third world or lami countr*).ti,ab.	5133	
13	transitional countr*.ti,ab.	140	
14	or/6-13	3377464	
15	5 and 14	160554	**Population and LMIC terms combined**
16	exp Micronutrients/	604217	Micronutrient and calcium supplements
17	(Vitamin* or provitamin* or mineral* or trace element* or provitamin* or vitamin*A or vitamin*B or vitamin*B1 or vitamin*B2 or vitamin*B6 or vitamin*B12 or niacin or vitamin*C or vitamin*D or vitamin*E or folic acid or iron or IFA or zinc or copper or selenium or iodine or calcium or MMN or multi*vitamin* or multiple micronutrient or magnesium).mp.	1449315	
18	exp Dietary Supplements/	60802	Food supplements
19	(fortified food or food fortified or fortified, food or dietary product* or dietary food* or specialized food or foods, specialized or dietary supplement* or supplement, dietary or supplements, dietary or food supplement* or supplement, food or supplements, food or lipid based supplement or lipid supplement or soy based supplement or soy supplement or balanced energy protein supplement).mp.	64914	
20	exp Anthelmintics/	55622	Deworming
21	(Albendazole or mebendazole or praziquantel or ivermectin or benzimidazole or praziquantel).mp.	24027	
22	exp Prenatal education/	148	Education
23	(Maternal education or mother education or education, maternal or education, mother).mp.	3800	
24	exp Sanitation/	81650	WASH
25	(waste water management or drinking water or sanitation or sewage disposal or septic tank or latrine* or toilet* or hygiene or WASH or chlorine tablet* or hand washing or water store or potty or soap or detergent).mp.	177103	
26	or/16-25	1941605	**Interventions combined**

© 2019 Park JJH et al. *JAMA Network Open*.

No.	Terms	Hits	Comments
27	Randomized Controlled Trials as Topic/	114422	RCT filter
28	Randomized controlled trial/	454848	
29	Random Allocation/	93424	
30	Double Blind Method/	144395	
31	Single Blind Method/	24709	
32	Clinical trial/	508768	
33	Controlled clinical trial.pt.	92204	
34	Randomized controlled trial.pt.	454848	
35	Multicenter study.pt.	229457	
36	Clinical trial.pt.	508768	
37	exp Clinical Trials as topic/	310995	
38	(clinical adj trial$).tw.	294100	
39	((singl$ or doubl$ or treb$ or tripl$) adj (blind$3 or mask$3)).tw.	152960	
40	PLACEBOS/	33812	
41	placebo$.tw.	190010	
42	randomly allocated.tw.	23321	
43	(allocated adj2 random$).tw.	26294	
44	or/27-43	1418960	**RCT combined**
45	case report.tw.	261506	Case report, letter, and historical article
46	letter/	972486	
47	Historical article/	343572	
48	or/45-47	1563653	
49	44 not 48	1386187	Excluding case report, letter, and historical article
50	Humans/	16917249	
51	Animals/	6163165	
52	51 not 50	4398339	Animals
53	49 not 52	1303603	**Final study terms: Excluding animals**
54	15 and 26 and 53	1698	**Population, interventions and study design combined**
55	limit 54 to english language	1531	Limit to English

© 2019 Park JJH et al. *JAMA Network Open*.

eTable 5. Search strategy for children life stages: CENTRAL

Executed on August 14, 2018

No.	Terms	Hits	Comments
1	exp Infant/	28139	Infant term
2	exp Infant, Newborn/	13684	Newborn term
3	(Infant or infan* or newborn* or new-born* or baby or babies or neonat* or perinat* or postnat* or child* or kid* or toddler* or youth* or pediatrics or pediatric* or paediatric* or peadiatric*).mp.	163817	
4	or/1-3	163817	**Population terms combined**
5	Developing Countries/	702	LMIC terms
6	(Africa or Asia or Caribbean or West Indies or South America or Latin America or Central America).hw,ti,ab,cp.	7824	
7	(Afghanistan or Albania or Algeria or Angola or Antigua or Barbuda or Argentina or Armenia or Armenian or Aruba or Azerbaijan or Bahrain or Bangladesh or Barbados or Benin or Byelarus or Byelorussian or Belarus or Belorussian or Belorussia or Belize or Bhutan or Bolivia or Bosnia or Herzegovina or Hercegovina or Botswana or Brasil or Brazil or Bulgaria or Burkina Faso or Burkina Fasso or Upper Volta or Burundi or Urundi or Cambodia or Khmer Republic or Kampuchea or Cameroon or Cameroons or Cameron or Camerons or Cape Verde or Central African Republic or Chad or Chile or China or Colombia or Comoros or Comoro Islands or Comores or Mayotte or Congo or Zaire or Costa Rica or Cote d'Ivoire or Ivory Coast or Croatia or Cuba or Cyprus or Czechoslovakia or Czech Republic or Slovakia or Slovak Republic or Djibouti or French Somaliland or Dominica or Dominican Republic or East Timor or East Timur or Timor Leste or Ecuador or Egypt or United Arab Republic or El Salvador or Eritrea or Estonia or Ethiopia or Fiji or Gabon or Gabonese Republic or Gambia or Gaza or Georgia Republic or Georgian Republic or Ghana or Gold Coast or Greece or Grenada or Guatemala or Guinea or Guam or Guiana or Guyana or Haiti or Honduras or Hungary or India or Maldives or Indonesia or Iran or Iraq or Isle of Man or Jamaica or Jordan or Kazakhstan or Kazakh or Kenya or Kiribati or Korea or Kosovo or Kyrgyzstan or Kirghizia or Kyrgyz Republic or Kirghiz or Kirgizstan or Lao PDR or Laos or Latvia or Lebanon or Lesotho or Basutoland or Liberia or Libya or Lithuania or Macedonia or Madagascar or Malagasy Republic or Malaysia or Malaya or Malay or Sabah or Sarawak or Malawi or Nyasaland or Mali or Malta or Marshall Islands or Mauritania or Mauritius or Agalega Islands or Mexico or Micronesia or Middle East or Moldova or Moldovia or Moldovian or Mongolia or Montenegro or Morocco or Ifni or Mozambique or Myanmar or Myanma or Burma or Namibia or Nepal or Netherlands Antilles or New Caledonia or Nicaragua or Niger or Nigeria or Northern Mariana Islands or Oman or Muscat or Pakistan or Palau or Palestine or Panama or Paraguay or Peru or Philippines or Philipines or Phillipines or Phillippines or Poland or Portugal or Puerto Rico or Romania or Rumania or Roumania or Russia or Russian or Rwanda or Ruanda or Saint Kitts or St Kitts or Nevis or Saint Lucia or St Lucia or Saint Vincent or St Vincent or Grenadines or Samoa or Samoan Islands or Navigator Island or Navigator Islands or Sao Tome or Saudi Arabia or Senegal or Serbia or Montenegro or Seychelles or Sierra Leone or Slovenia or Sri Lanka or Ceylon or Solomon Islands or Somalia or South Africa or Sudan or Suriname or Surinam or Swaziland or Syria or Tajikistan or Tadzhikistan or	76644	

© 2019 Park JJH et al. *JAMA Network Open*.

No.	Terms	Hits	Comments
	Tadjikistan or Tadzhik or Tanzania or Thailand or Togo or Togolese Republic or Tonga or Trinidad or Tobago or Tunisia or Turkey or Turkmenistan or Turkmen or Uganda or Ukraine or Uruguay or USSR or Soviet Union or Union of Soviet Socialist Republics or Uzbekistan or Uzbek or Vanuatu or New Hebrides or Venezuela or Vietnam or Viet Nam or West Bank or Yemen or Yugoslavia or Zambia or Zimbabwe or Rhodesia).hw,ti,ab,cp.		
8	((developing or less* developed or under developed or underdeveloped or middle income or low* income or underserved or under served or deprived or poor*) adj (countr* or nation? or population? or world)).ti,ab.	4060	
9	((developing or less* developed or under developed or underdeveloped or middle income or low* income) adj (economy or economies)).ti,ab.	15	
10	(low* adj (gdp or gnp or gross domestic or gross national)).ti,ab.	39	
11	(low adj3 middle adj3 countr*).ti,ab.	528	
12	(lmic or lmics or third world or lami countr*).ti,ab.	167	
13	transitional countr*.ti,ab.	3	
14	or/5-13	81242	
15	4 and 14	21879	**Population and LMIC terms combined**
16	exp Micronutrients/	18026	Micronutrient supplements and calcium
17	(Vitamin* or provitamin* or mineral* or trace element* or provitamin* or vitamin*A or vitamin*B or vitamin*B1 or vitamin*B2 or vitamin*B6 or vitamin*B12 or niacin or vitamin*C or vitamin*D or vitamin*E or folic acid or iron or IFA or zinc or copper or selenium or iodine or calcium or MMN or multi*vitamin* or multiple micronutrient or magnesium).mp.	61133	
18	exp Dietary Supplements/	9748	Food supplements
19	(fortified food or food fortified or fortified, food or dietary product* or dietary food* or specialized food or foods, specialized or dietary supplement* or supplement, dietary or supplements, dietary or food supplement* or supplement, food or supplements, food or lipid based supplement or lipid supplement or soy based supplement or soy supplement or fatty acid supplement or omega fatty acid supplement or DHA or EPA or fish oil or long chain fatty acid).mp.	13945	
20	exp Kangaroo-Mother Care Method	43	Kangaroo care
21	(Kangaroo care or skin-to-skin contact or skin to skin contact or kangarooing).mp.	274	
22	exp Anthelmintics/	1387	Deworming
23	(Albendazole or mebendazole or praziquantel or ivermectin or benzimidazole or praziquantel).mp.	1388	
24	exp Breast feeding/ and (exp Health Promotion/ or Health Education/)	182	Maternal and breastfeeding education
25	(Maternal education or mother education or education, maternal or education, mother or breast feeding promotion or breastfeeding promotion or breast feeding education or breastfeeding education).mp.	449	
26	exp Sanitation/	346	WASH
27	(waste water management or drinking water or sanitation or sewage disposal or septic tank or latrine* or toilet* or hygiene or WASH or	9931	

© 2019 Park JJH et al. *JAMA Network Open*.

No.	Terms	Hits	Comments
	chlorine tablet* or hand washing or water store or potty or soap or detergent).mp.		
28	or/16-27	86993	**Interventions combined**
29	15 and 28	5117	**Population and interventions combined**
30	Limit 29 to English	4663	Limit to English

© 2019 Park JJH et al. *JAMA Network Open*.

eTable 6. Search strategy for children life stages: Embase

Executed on August 14, 2018

No.	Terms	Hits	Comments
1	exp Infant/	984893	Infant term
2	exp Infant, Newborn/	536428	Newborn term
3	(Infant or infan* or newborn* or new-born* or baby or babies or neonat* or perinat* or postnat* or child* or kid* or toddler* or youth* or pediatrics or pediatric* or paediatric* or peadiatric*).mp.	4519804	
4	or/1-3	4519812	**Population terms combined**
5	Developing Country.sh.	88669	LMIC terms
6	(Africa or Asia or Caribbean or West Indies or South America or Latin America or Central America).hw,ti,ab,cp.	301639	
7	(Afghanistan or Albania or Algeria or Angola or Antigua or Barbuda or Argentina or Armenia or Armenian or Aruba or Azerbaijan or Bahrain or Bangladesh or Barbados or Benin or Byelarus or Byelorussian or Belarus or Belorussian or Belorussia or Belize or Bhutan or Bolivia or Bosnia or Herzegovina or Hercegovina or Botswana or Brasil or Brazil or Bulgaria or Burkina Faso or Burkina Fasso or Upper Volta or Burundi or Urundi or Cambodia or Khmer Republic or Kampuchea or Cameroon or Cameroons or Cameron or Camerons or Cape Verde or Central African Republic or Chad or Chile or China or Colombia or Comoros or Comoro Islands or Comores or Mayotte or Congo or Zaire or Costa Rica or Cote d'Ivoire or Ivory Coast or Croatia or Cuba or Cyprus or Czechoslovakia or Czech Republic or Slovakia or Slovak Republic or Djibouti or French Somaliland or Dominica or Dominican Republic or East Timor or East Timur or Timor Leste or Ecuador or Egypt or United Arab Republic or El Salvador or Eritrea or Estonia or Ethiopia or Fiji or Gabon or Gabonese Republic or Gambia or Gaza or Georgia Republic or Georgian Republic or Ghana or Gold Coast or Greece or Grenada or Guatemala or Guinea or Guam or Guiana or Guyana or Haiti or Honduras or Hungary or India or Maldives or Indonesia or Iran or Iraq or Isle of Man or Jamaica or Jordan or Kazakhstan or Kazakh or Kenya or Kiribati or Korea or Kosovo or Kyrgyzstan or Kirghizia or Kyrgyz Republic or Kirghiz or Kirgizstan or Lao PDR or Laos or Latvia or Lebanon or Lesotho or Basutoland or Liberia or Libya or Lithuania or Macedonia or Madagascar or Malagasy Republic or Malaysia or Malaya or Malay or Sabah or Sarawak or Malawi or Nyasaland or Mali or Malta or Marshall Islands or Mauritania or Mauritius or Agalega Islands or Mexico or Micronesia or Middle East or Moldova or Moldovia or Moldovian or Mongolia or Montenegro or Morocco or Ifni or Mozambique or Myanmar or Myanma or Burma or Namibia or Nepal or Netherlands Antilles or New Caledonia or Nicaragua or Niger or Nigeria or Northern Mariana Islands or Oman or Muscat or Pakistan or Palau or Palestine or Panama or Paraguay or Peru or Philippines or Philipines or Phillipines or Phillippines or Poland or Portugal or Puerto Rico or Romania or Rumania or Roumania or Russia or Russian or Rwanda or Ruanda or Saint Kitts or St Kitts or Nevis or Saint Lucia or St Lucia or Saint Vincent or St Vincent or Grenadines or Samoa or Samoan Islands or Navigator Island or Navigator Islands or Sao Tome or Saudi Arabia or Senegal or Serbia or Montenegro or Seychelles or Sierra Leone or Slovenia or Sri Lanka or Ceylon or Solomon Islands or Somalia or	3765601	

© 2019 Park JJH et al. *JAMA Network Open*.

No.	Terms	Hits	Comments
	South Africa or Sudan or Suriname or Surinam or Swaziland or Syria or Tajikistan or Tadzhikistan or Tadjikistan or Tadzhik or Tanzania or Thailand or Togo or Togolese Republic or Tonga or Trinidad or Tobago or Tunisia or Turkey or Turkmenistan or Turkmen or Uganda or Ukraine or Uruguay or USSR or Soviet Union or Union of Soviet Socialist Republics or Uzbekistan or Uzbek or Vanuatu or New Hebrides or Venezuela or Vietnam or Viet Nam or West Bank or Yemen or Yugoslavia or Zambia or Zimbabwe or Rhodesia).hw,ti,ab,cp.		
8	((developing or less* developed or under developed or underdeveloped or middle income or low* income or underserved or under served or deprived or poor*) adj (countr* or nation? or population? or world)).ti,ab.	101323	
9	((developing or less* developed or under developed or underdeveloped or middle income or low* income) adj (economy or economies)).ti,ab.	541	
10	(low* adj (gdp or gnp or gross domestic or gross national)).ti,ab.	309	
11	(low adj3 middle adj3 countr*).ti,ab.	11270	
12	(lmic or lmics or third world or lami countr*).ti,ab.	6366	
13	transitional countr*.ti,ab.	199	
14	or/5-13	3958832	
15	4 and 14	728626	**Population and LMIC terms combined**
16	exp Micronutrients/	35349	Micronutrient supplements and calcium
17	(Vitamin* or provitamin* or mineral* or trace element* or provitamin* or vitamin*A or vitamin*B or vitamin*B1 or vitamin*B2 or vitamin*B6 or vitamin*B12 or niacin or vitamin*C or vitamin*D or vitamin*E or folic acid or iron or IFA or zinc or copper or selenium or iodine or calcium or MMN or multi*vitamin* or multiple micronutrient or magnesium).mp.	1901525	
18	exp Dietary Supplements/	5340	Food supplements
19	(fortified food or food fortified or fortified, food or dietary product* or dietary food* or specialized food or foods, specialized or dietary supplement* or supplement, dietary or supplements, dietary or food supplement* or supplement, food or supplements, food or lipid based supplement or lipid supplement or soy based supplement or soy supplement or fatty acid supplement or omega fatty acid supplement or DHA or EPA or fish oil or long chain fatty acid).mp.	81370	
20	exp Kangaroo-Mother Care Method	768	Kangaroo care
21	(Kangaroo care or skin-to-skin contact or skin to skin contact or kangarooing).mp.	1512	
22	exp Anthelmintics/	116693	Deworming
23	(Albendazole or mebendazole or praziquantel or ivermectin or benzimidazole or praziquantel).mp.	46516	
24	exp Breast feeding/ and (exp Health Promotion/ or Health Education/)	3251	Maternal and breastfeeding education
25	(Maternal education or mother education or education, maternal or education, mother or breast feeding promotion or breastfeeding	7329	

© 2019 Park JJH et al. *JAMA Network Open*.

No.	Terms	Hits	Comments
	promotion or breast feeding education or breastfeeding education).mp.		
26	exp Sanitation/	396291	WASH
27	(waste water management or drinking water or sanitation or sewage disposal or septic tank or latrine* or toilet* or hygiene or WASH or chlorine tablet* or hand washing or water store or potty or soap or detergent).mp.	320752	
28	or/16-27	2635168	**Interventions combined**
29	randomized controlled trial.tw.	73033	RCT filter
30	controlled clinical trial.tw.	15579	
31	randomized.ti.	167263	
32	randomized.ab.	571858	
33	placebo.ab.	259821	
34	placebo.ti.	44157	
35	placebo$.tw.	269336	
36	Clinical Trials as Topic/	63385	
37	randomly.ab.	371280	
38	randomly.ti.	1557	
39	trial.ti.	243423	
40	Clinical Trial/	967438	
41	Randomized Controlled Trial/	489746	
42	controlled clinical trial/	455586	
43	multicenter study/	177036	
44	Phase 3 clinical trial/	32667	
45	Phase 4 clinical trial/	2841	
46	exp RANDOMIZATION/	77284	
47	Single Blind Procedure/	30519	
48	Double Blind Procedure/	146899	
49	Crossover Procedure/	54524	
50	PLACEBO/	320337	
51	randomi?ed controlled trial$.tw.	175450	
52	rct.tw.	27432	
53	(random$ adj2 allocat$).tw.	35973	
54	single blind$.tw.	20682	
55	double blind$.tw.	186640	
56	((treble or triple) adj blind$).tw.	775	
57	or/29-56	2098711	Combined RCT terms
58	Case Study/	52371	Case report, letter, historical article, and other irrelvant publications
59	case report.tw.	357037	
60	abstract report/ or letter/	1044306	
61	Conference proceeding.pt.	0	
62	Conference abstract.pt.	2905562	
63	Editorial.pt.	556791	
64	Letter.pt.	1004205	
65	Note.pt.	703121	
66	or/58-65	5576079	Combined

© 2019 Park JJH et al. *JAMA Network Open*.

No.	Terms	Hits	Comments
			case report, letter, historical article, and other irrelvant publications
67	57 not 66	1605554	Excluding case report, letter, historical article, and other irrelvant publications
68	Humans/	13728469	
69	Animals/	1688264	
70	69 not 68	1327415	Animal studies
71	67 not 70	1565435	**Final study design term: studying animal studies**
72	15 and 28 and 71	8978	**Population, interventions and study design combined**
73	limit 72 to english language	7748	Limit to English

© 2019 Park JJH et al. *JAMA Network Open*.

eTable 7. Search strategy for children life stages: MEDLINE

Last conducted August 14 2018

No.	Terms	Hits	Comments
1	exp Infant/	1056827	Infant term
2	exp Infant, Newborn/	562096	Newborn term
3	(Infant or infan* or newborn* or new-born* or baby or babies or neonat* or perinat* or postnat* or child* or kid* or toddler* or youth* or pediatrics or pediatric* or paediatric* or peadiatric*).mp.	3749589	
4	or/1-3	3749589	**Population terms combined**
5	Developing Countries.sh,kf.	80388	LMIC terms
6	(Africa or Asia or Caribbean or West Indies or South America or Latin America or Central America).hw,kf,ti,ab,cp.	237160	
7	(Afghanistan or Albania or Algeria or Angola or Antigua or Barbuda or Argentina or Armenia or Armenian or Aruba or Azerbaijan or Bahrain or Bangladesh or Barbados or Benin or Byelarus or Byelorussian or Belarus or Belorussian or Belorussia or Belize or Bhutan or Bolivia or Bosnia or Herzegovina or Hercegovina or Botswana or Brasil or Brazil or Bulgaria or Burkina Faso or Burkina Fasso or Upper Volta or Burundi or Urundi or Cambodia or Khmer Republic or Kampuchea or Cameroon or Cameroons or Cameron or Camerons or Cape Verde or Central African Republic or Chad or Chile or China or Colombia or Comoros or Comoro Islands or Comores or Mayotte or Congo or Zaire or Costa Rica or Cote d'Ivoire or Ivory Coast or Croatia or Cuba or Cyprus or Czechoslovakia or Czech Republic or Slovakia or Slovak Republic or Djibouti or French Somaliland or Dominica or Dominican Republic or East Timor or East Timur or Timor Leste or Ecuador or Egypt or United Arab Republic or El Salvador or Eritrea or Estonia or Ethiopia or Fiji or Gabon or Gabonese Republic or Gambia or Gaza or Georgia Republic or Georgian Republic or Ghana or Gold Coast or Greece or Grenada or Guatemala or Guinea or Guam or Guiana or Guyana or Haiti or Honduras or Hungary or India or Maldives or Indonesia or Iran or Iraq or Isle of Man or Jamaica or Jordan or Kazakhstan or Kazakh or Kenya or Kiribati or Korea or Kosovo or Kyrgyzstan or Kirghizia or Kyrgyz Republic or Kirghiz or Kirgizstan or Lao PDR or Laos or Latvia or Lebanon or Lesotho or Basutoland or Liberia or Libya or Lithuania or Macedonia or Madagascar or Malagasy Republic or Malaysia or Malaya or Malay or Sabah or Sarawak or Malawi or Nyasaland or Mali or Malta or Marshall Islands or Mauritania or Mauritius or Agalega Islands or Mexico or Micronesia or Middle East or Moldova or Moldovia or Moldovian or Mongolia or Montenegro or Morocco or Ifni or Mozambique or Myanmar or Myanma or Burma or Namibia or Nepal or Netherlands Antilles or New Caledonia or Nicaragua or Niger or Nigeria or Northern Mariana Islands or Oman or Muscat or Pakistan or Palau or Palestine or Panama or Paraguay or Peru or Philippines or Philipines or Phillipines or Phillippines or Poland or Portugal or Puerto Rico or Romania or Rumania or Roumania or Russia or Russian or Rwanda or Ruanda or Saint Kitts or St Kitts or Nevis or Saint Lucia or St Lucia or Saint Vincent or St Vincent or Grenadines or Samoa or Samoan Islands or Navigator Island or Navigator Islands or Sao Tome or Saudi Arabia or Senegal or Serbia or Montenegro or Seychelles or Sierra Leone or Slovenia or Sri Lanka or Ceylon or Solomon Islands or Somalia or	3282292	

© 2019 Park JJH et al. *JAMA Network Open*.

No.	Terms	Hits	Comments
	South Africa or Sudan or Suriname or Surinam or Swaziland or Syria or Tajikistan or Tadzhikistan or Tadjikistan or Tadzhik or Tanzania or Thailand or Togo or Togolese Republic or Tonga or Trinidad or Tobago or Tunisia or Turkey or Turkmenistan or Turkmen or Uganda or Ukraine or Uruguay or USSR or Soviet Union or Union of Soviet Socialist Republics or Uzbekistan or Uzbek or Vanuatu or New Hebrides or Venezuela or Vietnam or Viet Nam or West Bank or Yemen or Yugoslavia or Zambia or Zimbabwe or Rhodesia).hw,kf,ti,ab,cp.		
8	((developing or less* developed or under developed or underdeveloped or middle income or low* income or underserved or under served or deprived or poor*) adj (countr* or nation? or population? or world)).ti,ab.	81508	
9	((developing or less* developed or under developed or underdeveloped or middle income or low* income) adj (economy or economies)).ti,ab.	422	
10	(low* adj (gdp or gnp or gross domestic or gross national)).ti,ab.	213	
11	(lmic or lmics or third world or lami countr*).ti,ab.	5320	
12	transitional countr*.ti,ab.	142	
13	or/5-12	3416285	**LMIC terms combined**
14	4 and 13	631731	**Population and LMIC terms combined**
15	exp Micronutrients/	604217	Micronutrient supplements and calcium
16	(Vitamin* or provitamin* or mineral* or trace element* or provitamin* or vitamin*A or vitamin*B or vitamin*B1 or vitamin*B2 or vitamin*B6 or vitamin*B12 or niacin or vitamin*C or vitamin*D or vitamin*E or folic acid or iron or IFA or zinc or copper or selenium or iodine or calcium or MMN or multi*vitamin* or multiple micronutrient or magnesium).mp.	1465505	
17	exp Dietary Supplements/	60802	Food supplements
18	(fortified food or food fortified or fortified, food or dietary product* or dietary food* or specialized food or foods, specialized or dietary supplement* or supplement, dietary or supplements, dietary or food supplement* or supplement, food or supplements, food or lipid based supplement or lipid supplement or soy based supplement or soy supplement or fatty acid supplement or omega fatty acid supplement or DHA or EPA or fish oil or long chain fatty acid).mp.	92414	
19	exp Kangaroo-Mother Care Method	242	Kangaroo care
20	(Kangaroo care or skin-to-skin contact or skin to skin contact or kangarooing).mp.	833	
21	exp Anthelmintics/	55622	Deworming
22	(Albendazole or mebendazole or praziquantel or ivermectin or benzimidazole or praziquantel).mp.	24331	
23	exp Breast feeding/ and (exp Health Promotion/ or Health Education/)	2875	Maternal and breastfeeding education
24	(Maternal education or mother education or education, maternal or education, mother or breast feeding promotion or breastfeeding	4877	

© 2019 Park JJH et al. *JAMA Network Open*.

No.	Terms	Hits	Comments
	promotion or breast feeding education or breastfeeding education).mp.		
25	exp Sanitation/	81650	WASH
26	(waste water management or drinking water or sanitation or sewage disposal or septic tank or latrine* or toilet* or hygiene or WASH or chlorine tablet* or hand washing or water store or potty or soap or detergent).mp.	179104	
27	or/15-26	1986960	**Interventions combined**
28	Randomized Controlled Trials as Topic/	114422	RCT filter
29	Randomized controlled trial/	454849	
30	Random Allocation/	93424	
31	Double Blind Method/	144395	
32	Single Blind Method/	24709	
33	Clinical trial/	508777	
34	Controlled clinical trial.pt.	92204	
35	Randomized controlled trial.pt.	454849	
36	Multicenter study.pt.	229457	
37	Clinical trial.pt.	508777	
38	exp Clinical Trials as topic/	310995	
39	(clinical adj trial$).tw.	301382	
40	((singl$ or doubl$ or treb$ or tripl$) adj (blind$3 or mask$3)).tw.	154985	
41	PLACEBOS/	33812	
42	placebo$.tw.	192948	
43	randomly allocated.tw.	23748	
44	(allocated adj2 random$).tw.	26761	
45	or/28-44	1429526	**RCT terms combined**
46	case report.tw.	266711	Case report, letter, and historical article
47	letter/	978349	
48	Historical article/	343572	
49	or/46-48	1574610	
50	45 not 49	1396681	RCT without case report, letter, and historical article
51	Humans/	16917249	
52	Animals/	6163165	
53	52 not 51	4398339	Excluding animals
54	50 not 53	1314097	**Study design terms combined**
55	14 and 27 and 54	6055	**Population, interventions and study design combined**
56	limit 55 to english language	5437	Limit to

© 2019 Park JJH et al. *JAMA Network Open*.

No.	Terms	Hits	Comments
			English

© 2019 Park JJH et al. *JAMA Network Open*.

© 2019 Park JJH et al. *JAMA Network Open*.

List of included studies for each life stage

eTable 8. List of included studies for pregnancy life stage

Trial ID	Trial Registry	Study ID	Title
Adu-Afarwuah 2015	NCT00970866	Adu-Afarwuah 2015 [70]	Lipid-based nutrient supplement increases the birth size of infants of primiparous women in Ghana
Adu-Afarwuah 2015	NCT00970866	Adu-Afarwuah 2016 [71]	Small-quantity, lipid-based nutrient supplements provided to women during pregnancy and 6 mo postpartum and to their infants from 6 mo of age increase the mean attained length of 18-mo-old children in semi-urban Ghana: A randomized controlled trial
Adu-Afarwuah 2015	NCT00970866	Prado 2016 [72]	Effects of pre- and post-natal lipid-based nutrient supplements on infant development in a randomized trial in Ghana
Ahmad 2016	NR	Ahmad 2016 [73]	Maternal zinc supplementation improves hepatitis b antibody responses in infants but decreases plasma zinc level
Alizadeh 2016	IRCT2013020612383N1	Alizadeh 2016 [74]	Is routine iron supplementation necessary in pregnant women with high hemoglobin?
Asci 2016	NR	Asci 2016 [75]	Effect of lifestyle interventions of pregnant women on their dietary habits, lifestyle behaviors, and weight gain: A randomized controlled trial
Asemi 2012	NR	Asemi 2012 [76]	Effect of calcium-vitamin D supplementation on metabolic profiles in pregnant women at risk for pre-eclampsia: a randomized placebo-controlled trial
Asemi 2013	NR	Asemi 2013 [77]	Vitamin D supplementation affects serum high-sensitivity C-reactive protein, insulin resistance, and biomarkers of oxidative stress in pregnant women.
Asemi 2015	NR	Asemi 2015 [78]	The effect of multi mineral-vitamin d supplementation on pregnancy outcomes in pregnant women at risk for pre-eclampsia
Asemi 2016	NR	Asemi 2016 [79]	Calcium-vitamin d co-supplementation affects metabolic profiles, but not pregnancy outcomes, in healthy pregnant women
Ashorn 2015A	NCT01239693	Ashorn 2015A [80]	Supplementation of maternal diets during pregnancy and for 6 months postpartum and infant diets thereafter with small-quantity lipid-based nutrient supplements does not promote child growth by 18 months of age in rural Malawi: A randomized controlled trial
Ashorn 2015A	NCT01239693	Ashorn 2015B [81]	The impact of lipid-based nutrient supplement provision to pregnant women on newborn size in rural Malawi: A randomized controlled trial
Ashorn 2015A	NCT01239693	Stewart 2015 [82]	Maternal cortisol and stress are associated with birth outcomes, but are not affected by lipid-based nutrient supplements during pregnancy: An analysis of data from a randomized controlled trial in rural Malawi
AViDD	NR	Roth 2010 [83]	Randomized placebo-controlled trial of high-dose prenatal third-trimester vitamin D3

© 2019 Park JJH et al. *JAMA Network Open*.

Trial ID	Trial Registry	Study ID	Title
			supplementation in Bangladesh: the AViDD trial.
Belizan 1991	NR	Belizan 1991 [84]	Calcium supplementation to prevent hypertensive disorders of pregnancy
Bhutta 2009a	NR	Bhutta 2009a [85]	A comparative evaluation of multiple micronutrient and iron-folate supplementation during pregnancy in Pakistan: impact on pregnancy outcomes
Callaghan-Gillespie 2017	NCT02120599	Callaghan-Gillespie 2017 [86]	Trial of ready-to-use supplemental food and corn-soy blend in pregnant Malawian women with moderate malnutrition: A randomized controlled clinical trial
CARING trial	ISCRTN51505201	Nair 2017 [87]	Effect of participatory women's groups and counselling through home visits on children's linear growth in rural eastern India (caring trial): A cluster-randomised controlled trial
Castillo-Duran 2001	NR	Castillo-Duran 2001 [88]	Controlled trial of zinc supplementation in Chilean pregnancy adolescents
Caulfield 1999	NR	Caulfield 1999 [89]	Adding zinc to prenatal iron and folate supplements improves maternal and neonatal zinc status in a Peruvian population
Ceesay 1997	NR	Ceesay 1997 [90]	Effects on birth weight and perinatal mortality of maternal dietary supplements in rural Gambia: 5 year randomised controlled trial
Christian 2003	NR	Christian 2003 [91]	Effects of alternative maternal micronutrient supplements on low birth weight in rural Nepal: double blind randomised community trial.
Christian 2003	NR	Kozuki 2015 [92]	Risk factors and neonatal/infant mortality risk of small-for-gestational-age and preterm birth in rural nepal
Cox 2005	NR	Cox 2005 [93]	Maternal vitamin A supplementation and immunity to malaria in pregnancy in Ghanaian primigravids
Dandamrongrak 2016	NR	Dandamrongrak 2016 [94]	Correlation between iodine supplement in pregnancy and neonatal tsh level
Danesh 2010	NR	Danesh 2010 [95]	Effects of zinc supplementation during pregnancy on pregnancy outcome in women with history of preterm delivery: a double-blind randomized, placebo-controlled trial
Devi 2017	CTRI/2016/01/006578	Devi 2017 [96]	Combined vitamin b-12 and balanced protein-energy supplementation affect homocysteine remethylation in the methionine cycle in pregnant south Indian women of low vitamin b-12 status
Dijkhuizen 2004 BC	NR	Dijkhuizen 2004 BC [97]	Zinc plus beta-carotene supplementation of pregnant women is superior to beta-carotene supplementation alone in improving vitamin A status in both mothers and infants
Diogenes 2013	NCT01732328	Diogenes 2013 [98]	Effect of calcium plus vitamin D supplementation during pregnancy in Brazilian adolescent mothers: a randomized, placebo-controlled trial.
Diogenes 2013	NCT01732328	Diogenes 2015 [99]	Calcium plus vitamin d supplementation during the third trimester of pregnancy in adolescents accustomed to low calcium diets does not affect infant bone mass at early lactation in a

© 2019 Park JJH et al. *JAMA Network Open*.

Trial ID	Trial Registry	Study ID	Title
			randomized controlled trial
ENID trial	ISRCTN49285450	Johnson 2017 [100]	Association of prenatal lipid-based nutritional supplementation with fetal growth in rural Gambia
ENID trial	ISRCTN49285450	Jobarteh 2017 [101]	Mrna levels of placental iron and zinc transporter genes are upregulated in Gambian women with low iron and zinc status
Etheredge 2015	NCT01119612	Etheredge 2015 [102]	Iron supplementation in iron-replete and nonanemic pregnant women in Tanzania: A randomized clinical trial
Fawzi 2007	NCT00197548	Fawzi 2007 [103]	Vitamins and perinatal outcomes among HIV-negative women in Tanzania
Fleming 1968	NR	Fleming 1968 [104]	The prevention of megaloblastic anaemia in pregnancy in Nigeria.
Friss 2004	NR	Friss 2004 [105]	Effect of micronutrient supplementation on gestational length and birth size: a randomized, placebo- controlled, double-blind effectiveness trial in Zimbabwe
Goldberg 2013	ISRCTN96502494	Goldberg 2013 [106]	Randomized, placebo-controlled, calcium supplementation trial in pregnant Gambian women accustomed to a low calcium intake: effects on maternal blood pressure and infant growth
Gonzalez-Casanova 2015	NCT00646360	Gonzalez-Casanova 2015 [107]	Prenatal supplementation with docosahexaenoic acid has no effect on growth through 60 months of age
Gonzalez-Casanova 2015	NCT00646360	Gutierrez-Gomez 2017 [108]	Prenatal docosahexaenoic acid supplementation does not affect nonfasting serum lipid and glucose concentrations of offspring at 4 years of age in a follow-up of a randomized controlled clinical trial in Mexico
Gonzalez-Casanova 2015	NCT00646360	Ramakrishnan 2016B [109]	Prenatal supplementation with dha improves attention at 5 y of age: A randomized controlled trial
Gowachirapant 2017	NCT00791466	Gowachirapant 2017 [110]	Effect of iodine supplementation in pregnant women on child neurodevelopment: A randomised, double-blind, placebo-controlled trial
Hafeez 2005	NR	Hafeez 2005 [111]	Oral zinc supplementation in pregnant women and its effect on birth weight: a randomised controlled trial
Hanieh 2017	Australia New Zealand Clinical Trials Registry: 12610000944033.	Hanieh 2017 [112]	Effect of low-dose versus higher-dose antenatal iron supplementation on child health outcomes at 36 months of age in viet nam: Longitudinal follow-up of a cluster randomised controlled trial
Hashemipour 2014	NR	Hashemipour 2014 [113]	Effect of treatment of vitamin D deficiency and insufficiency during pregnancy on fetal growth indices and maternal weight gain: a randomized clinical trial
Hossain 2014	NCT01418664	Hossain 2014 [114]	Obstetric and neonatal outcomes of maternal vitamin D supplementation: results of an open label randomized controlled trial of antenatal vitamin D supplementation in Pakistani women

© 2019 Park JJH et al. *JAMA Network Open*.

Trial ID	Trial Registry	Study ID	Title
Huybregts 2009	NCT00909974	Huybregts 2009 [115]	Prenatal food supplementation fortified with multiple micronutrients increases birth length: a randomized controlled trial in rural Burkina Faso
Huybregts 2009	NCT00909974	Toe 2015 [116]	Seasonality modifies the effect of a lipid-based nutrient supplement for pregnant rural women on birth length
Iyengar 1975	NR	Iyengar 1975 [117]	Effect of folic acid supplementation on birth weight of infants
Janmohamed 2016	NCT01413776	Janmohamed 2016 [118]	Prenatal supplementation with corn soya blend plus reduces the risk of maternal anemia in late gestation and lowers the rate of preterm birth but does not significantly improve maternal weight gain and birth anthropometric measurements in rural cambodian women: A randomized trial
JiVitA-3	NCT000860470	West 2014 [119]	Effect of maternal multiple micronutrient vs iron-folic acid supplementation on infant mortality and adverse birth outcomes in rural Bangladesh: the JiVitA-3 randomized trial
JiVitA-3	NCT000860470	Christian 2016 [120]	Effects of prenatal multiple micronutrient supplementation on growth and cognition through 2 y of age in rural Bangladesh: The jivita-3 trial
Kaestel 2005	NR	Kaestel 2005 [121]	Effects of prenatal multimicronutrient supplements on birth weight and perinatal mortality: a randomised, controlled trial in Guinea-Bissau
Kardjati 1988	NR	Kardjati 1988 [122]	Energy supplementation in the last trimester of pregnancy in East Java: I. Effect on birthweight
Katz 2000	NR	Katz 2000 [123]	Maternal low-dose vitamin A or beta-carotene supplementation has no effect on fetal loss and early infant mortality: a randomized cluster trial in Nepal
Kumar 2009	NR	Kumar 2009 [124]	Calcium supplementation for the prevention of pre-eclampsia
Kumwenda 2002	NR	Kumwenda 2002 [125]	Antenatal vitamin A supplementation increases birth weight and decreases anemia among infants born to human immunodeficiency virus-infected women in Malawi
Larocque 2006	NR	Larocque 2006 [126]	A double-blind randomized controlled trial of antenatal mebendazole to reduce low birthweight in a hookworm-endemic area of Peru
Lopez-Jaramillo 1989	NR	Lopez-Jaramillo 1989 [127]	Calcium supplementation reduces the risk of pregnancy- induced hypertension in an Andes population
Lopez-Jaramillo 1989	NR	Lopez-Jaramillo 1990 [128]	Dietary calcium supplementation and prevention of pregnancy hypertension
Lopez-Jaramillo 1997	NR	Lopez-Jaramillo 1997 [129]	Calcium supplementation and the risk of preeclampsia in Ecuadorian pregnant teenagers
Lui 2013	NCT00133744	Lui 2013 [130]	Micronutrient supplementation and pregnancy outcomes: Double-blind randomized controlled trial in China
Lui 2013	NCT00133744	Wang 2016 [131]	Modifying effects of maternal hb concentration

© 2019 Park JJH et al. *JAMA Network Open*.

Trial ID	Trial Registry	Study ID	Title
			on infant birth weight in women receiving prenatal iron-containing supplements: A randomised controlled trial
Mardones 2007	NR	Mardones 2007 [132]	Effects of a dairy product fortified with multiple micronutrients and omega-3 fatty acids on birth weight and gestation duration in pregnant Chilean women
Marya 1988	NR	Marya 1988 [133]	Effect of vitamin D supplementation during pregnancy on foetal growth
Menedez 1994	NR	Menedez 1994 [134]	The effects or iron supplementation during pregnancy, given by traditional birth attendants, on the prevalence of anaemia and malaria
Merialdi 2004	NR	Merialdi 2004 [135]	Randomized controlled trial of prenatal zinc supplementation and the development of fetal heart rate
MINIMat	ISRCTN16581394	Persson 2012 [136]	Effects of Prenatal Micronutrient and Early Food Supplementation on Maternal Hemoglobin, Birth Weight, and Infant Mortality Among Children in Bangladesh. The MINIMat Randomized Trial
MINIMat	ISRCTN16581394	Frith 2015 [137]	Early prenatal food supplementation ameliorates the negative association of maternal stress with birth size in a randomised trial
MINIMat	ISRCTN16581394	Khan 2015 [138]	Early invitation to food and/or multiple micronutrient supplementation in pregnancy does not affect body composition in offspring at 54 months: Follow-up of the minimat randomised trial, Bangladesh
MINIMat	ISRCTN16581394	Kallioinen 2017 [139]	Prenatal early food and multiple micronutrient supplementation trial reduced infant mortality in Bangladesh, but did not influence morbidity
MINIMat	ISRCTN16581394	Khan 2017 [140]	Effect of a randomised exclusive breastfeeding counselling intervention nested into the minimat prenatal nutrition trial in Bangladesh
MINIMat	ISRCTN16581394	Ekstrom 2016 [141]	Effects of prenatal micronutrient and early food supplementation on metabolic status of the offspring at 4.5 years of age. The minimat randomized trial in rural Bangladesh
Mohammad-Alizadeh-Charandabi 2015	IRCT2013040810324N12	Mohammad-Alizadeh-Charandabi 2015	The effect of vitamin d and calcium plus vitamin d during pregnancy on pregnancy and birth outcomes: A randomized controlled trial
MDIG	NCT01924013	Roth 2018 [142]	Vitamin D Supplementation in Pregnancy and Lactation and Infant Growth
Mojibian 2015	IRCT201107237097N1	Mojibian 2015 [143]	The effects of vitamin d supplementation on maternal and neonatal outcome: A randomized clinical trial
Mwangi 2015	NCT01308112	Mwangi 2015 [144]	Effect of daily antenatal iron supplementation on plasmodium infection in kenyan women: A randomized clinical trial
Ndibazza 2011	NR	Ndibazza 2011 [145]	Effects of Deworming during Pregnancy on Maternal and Perinatal Outcomes in Entebbe, Uganda: A Randomized Controlled Trial
Ndyomugyenyi 2008	NR	Ndyomugyenyi 2008 [146]	Efficacy of ivermectin and albendazole alone and in combination for treatment of soil-

© 2019 Park JJH et al. *JAMA Network Open*.

Trial ID	Trial Registry	Study ID	Title
			transmitted helminths in pregnancy and adverse events: a randomized open label controlled intervention trial in Masindi district, western Uganda
ObaapaVitA	NCT00211341	Kirkwood 2010 [147]	Effect of vitamin A supplementation in women of reproductive age on maternal survival in Ghana (ObaapaVitA): a cluster-randomised, placebo-controlled trial
Osendarp 2000	NR	Osendarp 2000 [148]	A randomized, placebo-controlled trial of the effect of zinc supplementation during pregnancy outcome in Bangladeshi urban poor
Osrin 2005	ISRCTN88625934	Osrin 2005 [149]	Effects of antenatal micronutrient supplementation on birthweight and gestational duration in Nepal: double-blind, randomised controlled trial
Osrin 2005	ISRCTN88625934	Devakumar 2015 [150]	Effects of antenatal multiple micronutrient supplementation on lung function in mid-childhood: Follow-up of a double-blind randomised controlled trial in nepal
Owens 2015	ISRCTN13687662	Owens 2015 [151]	Periconceptional multiple-micronutrient supplementation and placental function in rural Gambian women: A double-blind, randomized, placebo-controlled trial
PRECONCEPT	NCT01665378	Ramakrishnan 2016A [152]	Neither preconceptional weekly multiple micronutrient nor iron-folic acid supplements affect birth size and gestational age compared with a folic acid supplement alone in rural vietnamese women: A randomized controlled trial
PROFEG	NCT00488579	Hemminki 2016 [153]	Is selective prenatal iron prophylaxis better than routine prophylaxis: Final results of a trial (profeg) in maputo, mozambique
PROFEG	NCT00488579	Nwaru 2015 [154]	A pragmatic randomised controlled trial on routine iron prophylaxis during pregnancy in maputo, mozambique (profeg): Rationale, design, and success
Purwar 1996	NR	Purwar 1996 [155]	Calcium supplementation and prevention of pregnancy induced hypertension
Radhika 2003	NR	Radhika 2003 [156]	Red palm oil supplementation: a feasible diet-based approach to improve the vitamin A status of pregnant women and their infants.
Ramakrishnan 2003	NR	Ramakrishnan 2003 [157]	Multiple micronutrient supplementation during pregnancy does not lead to greater infant birth size than does iron-only supplementation: a randomized controlled trial in a semirural community in Mexico
Rang-Din Nutrition study	NCT01715038	Mridha 2016 [158]	Lipid-based nutrient supplements for pregnant women reduce newborn stunting in a cluster-randomized controlled effectiveness trial in Bangladesh
Rang-Din Nutrition study	NCT01715038	Mridha 2017 [159]	Prenatal lipid-based nutrient supplements do not affect pregnancy or childbirth complications or cesarean delivery in Bangladesh: A cluster-randomized controlled effectiveness trial

Trial ID	Trial Registry	Study ID	Title
Rang-Din Nutrition study	NCT01715038	Matias 2016 [160]	Prenatal lipid-based nutrient supplements affect maternal anthropometric indicators only in certain subgroups of rural Bangladeshi women
Roberfroid 2008	NCT00642408	Roberfroid 2008 [161]	Effects of maternal multiple micronutrient supplementation on fetal growth: a double- blind randomized controlled trial in rural Burkina Faso
Ruangvutilert 2017	NCT00746551	Ruangvutilert 2017 [162]	Low-dose weekly intravenous iron sucrose versus daily oral iron for iron deficiency anemia in late pregnancy: A randomized controlled trial
Saaka 2009	NR	Saaka 2009 [163]	Effect of prenatal zinc supplementation on birthweight
Sablok 2015	NR	Sablok 2015 [164]	Supplementation of vitamin D in pregnancy and its correlation with feto-maternal outcome.
Sayyah-Melli 2016	IRCT201402175283N9	Sayyah-Melli 2016 [165]	The effect of high dose folic acid throughout pregnancy on homocysteine (hcy) concentration and pre-eclampsia: A randomized clinical trial
SUMMIT 2008	ISRCTN34151616	SUMMIT 2008 [166]	Effect of maternal multiple micronutrient supplementation on fetal loss and infant death in Indonesia: a double-blind cluster-randomised trial
Sunawang 2009	NR	Sunawang 2009 [167]	Preventing low birth weight through maternal multiple micronutrient supplementation: a cluster-randomized controlled trial in Indramayu, West Java
Taherian 2002	NR	Taherian 2002 [168]	Prevention of preeclampsia with low-dose aspirin or calcium supplementation
Villar 2006	NR	Villar 2006 [169]	World Health Organization randomized trial of calcium supplementation among low calcium intake pregnant women
Wanchu 2001	NR	Wanchu 2001 [170]	Calcium supplementation in pre-eclampsia
Whitfield 2016	NCT02221063	Whitfield 2016 [171]	Perinatal consumption of thiamine-fortified fish sauce in rural Cambodia: A randomized clinical trial
Yu 2008	NR	Yu 2008 [172]	Vitamin D deficiency and supplementation in pregnant women of four ethnic groups
Zagre 2007	NR	Zagre 2007 [173]	Prenatal multiple micronutrient supplementation has greater impact on birthweight than supplementation with iron and folic acid: a cluster-randomized, double-blind, controlled programmatic study in rural Niger
Zahiri Sorouri 2016	NR	Zahiri Sorouri 2016 [174]	The effect of zinc supplementation on pregnancy outcome: A randomized controlled trial
Zeng 2008	ISRCTN08850194	Zeng 2008 [175]	Impact of micronutrient supplementation during pregnancy on birth weight, duration of gestation and perinatal mortality in rural western China: double blind cluster randomised controlled trial
Zhao 2015	NCT02221752	Zhao 2015 [176]	Prenatal iron supplementation reduces maternal anemia, iron deficiency, and iron deficiency anemia in a randomized clinical trial in rural china, but iron deficiency remains widespread in mothers and neonates
Ziaei 2007	NR	Ziaei 2007 [177]	Iron status markers in nonanemic pregnant women with and without iron supplementation

© 2019 Park JJH et al. *JAMA Network Open*.

© 2019 Park JJH et al. *JAMA Network Open*.

eTable 9. List of included studies for exclusive breastfeeding life stage (0-6m)

Trial ID	Registry number	First author, year	Title
Acharya 2014[178]	NR	Acharya 2014	Randomized Control Trial of Kangaroo Mother Care in Low Birth Weight Babies at a Tertiary Level Hospital
Adu-Afarwuah 2016[71,179]	NCT00970866	Adu-Afarwuah 2016	Small-quantity, lipid-based nutrient supplements provided to women during pregnancy and 6 mo postpartum and to their infants from 6 mo of age increase the mean attained length of 18-mo-old children in semi-urban Ghana: a randomized controlled trial
		Adu-Afarwuah 2017	Maternal supplementation with small-quantity lipid-based nutrient supplements compared with multiple micronutrients, but not with iron and folic acid, reduces the prevalence of low gestational weight gain in semi-urban ghana: A randomized controlled trial
Ashorn 2015A[180,181]	NCT01239693	Ashorn 2015A	Supplementation of Maternal Diets during Pregnancy and for 6 Months Postpartum and Infant Diets Thereafter with Small-Quantity Lipid-Based Nutrient Supplements Does Not Promote Child Growth by 18 Months of Age in Rural Malawi: A Randomized Controlled Trial
		Ashorn 2015B	The impact of lipid-based nutrient supplement provision to pregnant women on newborn size in rural Malawi: a randomized controlled trial
Boo 2007[182]	NR	Boo 2007	Short duration of skin-to-skin contact: effects on growth and breastfeeding
CARING trial[87]	ISCRTN51505201	Nair 2017	Effect of participatory women's groups and counselling through home visits on children's linear growth in rural eastern india (caring trial): A cluster-randomised controlled trial
Feliciano 1994[183]	NR	Feliciano 1994	Seasonal and Geographical Variations in the Growth Rate of Infants in China Receiving Increasing Dosages of Vitamin D Supplements
Gathwala 2010[184]	NR	Gathwala 2010	Effect of Kangaroo Mother Care on physical growth, breastfeeding and its acceptability
Goodstart[185,186]	NR	Tomlinson 2011	An effectiveness study of an integrated, community-based package for maternal, newborn, child and HIV care in South Africa: study protocol for a randomized controlled trial
	NR	Tomlinson 2014	Goodstart: a cluster randomised effectiveness trial of an integrated, community-based package for maternal and newborn care, with prevention of mother-to-child transmission of HIV in a South African township
Habib 2015[187]	NCT01229579	Habib 2015	Zinc supplementation fails to increase the immunogenicity of oral poliovirus vaccine: A randomized controlled trial
Hamadani 2001[188]	NR	Hamadani 2001	Randomized controlled trial of the effect of zinc supplementation on the mental development of bangladeshi infants
JiVitA-3[189,190]	NCT00860470	Christian 2016	Effects of prenatal multiple micronutrient

© 2019 Park JJH et al. *JAMA Network Open*.

Trial ID	Registry number	First author, year	Title
			supplementation on growth and cognition through 2 y of age in rural Bangladesh: the JiVitA-3 Trial
		West 2014	Effect of maternal multiple micronutrient vs iron-folic acid supplementation on infant mortality and adverse birth outcomes in rural Bangladesh: the JiVitA-3 randomized trial.
Kumbhojkar 2016[191]	NR	Kumbhojkar 2016	Kangaroo Mother Care (KMC): An Alternative to Conventional Method of Care for Low Birth Weight Babies
Le Roux 2013[192,193]	NCT00996528	Le Roux 2013	Outcomes of home visits for pregnant mothers and their infants: a cluster randomized controlled trial
		Rotheram-Borus 2014	A Cluster Randomised Controlled Effectiveness Trial Evaluating Perinatal Home Visiting among South African Mothers/Infants
Locks 2016[194,195]	NCT00421668	Locks 2016	Effect of zinc and multivitamin supplementation on the growth of Tanzanian children aged 6–84 wk: a randomized, placebo-controlled, double-blind trial
		Locks L 2015	Effect of zinc & multiple micronutrient supplements on growth in tanzanian children
Lonnerdal 2017[196]	NCT00970398	Lonnerdal 2017	Growth, Nutrition, and Cytokine Response of Breast-fed Infants and Infants Fed Formula With Added Bovine Osteopontin
LUCOMAI[197]	NCT01977365	Nikiema 2017	Effectiveness of facility-based personalized maternal nutrition counseling in improving child growth and morbidity up to 18 months: A cluster-randomized controlled trial in rural Burkina Faso
MDIG[142]	NCT01924013	Roth 2018	Vitamin D Supplementation in Pregnancy and Lactation and Infant Growth
Osendarp 2002[198]	NR	Osendarp 2002	Effect of zinc supplementation between 1 and 6 mo of life on growth and morbidity of Bangladeshi infants in urban slums on the mental development of Bangladeshi infants
Ostadrahimi 2017[199]	NR	Ostadrahimi 2017	The effect of perinatal fish oil supplementation on neurodevelopment and growth of infants: a randomized<br>controlled trial
PROMISE EBF[200-202]	NCT00397150	Engebretsen 2014	Growth effects of exclusive breastfeeding promotion by peer counsellors in sub-Saharan Africa: the cluster-randomised PROMISE EBF trial
		Fadnes 2016	Effects of an exclusive breastfeeding intervention for six months on growth patterns of 4–5 year old children in Uganda: the cluster-randomised PROMISE EBF trial
		Tylleskar 2011	Exclusive breastfeeding promotion by peer counsellors in sub-Saharan Africa (PROMISE-EBF): a cluster-randomised trial.
RDNS[203,204]	NCT01715038	Dewey 2017	Lipid-based nutrient supplementation in the first 1000 d improves child growth in Bangladesh: a cluster-randomized effectiveness trial

© 2019 Park JJH et al. *JAMA Network Open*.

Trial ID	Registry number	First author, year	Title
		Mridha 2016	Lipid-based nutrient supplements for pregnant women reduce newborn stunting in a cluster-randomized controlled effectiveness trial in Bangladesh
Shafique 2016[205]	NCT01455636	Shafique 2016	Mineral- and vitamin-enhanced micronutrient powder reduces stunting in full-term low-birth-weight infants receiving nutrition, health, and hygiene education: A 2 x 2 factorial, cluster-randomized trial in bangladesh
Simondon 1996[206]	NR	Simondon 1996	Effect of early, short-term supplementation on weight and<br>linear growth of 4-7-mo-old infants in developing countries:<br>a four-country randomized trial
Suman 2008[207]	NR	Suman 2008	Kangaroo mother care for low birth weight infants: a randomized controlled trial
Urban 2008[208]	NR	Urban 2008	Growth of infants born to HIV infected women when fed a biologically acidified starter formula with and without probiotics
Vazir 2013[209]	NR	Vazir 2013	Cluster-randomized trial on complementary and responsive feeding education to caregivers found improved dietary intake, growth, and development among rural Indian toddlers
Velaphi 2008[210]	NR	Velaphi 2008	Growth and metabolism of infants born to women infected with human immunodeficiency virus and fed acidified whey-adapted starter formulas

© 2019 Park JJH et al. *JAMA Network Open*.

eTable 10. List of included studies for complementary feeding life stage (6-24m)

Trial ID	Trial Registry	First author, year	Title
Adu-Afarwuah 2007	NR	Adu-Afarwuah 2007[211]	Randomized comparison of 3 types of micronutrient supplements for home fortification of complementary foods in Ghana: effects on growth and motor development.
Ashorn 2015	NCT01239693	Ashorn 2015[80]	Supplementation of Maternal Diets during Pregnancy and for 6 Months Postpartum and Infant Diets Thereafter with Small-Quantity Lipid-Based Nutrient Supplements Does Not Promote Child Growth by 18 Months of Age in Rural Malawi: A Randomized Controlled Trial
Black 2004A	NR	Black 2004A[212]	Iron and zinc supplementation promote motor development and exploratory behavior among Bangladeshi infants
Black 2004A	NR	Fischer Walker 2009 [213]	Low-dose weekly supplementation of iron and/or zinc does not affect growth among Bangladeshi infants
Brown 2007	NR	Brown 2007[214]	Comparison of the effects of zinc delivered in a fortified food or a liquid supplement on the growth, morbidity, and plasma zinc concentrations of young Peruvian children
CARING trial	ISCRTN51505201	Nair 2017[87]	Effect of participatory women's groups and counselling through home visits on children's linear growth in rural eastern india (caring trial): A cluster-randomised controlled trial
Castillo-Duran 1995	NR	Castillo-Duran 1995[215]	Zinc supplementation and growth of infants born small for gestational age
Castillo-Duran 2001	NR	Castillo-Duran 2001[88]	Effect of zinc supplementation on development and growth of Chilean infants.
CIGNIS trial	ISRCTN37460449	Chilenje Infant Growth 2010[216]	Micronutrient fortification to improve growth and health of maternally hiv-unexposed and exposed zambian infants: A randomised controlled trial
CIGNIS trial	ISRCTN37460449	Filteau 2011 [217]	Provision of micronutrient-fortified food from 6 months of age does not permit hiv-exposed uninfected zambian children to catch up in growth to hiv-unexposed children: A randomized controlled trial
Cohen 1994A	NR	Cohen 1994A [218]	Determinants of growth from birth to 12 months among breast-fed Honduran infants in relation to age of introduction of complementary foods.
Dewey 2002	NR	Dewey 2002[219]	Iron supplementation affects growth and morbidity of breast-fed infants: Results of a randomized trial in sweden and honduras
Dijkhuizen 2001	NR	Dijkhuizen 2001[220]	Effects of iron and zinc supplementation in Indonesian infants on micronutrient status and growth.
Doherty 1998	NR	Doherty 1998[221]	Zinc and rehabilitation from severe protein-energy malnutrition: Higher-dose regimens are associated with increased mortality
Duggan 2003	NR	Duggan 2003 [222]	Oligofructose-supplemented infant cereal: 2 randomized, blinded, community-based trials in peruvian infants
Fahmida 2007	NR	Fahmida 2007	Zinc-iron, but not zinc-alone supplementation,

© 2019 Park JJH et al. *JAMA Network Open*.

Trial ID	Trial Registry	First author, year	Title
		[223]	increased linear growth of stunted infants with low haemoglobin
Fink 2017	NCT02242539	Fink 2017 [224]	Home- and community-based growth monitoring to reduce early life growth faltering: An open-label, cluster-randomized controlled trial
Giovannini 2006	NR	Giovannini 2006 [225]	Double-blind, placebo-controlled trial comparing effects of supplementation with two different combinations of micronutrients delivered as sprinkles on growth, anemia, and iron deficiency in Cambodian infants
Goto 2009	NCT00607074	Goto 2009 [226]	Impact of anti-giardia and anthelminthic treatment on infant growth and intestinal permeability in rural Bangladesh: a randomised double-blind controlled study
Helmizar 2017	NR	Helmizar 2017 [227]	Local food supplementation and psychosocial stimulation improve linear growth and cognitive development among indonesian infants aged 6 to 9 months
Hess 2015	NCT00944281	Hess 2015 [228]	Small-Quantity Lipid-Based Nutrient Supplements, Regardless of Their Zinc Content, Increase Growth and Reduce the Prevalence of Stunting and Wasting in Young Burkinabe Children: A Cluster-Randomized Trial
Iannotti 2014	NCT01552512	Iannotti 2014 [229]	Linear growth increased in young children in an urban slum of Haiti: a randomized controlled trial of a lipid-based nutrient supplement
Idjradinata 1993	NR	Idjradinata 1993 [230]	Reversal of developmental delays in iron-deficient anaemic infants treated with iron.
Idjradinata 1994	NR	Idjradinata 1994 [231]	Adverse effect of iron supplementation on weight gain of iron-replete young children
Jack 2012	ACTRN-12608000069358	Jack 2012 [232]	Effect of micronutrient Sprinkles on reducing anemia: a cluster-randomized effectiveness trial.
Jones 2015	NCT01593969	Jones 2015 [233]	Ready-to-use therapeutic food with elevated n-3 polyunsaturated fatty acid content, with or without fish oil, to treat severe acute malnutrition: A randomized controlled trial
Joseph 2015A	NCT01314937	Joseph 2015A [234]	The effect of deworming on growth in one-year-old children living in a soil-transmitted helminth-endemic area of peru: A randomized controlled trial
Joseph 2015A	NCT01314937	Joseph 2015B [235]	The effect of deworming on early childhood development in peru: A randomized controlled trial
Krebs 2012	NCT01084109	Krebs 2012[236]	Randomized controlled trial of meat compared with multimicronutrient-fortified cereal in infants and toddlers with high stunting rates in diverse settings
Kupka 2013	NCT00197730	Kupka 2013[237]	Multivitamin supplements have no effect on growth of tanzanian children born to hiv-infected mothers
Kuusipalo 2006	NR	Kuusipalo 2006 [238]	Growth and change in blood haemoglobin concentration among underweight Malawian infants receiving fortified spreads for 12 weeks: a preliminary trial
Lartey 1999	NR	Lartey 1999 [239]	A randomized, community-based trial of the effects of improved, centrally processed

© 2019 Park JJH et al. *JAMA Network Open*.

Trial ID	Trial Registry	First author, year	Title
			complementary foods on growth and micronutrient status of ghanaian infants from 6 to 12 mo of age
LCNI-5	NCT00524446	Mangani 2015 [240]	Effect of complementary feeding with lipid-based nutrient supplements and corn-soy blend on the incidence of stunting and linear growth among 6- to 18-month-old infants and children in rural malawi
LCNI-5	NCT00524446	Mangani 2013	Effect of complementary feeding with lipid-based nutrient supplements and corn-soy blend on the incidence of stunting and linear growth among 6- to 18-month-old infants and children in rural Malawi
LCNI-5	NCT00524446	Mangani 2014[241]	Providing lipid-based nutrient supplements does not affect developmental milestones among Malawian children
Lin 2008	NR	Lin 2008[242]	An energy-dense complementary food is associated with a modest increase in weight gain when compared with a fortified porridge in malawian children aged 6-18 months
Lind 2004	NR	Lind 2004 [243]	A community-based randomized controlled trial of iron and zinc supplementation in Indonesian infants: effects on growth and development
Locks 2016	NCT00421668	Locks 2016[244]	Effect of zinc and multivitamin supplementation on the growth of tanzanian children aged 6-84 wk: A randomized, placebo-controlled, double-blind trial
LUCOMA	NCT01977365	Nikiema 2017 [197]	Effectiveness of facility-based personalized maternal nutrition counseling in improving child growth and morbidity up to 18 months: A cluster-randomized controlled trial in rural burkina faso
Maleta 2015	NCT00945698	Maleta 2015 [245]	Provision of 10-40 g/d Lipid-Based Nutrient Supplements from 6 to 18 Months of Age Does Not Prevent Linear Growth Faltering in Malawi.
Mamiro 2004	NR	Mamiro 2004 [246]	Processed complementary food does not improve growth or hemoglobin status of rural tanzanian infants from 6-12 months of age in kilosa district, tanzania
Mazariegos 2010	NR	Mazariegos 2010 [247]	Neither a zinc supplement nor phytate-reduced maize nor their combination enhance growth of 6- to 12-month-old Guatemalan infants
Mda 2013	NR	Mda 2013 [248]	Impact of multi-micronutrient supplementation on growth and morbidity of hiv-infected south african children [248]
Medeiros 2015	NCT00701246	Medeiros 2015 [249]	The effect of folic acid supplementation with ferrous sulfate on the linear and ponderal growth of children aged 6-24 months: A randomized controlled trial
Muhoozi 2017	NCT02098031	Muhoozi 2017 [250]	Nutrition, hygiene, and stimulation education to improve growth, cognitive, language, and motor development among infants in uganda: A cluster-randomized trial
Newton 2016	NCT01751009	Newton 2016 [251]	Vitamin a status and body pool size of infants before and after consuming fortified home-based complementary foods
Oelofse 2003	NR	Oelofse 2003	The effect of a micronutrientfortified

© 2019 Park JJH et al. *JAMA Network Open*.

Trial ID	Trial Registry	First author, year	Title
		[252]	complementary food on micronutrient status, growth and development of 6- to 12-month-old disadvantaged urban South African infants
Olney 2006	NR	Olney 2006 [253]	Combined iron and folic acid supplementation with or without zinc reduces time to walking unassisted among Zanzibari infants 5- to 11-mo old
Osei 2015	NR	Osei 2015 [254]	Adding multiple micronutrient powders to a homestead food production programme yields marginally significant benefit on anaemia reduction among young children in nepal
Pham 2012	NR	Pham 2012 [255]	A six-month intervention with two different types of micronutrient-fortified complementary foods had distinct short- and long-term effects on linear and ponderal growth of vietnamese infants
Phuka 2009A	NCT00131222	Phuka 2009A [256]	Supplementary feeding with fortified spread among moderately underweight 6-18-month-old rural malawian children
Phuka 2012	NCT00131209	Phuka 2012 [257]	Developmental outcomes among 18-month-old malawians after a year of complementary feeding with lipid-based nutrient supplements or corn-soy flour
Phuka 2012	NCT00131209	Phuka 2009B [258]	Postintervention growth of Malawian children who received 12-mo dietary complementation with a lipid-based nutrient supplement or maize-soy flour.
Ramakrishnan 2009	NR	Ramakrishnan 2009 [259]	Multiple micronutrient supplementation during early childhood increases child size at 2 y of age only among high compliers
RDNS	NCT01715038	Dewey 2017 [260]	Lipid-based nutrient supplementation in the first 1000 d improves child growth in bangladesh: A cluster-randomized effectiveness trial
RDNS	NCT01715038	Matias S 2017 [261]	Effects of lipid-based nutrient supplements v. micronutrient powders on nutritional and developmental outcomes among Peruvian infants
Rivera 1998	NR	Rivera 1998 [262]	Zinc supplementation improves the growth of stunted rural Guatemalan infants
Rivera 2001	NR	Rivera 2001 [263]	Multiple micronutrient supplementation increases the growth of mexican infants
Saleem 2014	NCT01128517	Saleem 2014 [264]	Impact of maternal education about complementary feeding on their infants' nutritional outcomes in low- and middle-income households: A community-based randomized interventional study in karachi, pakistan
SEAMTIZI	NR	Dijkhuizen 2008 [265]	Zinc supplementation improved length growth only in anemic infants in a multi-country trial of iron and zinc supplementation in South-East Asia
SEAMTIZI	NR	Berger 2006 [266]	Efficacy of combined iron and zinc supplementation on micronutrient status and growth in Vietnamese infants
SEAMTIZI	NR	Walravens 1989 [267]	Iron and zinc supplementation improved iron and zinc status, but not physical growth, of apparently healthy, breast- fed infants in rural communities of northeast Thailand
SEAMTIZI	NR	Wieringa 2003 [268]	Redistribution of vitamin A after iron supplementation in Indonesian infants

© 2019 Park JJH et al. *JAMA Network Open*.

Trial ID	Trial Registry	First author, year	Title
Shafique 2016	NCT01455636	Shafique 2016 [205]	Mineral- and vitamin-enhanced micronutrient powder reduces stunting in full-term low-birth-weight infants receiving nutrition, health, and hygiene education: A 2 x 2 factorial, cluster-randomized trial in bangladesh
Shafique 2016	NCT01455636	Shafique S 2013 [269]	Prevention of linear growth faltering among low birth weight infants in rural bangladesh: A community-based cluster randomized trial
Shafique 2016	NCT01455636	Singla 2014 [270]	A 22-element micronutrient powder benefits language but not cognition in bangladeshi full-term low-birth-weight children
Skau 2015	ISRCTN19918531	Skau 2015 [271]	Effects of animal source food and micronutrient fortification in complementary food products on body composition, iron status, and linear growth: A randomized trial in cambodia
Smuts 2005A	NR	Smuts 2005A [272]	Efficacy of a foodlet-based multiple micronutrient supplement for preventing growth faltering, anemia, and micronutrient deficiency of infants: The four country iris trial pooled data analysis
Smuts 2005A	NR	Smuts 2005B [273]	Efficacy of multiple micronutrient supplementation for improving anemia, micronutrient status, and growth in south african infants
Taneja 2010	NCT00272116	Taneja 2010[274]	Zinc supplementation for four months does not affect growth in young north indian children
Tang 2014	NCT00726102	Tang 2014[275]	Meat as complementary food for older breastfed infants and toddlers: A randomized, controlled trial in rural china
Thakwalakwa 2012	NCT00420368	Thakwalakwa 2012[276]	An effectiveness trial showed lipid-based nutrient supplementation but not corn-soya blend offered a modest benefit in weight gain among 6- to 18-month-old underweight children in rural malawi
Thakwalakwa 2012	NCT00420368	Thakwalakwa 2010 [277]	A lipid-based nutrient supplement but not corn-soy blend modestly increases weight gain among 6- to 18-month-old moderately underweight children in rural Malawi
The JiVitA-4-trial	NR	Christian 2015 [278]	Effect of fortified complementary food supplementation on child growth in rural Bangladesh: A cluster-randomized trial
Thu 1999	NR	Thu 1999 [279]	Effect of daily and weekly micronutrient supplementation on micronutrient deficiencies and growth in young Vietnamese children
Umeta 2000	NR	Umeta 2000 [280]	Zinc supplementation and stunted infants in Ethiopia: a randomised controlled trial
Untoro 2005	NR	Untoro 2005 [281]	Multiple micronutrient supplements improve micronutrient status and anemia but not growth and morbidity of indonesian infants: A randomized, double-blind, placebo-controlled trial
Wasantwisut 2006	NR	Wasantwisut 2006 [282]	Iron and zinc supplementation improved iron and zinc status, but not physical growth, of apparently healthy, breast-fed infants in rural communities of northeast Thailand.
WASH B Bangladesh	NCT01590095	Luby 2018[283]	Effects of water quality, sanitation, handwashing, and nutritional interventions on diarrhoea and child growth in rural Bangladesh: a cluster randomised

© 2019 Park JJH et al. *JAMA Network Open*.

Trial ID	Trial Registry	First author, year	Title
			controlled trial
WASH B Kenya	NCT01704105	Null 2018[284]	Effects of water quality, sanitation, handwashing, and nutritional interventions on diarrhoea and child growth in rural Kenya: a cluster-randomised controlled trial
Williams 2007	NR	Williams 2007[285]	A double-blind, placebo-controlled, glutamine-supplementation trial in growth-faltering gambian infants
Zlotkin 2003	NR	Zlotkin 2003[286]	Home-Fortification with Iron and Zinc Sprinkles or Iron Sprinkles Alone Successfully Treats Anemia in Infants and Young Children

© 2019 Park JJH et al. *JAMA Network Open*.

List of Excluded studies for each life stage

eTable 11. List of excluded studies for pregnancy life stage with reasons

First author	Year	Title	Reason for exclusion
Adams	1978	Effect of nutritional supplementation in pregnancy. I. Outcome of pregnancy	Outcomes
Adu-Afarwuah S	2017	Prenatal supplementation with small-quantity lipid-based nutrient supplements or multiple micronutrients increases urinary iodine concentration in semi-urban Ghana: A randomized controlled trial	Other
Akbarzadeh	2016	Teaching attachment behaviors to pregnant women: A randomized controlled trial of effects on infant mental health from birth to the age of three months	Outcomes
Ali	2017	Effect of maternal antenatal and newborn supplementation with vitamin a on cognitive development of school-aged children in rural Bangladesh: A follow-up of a placebo-controlled, randomized trial	Population
Amarante	2011	Do cash transfers improve birth outcomes? Evidence from matched vital statistics, social security and program data	Study design
Aminisani	2009	Zinc supplementation during pregnancy: A randomized controlled trial	Outcomes
Amiri	2017	Can an educational intervention improve iodine nutrition status in pregnant women? A randomized controlled trial	Other
Atton	1990	Selective supplementation in pregnancy: effect on birth weight	Population
Azami	2017	The effects of multi mineral-vitamin d and vitamins (c+e) supplementation in the prevention of preeclampsia: An rct	Outcomes
Bah	2017	Serum hepcidin concentrations decline during pregnancy and may identify iron deficiency: Analysis of a longitudinal pregnancy cohort in the Gambia	Outcomes
Barber	2008	The impact of Mexico's conditional cash transfer programme, Oportunidades, on birthweight	Study design
Barber	2010	Empowering women: how Mexico's conditional cash transfer programme raised prenatal care quality and birth weight	Study design
Beau	2014	Pandemic A/H1N1 influenza vaccination during pregnancy: a comparative study using the EFEMERIS database	Study design
Blackwell	1973	Prospective maternal nutrition study in Taiwan: rationale, study design, feasibility and preliminary findings	Outcomes
Boggess	1997	A randomised controlled trial of the effect of third trimester calcium supplementation on maternal haemodynamic function	Outcomes
Bouckaert K	2017	Effect of multiple micronutrient supplementation in lactating women on infant growth and morbidity: A double-blind randomized controlled trial in rural burkina faso	Other
Brooke	1980	Vitamin D supplements in pregnant Asian women: effects on calcium status and fetal growth	Population
Brough	2010	Effect of multiple-micronutrient supplementation on maternal nutrient status, infant birth weight and gestational age at birth in a low-income, multi-ethnic population	Population
Brown	1983	Protein energy supplements in primigravid women at risk of low birthweight	Outcomes
Caan	1987	Benefits associated with wic supplemental feeding during the interpregnancy interval	Outcomes
Cantu	2013	Selective uptake of influenza vaccine and pregnancy outcomes	Study design
Cardoso	2016	The effect of vitamin e and vitamin c on the prevention of preeclampsia and newborn outcome: A case-control study	Study design
Chambers	2013	Risks and safety of pandemic H1N1 influenza vaccine in pregnancy: birth defects, spontaneous abortion, preterm delivery, and small for gestational age infants	Study design
Chandrasiri	2015	The impact of lipid-based nutrient supplementation on anti-malarial antibodies in pregnant women in a randomized controlled trial	Outcomes
Changamire	2015	Effect of multivitamin supplements on weight gain during pregnancy among hiv-negative women in tTanzania	Outcomes
Chapman	2004	Effectiveness of breastfeeding peer counseling in a low-income, predominantly latina population: A randomized controlled trial	Outcomes
Charles	2005A	Folic acid supplements in pregnancy and birth outcome: re-analysis of a large randomised controlled trial and update of Cochrane review	Population
Christian	2001	Maternal night blindness increases risk of mortality in the first 6 months of life among infants in nepal	Outcomes
Christian	2003	Effects of maternal micronutrient supplementation on fetal loss and infant mortality: A cluster-randomized trial in nepal	Outcomes
Christian	2003	Supplementation with micronutrients in addition to iron and folic acid does not further improve the hematologic status of pregnant women in rural nepal	Outcomes
Christian	2016	Antenatal micronutrient supplementation and third trimester cortisol and erythropoietin concentrations	Outcomes
Clearly	2014	2009A/H1N1 influenza vaccination in pregnancy: uptake and pregnancy out- comes - a historical cohort study	Study design
Cogswell	2003	Iron supplementation during pregnancy, anemia, and birth weight: a randomized	Population

© 2019 Park JJH et al. *JAMA Network Open*.

First author	Year	Title	Reason for exclusion
		controlled trial	
Crowther	1999	Calcium supplementation in nulliparous women for the prevention of pregnancy-induced hypertension, preeclampsia and preterm birth: an Australian randomized trial Fracog and the ACT study group	Population
Czeizel	1994	Postnatal somatic and mental development after periconceptional multivitamin supplementation	Outcomes
Darling	2017	Maternal dietary l-arginine and adverse birth outcomes in dar es salaam, Tanzania	Outcomes
Darling b	2017	Vitamin a and zinc supplementation among pregnant women to prevent placental malaria: A randomized, double-blind, placebo-controlled trial in Tanzania	Outcomes
Dewey	2017	Lipid-based nutrient supplementation in the first 1000 d improves child growth in Bangladesh: A cluster-randomized effectiveness trial	Outcomes
Dibley M	2017	Effect of iron-folic acid supplementation starting during early trimester of pregnancy on neonatal mortality: Findings from a large community-based randomized controlled trial in rural Bangladesh	Other
Ditai J	2016	A pilot cluster randomised trial of alcohol-based hand rub to prevent community neonatal sepsis in rural Uganda	Other
Dodds	2012	The association between influenza vaccine in pregnancy and adverse neonatal outcomes	Population
Dwarkanath	2016	Energy and protein supplementation does not affect protein and amino acid kinetics or pregnancy outcomes in underweight Indian women	Population
Elwood	1981	Child growth (0-5 years), and the effect of entitlement to a milk supplement	Population
Eskeland	1997	Iron supplementation in pregnancy: is less enough? A randomized, placebo controlled trial of low dose iron supplementation with and without heme iron	Population
Fabiani	2015	A/H1N1 pandemic influenza vaccination: A retrospective evaluation of adverse maternal, fetal and neonatal outcomes in a cohort of pregnant women in Italy	Study design
Fathi Maroufi	2017	Effects of high and low doses of folic acid on the soluble receptor activator of nuclear factor-kappa b ligand/osteoprotegerin ratio during pregnancy	Outcomes
Fawzi	1998	Randomised trial of effects of vitamin supplements on pregnancy outcomes and T cell counts in HIV-1-infected women in Tanzania	Outcomes
Fawzi	2005	Trial of zinc supplements in relation to pregnancy outcomes, hematologic indicators, and t cell counts among hiv-1-infected women in Tanzania	Outcomes
Fell	2012	H1N1 influenza vaccination during pregnancy and fetal and neonatal outcomes.	Study design
Fernald	2016	A cluster-randomized, controlled trial of nutritional supplementation and promotion of responsive parenting in Madagascar: The mahay study design and rationale	Other
Finkelstein	2017	Vitamin b12 status in pregnant women and their infants in south India	Outcomes
Friebert	2017	Adolescent pregnancy and nutrition: A subgroup analysis from the mamachiponde study in Malawi	Outcomes
Gernand	2015	Effects of prenatal multiple micronutrient supplementation on fetal growth factors: A cluster-randomized, controlled trial in rural Bangladesh	Outcomes
Girija	1984	Influence of dietary supplementation during pregnancy on lactation performance.	Outcomes
Gliles	1971	The effect of prescribing folic acid during pregnancy on birthweight and duration of pregnancy. A double blind trial	Population
Goldenberg	1995	The effect of zinc supplementation on pregnancy outcome	Population
Gonzalez-Casanova	2016	Maternal single nucleotide polymorphisms in the fatty acid desaturase 1 and 2 coding regions modify the impact of prenatal supplementation with dha on birth weight	Study design
Goonewardene	2017	Randomized control trial comparing effectiveness of weekly versus daily antenatal oral iron supplementation in preventing anemia during pregnancy	Outcomes
Grant	2014	Vitamin D during pregnancy and infancy and infant serum 25-hydroxyvitamin D concentration	Population
Gunaratna	2015	Multivitamin and iron supplementation to prevent periconceptional anemia in rural Tanzanian women: A randomized, controlled trial	Population
Haberg	2013	Risk of fetal death after pandemic influenza virus infection or vaccination	Study design
Harvey	2007	Effect of high-dose iron supplements on fractional zinc absorption and status in pregnant women	Population
Heikkinen	2012	Safety of MF59-adjuvanted A/ H1N1 influenza vaccine in pregnancy: a comparative cohort study	Study design
Hemminki	1991	A randomized comparison of routine vs selective iron supplementation during pregnancy.	Outcomes
Hollis	2011	Vitamin D supplementation during pregnancy: Double blind, randomized clinical trial of safety and effectiveness	Population
Hunt	1984	Zinc supplementation during pregnancy: effects on selected blood constituents and on progress and outcome of pregnancy in low-income women of Mexican descent	Population
Hunt	1985	Zinc supplementation during pregnancy in low income teenagers of Mexican descent: effects on selected blood constituents and on progress and outcome of pregnancy	Population
Huynh Dtt	2017	Maternal nutritional adequacy and gestational weight gain in vietnamese pregnant women	Other

© 2019 Park JJH et al. *JAMA Network Open*.

First author	Year	Title	Reason for exclusion
Iannotti	2008	Maternal zinc supplementation and growth in Peruvian infants	Outcomes
Ijumba	2015	Effect of an integrated community-based package for maternal and newborn care on feeding patterns during the first 12 weeks of life: A cluster-randomized trial in a south African township	Outcomes
Iyengar	1967	Effects of dietary supplements late in pregnancy on the expectant mother and her newborn	Outcomes
Jafarbegloo	2000	Gastrointestinal complications of ferrous sulfate in pregnant women: A randomized double-blind placebo-controlled trial	Outcomes
Jamilian	2016	Effects of probiotic supplementation on metabolic status in pregnant women: A randomized, double-blind, placebo-controlled trial	Outcomes
Johnson W	2016	Pre-pregnancy energy balance, gestational weight gain, and small-for-gestational age in rural Gambia: The early nutrition and immune development trial (isrctn49285450)	Other
Jonsson	1996	Zinc supplementation during pregnancy: a double blind randomised controlled trial	Population
Jorgensen	2017	Lipid-based nutrient supplements during pregnancy and lactation did not affect human milk oligosaccharides and bioactive proteins in a randomized trial	Outcomes
Kabir	2017	Identifying maternal and infant factors associated with newborn size in rural Bangladesh by partial least squares (pls) regression analysis	Study design
Kallen	2012	Vaccination against H1N1 influenza with Pandemrix(®) during pregnancy and delivery outcome: a Swedish register study.	Study design
Kambarami	2016	Factors associated with community health worker performance differ by task in a multi-tasked setting in rural Zimbabwe	Outcomes
Kamm	2016	Is pregnancy a teachable moment to promote handwashing with soap among primiparous women in rural Bangladesh? Follow-up of a randomised controlled trial	Outcomes
Kaseb	2002	Effect of traditional food supplementation during pregnancy on maternal weight gain and birthweight	Outcomes
Khan	2011	Effects of prenatal food and micronutrient supplementation on child growth from birth to 54 months of age: a randomized trial in Bangladesh	Outcomes
Klevor	2016	Lipid-based nutrient supplements providing approximately the recommended daily intake of vitamin a do not increase breast milk retinol concentrations among Ghanaian women	Outcomes
Kusin	1992	Energy supplementation during pregnancy and postnatal growth	Outcomes
Lechtig	1975	Effect of food supplementation during pregnancy on birthweight	Study design
Lee	2005	Effect of time of initiation and dose of prenatal iron and folic acid supplementation on iron and folate nutriture of Korean women during pregnancy	Study design
Legge	2014	Rates and determinants of seasonal influenza vaccination in pregnancy and association with neonatal outcomes	Population
Levine	1997	Trial of calcium to prevent preeclampsia	Population
Li	2017	Effects of prenatal micronutrient supplementation on spontaneous preterm birth: A double-blind randomized controlled trial in china	Outcomes
Luby	2018	Effects of water quality, sanitation, handwashing, and nutritional interventions on diarrhoea and child growth in rural Bangladesh: A cluster randomised controlled trial	Outcomes
Ludvigsson	2013	Influenza H1N1 vaccination and adverse pregnancy outcome	Study design
Mahomed	1989	Zinc supplementation during pregnancy: a double blind randomised controlled trial	Population
Makrides	2003	Efficacy and tolerability of low-dose iron supplements during pregnancy: a randomised controlled trial	Population
Mardones-Santander	1988	Effect of a milk-based food supplement on maternal nutritional status and fetal growth in underweight Chilean women	Outcomes
Matias	2017	Home fortification during the first 1000 d improves child development in Bangladesh: A cluster-randomized effectiveness trial	Outcomes
McDonald	1981	The Bacon Chow study: maternal nutrition supplementation and birth weight of offspring	Outcomes
Mehra S	2017	Big data methods and pupillary response: Cracking the code of dark adaptometry for vitamin a deficiency	Intervention
Meier	2003	Prevention of iron deficiency anemia in adolescent and adult pregnancies	Population
Merialdi	2004	Randomized controlled trial of prenatal zinc supplementation and fetal bone growth	Outcomes
Metcoff	1985	Effect of food supplementation (WIC) during pregnancy on birth weight	Population
Mikami	2017	Effect of prenatal counseling on breastfeeding rates in mothers of twins	Population
Mohammed H	2015	A cluster rct evaluating the effect of iodized salt on infant development in amhara region of Ethiopia	Population
Mora	1978	The effect of nutritional supplementation on calorie and protein intake of pregnant women	Outcomes
Mora	1979	Nutritional supplementation and the outcome of pregnancy. I. Birth weight	Outcomes
Mridha	2017	Daily consumption of lipid-based nutrient supplements containing 250 mug iodine does not increase urinary iodine concentrations in pregnant and postpartum women in Bangladesh	Outcomes
Mwangi Mn	2015	Antenatal iron supplementation and serum non-transferrin bound iron in Kenyan women: A randomized placebo-controlled trial	Intervention

© 2019 Park JJH et al. *JAMA Network Open*.

First author	Year	Title	Reason for exclusion
Nanthini	2017	A comparative prospective study to assess the efficacy and safety of iron sucrose versus iron sorbitol citric acid in pregnant women with iron deficiency anemia in a tertiary care hospital	Other
Nguyen	2016	Impact of preconception micronutrient supplementation on anemia and iron status during pregnancy and postpartum: A randomized controlled trial in rural Vietnam	Outcomes
Nguyen	2017	Integrating nutrition interventions into an existing maternal, neonatal, and child health program increased maternal dietary diversity, micronutrient intake, and exclusive breastfeeding practices in Bangladesh: Results of a cluster-randomized program evaluation	Outcomes
Nguyen P	2016	The influence of maternal nutrition on fetal size based on ultrasound measurements throughout pregnancy	Other
Nguyen P	2017	Feasibility and impacts of integrating nutrition interventions into an existing maternal, neonatal, and child health platform in Bangladesh	Other
Normando	2016	Calcium plus vitamin d supplementation during pregnancy interacts with polymorphisms in the promoter region of the vdr gene to affect postpartum bone mass of Brazilian adolescent mothers: A randomized controlled trial	Outcomes
Null	2018	Effects of water quality, sanitation, handwashing, and nutritional interventions on diarrhoea and child growth in rural Kenya: A cluster-randomised controlled trial	Outcomes
Nwaru	2015	Adherence in a pragmatic randomized controlled trial on prophylactic iron supplementation during pregnancy in Maputo, Mozambique	Outcomes
Oaks	2016	Late-pregnancy salivary cortisol concentrations of Ghanaian women participating in a randomized controlled trial of prenatal lipid-based nutrient supplements	Outcomes
Oaks	2017	Effects of a lipid-based nutrient supplement during pregnancy and lactation on maternal plasma fatty acid status and lipid profile: Results of two randomized controlled trials	Outcomes
Okronipa	2018	Maternal supplementation with small-quantity lipid-based nutrient supplements during pregnancy and lactation does not reduce depressive symptoms at 6 months postpartum in Ghanaian women: A randomized controlled trial	Outcomes
Okronipa Het	2016	The impact of lipid-based nutrient supplements on maternal depression at 6 mo postpartum in Ghana: A randomized-controlled trial	Other
Omotayo	2017	A simplified regimen compared with who guidelines decreases antenatal calcium supplement intake for prevention of preeclampsia in a cluster-randomized noninferiority trial in rural Kenya	Outcomes
Osendarp	2000	A randomized, placebo-controlled trial of the effect of zinc supplementation during pregnancy on pregnancy outcome in Bangladeshi urban poor	Outcomes
Osendarp	2001	Zinc supplementation during pregnancy and effects on growth and morbidity in low birthweight infants: A randomised placebo controlled trial	Outcomes
Otoo G	2016	Effect of nutrition education with an emphasis on consumption of iron-rich foods on hemoglobin levels of pregnant women in Ghana	Other
Pastemak	2012A	Risk of adverse fetal outcomes following administration of a pandemic influenza A (H1N1) vaccine during pregnancy	Study design
Pasternak	2012B	Vaccination against pandemic A/H1N1 2009 influenza in pregnancy and risk of fetal death: cohort study in Denmark.	Study design
Pedley	2016	Improving motor development in infancy with iron supplementation	Other
Prado a	2016	Effects of maternal and child lipid-based nutrient supplements on infant development: A randomized trial in Malawi	Outcomes
Prentice	1987	Increased birthweight after prenatal dietary supplementation of rural African women	Outcomes
Preziosi	1997	Effect of iron supplementation on the iron status of pregnant women: Consequences for newborns	Outcomes
Rashid	2016	Temperature during pregnancy influences the fetal growth and birth size	Outcomes
Rasmussen	2010	Maternal supplementation differentially affects the mother and newborn	Study design
Rezk	2016	Lactoferrin versus ferrous sulphate for the treatment of iron deficiency anemia during pregnancy: A randomized clinical trial	Intervention
Robertson	1991b	Zinc supplementation during pregnancy	Population
Ross	1985	Differing effects of low and high bulk maternal dietary supplements during pregnancy	Population
Rostami	2017	Rationale and design of khuzestan vitamin d deficiency screening program in pregnancy: A stratified randomized vitamin d supplementation controlled trial	Outcomes
Roth D	2017	Maternal vitamin d supplementation during pregnancy and lactation to promote infant growth in dhaka, Bangladesh (mdig trial): A randomized controlled trial	Study design
Rubinstein	2013	Influenza A/H1N1 MF59 adjuvanted vaccine in pregnant women and adverse perinatal outcomes: multicentre study	Study design
Rush	1980	A randomized controlled trial of prenatal nutritional supplementation in New York City	Population
Sanchez-Ramos	1994	Prevention of pregnancy induced hypertension by calcium supplementation in angiotensin II sensitive patients	Population
Sanchez-Ramos	1995	Calcium supplementation in mild preeclampsia remote from term: a randomized double clinical trial	Population
Schlossman N	2015	Effects of two ready to use supplementary foods (rusf) containing different levels of	Other

© 2019 Park JJH et al. *JAMA Network Open*.

First author	Year	Title	Reason for exclusion
		dairy protein on mother's nutritional status in rural guinea-bissau	
Schmidt	2001	Vitamin A and iron supplementation of Indonesian pregnant women benefits vitamin A status of their infants	Outcomes
Schmidt	2002	Randomised double-blind trial of the effect of vitamin a supplementation of Indonesian pregnant women on morbidity and growth of their infants during the first year of life	Outcomes
Siddiqua	2016	Vitamin b12 supplementation during pregnancy and postpartum improves b12 status of both mothers and infants but vaccine response in mothers only: A randomized clinical trial in Bangladesh	Outcomes
Siega-Riz	2001	The effects of prophylactic iron given in prenatal supplements on iron status and birth outcomes: a randomized controlled trial.	Population
Simmer	1991a	A double-blind trial of zinc supplementation in pregnancy	Population
Srinivasan	2017	Effects of maternal vitamin b12 supplementation on early infant neurocognitive outcomes: A randomized controlled clinical trial	Outcomes
Stewart	2017	The impact of maternal diet fortification with lipid-based nutrient supplements on postpartum depression in rural Malawi: A randomised-controlled trial	Outcomes
Sumarmi S	2017	Prolonging micronutrients supplementation 2-6 months prior to pregnancy significantly improves birth weight by increasing hpl production and controlling il-12 concentration: A randomized double blind controlled study	Other
Tariq	2015	Parenteral iron therapy in the treatment of iron deficiency anemia during pregnancy: A randomized controlled trial	Outcomes
Taylor	1982	Effect of iron supplementation on serum ferritin levels during and after pregnancy	Population
Tofail	2008	Effects of prenatal food and micronutrient supplementation on infant development: a randomized trial from maternal and infant nutrition intervention, Matlab (MINIMat) study	Outcomes
Tontisirin	1986	Formulation and evaluation of supplementary foods for Thai pregnant women	Outcomes
Ullah B	2017	Effect of pre-and postnatal nutritional supplements on childhood illnesses in Bangladesh: A cluster-randomized effectiveness trial	Other
Van den Broek	2006	Randomised trial of vitamin A supplementation in pregnant women in rural Malawi found to be anaemic on screening by HemoCue	Outcomes
Viegas	1982A	Dietary protein energy supplementation of pregnant Asian mothers at Sorrento, Birmingham. II: Selective during third trimester only	Population
Viegas	1982B	Dietary protein energy supplementation of pregnant Asian mothers at Sorrento, Birmingham. I: Unselective during second and third trimesters	Population
Villar	1987	Calcium supplementation reduces blood pressure during pregnancy: results of a randomized controlled clinical trial	Population
Villar	1990	Calcium supplementation during pregnancy may reduce preterm in high-risk populations.	Population
Ward Ka	2016	Maternal calcium supplementation and offspring growth	Other
West	1999	Double blind, cluster randomised trial of low dose supplementation with vitamin A or beta carotene on mortality related to pregnancy in Nepal	Outcomes
Yu	2009	Vitamin D deficiency and supplementation during pregnancy	Population

© 2019 Park JJH et al. *JAMA Network Open*.

First author	Year	Title	Reason for exclusion
Ziaei	2016	A prenatal multiple micronutrient supplement produces higher maternal vitamin b-12 concentrations and similar folate, ferritin, and zinc concentrations as the standard 60-mg iron plus 400-mg folic acid supplement in rural Bangladeshi women	Outcomes

© 2019 Park JJH et al. *JAMA Network Open*.

eTable 12. List of excluded studies for exclusive breastfeeding stage with reasons

First author, year	Title	Reason
Aaby 2010	Non-specific effects of standard measles vaccine at 4.5 and 9 months of age on childhood mortality: Randomised controlled trial	Intervention
Aakko 2017	Lipid-based nutrient supplements do not affect gut bifidobacterium microbiota in Malawian infants: A randomized trial	Outcome
Adair 1994	Growth dynamics during the first two years of life: A prospective study in the Philippines	Study design
Adams 2017	Maternal and child supplementation with lipid-based nutrient supplements, but not child supplementation alone, decreases self-reported household food insecurity in some settings	Outcome
Adu-Afarwuah 2007	Randomized comparison of 3 types of micronutrient supplements for home fortification of complementary foods in Ghana: effects on growth and motor development.	Population
Aggarwal 2016	Selenium supplementation for prevention of late-onset sepsis in very low birth weight preterm neonates	Outcome
Agostoni 1995	Neurodevelopmental quotient of healthy term infants at 4 months and feeding practice: The role of long-chain polyunsaturated fatty acids	Outcome
Agostoni 1997	Developmental quotient at 24 months and fatty acid composition of diet in early infancy: A follow up study.	Other
Agostoni 2007	Functional ingredients in the complementary feeding period and long-term effects.	Study design
Agudelo 2016	The effect of skin-to-skin contact at birth, early versus immediate, on the duration of exclusive human lactancy in full-term newborns treated at the clinica universidad de la sabana: Study protocol for a randomized clinical trial	Other
Ahmad 2015	The effect of postpartum vitamin a supplementation on breast milk immune regulators and infant immune functions: Study protocol of a randomized, controlled trial	Other
Ahmad 2016	Maternal zinc supplementation improves hepatitis b antibody responses in infants but decreases plasma zinc level	Outcome
Ahmed 2008	Breastfeeding preterm infants: an educational program to support mothers of preterm infants in Cairo, Egypt	Outcome
Ahn 1980	Growth of the exclusively breast-fed infant.	Study design
Akeson 1996	Human milk and standard infant formula together with high quality supplementary foods is sufficient for normal growth during infancy.	Study design
Alarcon 1991	Clinical trial of home available, mixed diets versus a lactose-free, soy-protein formula for the dietary management of acute childhood diarrhea	Population
Alarcon 1992	Effect of inclusion of beans in a mixed diet for the treatment of Peruvian children with acute watery diarrhea	Population
Alarcon 2003	Effect of oral supplementation on catch-up growth in picky eaters	Population
Ali 2009	Kangaroo Mother Care as compared to conventional care for low birth weight babies Düşük doğum ağırlıklı bebekler için Kanguru anne bakımının geleneksel bakımla karşılaştırılması	Outcome
Ali 2017	Effect of maternal antenatal and newborn supplementation with vitamin a on cognitive development of school-aged children in rural Bangladesh: A follow-up of a placebo-controlled, randomized trial	Study design
Altmann M 2017	The impact of adding a household wash package to cmam program: A cluster-randomized controlled trial in chad	Outcome
Anderson 2003	Early skin-to-skin contact for mothers and their healthy newborn infants.	Outcome
Anderson 2010	Effects of maternal micronutrient supplementation on fetal loss and under- 2-years child mortality: long-term follow-up of a randomised controlled trial from Guinea-Bissau	Outcome
Angeles 1993	Decreased rate of stunting among anemic Indonesian preschool children through iron supplementation	Population
Anonymous 2017A	Effect of heat application during intramuscular injection of vitamin k in pain prevention in neonates	Outcome
Anonymous 2017B	Effects of preconceptional weekly micronutrient supplements on maternal and child anemia during the first 2 years of life	Outcome
Armbrust 2016	The Charite cesarean birth: a family orientated approach of cesarean section	Outcome
Ash 2003	Randomized efficacy trial of a micronutrient-fortified beverage in primary school children in Tanzania	Population
Auerbach 1990	Sequential and simultaneous breast pumping: a comparison.	Outcome
Auestad 1997	Visual acuity, erythrocyte fatty acid composition, and growth in term infants fed formulas with long chain polyunsaturated fatty acids for one year.	Population
Auestad 2001	Growth and development in term infants fed long-chain polyunsaturated fatty acids: A double-masked, randomized, parallel, prospective, multivariate study.	Population
Awasthi 2005	Effectiveness of biweekly versus daily iron-folic acid administration on anaemia status in preschool children	Population
Banupriya 2018	Short term oral zinc supplementation among babies with neonatal sepsis for reducing mortality and improving outcome - a double-blind randomized controlled trial	Outcome
Baqui 2003	Simultaneous weekly supplementation of iron and zinc is associated with lower morbidity	Outcome

© 2019 Park JJH et al. *JAMA Network Open*.

First author, year	Title	Reason
	due to diarrhea and acute lower respiratory infection in Bangladeshi infants	
Bass 2007	Exercise and calcium combined results in a greater osteogenic effect than either factor alone: a blinded randomized placebo-controlled trial in boys	Other
Bates 1993	A trial of zinc supplementation in young rural Gambian children.	Outcome
Beiranvand 2014	The effects of skin-to-skin contact on temperature and breastfeeding successfulness in full-term newborns after cesarean delivery.	Outcome
Benn 1997	Randomised trial of effect of vitamin A supplementation on antibody response to measles vaccine in Guinea-Bissau, West Africa	Outcome
Berger 2006	Efficacy of combined iron and zinc supplementation on micronutrient status and growth in Vietnamese infants	Population
Bergman 2004	Kangaroo mother care from birth compared to conventional incubator care	Outcome
Bernabe-Garcia 2012	Effectiveness of four manual breast pumps for mothers after preterm delivery in a developing country	Outcome
Bhan 1988	Comparison of a lactose-free cereal-based formula and cow's milk in infants and children with acute gastroenteritis	Population
Bhandari 2001	Food supplementation with encouragement to feed it to infants from 4 to 12 months of age has a small impact on weight gain	Population
Bhandari 2002	Effect of routine zinc supplementation on pneumonia in children aged 6 months to 3 years: randomised controlled trial in an urban slum	Outcome
Bhandari 2007	Adding zinc to supplemental iron and folic acid does not affect mortality and severe morbidity in young children	Outcome
Bhatia and Seshadri 1992	Growth performance in anemia and following iron supplementation	Population
Birch 1998	Visual acuity and the essentiality of docosahexaenoic acid and arachidonic acid in the diet of term infants	Population
Birch 2000	A randomized controlled trial of early dietary supply of LCP and mental development in term infants.	Population
Birch 2002	A randomized controlled trial of long-chain polyunsaturated fatty acid supplementation of formula in term infants after weaning at 6 wk of age	Population
Birungi 2015	Effect of breastfeeding promotion on early childhood caries and breastfeeding duration among 5 year old children in eastern Uganda: A cluster randomized trial	Outcome
Black 2004A	Iron and zinc supplementation promote motor development and exploratory behavior among Bangladeshi infants	Population
Black 2004B	Cognitive and motor development among small-for-gestational-age infants: impact of zinc supplementation, birth weight, and caregiving practices.	Outcome
Blaymore Bier 1996	Comparison of Skin-to-Skin Contact With Standard Contact in Low-Birth-Weight Infants Who Are Breast-Fed	Outcome
Boisson 2013	Effect of Household-Based Drinking Water Chlorination on Diarrhoea Among Children Under Five in Orissa, India: a Double-Blind Randomised Placebo-Controlled Trial	Population
Bonjour 1997	Calcium-enriched foods and bone mass growth in prepuber- tal girls: a randomized, double-blind, placebo-controlled trial	Other
Boo 2001	Contamination of breast milk obtained by manual expression and breast pumps in mothers of very low birthweight infants	Outcome
Borg B 2017	Efficacy of a locally-produced multiple micronutrient-fortified ready-to-use supplementary food (rusf) for children under two years in cambodia	Outcome
Bouckaert K 2017	Effect of multiple micronutrient supplementation in lactating women on infant growth and morbidity: A double-blind randomized controlled trial in rural burkina faso	Other
Bougma K 2015	Iodized salt improves child's iodine status, mental development, and physical growth in a cluster randomized trial in ethiopia	Other
Boutte 1985	Comparison of hand and electric operated breast pumps.	Outcome
Briceno 2017	Are there synergies from combining hygiene and sanitation promotion campaigns: Evidence from a large-scale cluster-randomized trial in rural tanzania	Outcome
Brooks 2005	Effect of weekly zinc supplements on incidence of pneumonia and diarrhoea in children younger than 2 years in an urban, low-income population in Bangladesh:randomised controlled trial	Population
Brown 1991	Complementary Feeding of Young children in Developing Countries: a Review of Current Scientific Knowledge	Study design
Brown 1991	Clinical trial of modified whole milk, lactose-hydrolyzed whole milk, or cereal-milk mixtures for the dietary management of acute childhood diarrhea	Population
Brown 2007	Comparison of the effects of zinc delivered in a fortified food or a liquid supplement on the growth, morbidity, and plasma zinc concentrations of young Peruvian children	Population
Brown 2015	Acceptability of two ready-to-use therapeutic foods by HIV-positive patients in vietnam	Outcome
Burton 2013	Randomized trial comparing the effectiveness of two electric breast pumps in the NICU	Outcome
Bystrova 2003	Skin- to-skin contact may reduce negative consequences of “the stress of being born”: a study on temperature in newborn infants, subjected to	Outcome

© 2019 Park JJH et al. *JAMA Network Open*.

First author, year	Title	Reason
	different ward routines in St. Petersburg	
Cameron 2004	The effect of calcium supplementation on bone density in premenarcheal females: a co-twin approach	Population
Cameron Sl 2015	Lactation consultant support from late pregnancy with an educational intervention at 4 months of age delays the introduction of complementary foods in a randomized controlled trial	Outcome
Campbell 2016	Effect of complementary food supplementation on breastfeeding and home diet in rural Bangladeshi children	Outcome
Campbell 2016a	Water, sanitation and hygiene related risk factors for soil-transmitted helminth and giardia duodenalis infections in rural communities in timor-leste	Study design
Campbell 2017a	Investigations into the association between soil-transmitted helminth infections, haemoglobin and child development indices in manufahi district, timor-leste	Study design
Campbell 2017b	Water, sanitation and hygiene (wash) and environmental risk factors for soil-transmitted helminth intensity of infection in timor-leste, using real time pcr	Study design
Campbell R 2015	Children receiving complementary food supplements (cfs) have higher nutrient intakes from home foods in jivita-4 trial rural Bangladesh	Outcome
Carfoot 2004	The value of a pilot study in breast-feeding research	Outcome
Carfoot 2005	A randomised controlled trial in the north of England examining the effects of skin-to-skin care on breast feeding	Outcome
Carlsen 2013	Telephone-based support prolongs breastfeeding duration in obese women: a randomized trial	Outcome
Carlson 1996	A randomized trial of visual attention of preterm infants fed docosahexaenoic acid until two months	Outcome
Carlson 1997	Functional effects of increasing omega-3 fatty acid intake	Outcome
Carlsson 1978	Effects of amount of contact between mother and child on the mother’s nursing behavior	Outcome
Carter Rc 2015	Effects of zinc and multivitamin supplementation on hematologic status during infancy	Outcome
Castillo 1996	Breast-feeding and the Nutritional Status of Nursing Children in Chile	Study design
Castillo-Durán 1994	Zinc supplementation increases growth velocity of male children and adolescents with short stature	Population
Castillo-Duran 1995	Zinc supplementation and growth of infants born small for gestational age	Population
Castillo-Duran 2001	Effect of zinc supplementation on development and growth of Chilean infants.	Population
Cattaneo 1998	Kangaroo mother care for low birthweight infants: a randomized controlled trial in different settings	Outcome
Cavan 1993	Growth and body composition of periurban Guate- malan children in relation to zinc status: a longitudinal zinc intervention trial	Population
Cha 2015	The effect of improved water supply on diarrhea prevalence of children under five in the volta region of Ghana: A cluster-randomized controlled trial	Outcome
Chang 2010	Supplementing iron and zinc: Double blind, randomized evaluation of separate or combined delivery	Outcome
Charpak 1997	A randomized, controlled trial of kangaroo mother care: results of follow-up at 1 year of corrected age.	Outcome
Charpak 2001	A Randomized, Controlled Trial of Kangaroo Mother Care: Results of Follow-Up at 1 Year of Corrected Age	Outcome
Chen 2012	Effects of vitamin A, vitamin A plus zinc, and multiple micronutrients on anemia in preschool children in Chongqing, China	Population
Cheng 1993	Impact of large-dose vitamin A supplementation on childhood diarrhoea, respiratory disease and growth	Other
Cheung 2016	Gut microbiota in Malawian infants in a nutritional supplementation trial	Outcome
Chevalley 2005	Skeletal site selectivity in the effects of calcium supplementation on areal bone mineral density gain: a randomized, double-blind, placebo-controlled trial in prepubertal boys	Other
Chew 1993	Is dilution of cows' milk formula necessary for dietary management of acute diarrhoea in infants aged less than 6 months?	Population
Chhagan 2009	Effect of micronutrient supplementation on diarrhoeal disease among stunted children in rural South Africa	Outcome
Chhagan 2010	Effect on longitudinal growth and anemia of zinc or multiple micronutrients added to vitamin a: A randomized controlled trial in children aged 6-24 months	Population
Chi Luong 2016	Newly born low birthweight infants stabilise better in skin-to-skin contact than when separated from their mothers: A randomised controlled trial	Outcome
Chilenje Infant Growth 2010	Micronutrient fortification to improve growth and health of maternally HIV-unexposed and exposed Zambian infants: A randomised controlled trial	Population
Christensson 1992	Temperature, metabolic adaptation and crying in healthy full-term newborns cared for skin-to-skin or in a cot	Outcome
Christensson 1995	Separation distress call in the human neonate in the absence of maternal body contact	Outcome
Christian 2003A	Effects of alternative maternal micronutrient supplements on low birth weight in rural Nepal: double blind randomised community trial	Outcome

© 2019 Park JJH et al. *JAMA Network Open*.

First author, year	Title	Reason
Christian 2003B	Effects of maternal micronutrient supplementation on fetal loss and infant mortality: a cluster-randomized trial in Nepal.	Outcome
Christian 2013	Effects of vitamin A and beta-carotene supplementation on birth size and length of gestation in rural Bangladesh: a cluster-randomized trial	Outcome
Christian 2015	Effect of fortified complementary food supplementation on child growth in rural Bangladesh: A cluster-randomized trial	Population
Chwang 1988	Iron supplementation and physical growth of rural Indonesian children	Population
Chwo 1999	Early kangaroo care for 34-36 week preterm infants: effects on temperature, weight, cortisol, and behavior [dissertation]	Outcome
Ciliberto 2005	Comparison of home-based therapy with ready-to-use therapeutic food with standard therapy in the treatment of malnourished Malawian children: A controlled, clinical effectiveness trial	Population
Clarke Se 2015	Seasonal malaria chemoprevention combined with micronutrient supplementation delivered through community preschools: Findings from a cluster randomized trial in mali	Other
Clarke Se 2016	Impact of micronutrient supplementation combined with malaria chemoprevention on malaria, anaemia and cognitive development in early childhood: Findings from a cluster randomized study in southern mali	Other
Clasen 2012	The Effect of Improved Rural Sanitation on Diarrhoea and Helminth Infection: Design of a Cluster- randomized Trial in Orissa, India	Study design
Clasen T 2014	The effectiveness of a rural sanitation intervention on health and orissa, India: A clusterrandomized, controlled trial	Population
Cohen 1994	Determinants of Growth From Birth to 12 Months Amon Breast-Fed Honduran Infants in Relation to Age of Introduction of Comlementary Foods	Study design
Cohen 1994A	Determinants of growth from birth to 12 months among breast-fed Honduran infants in relation to age of introduction of complementary foods.	Population
Coles 2015	Dose and timing of prenatal alcohol exposure and maternal nutritional supplements: Developmental effects on 6-month-old infants	Outcome
Correia-Santos 2011	Dietary supplements for the lactating adolescent mother: influence on plasma micronutrients [Suplementos dieteticos opara la madre adolescente en periodo de lactancia: su influencia en micronutrirnetes del plasma]	Population
Costa 1989	A comparison of colony counts of breast milk using two methods of breast cleansing	Outcome
Courteix 2005	Cumulative effects of calcium supplementation and physical activity on bone accretion in premenarchal children: a double-blind randomised placebo-controlled trial	Population
Craig 1982	The effect of early contact on maternal perception of infant behavior	Other
Cruz Rj 2016	Randomized controlled trialon the effect of 10 grams moringa oleifera powder leaves on the level of hemoglobin and hematocrit on infants age 6-9 months	Other
Curry 1982	The effect of skin-to-skin contact between mother and infant during the first hour following delivery on the mother's maternal attachment behavior and self concept [dissertation]	Outcome
da Cunha 2016	Breast milk supplementation and preterm infant development after hospital discharge: A randomized clinical trial	Outcome
Daly Williams 1999	Iron supplemented formula milk related to reduction in psychomotor decline in infants from inner city areas: randomised study	Outcome
Dandamrongrak 2016	Correlation between iodine supplement in pregnancy and neonatal tsh level	Outcome
de Almeida 2005	Effect of fortification of drinking water with iron plus ascorbic acid or with ascorbic acid alone on hemoglobin values and anthropometric indicators in preschool children in day-care centers in southeast brazil	Population
De Carvalho 1985	Frequency of milk expression and milk production by mothers of nonnursing premature neonates	Outcome
De Chateau 1977	Long-term effect on mother- infant behaviour of extra contact during the first hour post partum. II. A follow-up at three months	Outcome
De Moura 2015	Cassava intake and vitamin a status among women and preschool children in akwa- ibom, nigeria	Population
de Paula 2001	The use of sugar fortified with iron tris-glycinate chelate in the prevention of iron deficiency anemia in preschool children	Outcome
Dehghani 2015	A randomized controlled trial of kangaroo mother care versus conventional method on vital signs and arterial oxygen saturation rate in newborns who were hospitalized in neonatal intensive care unit	Outcome
Desai 2015	The shine trial infant feeding intervention: Pilot study of effects on maternal learning and infant diet quality in rural Zimbabwe	Outcome
Dewey 1997	A randomized intervention study of the effects of discontinuing coffee intake on growth and morbidity of iron-deficient Guatemalan toddlers	Intervention
Dewey 1999	Age of introduction of complementary foods and growth of term, low-birth-weight, breast-fed infants: a randomized intervention study in Honduras	Study design
Dewey 1999A	Age of introduction of complementary foods and growth of term, low-birth-weight, breast-fed infants: a randomized intervention study in Honduras.	Outcome

© 2019 Park JJH et al. *JAMA Network Open*.

First author, year	Title	Reason
Dewey 2002	Iron supplementation affects growth and morbidity of breast-fed infants: Results of a randomized trial in Sweden and honduras	Population
Diaz-gomez 2003	The effect of zinc supplementation on linear growth, body composition, and growth factors in preterm infants	Population
Dibba 2000	Effect of calcium supplementation on bone mineral accretion in Gambian children accustomed to a low-calcium diet	Outcome
Dijkhuizen 2001	Effects of iron and zinc supplementation in Indonesian infants on micronutrient status and growth.	Population
Dijkhuizen 2008	Zinc supplementation improved length growth only in anemic infants in a multi-country trial of iron and zinc supplementation in South-East Asia	Population
Doherty 1998	Zinc and rehabilitation from severe protein-energy malnutrition: Higher-dose regimens are associated with increased mortality	Population
Domellof 2001	Iron requirements of term, breast-fed infants: a study in Sweden and Honduras.	Outcome
Dossa 2001	Multivitamin-multimineral and iron supplementation did not improve appetite of young stunted and anemic Beninese children	Outcome
Dossa 2002	Effects of multivitamin-multimineral supplementation on appetite of stunted young Beninese children	Outcome
du Preez 2010	Solar disinfection of drinking water in the prevention of dysentery in South African children aged under 5 years: the role of participant motivation	Outcome
du Preez 2011	Randomized intervention study of solar disinfection of drinking water in the prevention of dysentery in Kenyan children aged under 5 years	Population
Duffy 1997	Positive effects of an antenatal group teaching session on postnatal nipple pain, nipple trauma and breast feeding rates	Outcome
Duggan 2003	Oligofructose-supplemented infant cereal: 2 randomized, blinded, community-based trials in Peruvian infants	Population
Duijts 2010	Prolonged<br>and exclusive breastfeeding reduces the risk of infectious<br>diseases in infancy	Study design
Duncan 1993	Exclusive breast-feeding for at least 4 months protects against otitis media	Study design
Edraki M 2015	Comparison of the effects of attachment training for mothers on the behavioral responses of premature infants: A randomized clinical trial	Outcome
Eka Pratiwi 2009	Effect of kangaroo method on the risk of hypothermia and duration of birth weight regain in low birth weight infants: A randomized controlled trial	Outcome
Ekbote 2011	A pilot randomized controlled trial of oral calcium and vitamin D supplementation using fortified laddoos in underprivileged Indian toddlers	Population
Ekcharoen 2015	Comparing growth rates after hospital discharge of preterm infants fed with either post-discharge formula or high-protein, medium-chain triglyceride containing formula	Study design
El Frargy 2017	Zinc supplementation as an adjuvant treatment in neonatal sepsis	Outcome
El-Farghali 2015	Early zinc supplementation and enhanced growth of the low-birth weight neonate	Outcome
Engstrom 2008	Effectiveness of daily and weekly iron supplementation in the prevention of anemia in infants.	Other
Ercumen 2015	Effects of source- versus household contamination of tubewell water on child diarrhea in rural Bangladesh: A randomized controlled trial	Outcome
Ermis 2002	Effects of three different iron supplementations in term healthy infants after 5 months of life.	Other
Evangelista-Salazar 2004	Evaluation of the preventive effect of the intermittent provision of iron and vitamin C on the reduction of the iron and neurodevelopment in infants	Other
Evelein 2011	The association between breastfeeding and the cardiovascular system in early childhood.	Study design
Fabiansen 2017	Effectiveness of food supplements in increasing fat-free tissue accretion in children with moderate acute malnutrition: A randomised 2 x 2 x 3 factorial trial in burkina faso	Outcome
Fahmida 2007	Zinc-iron, but not zinc-alone supplementation, increased linear growth of stunted infants with low haemoglobin	Population
Fahmida 2007	Zinc-iron, but not zinc-alone supplementation, increased linear growth of stunted infants with low haemoglobin.	Other
Fallahrokni A 2017	Zinc supplementation is an effective and feasible strategy to prevent growth retardation in 6 to 24 month children: A pragmatic randomized double blind trial	Other
Faqih 2006	Effectiveness of intermittent iron treatment of two- to six-year-old Jordanian children with iron-deficiency anemia	Population
Fardig 1980	A comparison of skin-to-skin contact and radiant heaters in promoting neonatal thermoregulation	Outcome
Fawzi 1997	Dietary vitamin a intake in relation to child growth	Study design
Fayad 1999	Comparison of soy-based formulas with lactose and with sucrose in the treatment of acute diarrhea in infants	Population
Feher 1989	Increasing breast<br>milk production for premature infants with a relaxation/<br>imagery audiotape	Outcome
Ferber 2004	The effect of skin-to-skin contact (kangaroo care) shortly after birth on the neurobehavioral	Outcome

© 2019 Park JJH et al. *JAMA Network Open*.

First author, year	Title	Reason
	responses of the term newborn: a randomized, controlled trial	
Fernandeez-Rao S 2015	Multiple micronutrients and early learning interventions promote infant micronutrient status and development	Other
Fewtrell 2001a	Randomized study comparing the efficacy of a novel manual breast pump with a mini-electric breast pump in mothers of term infants	Outcome
Fewtrell 2001b	Randomized trial comparing the efficacy of a novel manual breast pump with a standard electric breast pump in mothers who delivered preterm infants	Outcome
Filteau 2011	Provision of micronutrient-fortified food from 6 months of age does not permit HIV-exposed uninfected Zambian children to catch up in growth to HIV-unexposed children: A randomized controlled trial	Population
Finch 2002	Breastfeeding education program with incentives increases exclusive breastfeeding among urban WIC participants	Outcome
Fink 2017	Home- and community-based growth monitoring to reduce early life growth faltering: An open-label, cluster-randomized controlled trial	Population
Fischer Walker 2006	Zinc supplementation for the treatment of diarrhea in infants in Pakistan, India and ethiopia	Outcome
Fischer Walker 2009	Low-dose weekly supplementation of iron and/or zinc does not affect growth among Bangladeshi infants	Population
Flaherman 2012	Randomised trial comparing hand expression with breast pumping for mothers of term newborns feeding poorly	Outcome
Flax 2013	Growth and micronutrient status of HIV-exposed infants in the breastfeeding, antiretrovirals, and nutrition study during the first year of life	Outcome
Flax 2014	Integrating group counseling, cell phone messaging, and participant-generated songs and dramas into a microcredit program increases Nigerian women's adherence to international breastfeeding recommendations	Outcome
Flax 2015	Provision of lipid-based nutrient supplements to Honduran children increases their dietary macro- and micronutrient intake without displacing other foods	Population
Forster 2004	Two mid-pregnancy interventions to increase the initiation and duration of breastfeeding: a randomized controlled trial.	Outcome
Francis 2008	Maternal/infant lactation characteristics (MILC) study: a comparison of single electric pumping devices	Outcome
Freeman 2016	The impact of a rural sanitation programme on safe disposal of child faeces: A cluster randomised trial in odisha, India	Outcome
Friedrich 2018	Enhancing handwashing frequency and technique of primary caregivers in harare, zimbabwe: A cluster-randomized controlled trial using behavioral and microbial outcomes	Outcome
Friel 1993	Zinc supplemetation in very-low-birth-weight infants	Population
Friis 1997	The impact of zinc supplementation on growth and body composition: a randomized, controlled trial among rural Zimbabwean schoolchildren	Population
Furuhjelm 2009	Fish oil supplementation in pregnancy and lactation may decrease the risk of infant allergy	Population
Garate-Gallardo 2015	Comparing different maize supplementation strategies to improve resilience and resistance against gastrointestinal nematode infections in browsing goats	Outcome
Gardner 1998	Zinc supplementation: effects on the growth and morbidity of undernourished Jamaican children	Population
Gardner 2005	Zinc supplementation and psychosocial stimulation: effects on the development of undernourished Jamaican children	Population
Gargano 2018	Efficacy and tolerability outcomes of a phase ii, randomized, open-label, multicenter study of a new water-dispersible pediatric formulation of dihydroartemisinin-piperaquine for the treatment of uncomplicated plasmodium falciparum malaria in african infants	Study design
Garmendia 2015	Effectiveness of a normative nutrition intervention (diet, physical activity and breastfeeding) on maternal nutrition and offspring growth: The chilean maternal and infant nutrition cohort study (chimincs)	Population
Garza 1982	Effects of methods of collection and storage on nutrients in human milk	Outcome
Gathwala 2008	KMC facilitates mother baby attachment in low birth weight infants.	Outcome
Gavhane 2016	Long term outcomes of kangaroo mother care in very low birth weight infants	Outcome
Ghavane 2012	Kangaroo Mother Care in Kangaroo ward for improving the growth and breastfeeding outcomes when reaching term gestational age in very low birth weight infants.	Outcome
Gibson 1989	A growth-limiting, mild zinc-deficiency syndrome in some southern Ontario boys with low height percentiles	Other
Gibson 1997A	Effect of increasing breast milk docosahexaenoic acid on plasma and erythrocyte phospholipid fatty acids and neural indices of exclusively breast-fed infants	Population
Gibson 1998	The effects of diets rich in docosahexaenoic acid and/or gamma-linolenic acid on plasma fatty acid profiles in term infants	Study design
Gibson 2009	Safety of supplementing infant formula with long-chain polyunsaturated fatty acids and Bifidobacterium lactis in term infants: a randomised controlled trial	Study design
Gill 1997	Effect of fortification of drinking water with iron plus ascorbic acid or with ascorbic acid alone on hemoglobin values and anthropometric indicators in preschool children in day-care centers in Southeast Brazil	Outcome

© 2019 Park JJH et al. *JAMA Network Open*.

First author, year	Title	Reason
Giovannini 2006	Double-blind, placebo-controlled trial comparing effects of supplementation with two different combinations of micronutrients delivered as sprinkles on growth, anemia, and iron deficiency in Cambodian infants	Population
Girish 2013	Impact and feasibility of breast crawl in a tertiary care hospital	Outcome
Glinz 2015	The effect of iron-fortified complementary food and intermittent preventive treatment of malaria on anaemia in 12- to 36-month-old children: A cluster-randomised controlled trial	Population
Glinz 2017	Iron fortified complementary foods containing a mixture of sodium iron edta with either ferrous fumarate or ferric pyrophosphate reduce iron deficiency anemia in 12- to 36-month-old children in a malaria endemic setting: A secondary analysis of a cluster-randomized controlled trial	Outcome
Goto 2009	Impact of anti-giardia and anthelminthic treatment on infant growth and intestinal permeability in rural Bangladesh: a randomised double-blind controlled study	Population
Gouchon 2010	Skin-to-skin contact after cesarean delivery: an experimental study	Outcome
Goudarzvand 2017	Comparison of conventional phototherapy and phototherapy along with kangaroo mother care on cutaneous bilirubin of neonates with physiological jaundice	Outcome
Grange 1994	Evaluation of a maize-cowpea-palm oil diet for the dietary management of Nigerian children with acute, watery diarrhea	Population
Grantham-McGregor 1993	The effect of nutritional supplementation and stunting on morbidity in young children: The jamaican study	Population
Greenland 2016	Multiple behaviour change intervention for diarrhoea control in lusaka, zambia: A cluster randomised trial	Population
Grillenberger 2003	Food supplements have a positive impact on weight gain and the addition of animal source foods increases lean body mass of Kenyan schoolchildren	Population
Groh-Wargo 1995	The utility of a bilateral breast pumping system for mothers of premature infants	Outcome
Guldan 2000	Culturally appropriate nutrition education improves infant feeding and growth in rural Sichuan, China.	Population
Gupta 1982	Effect of periodic antiascaris and antigiardia treatment on nutritional status of preschool children	Population
Gupta 2016	Vitamin d supplementation for treatment and prevention of pneumonia in under-five children: A randomized double-blind placebo controlled trial	Population
Gupta 2017	Complementary feeding at 4 versus 6 months of age for preterm infants born at less than 34 weeks of gestation: A randomised, open-label, multicentre trial	Outcome
Guzman 1958	Growth and development of Central American children: growth responses of rural Guatemalan school children to daily administration of penicillin and aureomycin	Population
Haber Ja 2016	The effect of maternal supplementation with a lipid-based nutrient supplement on infant micronutrient intake in Guatemalan women and infants	Study design
Haber Ja 2016A	The short-term response of breast milk micronutrient concentrations to a lipid-based nutrient supplement in Guatemalan women	Outcome
Hadi 1999	Respiratory infections reduce the growth response to vitamin a supplementation in a randomized controlled trial	Outcome
Hadi 2000	Vitamin a supplementation selectively improves the linear growth of Indonesian preschool children: Results from a randomized controlled trial	Outcome
Hadi 2004	Complex interactions with infection and diet may explain seasonal growth responses to vitamin a in preschool aged Indonesian children	Outcome
Hales 1977	Defining the limits of the maternal sensitive period	Outcome
Hall 2002	A randomised trial in Mali of the effectiveness of weekly iron supplements given by teachers on the haemoglobin concentrations of schoolchildren	Population
Hall 2007	An evaluation of the impact of a school nutrition programme in Vietnam	Study design
Harding 2017	Adherence to recommendations on lipid-based nutrient supplement and iron and folic acid tablet consumption among pregnant and lactating women participating in a community health programme in northwest Bangladesh	Outcome
Hartinger 2016	Improving household air, drinking water and hygiene in rural Peru: A community-randomized-controlled trial of an integrated environmental home-based intervention package to improve child health	Outcome
Hartinger Sm 2012	A community randomized controlled trial of an integrated home-based intervention improving household-air pollution, drinking water quality and hygiene in rural Peru	Population
Haschke 1988	Iron intake and iron nutritional status of infants fed	Population
Hauner 2009	The impact of nutritional fatty acids during pregnancy and lactation on early human adipose tissue development	Population
Heikens 1989	The Kingston project. I. Growth of malnourished children during rehabilitation in the community, given a high energy supplement	Other
Heikens 1993	The Kingston project. Ii. The effects of high energy supplement and metronidazole on malnourished children rehabilitated in the community: Anthropometry	Other
Heinig 1993	Intake and growth of breast-fed and formula- fed infants in relation to the timing of introduction of complementary foods: the DARLING study	Study design

© 2019 Park JJH et al. *JAMA Network Open*.

First author, year	Title	Reason
Heinig 2006	Zinc supplementation does not affect growth, morbidity, or motor development of US term breastfed infants at 4–10 mo of age	Outcome
Helland 2001	Similar effects on infants of n-3 and n-6 fatty acids supplementation to pregnant and lactating women	Population
Helmizar 2017	Local food supplementation and psychosocial stimulation improve linear growth and cognitive development among Indonesian infants aged 6 to 9 months	Population
Hemsworth 2016	Lipid-based nutrient supplements increase energy and macronutrient intakes from complementary food among Malawian infants	Outcome
Heon 2011	Nursing Interventions Related to Breastfeeding in Preterm Infants	Outcome
Hess 2015	Iodine status of young burkinabe children receiving small-quantity lipid-based nutrient supplements and iodised salt: A cluster-randomised trial	Outcome
Hess 2015a	Small-Quantity Lipid-Based Nutrient Supplements, Regardless of Their Zinc Content, Increase Growth and Reduce the Prevalence of Stunting and Wasting in Young Burkinabe Children: A Cluster-Randomized Trial	Population
Hettiarachchi 2008	The efficacy of micronutrient supplementation in reducing the prevalence of anaemia and deficiencies of zinc and iron among adolescents in Sri Lanka	Outcome
Hill 1999	Breastfeeding experience and milk weight in lactating mothers pumping for preterm infants	Outcome
Hollis 2004	Vitamin D requirements during lac- tation: high-dose maternal supplementation as therapy to prevent hypovitaminosis D for both the mother and the nurs-<br>ing infant	Other
Holscher 2012	Bifidobacterium lactis Bb12 Enhances Intestinal Antibody Response in Formula-Fed Infants: A Randomized, Double-Blind, Controlled Trial	Population
Hong 1992	Growth promoting effect of zinc supplementation in infants of high-risk pregnancies.	Other
Hopkinson 2009	Maternal response to two electric breast pumps	Outcome
Hossain 2005	Increased food intake after the addition of amylase-rich flour to supplementary food for malnourished children in rural communities of Bangladesh	Outcome
Hossain Mdi 2016	Home-based care with follow-up at outpatient unit or community-follow-up center with/without food supplementation and/or psychosocial stimulation of children with moderate acute malnutrition in Bangladesh	Outcome
Huang 2006	Effect of very early kangaroo care on extrauterine temperature adaptation in newborn infants with hypothermia problem	Outcome
Huey S 2017	Pre-intervention characterization of nutritional status to estimate burden and potential to benefit among mothers and their children living in urban slums of Mumbai, India	Study design
Huffman 1987	Nutrition and fertility in Bangladesh: breastfeeding and post partum amenorrhea	Study design
Husaini 1991	Developmental effects of short-term supplementary feeding in nutritionally-at-risk Indonesian infants.	Population
Husaini 1996	The effects of high energy and micronutrient supplementation on iron status in nutritionally at risk infants	Other
Huybregts 2012	The effect of adding ready-to-use supplementary food to a general food distribution on child nutritional status and morbidity: A cluster-randomized controlled trial	Population
Huybregts 2017	The impact of integrated prevention and treatment on child malnutrition and health: The promis project, a randomized control trial in burkina faso and mali	Outcome
Huynh Dtt 2017	Maternal nutritional adequacy and gestational weight gain in vietnamese pregnant women	Other
Hyder 2007	A multiple-micronutrient- fortified beverage affects hemoglobin, iron, and vitamin A status and growth in adolescent girls in rural Bangladesh	Outcome
Iannotti 2014	Linear growth increased in young children in an urban slum of Haiti: a randomized controlled trial of a lipid-based nutrient supplement	Population
Iannotti 2015	Early growth velocities and weight gain plasticity improve linear growth in Peruvian infants	Outcome
Iannotti 2017	Eggs early in complementary feeding increase choline pathway biomarkers and dha: A randomized controlled trial in ecuador	Outcome
Idjradinata 1993	Reversal of developmental delays in iron-deficient anaemic infants treated with iron.	Population
Idjradinata 1994	Adverse effect of iron supplementation on weight gain of iron-replete young children	Population
Ihab A 2014	The Impact of Animal Source food (ASF) on the Growth of Malnourished Children in Bachok, Kelantan: Randomized Controlled Intervention Trial	Outcome
Ijumba 2015	Effect of an integrated community-based package for maternal and newborn care on feeding patterns during the first 12 weeks of life: A cluster-randomized trial in a south african township	Outcome
Inayati 2012	Combined intensive nutrition education and micronutrient powder supplementation improved nutritional status of mildly wasted children on nias island, Indonesia	Population
Innis 1996b	Blood lipid docosahexaenoic and arachidonic acid in term gestation infants fed formulas with high docosa- hexaenoic acid, low eicosapentaenoic acid fish oil	Population
Innis 1997	Visual acuity and blood lipids in term infants fed human milk or formulae	Population
Islam 2018	Study protocol for a randomized, double-blind, community-based efficacy trial of various doses of zinc in micronutrient powders or tablets in young Bangladeshi children	Other
Islam Khan 2013	Effects of pre- and postnatal nutrition interventions on child growth and body composition: The minimat trial in rural Bangladesh	Outcome
Iuliano-Burns 2003	Regional specificity of exercise and calcium during skeletal growth in girls: a randomized	Other

© 2019 Park JJH et al. *JAMA Network Open*.

First author, year	Title	Reason
	controlled trial	
Jack 2012	Effect of micronutrient Sprinkles on reducing anemia: a cluster-randomized effectiveness trial.	Population
Jaeggi 2015	Iron fortification adversely affects the gut microbiome, increases pathogen abundance and induces intestinal inflammation in Kenyan infants	Outcome
Jakobsen 2008	Promotion of exclusive breastfeeding is not likely to be cost effective in West Africa. A randomized intervention study from Guinea-Bissau	Outcome
Jannat Kke 2015	Observed handwashing behavior during infant feeding, intervention assessment of a large randomized controlled trial (rct) in rural Bangladesh	Other
Jayamala 2015	Impact of music therapy on breast milk secretion in mothers of premature newborns	Outcome
Jensen 1997	Effect of dietary linoleic/alpha-linolenic acid ratio on growth and visual function of term infants	Population
Jensen 1999	Effects of maternal docosahexaenoic acid supplementation on visual function and growth of breast-fed term infants.	Population
Jensen 2005	Effects of maternal docosahexaenoic acid intake on visual function and neurodevelopment in breastfed term infants	Population
Johnson W 2016	Pre-pregnancy energy balance, gestational weight gain, and small-for-gestational age in rural Gambia: The early nutrition and immune development trial (isrctn49285450)	Outcome
Jones 2001	A randomised<br>controlled trial to compare methods of milk expression after preterm delivery	Outcome
Jones 2015	Ready-to-use therapeutic food with elevated n-3 polyunsaturated fatty acid content, with or without fish oil, to treat severe acute malnutrition: A randomized controlled trial	Population
Jordan I 2015	Food based nutrition education improved complementary feeding practices and nutritional status of children below 2 years in Malawi	Outcome
Jorgensen 1998	Effect of formula supplemented with docosahexaenoic acid and gamma- linolenic acid on fatty acid status and visual acuity in term infants. J. Pediatr.	Outcome
Jorgensen 2017	Lipid-based nutrient supplements during pregnancy and lactation did not affect human milk oligosaccharides and bioactive proteins in a randomized trial	Outcome
Joseph 2015	The effect of deworming on early childhood development in Peru: A randomized controlled trial	Population
Joseph 2015A	The effect of deworming on growth in one-year-old children living in a soil-transmitted helminth-endemic area of Peru: A randomized controlled trial	Population
Joseph 2016	Adverse events from a randomized, multi-arm, placebo-controlled trial of mebendazole in children 12-24 months of age	Outcome
Joseph Sa 2013	Malnutrition and the critical growth window in an sthendemic area of Peru: Worm infections a barrier to achieving child-related mdgs?	Other
Juliana K 2017	Bioequivalence of micronutrient powders to conventional fortification on serum zinc levels of moderately malnourished children in thika informal settlements, Kenya	Outcome
Kabir 1998	Increased height gain of children fed a high-protein diet during convalescence from shigellosis: a six-month follow-up study	Outcome
Kadam 2005	Feasibility of Kangaroo Mother Care in Mumbai	Outcome
Kaestel 2005	Effects of prenatal multimicronutrient supplements on birth weight and perinatal mortality: a randomised, controlled trial in Guinea-Bissau	Outcome
Kajosaari 1983	Atopy prevention in childhood: the role<br>of diet: prospective 5-year follow-up of high-risk infants with six months exclusive breastfeeding and solid food elimination	Study design
Kamm 2016	Is pregnancy a teachable moment to promote handwashing with soap among primiparous women in rural Bangladesh? Follow-up of a randomised controlled trial	Outcome
Kang Y 2015	Effectiveness of a communitybased participatory nutrition promotion program to improve linear and ponderal growth in children 6 to 24 months of age in rural eastern ethiopia: A cluster randomized trial	Other
Kaplowitz 1983	The effect of an educational program on the decision to breastfeed	Outcome
Karanja 2010	The TOTS community intervention to prevent 117.<br>overweight in American Indian toddlers: a feasibility and<br>efficacy study	Population
Karimi A 2015	The effect of an interventional program based on the theory of ethology on infant breastfeeding competence	Outcome
Kaseb 2013	Efficacy of zinc supplementation on improvement of weight and height growth of healthy 9–18 year children in Iran	Population
Kastner 2005	Mother-child relationship before, during and after birth	Outcome
Keikhaei 2007	Iron-deficiency anemia among children in southwest iran	Study design
Keith 2012	The effect of music- based listening interventions on the volume, fat content, and caloric content of breast milk-produced by mothers of premature and critically ill infants	Outcome
Kellams 2016	The impact of a prenatal education video on rates of breastfeeding initiation and exclusivity during the newborn hospital stay in a low-income population	Outcome
Khademloo 2009	Comparison of the effectiveness of weekly and daily iron supplementation in 6- to 24-months-old babies in urban health centers of Sari, Iran.	Outcome

© 2019 Park JJH et al. *JAMA Network Open*.

First author, year	Title	Reason
Khadivzadeh 2004	Effect of exclusive breastfeeding and complementary feeding on infant growth and morbidity	Study design
Khadivzadeh 2009	The effect of mother- infant skin to skin contact on mother's attachment.	Outcome
Khan 1984	Breastfeeding, growth and diarrhoea in rural Bangladesh children	Population
Khan 2013	Effects of exclusive breastfeeding intervention on child growth and body composition: the MINIMat trial, Bangladesh	Population
Kikafunda 1998	Effect of zinc sup- plementation on growth and body composition of Ugandan preschool children: a randomized, controlled, intervention trial	Population
Kim S 2017A	Exposure to Large-Scale Social and Behavior Change Communication Interventions Is Associated with Improvements in Infant and Young Child Feeding Practices in Ethiopia	Other
Kim S 2017B	Two-year sustained impacts of largescale social and behavior change communication interventions to improve infant and young child feeding in Bangladesh	Outcome
Kimani-Murage 2017	Effectiveness of home-based nutritional counselling and support on exclusive breastfeeding in urban poor settings in Nairobi: A cluster randomized controlled trial	Outcome
Kirkwood 1996	Effect of vitamin a supplementation on the growth of young children in northern Ghana	Outcome
Kistin 1990	Breast-feeding rates among black urban low-income women: effect of prenatal education	Outcome
Klevor 2016	Lipid-based nutrient supplements providing approximately the recommended daily intake of vitamin a do not increase breast milk retinol concentrations among Ghanaian women	Outcome
Kluka 2004	A Randomized Controlled Trial to Test the Effect of an Antenatal Educational Intervention on Breastfeeding Duration Among Primiparous Women [thesis]	Outcome
Konyole S 2017	Effect on lean mass, linear growth and iron status of improved animal source foods and micronutrients fortified complementary foods among Kenyan young children: A randomized controlled trial	Other
Koo ww 2006	Posthospital discharge feeding for preterm infants: effects of standard compared with enriched milk formula on growth, bone mass, and body composition	Population
Kools 2005	A breast-feeding promotion and support program a randomized trial in The Netherlands	Outcome
Kordas 2009	The effects of iron and/or zinc supplementation on maternal reports of sleep in infants from nepal and zanzibar	Outcome
Kounnvaong 2011	Effect of daily versus weekly home fortification with multiple micronutrient powder on haemoglobin concentration of young children in a rural area, Lao People's Democratic Republic: a randomised trial.	Outcome
Kramer 2000	Short and Long Term Effects of Breast Feeding on Child Health	Outcome
Kramer 2007	Effects of prolonged and exclusive breastfeeding on child height, weight, adiposity, and blood pressure at age 6.5 y: evidence from a large randomized trial	Outcome
Krebs 2011	Complementary feeding: A global network cluster randomized controlled trial	Other
Krebs 2012	Randomized controlled trial of meat compared with multimicronutrient-fortified cereal in infants and toddlers with high stunting rates in diverse settings	Population
Kronborg 2012	Antenatal training to improve breast feeding: a randomised trial	Outcome
Kumar 2017	Supplementation of vitamin b12 or folic acid on hemoglobin concentration in children 6-36 months of age: A randomized placebo controlled trial	Outcome
Kumar T 2016	Supplementation of vitamin b12 or folic acid on hemoglobin concentration in children 6-36 months of age: A randomized placebo controlled trial	Outcome
Kupka 2013	Multivitamin supplements have no effect on growth of Tanzanian children born to HIV-infected mothers	Population
Kuusipalo 2006	Growth and change in blood haemoglobin concentration among underweight Malawian infants receiving fortified spreads for 12 weeks: a preliminary trial	Population
Kvestad 2015	Vitamin b12 and folic acid improve gross motor and problem-solving skills in young north Indian children: A randomized placebo-controlled trial	Outcome
Lagrone 2010	Locally produced ready-to-use supplementary food is an effective treatment of moderate acute malnutrition in an operational setting	Population
LaGrone 2012	A novel fortified blended flour, corn-soy blend "plus-plus," is not inferior to lipid-based ready-to-use supplementary foods for the treatment of moderate acute malnutrition in Malawian children	Outcome
Lampl 1978	The effects of protein supple- mentation on the growth and skeletal maturation of new Guinean school children	Population
Lamy Filho 2015	Effect of maternal skin-to-skin contact on decolonization of methicillin-oxacillin-resistant staphylococcus in neonatal intensive care units: A randomized controlled trial	Outcome
Langlois B 2017	Changes in household food insecurity between enrollment and exit from a blanket supplementary feeding program for children 6 - 23 months old in burkina faso	Other
Larnkjær 2014	Effect of milk proteins on linear growth and IGF variables in overweight adolescents	Outcome
Larson 2010	The added benefit of zinc supplementation after zinc treatment of acute childhood diarrhoea: a randomized, double-blind field trial	Outcome
Larson L 2017	Relation between diet, nutritional status, and stimulation and child development: A path analysis	Study design
Lartey 1999	A randomized, community-based trial of the effects of improved, centrally processed complementary foods on growth and micronutrient status of Ghanaian infants from 6 to 12 mo of age	Population

© 2019 Park JJH et al. *JAMA Network Open*.

First author, year	Title	Reason
Latham 1990	Improvements in growth following iron supplementation in young Kenyan school children	Other
Lauritzen 2004	Maternal fish oil supplementation in lactation: effect on visual acuity and n- 3 fatty acid content of infant erythrocytes	Outcome
Lavender 2005	Breastfeeding expectations versus reality: a cluster randomised controlled trial	Outcome
Lawless 1994	Iron supplementation improves appetite and growth in anemic Kenyan primary school children	Population
Lazarus 2018	The effect of probiotics and zinc supplementation on the immune response to oral rotavirus vaccine: A randomized, factorial design, placebo-controlled study among Indian infants	Outcome
Leroy 2016	Tubaramure, a food-assisted integrated health and nutrition program in burundi, increases maternal and child hemoglobin concentrations and reduces anemia: A theory-based cluster-randomized controlled intervention trial	Outcome
Li 2009	Effects of maternal multimicronutrient supplementation on the mental development of infants in rural western China: follow-up evaluation of a double-blind, randomized, controlled trial	Population
Li 2015	Prenatal micronutrient supplementation is not associated with intellectual development of young school-aged children	Population
Lima 2010	Effects of vitamin a supplementation on intestinal barrier function, growth, total parasitic, and specific giardia spp infections in brazilian children: A prospective randomized, double-blind, placebo-controlled trial	Population
Lima 2014	Effects of glutamine alone or in combination with zinc and vitamin a on growth, intestinal barrier function, stress and satiety-related hormones in brazilian shantytown children	Population
Lin 2008	An energy-dense complementary food is associated with a modest increase in weight gain when compared with a fortified porridge in Malawian children aged 6-18 months	Population
Lin 2009	Effect of beta-carotene supplementation on health and growth of vitamin a deficient children in china rural villages: A randomized controlled trial	Outcome
Lind 2004	A community-based randomized controlled trial of iron and zinc supplementation in Indonesian infants: effects on growth and development	Population
Lira 1998	Effect of zinc supplementation on the morbidity, immune function, and growth of low-birth-weight, full-term infants in northeast Brazil.	Other
Lira 2017	Effect of rrr-alpha-tocopherol supplementation on serum of breastfeeding women up to 60 days after delivery: A randomised controlled trial	Outcome
Liu 1995	Intermittent iron supplementation in Chinese pre-schoolchildren is efficient and safe	Population
Liu 2016	Reproductive health care and family planning among women in Nepal	Outcome
Lloyd 1993	Calcium supplementation and bone mineral density in adolescent girls	Outcome
Locks 2017	The effect of daily zinc and/or multivitamin supplements on early childhood development in Tanzania: Results from a randomized controlled trial	Outcome
Long 2006A	A double-blind, randomized, clinical trial of the effect of vitamin A and zinc supplementation on diarrheal disease and respiratory tract infections in children in Mexico City, Mexico	Outcome
Long 2007	Supplementation with vitamin A reduces watery diarrhoea and respiratory infections in Mexican children.	Outcome
Long 2011	Vitamin a supplementation modifies the association between mucosal innate and adaptive immune responses and resolution of enteric pathogen infections	Outcome
Lopriore 2004	Spread fortified with vita- mins and minerals induces catch-up growth and eradicates severe anemia in stunted refugee children aged 3–6 y	Population
Loui 2004	Nutrition of Very Low Birth Weight Infants Fed Human Milk with or without Supplemental Trace Elements: A Randomized Controlled Trial	Outcome
Louzada 2012	Long- term effectiveness of maternal dietary counseling in a low- income population: a randomized field trial	Outcome
Luby 2004	Effect of intensive handwashing promotion on childhood diarrhoea in high-risk communities in Pakistan: a randomised controlled trial	Outcome
Luby 2006	Combining drinking water treatment and hand washing for diarrhoea prevention, a cluster randomised controlled trial	Outcome
Luby 2018	Effects of water quality, sanitation, handwashing, and nutritional interventions on diarrhoea and child growth in rural Bangladesh: A cluster randomised controlled trial	Outcome
Lucas 1996	Randomized outcome trial of human milk fortification and developmental outcome in preterm infants	Population
Lucas 1999	Efficacy and safety of long- chain polyunsaturated fatty acid supplementation of infant-formula milk: A randomised trial	Population
Lucia 2007	Does maternal docosahexaenoic acid supplementation during pregnancy and lactation lower BMI in late infancy?	Population
Lumbanraja 2016	Influence of maternal factors on growth parameters in low-birth-weight babies with kangaroo mother care	Other
Lundeen 2010	Daily use of Sprinkles micronutrient powder for 2 months reduces anemia among children 6 to 36 months of age in the Kyrgyz Republic: a cluster- randomized trial	Outcome
Luong 2015	Newly born low birthweight infants stabilise better	Outcome

© 2019 Park JJH et al. *JAMA Network Open*.

First author, year	Title	Reason
	in skin-to-skin contact than when separated from their mothers: a randomised controlled trial	
Luoto 2014	Prebiotic and probiotic supplementation prevents rhinovirus infections in preterm infants: A randomized, placebo-controlled trial	Outcome
Lussier 2015	Daily breastmilk volume in mothers of very low birth weight neonates: A repeated-measures randomized trial of hand expression versus electric breast pump expression	Outcome
Lutter 1989	Nutritional supplementation: Effects on child stunting because of diarrhea	Outcome
Ly 2006	Early shortterm infant food supplementation, maternal weight loss and duration of breast-feeding: a randomised controlled trial in rural Senegal.	Outcome
Ma 2016	The effect of iron fortification on iron (fe) status and inflammation: A randomized controlled trial	Study design
MacArthur 2009	Antenatal peer support workers and initiation of breast feeding: cluster randomised controlled trial	Outcome
Macharia-Mutie 2012	Maize porridge enriched with a micronutrient powder containing low-dose iron as NaFeEDTA but not amaranth grain flour reduces anemia and iron deficiency in Kenyan preschool children	Outcome
Mahdavi 2015	A pilot study of symbiotic supplementation on breast milk mineral concentrations and growth of exclusively breast fed infants	Outcome
Mahmood 2011	Effect of mother-infant early skin-to-skin contact on breastfeeding status: A randomized controlled trial	Other
Makrides 1000b	A critical appraisal of the role of dietary long-chain polyunsaturated fatty acids on neural indices of term infants: A randomized, controlled trial.	Intervention
Makrides 1995	Are long-chain polyunsaturated fatty acids essential nutrients in infancy?	Population
Makrides 1999	Dietary long-chain polyunsaturated fatty acids do not influence growth of term infants: A randomized clinical trial	Population
Makrides 2000a	A randomized trial of different ratios of linoleic to alpha-linolenic acid in the diet of term infants: Effects on visual function and growth	Other
Malcolm 1970	Growth retardation in a new Guinea boarding school and its response to supplementary feeding	Population
Maleta 2004	Supplementary feeding of underweight, stunted Malawian children with a ready-to-use food	Population
Maleta 2015	Provision of 10-40 g/d Lipid-Based Nutrient Supplements from 6 to 18 Months of Age Does Not Prevent Linear Growth Faltering in Malawi.	Population
Maleta K 2013	Efficacy of reduced-cost lns formulations for infants and young children in Malawi: The ilins-dose trial	Other
Malik 2013	Short-course prophylactic zinc supplementation for diarrhea morbidity in infants of 6 to 11 months	Outcome
Mamiro 2004	Processed complementary food does not improve growth or hemoglobin status of rural tanzanian infants from 6-12 months of age in kilosa district, tanzania	Population
Mangani 2013	Effect of complementary feeding with lipid-based nutrient supplements and corn-soy blend on the incidence of stunting and linear growth among 6- to 18-month-old infants and children in rural Malawi	Population
Mangani 2014	Providing lipid-based nutrient supplements does not affect developmental milestones among Malawian children	Population
Mangani 2015	Effect of complementary feeding with lipid-based nutrient supplements and corn-soy blend on the incidence of stunting and linear growth among 6- to 18-month-old infants and children in rural Malawi	Population
Mangel 2015	Higher fat content in breastmilk expressed manually: a randomized trial	Outcome
Manger 2008	A micronutrient-fortified seasoning powder reduces morbidity and im- proves short-term cognitive function, but has no effect on anthropo- metric measures in primary school children in northeast Thailand: a randomized controlled trial	Population
Manno 2011	Rich micronutrient fortification of locally produced infant food does not improve mental and motor development of Zambian infants: a randomised controlled trial	Outcome
Mardones 2007	Effects of a dairy product fortified with multiple micronutrients and omega-3 fatty acids on birth weight and gestation duration in pregnant Chilean women	Outcome
Marin 2010	Randomized controlled trial of early skin-to- skin contact: effects on the mother and the newborn	Outcome
Marquis G 2017	Putting our eggs in more than one basket-lessons learned from working with multiple sectors in rural Ghana	Other
Marsh 1959	Comparative Hematologic Response to Iron Fortification of a Milk Formula for Infants	Outcome
Martinez-Estevez 2016	Effects of zinc supplementation in the prevention of respiratory tract infections and diarrheal disease in Colombian children: A 12-month randomised controlled trial	Outcome
Masanja 2015	Effect of neonatal vitamin a supplementation on mortality in infants in Tanzania (neovita): A randomised, double-blind, placebo-controlled trial	Outcome
Mathur 2015	Zinc supplementation in preterm neonates and neurological development, a randomized controlled trial	Outcome
Mathur 2016	Assessment of adequacy of supplementation of vitamin d in very low birth weight preterm	Intervention

© 2019 Park JJH et al. *JAMA Network Open*.

First author, year	Title	Reason
	neonates: A randomized controlled trial	
Matias 2017	Home fortification during the first 1000 d improves child development in Bangladesh: A cluster-randomized effectiveness trial	Outcome
Matias S 2017	Effects of lipid-based nutrient supplements v. micronutrient powders on nutritional and developmental outcomes among Peruvian infants	Population
Matos 2016	Prevention and treatment of anemia in infants through supplementation, assessing the effectiveness of using iron once or twice weekly	Outcome
Mattar 2007	Simple antenatal preparation to improve breastfeeding practice: a randomized controlled trial	Outcome
Maulen-Radovan 1994	Comparison of a rice-based, mixed diet versus a lactose-free, soy-protein isolate formula for young children with acute diarrhea	Population
Maurage 1998	Effect of two types of fish oil supplementation on plasma and erythrocyte phospholipids in formula-fed term infants.	Population
Mausezahl D 2015	Integrating environmental home-based interventions to improve the quality of household air, drinking water and hygiene in rural Peru: Community-randomised controlled trial	Outcome
Mazariegos 2010	Neither a zinc supplement nor phytate-reduced maize nor their combination enhance growth of 6- to 12-month-old Guatemalan infants	Population
Mazumder 2014	Effect of implementation of Integrated Management of Neonatal and Childhood illness programme on treatment seeking practices for morbidities in infants: cluster randomised trial	Outcome
Mazumder 2015	Efficacy of early neonatal supplementation with vitamin a to reduce mortality in infancy in haryana, India (neovita): A randomised, double-blind, placebo-controlled trial	Outcome
Mazurek 1999	Influence of immediate newborn care on infant adaptation to the environment	Outcome
McClellan 1980	Effects of early mother- infant contact following cesarean birth	Outcome
McDonald 2015	Daily zinc but not multivitamin supplementation reduces diarrhea and upper respiratory infections in Tanzanian infants: A randomized, double-blind, placebo-controlled clinical trial	Outcome
McGuigan 2011	A high compliance randomised controlled field trial of solar disinfection (SODIS) of drinking water and its impact on childhood diarrhoea in rural Cambodia.	Outcome
Mda 2013	Impact of multi-micronutrient supplementation on growth and morbidity of HIV-infected south African children	Population
Mebrahtu 2004	Low-dose daily iron supplementation for 12 months does not increase the prevalence of malarial infection or density of parasites in young Zanzibari children	Outcome
Medeiros 2015	The effect of folic acid supplementation with ferrous sulfate on the linear and ponderal growth of children aged 6-24 months: A randomized controlled trial	Population
Medoua 2016	Recovery rate of children with moderate acute malnutrition treated with ready-to-use supplementary food (rusf) or improved corn-soya blend (csb+): A randomized controlled trial	Population
Mehta 2011	A randomized trial of multivitamin supplementation in children with tuberculosis in tanzania	Outcome
Meier 2008	A comparison of the efficiency, efficacy, comfort, and convenience of two hospital-grade electric breast pumps for mothers of very low birthweight infants	Outcome
Meier 2012	Breast<br>pump suction patterns that mimic the human infant<br>during breastfeeding: greater milk output in less time<br>spent pumping for breast pump-dependent mothers with premature infants	Outcome
Meinzen-Derr 2006	Risk of infant anemia is associated with exclusive breast-feeding and maternal anemia in a Mexican cohort.	Outcome
Menon 2007	Micronutrient Sprinkles reduce anemia among 9- to 24-mo-old children when delivered through an integrated health and nutrition program in rural Haiti.	Outcome
Menon 2016	Impacts on breastfeeding practices of at-scale strategies that combine intensive interpersonal counseling, mass media, and community mobilization: Results of cluster-randomized program evaluations in Bangladesh and viet nam	Outcome
Mersmann 1993	Therapeutic Touch and Milk Letdown in Mothers of Non-nursing Preterm Infants [PhD Thesis]	Outcome
Mirnia 2017	Paternal skin-to-skin care and its effect on cortisol levels of the infants	Outcome
Mizuno 2004	Mother- infant skin-to-skin contact after delivery results in early recognition of own mother's milk odour.	Other
Mofd Ls 2016	The effect of maternal postpartum deworming on infection status, anemia and fatigue	Outcome
Mohammed H 2015	A cluster rct evaluating the effect of iodized salt on infant development in amhara region of ethiopia	Outcome
Mojibian 2015	The effects of vitamin d supplementation on maternal and neonatal outcome: A randomized clinical trial	Outcome
Moodley 2015	Single high-dose vitamin d at birth corrects vitamin d deficiency in infants in mexico	Outcome
Moore 2005	Randomized controlled trial or early mother-infant skin-to-skin contact and breastfeeding success	Other
Morley 1999	Iron fortified follow on formula from 9 to 18 months improves iron status but not	Outcome

© 2019 Park JJH et al. *JAMA Network Open*.

First author, year	Title	Reason
	development or growth: a randomised trial	
Mozaffari-Khosravi 2009	Effects of zinc supplementation on physical growth in 2–5-year-old children	Population
Muhangi 2013	Maternal HIV infection and other factors associated with growth outcomes of HIV-uninfected infants in entebbe, Uganda	Study design
Muhihi 2016	Risk factors for small-for-gestational-age and preterm births among 19,269 tanzanian newborns	Outcome
Muhoozi 2017	Nutrition, hygiene, and stimulation education to improve growth, cognitive, language, and motor development among infants in Uganda: A cluster-randomized trial	Population
Mullen 2012	The effects of micronutrient-fortified complementary/replacement food on intestinal permeability and systemic markers of inflammation among maternally HIV-exposed and unexposed Zambian infants	Outcome
Muller 2001	Effect of zinc supplementation on malaria and other causes of morbidity in west African children: randomised double blind placebo controlled trial	Outcome
Muller 2003	Effect of zinc supplementation on growth in West African children: a randomized double-blind placebo-controlled trial in rural Burkina Faso	Outcome
Mustila 2013	Pragmatic controlled trial to prevent childhood obesity in maternity and child health care clinics: pregnancy and infant weight outcomes (The VACOPP Study)	Study design
Muthayya 2009	Effect of fortification with multiple micronutrients and n23 fatty acids on growth and cognitive performance in Indian schoolchildren: the CHAMPION (Children's Health and Mental Performance Influenced by Optimal Nutrition) study	Population
Mwanri 2000	Supplemental vitamin A im- proves anemia and growth in anemic school children in Tanzania	Population
Nackers 2010	Effectiveness of ready-to-use therapeutic food compared to a corn/soy-blend-based pre-mix for the treatment of childhood moderate acute malnutrition in Niger	Population
Nagai 2010	Earlier versus later continuous Kangaroo Mother Care (KMC) for stable low-birth-weight infants: a randomized controlled trial	Outcome
Nahar 2015	Effect of a food supplementation and psychosocial stimulation trial for severely malnourished children on the level of maternal depressive symptoms in Bangladesh	Outcome
Naheed 2009	Zinc therapy for diarrhoea improves growth among Bangladeshi infants 6 to 11 months of age	Outcome
Nahidi 2011	Effect of early skin- to- skin contact of mother and newborn on mother's satisfaction.	Other
Nakamura 1993	Mild to moderate zinc deficiency in short children: effect of zinc supplementation on linear growth velocity	Other
Namara 2017	Effects of treating helminths during pregnancy and early childhood on risk of allergy-related outcomes: Follow-up of a randomized controlled trial	Outcome
Namaste S 2017	Comparing the effectiveness and cost-effectiveness of facility-versus community-based distribution of micronutrient powders in rural Uganda	Outcome
Nasehi 2012	The effect of early breastfeeding after cesarean section on the success of exclusive breastfeeding	Other
Navarro 2013	The double task of preventing malnutrition and overweight: a quasi-experimental community-based trial	Study design
Nazeri 2017	The effects of iodine fortified milk on the iodine status of lactating mothers and infants in an area with a successful salt iodization program: A randomized controlled trial	Outcome
Ndeezi 2010	Effect of multiple micronutrient supplementation on survival of HIV-infected children in Uganda: A randomized, controlled trial	Population
Ndibazza 2012	Impact of anthelminthic treatment in pregnancy and childhood on immunisations, infections and eczema in childhood: a randomised controlled trial.	Outcome
Neu 2010	Maternal Holding of Preterm Infants During the Early Weeks After Birth and Dyad Interaction at Six Months	Study design
Newton 2016	Vitamin a status and body pool size of infants before and after consuming fortified home-based complementary foods	Population
Nguyen 2002	Efficacy of daily and weekly iron supplementation for the control of iron deficiency anaemia in infants in rural Vietnam	Outcome
Nguyen 2017	Integrating nutrition interventions into an existing maternal, neonatal, and child health program increased maternal dietary diversity, micronutrient intake, and exclusive breastfeeding practices in Bangladesh: Results of a cluster-randomized program evaluation	Outcome
Nguyen P 2016	Influences of early child nutritional status and home environment on child development in Vietnam	Outcome
Nguyen P 2017	Feasibility and impacts of integrating nutrition interventions into an existing maternal, neonatal, and child health platform in Bangladesh	Outcome
Nimbalkar 2014A	Effect of early skin-to-skin contact following normal delivery on incidence of hypothermia in neonates more than 1800 g: randomized control trial	Study design
Nimbalkar S 2016	Immediate skin to skin placement of the newborn on mothers abdomen stabilizes heart rate and rapidly normalizes oxygen saturation: A randomized controlled trial	Outcome

© 2019 Park JJH et al. *JAMA Network Open*.

First author, year	Title	Reason
Ninh 1996	Zinc supplementation increases growth and circulating insulin-like growth factor I (IGF-I) in growth-retarded Vietnamese children	Outcome
Noel-Weiss 2006	Randomized controlled trial to determine effects of prenatal breastfeeding workshop on maternal breastfeeding self- efficacy and breastfeeding duration	Outcome
Nogueria (22) 2012	Use of Iron-Fortified Rice Reduces Anemia in Infants	Outcome
Nolan 2009	A pilot study of a nursing intervention protocol to minimize maternal-infant separation after Cesarean birth.	Outcome
Nopchinda 2002	Effect of bifidobacterium bb12 with or without streptococcus thermophilus supplemented formula on nutritional status	Other
Norouzi 2013	The impact of kangaroo care and music on maternal state anxiety	Other
Nowson 1997	A co-twin study of the effect of calcium supplementation on bone density during adolescence	Outcome
Null 2018	Effects of water quality, sanitation, handwashing, and nutritional interventions on diarrhoea and child growth in rural Kenya: A cluster-randomised controlled trial	Population
Nurko 1998	Successful use of a chicken-based diet for the treatment of severely malnourished children with persistent diarrhea: A prospective, randomized study	Population
Oaks 2017	Effects of a lipid-based nutrient supplement during pregnancy and lactation on maternal plasma fatty acid status and lipid profile: Results of two randomized controlled trials	Outcome
Obatolu 2003	Growth pattern of infants fed with a mixture of extruded malted maize and cowpea	Population
Ocansey Me 2017	Pre-and post-natal lipid-based nutrient supplements and cognitive, socioemotional and motor function in preschool-aged children in Ghana	Population
Oddy 1999	Association between breast feeding and asthma in 6 year old children: findings of a prospective birth cohort study.	Outcome
Oelofse 2003	The effect of a micronutrientfortified complementary food on micronutrient status, growth and development of 6- to 12-month-old disadvantaged urban South African infants	Population
Okronipa 2018	Maternal supplementation with small-quantity lipid-based nutrient supplements during pregnancy and lactation does not reduce depressive symptoms at 6 months postpartum in Ghanaian women: A randomized controlled trial	Outcome
Okronipa H 2017B	Impact of exposure to lipid-based nutrient supplements in early life on sweet taste preference of Ghanaian children aged 4-6 years: A non-inferiority study	Population
Okronipa Het 2016	The impact of lipid-based nutrient supplements on maternal depression at 6 mo postpartum in Ghana: A randomized-controlled trial	Outcome
Olenick 2010	The effect of structured group prenatal education on breastfeeding confidence, duration, and exclusivity to 12 weeks postpartum	Outcome
Olney 2006	Combined iron and folic acid supplementation with or without zinc reduces time to walking unassisted among Zanzibari infants 5- to 11-mo old	Population
Olsen 2000	The impact of iron supplementation on reinfection with intestinal helminths and Schistosoma mansoni in western Kenya	Population
Onayade 2004	THE FIRST SIX MONTH GROWTH AND ILLNESS OF EXCLUSIVELY AND NON-EXCLUSIVELY BREAST-FED INFANTS IN NIGERIA	Outcome
Osei 2015	Adding multiple micronutrient powders to a homestead food production programme yields marginally significant benefit on anaemia reduction among young children in nepal	Population
Osei A 2013	Using homestead food production program as a platform to deliver multiple micronutrient powders to infants and young children in Nepal	Other
Osrin 2005	Effects of antenatal multiple micronutrient supplementation on birthweight and gestational duration in Nepal: double-blind, randomised controlled trial.	Outcome
Owino 2007	Fortified complementary foods with or without alpha-amylase treatment increase hemoglobin but do not reduce breast milk intake of 9-mo-old Zambian infants	Outcome
Owino 2015	Winfood data from Kenya and Cambodia: Constraints on field procedures	Outcome
Paganini 2017	Prebiotic galacto-oligosaccharides mitigate the adverse effects of iron fortification on the gut microbiome: A randomised controlled study in Kenyan infants	Outcome
Palmer 2018	Impact of biofortified maize consumption on serum carotenoid concentrations in Zambian children	Outcome
Paragaroufalis 2014	A randomized double blind controlled safety trial evaluating d-lactic acid production in healthy infants fed a lactobacillus reuteri-containing formula	Outcome
Parker 2012	Effect of early breast milk expression on milk volume and timing of lactogenesis stage II among mothers of very low birth weight infants: A pilot study	Outcome
Partty 2013	Effects of early prebiotic and probiotic supplementation on development of gut microbiota and fussing and crying in preterm infants: A randomized, double-blind, placebo-controlled trial	Outcome
Pate 1999	EFFECTIVENESS OF WEB-BASED PROGRAMS IN IMPROVING BREASTFEEDING SELF-EFFICACY	Outcome
Paul 1996	Manual and pump methods of expression of breast milk	Outcome
Payne 2007	Benefit of vitamin a supplementation on ascaris reinfection is less evident in stunted children	Outcome
Pearson 2017	Effectiveness of household lockable pesticide storage to reduce pesticide self-poisoning in rural asia: A community-based, cluster-randomised controlled trial	Outcome

© 2019 Park JJH et al. *JAMA Network Open*.

First author, year	Title	Reason
Penny 2004	Randomized controlled trial of the effect of daily supplementation with zinc or multiple micronutrients on the morbidity, growth, and micronutrient status of young Peruvian children	Outcome
Penny 2005	Effectiveness of an educational intervention delivered through the health services to improve nutrition in young children: a cluster-randomised controlled trial.	Population
Pereira 1969	Lysine-supplemented wheat and growth of preschool children	Other
Pereira 1973	Feeding trials with lysine- and threonine-fortified rice	Other
Pereira 1979	A preliminary simulated iron fortification trial in South Indian preschool children	Other
Perkin 2016	Randomized trial of introduction of allergenic foods in breast-fed infants	Population
Perumal 2017	Prenatal vitamin d supplementation and infant vitamin d status in Bangladesh	Outcome
Pessoto 2010	Is a high quality expressed breast milk, according to expression method, possible for the premature baby in a third world country?	Outcome
Pettifor 1981	The effect of dietary calcium supplementation on serum calcium, phosphorus, and alkaline phosphatase concentrations in a rural black population	Outcome
Pham 2012	A six-month intervention with two different types of micronutrient-fortified complementary foods had distinct short- and long-term effects on linear and ponderal growth of Vietnamese infants	Population
Phuka 2009A	Supplementary feeding with fortified spread among moderately underweight 6-18-month-old rural Malawian children	Population
Phuka 2009B	Postintervention growth of Malawian children who received 12-mo dietary complementation with a lipid-based nutrient supplement or maize-soy flour.	Population
Phuka 2012	Developmental outcomes among 18-month-old Malawians after a year of complementary feeding with lipid-based nutrient supplements or corn-soy flour	Population
Pinelli 2001	Randomized trial of breastfeeding support in very low-birth-weight infants	Outcome
Pinnock 1988	Vitamin A status of children with a history of respiratory syncytial virus infection in infancy.	Other
Pisacane 1995	Iron status in breast-fed infants	Other
Pitchik H 2017	Prenatal nutrition, stimulation, and exposure to punishment are associated with early child motor, cognitive, and socioemotional development in dar es salaam, tanzania	Other
Pittard 1991	Bacterial contamination of human milk: container type and method of expression	Outcome
Pollitt 2000	Effects of an energy and micronutrient supplement on growth and development in undernourished children in Indonesia: Methods	Outcome
Pollitt 2000a	Effect of an energy and micro-nutrient supplement on growth and development in undernourished children in Indonesia: methods	Outcome
Ponder 1992	Docosahexaenoic acid status of term infants fed breast milk or infant formula containing soy oil or corn oil	Population
Pontes 2016	Cow's milk-based beverage consumption in 1- to 4-year-olds and allergic manifestations: An rct	Outcome
Prado 2012	Maternal multiple micronutrient supplements and child cognition: a randomized trial in Indonesia	Population
Prado 2016A	Effects of maternal and child lipid-based nutrient supplements on infant development: A randomized trial in Malawi	Outcome
Prado 2016B	Effects of pre- and post-natal lipid-based nutrient supplements on infant development in a randomized trial in Ghana	Outcome
Prado 2016C	Lipid-based nutrient supplements plus malaria and diarrhea treatment increase infant development scores in a cluster-randomized trial in burkina faso	Outcome
Prado 2016D	Provision of lipid-based nutrient supplements from age 6 to 18 months does not affect infant development scores in a randomized trial in Malawi	Population
Prasad 2016	Impact of Sorghum supplementation on growth and micronutrient status of school going children in Southern India — a randomized trial	Population
Prendergast 2011	Improved growth and anemia in HIV-infected African children taking cotrimoxazole prophylaxis	Population
Prime 2010	The effect of breastshield size and anatomy on milk removal in women	Outcome
Prudhon 2017	Effect of ready-to-use foods for preventing child undernutrition in niger: Analysis of a prospective intervention study over 15months of follow-up	Study design
Pulakka 2015	Effect of 12-month intervention with lipid-based nutrient supplements on physical activity of 18-month-old Malawian children: A randomised, controlled trial	Outcome
Pulakka 2017	Effect of 12-month intervention with lipid-based nutrient supplement on the physical activity of Malawian toddlers: A randomised, controlled trial	Outcome
Punthmatharith 2001	Randomized Controlled Trial of early Kangaroo Care (Skin-to-Skin) Care: Effects on Maternal Feelings, Maternal-Infant Interaction and Breastfeeding Success in Thailand dissertation]	Outcome
Radhakrishna 2013	Effectiveness of zinc supplementation to full term normal infants: A community based double blind, randomized, controlled, clinical trial	Population
Raeisi 2014	A single center study of the effects of trained fathers' participation in constant breastfeeding	Outcome
Rahman 1999	Long-term supplementation with iron does not enhance growth in malnourished	Population

© 2019 Park JJH et al. *JAMA Network Open*.

First author, year	Title	Reason
	Bangladeshi children	
Rahman 2001	Simultaneous zinc and vitamin A supplementation in Bangladeshi children: randomised double blind controlled trial	Outcome
Rahman 2002	Short-term supplementation with zinc and vitamin a has no significant effect on the growth of undernourished Bangladeshi children	Population
Ramakrishnan 1995	Vitamin a supplementation does not improve growth of preschool children: A randomized, double-blind field trial in south India	Other
Ramakrishnan 2003	Multiple micronutrient supplementation during pregnancy does not lead to greater infant birth size than does iron-only supplementation: a randomized controlled trial in a semirural community in Mexico	Outcome
Ramakrishnan 2004	Multimicronutrient interventions but not vitamin a or iron interventions alone improve child growth: Results of 3 meta-analyses	Study design
Ramakrishnan 2009	Multiple micronutrient supplementation during early childhood increases child size at 2 y of age only among high compliers	Population
Ramanathan 2001	Kangaroo Mother Care in Very Low Birth Weight Infants	Study design
Ramirez-Luzuriaga 2016	A food transfer program without a formal education component modifies complementary feeding practices in poor rural Mexican communities	Outcome
Rao 1992	Prolonged breast-feeding and malnutrition among rural Indian children below 3 years of age.	Outcome
Rasmussen 2011	Interventions to increase the duration of breastfeeding in obese mothers: the Bassett Improving Breastfeeding Study	Outcome
Rawat R 2015	Sale of micronutrient powders (mnps) by frontline workers (flws) enables high reach, but low uptake limits impact on anemia and iron status: A cluster randomized study in Bangladesh	Other
Reinbott 2016	Nutrition education linked to agricultural interventions improved child dietary diversity in rural Cambodia	Study design
Rivera 1991	Effect of supplementary feeding on recovery from mild to moderate wasting in preschool children	Outcome
Rivera 1995	Nutritional supplementation during the preschool years influences body size and composition of Guatemalan adolescents	Outcome
Rivera 1996	The recovery of Guatemalan children with mild to moderate wasting: Factors enhancing the impact of supplementary feeding	Outcome
Rivera 1998	Zinc supplementation improves the growth of stunted rural Guatemalan infants	Population
Rivera 2001	Multiple micronutrient supplementation increases the growth of Mexican infants	Population
Roberfroid 2008	Effects of maternal multiple micronutrient supplementation on fetal growth: a double-blind randomized controlled trial in rural Burkina Faso	Population
Roberfroid 2012	Impact of prenatal multiple micronutrients on survival and growth during infancy: a randomized controlled trial.	Outcome
Roberts 2000	A Comparison of Kangaroo Mother Care and Conventional Cuddling Care	Outcome
Rojas 2003	Somatic Growth of Preterm Infants During Skin-to-Skin Care Versus Traditional Holding: A Randomized, Controlled Trial	Population
Ronaghy 1969	Controlled zinc supplementation for malnourished school boys: a pilot experiment	Population
Ronaghy 1974	Zinc supplementation of malnourished schoolboys in Iran: increased growth and other effects	Population
Rosado 1997	Zinc supplementation reduced morbidity, but neither zinc nor iron supplementation affected growth or body composition of Mexican preschoolers	Population
Rosado 1999	Separate and joint effects of micronutrient deficiencies on linear growth	Population
Rosado 2009	Interaction of zinc or vitamin a supplementation and specific parasite infections on Mexican infants' growth: A randomized clinical trial	Population
Rosado 2011	Effectiveness of the nutritional supplement used in the Mexican oportunidades programme on growth, anaemia, morbidity and cognitive development in children aged 12-24 months	Population
Rossiter	The effect of a culture-specific education program to promote breastfeeding among Vietnamese women in Sydney	Outcome
Rothman 2015	Acceptability of novel small-quantity lipid-based nutrient supplements for complementary feeding in a peri-urban south african community	Outcome
Roy 1999	Impact of zinc supplementation on subsequent growth and morbidity in Bangladeshi children with acute diarrhoea	Outcome
Roy 2007	Prevention of malnutrition among young children in rural Bangladesh by a food-health-care educational intervention: a randomized, controlled trial.	Population
Ruel 1997	Impact of zinc supplementation on morbidity from diarrhea and respiratory infections among rural Guatemalan children	Outcome
Ruz 1997	A 14-mo zinc-supplementation trial in apparently healthy Chilean pre- school children	Population
Ryan 2014	Zinc or albendazole attenuates the progression of environmental enteropathy: A randomized controlled trial	Outcome
Ryser 2004	Breastfeeding attitudes, intention, and initiation in low-income women: the effect of the best start program.	Outcome
Saadi 2009	Effect of combined maternal and infant vitamin D supplementation on vitamin D status of	Outcome

First author, year	Title	Reason
	exclusively breastfed infants	
Saleem 2014	Impact of maternal education about complementary feeding on their infants' nutritional outcomes in low- and middle-income households: A community-based randomized interventional study in karachi, Pakistan	Population
Santos 2001	Nutrition counseling increases weight gain among Brazilian children.	Population
Santosham 1990	A comparison of rice-based oral rehydration solution and 'early feeding' for the treatment of acute diarrhea in infants	Population
Sarkar 2017	Study design and baseline results of an open-label cluster randomized community-intervention trial to assess the effectiveness of a modified mass deworming program in reducing hookworm infection in a tribal population in southern India	Outcome
Sarma 2006	Effect of micronutrient supplementation on health and nutritional status of schoolchildren: growth and morbidity	Population
Sato W 2017	A macro and micronutrient fortified complementary food supplement enhances plasma branched-chain amino acid levels in Ghanaian infants	Other
Sattar 2012	Efficacy of a high-dose in addition to daily low-dose vitamin a in children suffering from severe acute malnutrition with other illnesses	Study design
Savilahti 1987	Prolonged exclusive breast feeding and heredity as determinants in infantile atopy	Outcome
Sayeg 2000	Linear growth and zinc supplementation in children with short stature	Other
Sazawal 2006	Zinc supplementation reduces the incidence of persistent diarrhea and dysentery among low socioeconomic children in India	Outcome
Scalabrin 2009	Growth and Tolerance of Healthy Term Infants Receiving Hydrolyzed Infant Formulas Supplemented With Lactobacillus rhamnosus GG: Randomized, Double-Blind, Controlled Trial	Population
Schlegelmilch 2016	Evaluation of water, sanitation and hygiene program outcomes shows knowledge-behavior gaps in coast province, Kenya	Outcome
Schlickau 2005	Development and testing of a prenatal breastfeeding education intervention for Hispanic women	Outcome
Schroeder 1995	Age differences in the impact of nutritional supplementation on growth	Outcome
Schwartz 2014	Medium-term impact of a pro-breastfeeding and healthy complementary feeding intervention on growth and prevalence of overweight in children: a randomized clinical trial with adolescent mothers and grandmothers	Outcome
Scott 1998	Formula supplementation with long-chain polyunsaturated fatty acids: Are there developmental benefits?	Population
Scrimshaw 1953	Effect of dietary supplements and the administration of vitamin B12 and aureomycin on the growth of school children	Population
Semba 1995	Reduced seroconversion to measles in infants given vitamin A with measles vaccination	Outcome
Semba 2005	Effect of periodic vitamin a supplementation on mortality and morbidity of human immunodeficiency virus-infected children in Uganda: A controlled clinical trial	Outcome
Sempértegui 1996	Effects of short- term zinc supplementation on cellular immunity, respiratory symp- toms, and growth of malnourished Equadorian children	Other
Sempertegui 1999	The beneficial effects of weekly low-dose vitamin A supplementation on acute lower respiratory infections and diarrhea in Ecuadorian children.	Outcome
Serwint 1996	A randomized controlled trial of prenatal pediatric visits for urban, low-income families	Population
Shafique S 2013	Prevention of linear growth faltering among low birth weight infants in rural Bangladesh: A community-based cluster randomized trial	Population
Shafique S 2016	Mineral- and vitamin-enhanced micronutrient powder reduces stunting in full-term low-birth-weight infants receiving nutrition, health, and hygiene education: a 2 3 2 factorial, cluster-randomized trial in Bangladesh	Other
Shankar 2008	Effect of maternal multiple micronutrient supplementation on fetal loss and infant death in Indonesia: a double-blind cluster-randomised trial	Population
Sharieff 2006	Short- term daily or weekly administration of micronutrient Sprinkles has high compliance and does not cause iron overload in Chinese schoolchildren: a cluster-randomised trial	Outcome
Sharma 2016A	Efficacy of early skin-to-skin contact on the rate of exclusive breastfeeding in term neonates: A randomized controlled trial	Outcome
Sharma 2016B	The effect of kangaroo ward care in comparison with "intermediate intensive care" on the growth velocity in preterm infant with birth weight <1100 g: Randomized control trial	Population
Sharma 2016C	To compare cost effectiveness of 'kangaroo ward care' with 'intermediate intensive care' in stable very low birth weight infants (birth weight<1100 grams): A randomized control trial	Outcome
Sharma 2017	Study comparing "kangaroo ward care" with "intermediate intensive care" for improving the growth outcome and cost effectiveness: Randomized control trial	Other
Shatrugna 2006	Effect of micronutrient supplement on health and nutritional status of schoolchildren: bone health and body composition	Population
Sheikh 2017	The efficacy of early iron supplementation on postpartum depression, a randomized double-blind placebo-controlled trial	Outcome
Shiau 1997	Randomized controlled trial of kangaroo care with full term infants: effects on breastmilk maturation, breast engorgement, and breastfeeding status	Outcome
Siddiqua 2016	Vitamin b12 supplementation during pregnancy and postpartum improves b12 status of	Outcome

© 2019 Park JJH et al. *JAMA Network Open*.

First author, year	Title	Reason
	both mothers and infants but vaccine response in mothers only: A randomized clinical trial in Bangladesh	
Sikander 2015	Cognitive-behavioral counseling for exclusive breastfeeding in rural pediatrics: A cluster rct	Outcome
Silva 2008	Growth in non-anemic infants supplemented with different prophylactic iron doses	Other
Simondon 1997	Age at introduction of complementary food and physical growth from 2 to 9 months in rural Senegal	Outcome
Simondon 1997a	Lactational amenorrhea is associated with child age at the time of introduction of complementary food: a prospective cohort study in rural Senegal,West Africa.	Outcome
Singh H 2017	Daily supplementation with 400 iu vitamin d in term breast fed infants from 0-6 months and changes in total and bone specific alkaline phosphatase-a rct	Outcome
Singla 2014	A 22-element micronutrient powder benefits language but not cognition in Bangladeshi full-term low-birth-weight children	Population
Skau 2015	Effects of animal source food and micronutrient fortification in complementary food products on body composition, iron status, and linear growth: A randomized trial in cambodia	Population
Skau Jkh 2014	The use of linear programming to determine whether a formulated complementary food product can ensure adequate nutrients for 6- to 11-month-old Cambodian infants	Study design
Slayton 2016	A cluster randomized controlled evaluation of the health impact of a novel antimicrobial hand towel on the health of children under 2 years old in rural communities in nyanza province, Kenya	Outcome
Sloan 1994	Kangaroo mother metthod: randomised controlled trial of an alternative method of care for stabilised low-birthweight infants	Outcome
Slusher 2007	Electric breast pump use increases maternal milk volume in African nurseries	Outcome
Smith 2014	Multiple micronutrient supplementation transiently ameliorates environmental enteropathy in Malawian children aged 12-35 months in a randomized controlled clinical trial	Outcome
Smith 2016	The effect of neonatal vitamin a supplementation on morbidity and mortality at 12 months: A randomized trial	Outcome
Smuts 2005A	Efficacy of a foodlet-based multiple micronutrient supplement for preventing growth faltering, anemia, and micronutrient deficiency of infants: The four country iris trial pooled data analysis	Population
Smuts 2005B	Efficacy of multiple micronutrient supplementation for improving anemia, micronutrient status, and growth in south african infants	Population
Sneed 1981	The effects of ascorbic acid, vitamin B6, vitamin B12, and folic acid supplementation on the breast milk and maternal nutritional status of low socioeconomic lactating women	Population
Solon 2003	Effect of a multiple-micronutrient- fortified fruit powder beverage on the nutrition status, physical fitness, and cognitive performance of schoolchildren in the Philippines	Population
Some 2015	Effect of zinc added to a daily small-quantity lipid-based nutrient supplement on diarrhoea, malaria, fever and respiratory infections in young children in rural burkina faso: A cluster-randomised trial	Outcome
Somnath 2017	Therapeutic effect of vitamin d in acute lower respiratory infection: A randomized controlled trial	Outcome
Soofi 2017	Evaluation of the uptake and impact of neonatal vitamin a supplementation delivered through the lady health worker programme on neonatal and infant morbidity and mortality in rural Pakistan: An effectiveness trial	Outcome
Sosa 1976	The effect of early mother-infant contact on breastfeeding, infection and growth	Outcome
Specker 2003	Randomized trial of physical activity and calcium supplementation on bone mineral content in 3- to 5-year-old children	Other
Srinivasan 2016	Household and personal factors are sources of heterogenity in intestinal parasite clearance among Mexican children 6-15 months of age supplemented with vitamin a and zinc	Outcome
Srivastava 2014	Effect of very early skin to skin contact on success at breastfeeding and preventing early hypothermia in neonates	Study design
Stabell 1995	No evidence of fontanelle-bulging episodes after vitamin A supplementation of 6- and 9-month-old infants in Guinea Bissau	Other
Stellwagen 2010	Hand expression combined with pumping does not change the nutrient content of human milk from mothers of very low birthweight infants	Outcome
Stewart 2017A	Biological pathways through which eggs may influence child growth and development	Other
Stewart 2017B	The impact of maternal diet fortification with lipid-based nutrient supplements on postpartum depression in rural Malawi: A randomised-controlled trial	Outcome
Stewart C 2017	Factorial trial of lipid-based nutrient supplements with infant and young child feeding counseling with or without improved wash in Kenya: Effects on anemia, iron, vitamin a, vitamin b12 and folate	Other
Stobaugh 2016	Including whey protein and whey permeate in ready-to-use supplementary food improves recovery rates in children with moderate acute malnutrition: A randomized, double-blind clinical trial	Outcome

© 2019 Park JJH et al. *JAMA Network Open*.

First author, year	Title	Reason
Stobaugh 2017	Effect of a package of health and nutrition services on sustained recovery in children after moderate acute malnutrition and factors related to sustaining recovery: A cluster-randomized trial	Outcome
Stoltzfus 2001	Effects of iron supplementation and anthelmintic treatment on motor and language development of preschool children in Zanzibar: double blind, placebo controlled study	Outcome
Stoltzfus 2004	Low dose daily iron supplementation improves iron status and appetite but not anemia, whereas quarterly anthelminthic treatment improves growth, appetite and anemia in zanzibari preschool children	Population
Strand 2015	Vitamin b-12, folic acid, and growth in 6- to 30-month-old children: A randomized controlled trial	Outcome
Stutte 1988	The effects of breast massage on volume and fat content of human milk	Other
Suchdev 2010	Monitoring the marketing, distribution, and use of Sprinkles micronutrient powders in rural western Kenya. Food & Nutrition Bulletin 2010, 31(Supplement 2):	Outcome
Suchdev 2012	Selling sprinkles micronutrient powder reduces anemia, iron deficiency, and vitamin a deficiency in young children in western Kenya: A cluster-randomized controlled trial	Outcome
Suchdev 2016	Effects of community-based sales of micronutrient powders on morbidity episodes in preschool children in western Kenya	Outcome
Sudfeld 2015	Malnutrition and its determinants are associated with suboptimal cognitive, communication, and motor development in tanzanian children	Study design
Sung 2014	Treating infant colic with the probiotic Lactobacillus reuteri: Double blind, placebo controlled randomised trial	Outcome
Sungthong 2002	Once weekly is superior to daily iron supplementation on height gain but not on hematological improvement among schoolchildren in Thailand	Population
Sur 2003	Impact of zinc supplementation on diarrheal morbidity and growth pattern of low birth weight infants in kolkata, India: a randomized, double-blind, placebo-controlled, community-based study.	Population
Surkan 2015	The role of zinc and iron-folic acid supplementation on early child temperament and eating behaviors in rural nepal: A randomized controlled trial	Population
Svefors 2018	Cost-effectiveness of prenatal food and micronutrient interventions on under-five mortality and stunting: Analysis of data from the minimat randomized trial, Bangladesh	Study design
Svejda 1980	Mother-infant bonding: failure to generalize. Child Development	Population
Swarnkar 2016	Effect of kangaroo mother care on growth and morbidity pattern in low birth weight infants.	Study design
Syfrett 1993	Very Early and Virtually Continuous Kangaroo Care for 34-36 week Gestation Preterm Infants: Effects on Temperature, Breastfeeding, Supplementation and Weight [thesis]	Outcome
Tahan 2007	A randomized double-blind clinical trial of the effect of non-absorbable oral polymyxin on infants with severe infectious diarrhea	Outcome
Talukder A 2017	The role of small scale aquaculture and enhanced homestead food production in improving household food security and nutrition	Outcome
Tanchoco 2007	Diet supplemented with mct oil in the management of childhood diarrhea	Outcome
Taneja 2010	Zinc supplementation for four months does not affect growth in young north Indian children	Population
Tang 2014	Meat as complementary food for older breastfed infants and toddlers: A randomized, controlled trial in rural china	Population
Tang 2017	Iron in micronutrient powder promotes an unfavorable gut microbiota in Kenyan infants	Outcome
Tariku 2015	Application of the health belief model to teach complementary feeding messages in ethiopia	Outcome
Tavil 2003	Effect of twice weekly versus daily iron treatment in Turkish children with iron deficiency anemia.	Outcome
Tchum 2006	Evaluation of vitamin a supplementation regimens in Ghanaian postpartum mothers with the use of the modified-relative-dose-response test	Outcome
Tergestina 2016	A randomized double-blind controlled trial comparing two regimens of vitamin d supplementation in preterm neonates	Outcome
Teshome 2018	Adherence to home fortification with micronutrient powders in Kenyan pre-school children: Self-reporting and sachet counts compared to an electronic monitoring device	Outcome
Thakur 2012	Effect of nutrition education on exclusive breastfeeding for nutritional outcome of low birth weight babies	Outcome
Thakwalakwa 2010	A lipid-based nutrient supplement but not corn-soy blend modestly increases weight gain among 6- to 18-month-old moderately underweight children in rural Malawi	Population
Thakwalakwa 2012	An effectiveness trial showed lipid-based nutrient supplementation but not corn-soya blend offered a modest benefit in weight gain among 6- to 18-month-old underweight children in rural Malawi	Population
Thomson 1979	The importance of immediate postnatal contact: its effect on breastfeeding	Outcome
Thu 1999	Effect of daily and weekly micronutrient supplementation on micronutrient deficiencies and growth in young Vietnamese children	Population
Thukral 2012	Early skin-to-skin contact and breast-feeding behavior in term neonates: a randomized controlled trial	Outcome

© 2019 Park JJH et al. *JAMA Network Open*.

First author, year	Title	Reason
Timby 2014	Neurodevelopment, nutrition, and growth until 12 mo of age in infants fed a low-energy, low-protein formula supplemented with bovine milk fat globule membranes: A randomized controlled trial	Outcome
Tofall 2008	Effects of prenatal food and micronutrient supplementation on infant development: a randomized trial from the Maternal and Infant Nutrition Interventions, Matlab (MINIMat) study	Population
Tomiya 2017	The effect of vitamin a supplementation with 400 000 iu vs 200 000 iu on retinol concentrations in the breast milk: A randomized clinical trial	Outcome
Torabi 2014	The effect of calcium and phosphorus supplementation on metabolic bone disorders in premature infants	Outcome
Torrejon 2004	Zinc and Iron Nutrition in Chilean Children Fed Fortified Milk Provided by the Complementary National Food Program	Outcome
Trehan 2009	A randomized, double-blind, placebo-controlled trial of rifaximin, a nonabsorbable antibiotic, in the treatment of tropical enteropathy	Population
Trehan 2013	Antibiotics as part of the management of severe acute malnutrition	Population
Tuthill 2002	Randomized double-blind controlled trial on the effects on iron status in the rst year between a no added iron and standard infant formula received for three months*	Outcome
Ullah B 2017	Effect of pre-and postnatal nutritional supplements on childhood illnesses in Bangladesh: A cluster-randomized effectiveness trial	Other
Umeta 2000	Zinc supplementation and stunted infants in Ethiopia: a randomised controlled trial	Population
Unger 2017	Impact of fortified versus unfortified lipid-based supplements on morbidity and nutritional status: A randomised double-blind placebo-controlled trial in ill Gambian children	Outcome
Untoro 2005	Multiple micronutrient supplements improve micronutrient status and anemia but not growth and morbidity of Indonesian infants: A randomized, double-blind, placebo-controlled trial	Population
Vahdati 2017	Effect of kangaroo care combined with music on the mother-premature neonate attachment: A randomized controlled trial	Outcome
Vaidya 2005	Effect of early mother-baby close contact over the duration of exclusive breastfeeding	Outcome
Vaidya 2008	Effects of antenatal multiple micronutrient supplementation on children's weight and size at 2 years of age in nepal: Follow-up of a double-blind randomised controlled trial	Population
van Goor 2010	Supplementation of DHA but not DHA with arachidonic acid during pregnancy and lactation influences general movement quality in 12-week- old term infants	Outcome
Vasan 2004	Maternal milk volume (MMV) and milk transfer (MT) for low birth weight (LBW: <2500G) infants in Ecuador: comparison of electric breast pump and hand expression	Population
Vaziri 2016	Vitamin d supplementation during pregnancy on infant anthropometric measurements and bone mass of mother-infant pairs: A randomized placebo clinical trial	Outcome
Veenemans 2011	Effect of supplementation with zinc and other micronutrients on malaria in tanzanian children: A randomised trial	Outcome
Veenemans 2012	Effect of preventive supplementation with zinc and other micronutrients on non-malarial morbidity in tanzanian pre-school children: A randomized trial	Outcome
Venkatarao 1996	Effect of vitamin A supplementation to mother and infant on morbidity in infancy.	Outcome
Verhoef 2002	Malarial anemia leads to adequately increased erythropoiesis in asymptomatic Kenyan children.	Outcome
Verna M 2012	Parma pap project: A ready to use therapeutic food for moderately malnourished children in sierra leone	Other
Villalon 1992	Effect of early skin-to-skin contact on temperature regulation, heart rate, and respiratory rate in healthy, full-term newborns [Contacto precoz piel a piel: efecto sobre los parametros fisiologicos en las cuatro horas posteriores al parto en recien nacidos de termino sanos]	Study design
Villamor 2005	Vitamin supplementation of HIV-infected women improves postnatal child growth	Outcome
Vinod 2006	Impact of a multiple-micronutrient food supplement on the nutritional status of schoolchildren	Population
Wagner 2006	High-dose vitamin D3 supplementation in a cohort of breast- feeding mothers and their infants: a 6-month follow-up pilot study.	Population
Walker 1991	Nutritional supplementation, psychosocial stimulation, and growth of stunted children: the Jamaican study.	Population
Walravens 1983	Linear growth of low income pre- school children receiving a zinc supplement	Other
Walravens 1989	Iron and zinc supplementation improved iron and zinc status, but not physical growth, of apparently healthy, breast- fed infants in rural communities of northeast Thailand	Population
Walravens Pa 1992	Zinc supplements in breastfed infants	Outcome
Walter 1993	Effectiveness of Iron-Fortified Infant Cereal in Prevention of Iron Deficiency Anemia	Outcome
Wammes 2016	Community deworming alleviates geohelminth-induced immune hyporesponsiveness	Outcome
Wang 2005	Feeding practices in 105 counties in rural China.	Study design
Wang 2012	No effect of maternal micronutrient supplementation on early childhood growth in rural western China: 30 month follow-up evaluation of a double blind, cluster randomized controlled trial	Population
Wang 2017A	A combined intervention of zinc, multiple micronutrients, and albendazole does not	Outcome

© 2019 Park JJH et al. *JAMA Network Open*.

First author, year	Title	Reason
	ameliorate environmental enteric dysfunction or stunting in rural Malawian children in a double-blind randomized controlled trial	
Wang 2017B	Effectiveness of community-based complementary food supplement (yingyangbao) distribution in children aged 6-23 months in poor areas in china	Study design
Warthon-Medina 2015	The long term impact of micronutrient supplementation during infancy on cognition and executive function performance in pre-school children	Outcome
Warthon-Medina M 2015	The long term impact of multiple micronutrient supplementation during infancy on cognition and executive function performance in pre-school children	Study design
Wasantwisut 2006	Iron and zinc supplementation improved iron and zinc status, but not physical growth, of apparently healthy, breast-fed infants in rural communities of northeast Thailand.	Population
Wegmuller 2016	Efficacy and safety of hepcidin-based screen-and-treat approaches using two different doses versus a standard universal approach of iron supplementation in young children in rural Gambia: A double-blind randomised controlled trial	Outcome
Weizman 2006	Effect of a probiotic infant formula on infections in child care centers: comparison of two probiotic agents	Population
Wen 2012	Effectiveness of home based early intervention on children's BMI at age 2: randomised controlled trial	Outcome
West 1997	Effects of vitamin a on growth of vitamin a-deficient children: Field studies in nepal	Population
West 2014	Effect of maternal multiple micronutrient vs iron-folic acid supplementation on infant mortality and adverse birth outcomes in rural Bangladesh: the JiVitA-3 randomized trial	Outcome
Whitelaw 1988	Skin to skin contact for very low birth weight infants and their mothers	Outcome
Whiting S 2017	Feasibility study of an intervention of gifting chickens for young children in ethiopia for provision of egg and eggshell	Other
WHO 1994	An Evaluation of Infant Growth	Outcome
WHO 1997	Effects of timing of complementary foods on post-natal growth	Outcome
Widen 2015	Antiretroviral treatment is associated with iron deficiency in HIV-infected Malawian women that is mitigated with supplementation, but is not associated with infant iron deficiency during 24 weeks of exclusive breastfeeding	Outcome
Wieringa 2003	Redistribution of vitamin A after iron supplementation in Indonesian infants	Population
Willatts 1998a	Influence of long-chain polyunsaturated fatty acids on infant cognitive function	Population
Willatts 1998b	Effect of long-chain polyunsaturated fatty acids in infant formula on problem solving at 10 months of age	Population
Williams 2007	A double-blind, placebo-controlled, glutamine-supplementation trial in growth-faltering Gambian infants	Population
Wiria 2013	The effect of three-monthly albendazole treatment on malarial parasitemia and allergy: a household-based cluster-randomized, double-blind, placebo-controlled trial.	Population
Wolfberg 2004	Dads as breastfeeding advocates: results from a randomized controlled trial of an educational intervention	Outcome
Wolfsdorf 1973	Trimethoprim-sulphonamide mixture in the treatment of infantile gastro-enteritis	Outcome
Wong 2014	Antenatal education to increase exclusive breastfeeding. A randomized controlled trial	Outcome
Worku 2005	Kangaroo Mother Care: A Randomized Controlled Trial on Effectiveness of Early Kangaroo Mother Care for the Low Birthweight Infants in Addis Ababa, Ethiopia	Outcome
Wright 2016	Subsidized sachet water to reduce diarrheal disease in young children: A feasibility study in accra, Ghana	Outcome
Wuehler 2008	Dose-response trial of prophylactic zinc supplements, with or without copper, in young Ecuadorian children at risk of zinc deficiency	Outcome
Yang 2002	Effect of micronutrient supplementation on the growth of preschool children in China	Other
Yang 2004	Effect of daily or once weekly iron supplementation on growth and iron status of preschool children	Population
Yi it 2012	Does warming the breasts affect the amount of breastmilk production?	Outcome
Young M 2017	Role of preconception nutrition in offspring growth and risk of stunting across the first 1000 days in vietnam	Other
Yousafzai 2014	Effect of integrated responsive stimulation and nutrition interventions in the lady health worker programme in Pakistan on child development, growth, and health outcomes: A cluster-randomised factorial effectiveness trial	Outcome

© 2019 Park JJH et al. *JAMA Network Open*.

First author, year	Title	Reason
Yousefichaijan 2015	Oral zinc sulfate as adjuvant treatment in children with nephrolithiasis: A randomized, double-blind, placebo-controlled clinical trial	Outcome
Yurdakok 2004	Efficacy of daily and weekly iron supplementation on iron status in exclusively breast-fed infants	Outcome
Zadik 2004	Vitamin A and iron supplemen- tation is as efficient as hormonal therapy in constitutionally delayed children	Outcome
Zadik 2010	"Functional food" for acceleration of growth in short children born small for gestational age	Other
Zaman 2008	Training in complementary feeding counselling of healthcare workers and its influence on maternal behaviours and child growth: a cluster-randomized controlled trial in Lahore, Pakistan.	Population
Zeng 2008	Impact of micronutrient supplementation during pregnancy on birth weight, duration of gestation, and perinatal mortality in rural western China: double blind cluster randomised controlled trial	Outcome
Zhang 2016	Effectiveness of complementary food supplements and dietary counselling on anaemia and stunting in children aged 6-23 months in poor areas of qinghai province, china: A controlled interventional study	Outcome
Ziegler 2009	Iron status of breastfed infants is improved equally by medicinal iron and iron-fortified cereal	Population
Zimmermann 2007	Treatment of iodine deficiency in school-age children increases insulin-like growth factor (IGF)-I and IGF binding protein-3 concentrations and improves somatic growth	Population
Zinaman 1992	Acute prolactin and oxytocin responses and milk yield to infant suckling and artificial methods of expression in lactating women	Outcome
Zlotkin 2003	Home-Fortification with Iron and Zinc Sprinkles or Iron Sprinkles Alone Successfully Treats Anemia in Infants and Young Children	Population
Zlotkin 2013	Effect of iron fortification on malaria incidence in infants and young children in Ghana: A randomized trial	Outcome

© 2019 Park JJH et al. *JAMA Network Open*.

eTable 13. List of excluded studies for complementary feeding stage with reasons

First author, year	Title	Reason for exclusion
Aaby 2010	Non-specific effects of standard measles vaccine at 4.5 and 9 months of age on childhood mortality: Randomised controlled trial	Intervention
Aakko 2017	Lipid-based nutrient supplements do not affect gut bifidobacterium microbiota in malawian infants: A randomized trial	Outcome
Acharya 2014	Randomized Control Trial of Kangaroo Mother Care in Low Birth Weight Babies at a Tertiary Level Hospital	Study design
Adair 1994	Growth dynamics during the first two years of life: A prospective study in the Philippines	Study design
Adams 2017	Maternal and child supplementation with lipid-based nutrient supplements, but not child supplementation alone, decreases self-reported household food insecurity in some settings	Outcome
Adu-Afarwuah 2016	Small-quantity, lipid-based nutrient supplements provided to women during pregnancy and 6 mo postpartum and to their infants from 6 mo of age increase the mean attained length of 18-mo-old children in semi-urban ghana: A randomized controlled trial	Population
Adu-Afarwuah 2017	Maternal supplementation with small-quantity lipid-based nutrient supplements compared with multiple micronutrients, but not with iron and folic acid, reduces the prevalence of low gestational weight gain in semi-urban ghana: A randomized controlled trial	Population
Aggarwal 2016	Selenium supplementation for prevention of late-onset sepsis in very low birth weight preterm neonates	Outcome
Agostoni 1995	Neurodevelopmental quotient of healthy term infants at 4 months and feeding practice: The role of long-chain polyunsaturated fatty acids	Outcome
Agostoni 1997	Developmental quotient at 24 months and fatty acid composition of diet in early infancy: A follow up study.	Other
Agostoni 2007	Functional ingredients in the complementary feeding period and long-term effects.	Study design
Agudelo 2016	The effect of skin-to-skin contact at birth, early versus immediate, on the duration of exclusive human lactancy in full-term newborns treated at the clinica universidad de la sabana: Study protocol for a randomized clinical trial	Other
Ahmad 2015	The effect of postpartum vitamin a supplementation on breast milk immune regulators and infant immune functions: Study protocol of a randomized, controlled trial	Other
Ahmad 2016	Maternal zinc supplementation improves hepatitis b antibody responses in infants but decreases plasma zinc level	Outcome
Ahmed 2008	Breastfeeding preterm infants: an educational program to support mothers of preterm infants in Cairo, Egypt	Outcome
Ahn 1980	Growth of the exclusively breast-fed infant.	Study design
Akeson 1996	Human milk and standard infant formula together with high quality supplementary foods is sufficient for normal growth during infancy.	Study design
Alarcon 1991	Clinical trial of home available, mixed diets versus a lactose-free, soy-protein formula for the dietary management of acute childhood diarrhea	Outcome
Alarcon 1992	Effect of inclusion of beans in a mixed diet for the treatment of Peruvian children with acute watery diarrhea	Outcome
Alarcon 2003	Effect of oral supplementation on catch-up growth in picky eaters	Population
Ali 2009	Kangaroo Mother Care as compared to conventional care for low birth weight babies Düşük doğum ağırlıklı bebekler için Kanguru anne bakımının geleneksel bakımla karşılaştırılması	Outcome
Ali 2017	Effect of maternal antenatal and newborn supplementation with vitamin a on cognitive development of school-aged children in rural bangladesh: A follow-up of a placebo-controlled, randomized trial	Study design
Altmann M 2017	The impact of adding a household wash package to cmam program: A cluster-randomized controlled trial in chad	Outcome
Anderson 2003	Early skin-to-skin contact for mothers and their healthy newborn infants.	Outcome
Anderson 2010	Effects of maternal micronutrient supplementation on fetal loss and under- 2-years child mortality: long-term follow-up of a randomised controlled trial from Guinea-Bissau	Population
Angeles 1993	Decreased rate of stunting among anemic Indonesian preschool children through iron supplementation	Population
Anonymous 2017A	Effect of heat application during intramuscular injection of vitamin k in pain prevention in neonates	Outcome
Anonymous 2017B	Effects of preconceptional weekly micronutrient supplements on maternal and child anemia during the first 2 years of life	Outcome

© 2019 Park JJH et al. *JAMA Network Open*.

First author, year	Title	Reason for exclusion
Armbrust 2016	The Charite cesarean birth: a family orientated approach of cesarean section	Outcome
Ash 2003	Randomized efficacy trial of a micronutrient-fortified beverage in primary school children in Tanzania	Population
Ashorn 2015B	The impact of lipid-based nutrient supplement provision to pregnant women on newborn size in rural Malawi: a randomized controlled trial	Population
Auerbach 1990	Sequential and simultaneous breast pumping: a comparison.	Outcome
Auestad 1997	Visual acuity, erythrocyte fatty acid composition, and growth in term infants fed formulas with long chain polyunsaturated fatty acids for one year.	Population
Auestad 2001	Growth and development in term infants fed long-chain polyunsaturated fatty acids: A double-masked, randomized, parallel, prospective, multivariate study.	Population
Awasthi 2005	Effectiveness of biweekly versus daily iron-folic acid administration on anaemia status in preschool children	Population
Banupriya 2018	Short term oral zinc supplementation among babies with neonatal sepsis for reducing mortality and improving outcome - a double-blind randomized controlled trial	Outcome
Baqui 2003	Simultaneous weekly supplementation of iron and zinc is associated with lower morbidity due to diarrhea and acute lower respiratory infection in Bangladeshi infants	Outcome
Bass 2007	Exercise and calcium combined results in a greater osteogenic effect than either factor alone: a blinded randomized placebo-controlled trial in boys	Other
Bates 1993	A trial of zinc supplementation in young rural Gambian children.	Outcome
Beiranvand 2014	The effects of skin-to-skin contact on temperature and breastfeeding successfulness in full-term newborns after cesarean delivery.	Outcome
Benn 1997	Randomised trial of effect of vitamin A supplementation on antibody response to measles vaccine in Guinea-Bissau, West Africa	Outcome
Berger 1997	Effect of early, short-term supplementation on weight and linear growth of 4-7-mo-old infants in developing countries: a four-country randomized trial	Population
Bergman 2004	Kangaroo mother care from birth compared to conventional incubator care	Outcome
Bernabe-Garcia 2012	Effectiveness of four manual breast pumps for mothers after preterm delivery in a developing country	Outcome
Bhan 1988	Comparison of a lactose-free cereal-based formula and cow's milk in infants and children with acute gastroenteritis	Outcome
Bhandari 2001	Food supplementation with encouragement to feed it to infants from 4 to 12 months of age has a small impact on weight gain	Outcome
Bhandari 2002	Effect of routine zinc supplementation on pneumonia in children aged 6 months to 3 years: randomised controlled trial in an urban slum	Outcome
Bhandari 2007	Adding zinc to supplemental iron and folic acid does not affect mortality and severe morbidity in young children	Outcome
Bhatia and Seshadri 1992	Growth performance in anemia and following iron supplementation	Population
Birch 1998	Visual acuity and the essentiality of docosahexaenoic acid and arachidonic acid in the diet of term infants	Population
Birch 2000	A randomized controlled trial of early dietary supply of LCP and mental development in term infants.	Population
Birch 2002	A randomized controlled trial of long-chain polyunsaturated fatty acid supplementation of formula in term infants after weaning at 6 wk of age	Population
Birungi 2015	Effect of breastfeeding promotion on early childhood caries and breastfeeding duration among 5 year old children in eastern uganda: A cluster randomized trial	Outcome
Black 2004B	Cognitive and motor development among small-for-gestational-age infants: impact of zinc supplementation, birth weight, and caregiving practices.	Outcome
Blaymore Bier 1996	Comparison of Skin-to-Skin Contact With Standard Contact in Low-Birth-Weight Infants Who Are Breast-Fed	Outcome
Boisson 2013	Effect of Household-Based Drinking Water Chlorination on Diarrhoea Among Children Under Five in Orissa, India: a Double-Blind Randomised Placebo-Controlled Trial	Population
Bonjour 1997	Calcium-enriched foods and bone mass growth in prepuber- tal girls: a randomized, double-blind, placebo-controlled trial	Other
Boo 2001	Contamination of breast milk obtained by manual expression and breast pumps in mothers of very low birthweight infants	Outcome
Boo 2007	Short duration of skin-to-skin contact: Effects on growth and breastfeeding	Study design
Borg B 2017	Efficacy of a locally-produced multiple micronutrient-fortified ready-to-use supplementary food (rusf) for children under two years in cambodia	Outcome
Bouckaert K 2017	Effect of multiple micronutrient supplementation in lactating women on infant growth and morbidity: A double-blind randomized controlled trial in rural burkina faso	Other

© 2019 Park JJH et al. *JAMA Network Open*.

First author, year	Title	Reason for exclusion
Bougma K 2015	Iodized salt improves child's iodine status, mental development, and physical growth in a cluster randomized trial in ethiopia	Other
Boutte 1985	Comparison of hand and electric operated breast pumps.	Outcome
Briceno 2017	Are there synergies from combining hygiene and sanitation promotion campaigns: Evidence from a large-scale cluster-randomized trial in rural tanzania	Population
Brooks 2005	Effect of weekly zinc supplements on incidence of pneumonia and diarrhoea in children younger than 2 years in an urban, low-income population in Bangladesh:randomised controlled trial	Outcome
Brown 1991	Complementary Feeding of Young children in Developing Countries: a Review of Current Scientific Knowledge	Study design
Brown 1991	Clinical trial of modified whole milk, lactose-hydrolyzed whole milk, or cereal-milk mixtures for the dietary management of acute childhood diarrhea	Outcome
Brown 2015	Acceptability of two ready-to-use therapeutic foods by hiv-positive patients in vietnam	Population
Burton 2013	Randomized trial comparing the effectiveness of two electric breast pumps in the NICU	Outcome
Bystrova 2003	Skin- to-skin contact may reduce negative consequences of “the stress of being born”: a study on temperature in newborn infants, subjected to different ward routines in St. Petersburg	Outcome
Cameron 2004	The effect of calcium supplementation on bone density in premenarcheal females: a co-twin approach	Population
Cameron Sl 2015	Lactation consultant support from late pregnancy with an educational intervention at 4 months of age delays the introduction of complementary foods in a randomized controlled trial	Outcome
Campbell 2016	Effect of complementary food supplementation on breastfeeding and home diet in rural bangladeshi children	Outcome
Campbell 2016a	Water, sanitation and hygiene related risk factors for soil-transmitted helminth and giardia duodenalis infections in rural communities in timor-leste	Study design
Campbell 2017a	Investigations into the association between soil-transmitted helminth infections, haemoglobin and child development indices in manufahi district, timor-leste	Study design
Campbell 2017b	Water, sanitation and hygiene (wash) and environmental risk factors for soil-transmitted helminth intensity of infection in timor-leste, using real time pcr	Study design
Campbell R 2015	Children receiving complementary food supplements (cfs) have higher nutrient intakes from home foods in jivita-4 trial rural bangladesh	Outcome
Carfoot 2004	The value of a pilot study in breast-feeding research	Outcome
Carfoot 2005	A randomised controlled trial in the north of England examining the effects of skin-to-skin care on breast feeding	Outcome
Carlsen 2013	Telephone-based support prolongs breastfeeding duration in obese women: a randomized trial	Outcome
Carlson 1996	A randomized trial of visual attention of preterm infants fed docosahexaenoic acid until two months	Outcome
Carlson 1997	Functional effects of increasing omega-3 fatty acid intake	Outcome
Carlsson 1978	Effects of amount of contact between mother and child on the mother’s nursing behavior	Outcome
Carter Rc 2015	Effects of zinc and multivitamin supplementation on hematologic status during infancy	Outcome
Castillo 1996	Breast-feeding and the Nutritional Status of Nursing Children in Chile	Study design
Castillo-Durán 1994	Zinc supplementation increases growth velocity of male children and adolescents with short stature	Population
Cattaneo 1998	Kangaroo mother care for low birthweight infants: a randomized controlled trial in different settings	Outcome
Cavan 1993	Growth and body composition of periurban Guate- malan children in relation to zinc status: a longitudinal zinc intervention trial	Population
Cha 2015	The effect of improved water supply on diarrhea prevalence of children under five in the volta region of ghana: A cluster-randomized controlled trial	Outcome
Chang 2010	Supplementing iron and zinc: Double blind, randomized evaluation of separate or combined delivery	Outcome
Charpak 1997	A randomized, controlled trial of kangaroo mother care: results of follow-up at 1 year of corrected age.	Population
Charpak 2001	A Randomized, Controlled Trial of Kangaroo Mother Care: Results of Follow-Up at 1 Year of Corrected Age	Outcome
Chen 2012	Effects of vitamin A, vitamin A plus zinc, and multiple micronutrients on anemia in preschool children in Chongqing, China	Population

First author, year	Title	Reason for exclusion
Cheng 1993	Impact of large-dose vitamin A supplementation on childhood diarrhoea, respiratory disease and growth	Other
Cheung 2016	Gut microbiota in malawian infants in a nutritional supplementation trial	Outcome
Chevalley 2005	Skeletal site selectivity in the effects of calcium supplementation on areal bone mineral density gain: a randomized, double-blind, placebo-controlled trial in prepubertal boys	Other
Chew 1993	Is dilution of cows' milk formula necessary for dietary management of acute diarrhoea in infants aged less than 6 months?	Outcome
Chhagan 2009	Effect of micronutrient supplementation on diarrhoeal disease among stunted children in rural South Africa	Outcome
Chhagan 2010	Effect on longitudinal growth and anemia of zinc or multiple micronutrients added to vitamin a: A randomized controlled trial in children aged 6-24 months	Population
Chi Luong 2016	Newly born low birthweight infants stabilise better in skin-to-skin contact than when separated from their mothers: A randomised controlled trial	Outcome
Christensson 1992	Temperature, metabolic adaptation and crying in healthy full-term newborns cared for skin-to-skin or in a cot	Outcome
Christensson 1995	Separation distress call in the human neonate in the absence of maternal body contact	Outcome
Christian 2003A	Effects of alternative maternal micronutrient supplements on low birth weight in rural Nepal: double blind randomised community trial	Population
Christian 2003B	Effects of maternal micronutrient supplementation on fetal loss and infant mortality: a cluster-randomized trial in Nepal.	Population
Christian 2013	Effects of vitamin A and beta-carotene supplementation on birth size and length of gestation in rural Bangladesh: a cluster-randomized trial	Population
Christian 2016	Effects of prenatal multiple micronutrient supplementation on growth and cognition through 2 y of age in rural bangladesh: The jivita-3 trial	Population
Chwang 1988	Iron supplementation and physical growth of rural Indonesian children	Population
Chwo 1999	Early kangaroo care for 34-36 week preterm infants: effects on temperature, weight, cortisol, and behavior [dissertation]	Outcome
Ciliberto 2005	Comparison of home-based therapy with ready-to-use therapeutic food with standard therapy in the treatment of malnourished malawian children: A controlled, clinical effectiveness trial	Population
Clarke Se 2015	Seasonal malaria chemoprevention combined with micronutrient supplementation delivered through community preschools: Findings from a cluster randomized trial in mali	Other
Clarke Se 2016	Impact of micronutrient supplementation combined with malaria chemoprevention on malaria, anaemia and cognitive development in early childhood: Findings from a cluster randomized study in southern mali	Other
Clasen 2012	The Effect of Improved Rural Sanitation on Diarrhoea and Helminth Infection: Design of a Cluster- randomized Trial in Orissa, India	Study design
Clasen T 2014	The effectiveness of a rural sanitation intervention on health and orissa, india: A clusterrandomized, controlled trial	Population
Cohen 1994	Determinants of Growth From Birth to 12 Months Amon Breast-Fed Honduran Infants in Relation to Age of Introduction of Comlementary Foods	Study design
Coles 2015	Dose and timing of prenatal alcohol exposure and maternal nutritional supplements: Developmental effects on 6-month-old infants	Outcome
Correia-Santos 2011	Dietary supplements for the lactating adolescent mother: influence on plasma micronutrients [Suplementos dieteticos opara la madre adolescente en periodo de lactancia: su influencia en micronutrirnetes del plasma]	Population
Costa 1989	A comparison of colony counts of breast milk using two methods of breast cleansing	Outcome
Courteix 2005	Cumulative effects of calcium supplementation and physical activity on bone accretion in premenarchal children: a double-blind randomised placebo-controlled trial	Population
Craig 1982	The effect of early contact on maternal perception of infant behavior	Other
Cruz Rj 2016	Randomized controlled trialon the effect of 10 grams moringa oleifera powder leaves on the level of hemoglobin and hematocrit on infants age 6-9 months	Other
Curry 1982	The effect of skin-to-skin contact between mother and infant during the first hour following delivery on the mother's maternal attachment behavior and self concept [dissertation]	Outcome
da Cunha 2016	Breast milk supplementation and preterm infant development after hospital discharge: A randomized clinical trial	Outcome
Daly Williams 1999	Iron supplemented formula milk related to reduction in psychomotor decline in infants from inner city areas: randomised study	Outcome

© 2019 Park JJH et al. *JAMA Network Open*.

First author, year	Title	Reason for exclusion
Dandamrongrak 2016	Correlation between iodine supplement in pregnancy and neonatal tsh level	Outcome
de Almeida 2005	Effect of fortification of drinking water with iron plus ascorbic acid or with ascorbic acid alone on hemoglobin values and anthropometric indicators in preschool children in day-care centers in southeast brazil	Population
De Carvalho 1985	Frequency of milk expression and milk production by mothers of nonnursing premature neonates	Outcome
De Chateau 1977	Long-term effect on mother- infant behaviour of extra contact during the first hour post partum. II. A follow-up at three months	Outcome
De Moura 2015	Cassava intake and vitamin a status among women and preschool children in akwa-ibom, nigeria	Population
de Paula 2001	The use of sugar fortified with iron tris-glycinate chelate in the prevention of iron deficiency anemia in preschool children	Outcome
Dehghani 2015	A randomized controlled trial of kangaroo mother care versus conventional method on vital signs and arterial oxygen saturation rate in newborns who were hospitalized in neonatal intensive care unit	Outcome
Desai 2015	The shine trial infant feeding intervention: Pilot study of effects on maternal learning and infant diet quality in rural zimbabwe	Outcome
Dewey 1997	A randomized intervention study of the effects of discontinuing coffee intake on growth and morbidity of iron-deficient guatemalan toddlers	Population
Dewey 1999	Age of introduction of complementary foods and growth of term, low-birth-weight, breast-fed infants: a randomized intervention study in Honduras	Study design
Dewey 1999A	Age of introduction of complementary foods and growth of term, low-birth-weight, breast-fed infants: a randomized intervention study in Honduras.	Population
Diaz-gomez 2003	The effect of zinc supplementation on linear growth, body composition, and growth factors in preterm infants	Population
Dibba 2000	Effect of calcium supplementation on bone mineral accretion in Gambian children accustomed to a low-calcium diet	Population
Domellof 2001	Iron requirements of term, breast-fed infants: a study in Sweden and Honduras.	Population
Dossa 2001	Multivitamin-multimineral and iron supplementation did not improve appetite of young stunted and anemic beninese children	Population
Dossa 2002	Effects of multivitamin-multimineral supplementation on appetite of stunted young beninese children	Population
du Preez 2010	Solar disinfection of drinking water in the prevention of dysentery in South African children aged under 5 years: the role of participant motivation	Outcome
du Preez 2011	Randomized intervention study of solar disinfection of drinking water in the prevention of dysentery in kenyan children aged under 5 years	Population
Duffy 1997	Positive effects of an antenatal group teaching session on postnatal nipple pain, nipple trauma and breast feeding rates	Outcome
Duijts 2010	Prolonged<br>and exclusive breastfeeding reduces the risk of infectious<br>diseases in infancy	Study design
Duncan 1993	Exclusive breast-feeding for at least 4 months protects against otitis media	Study design
Edraki M 2015	Comparison of the effects of attachment training for mothers on the behavioral responses of premature infants: A randomized clinical trial	Outcome
Eka Pratiwi 2009	Effect of kangaroo method on the risk of hypothermia and duration of birth weight regain in low birth weight infants: A randomized controlled trial	Outcome
Ekbote 2011	A pilot randomized controlled trial of oral calcium and vitamin D supplementation using fortified laddoos in underprivileged Indian toddlers	Population
Ekcharoen 2015	Comparing growth rates after hospital discharge of preterm infants fed with either post-discharge formula or high-protein, medium-chain triglyceride containing formula	Study design
El Frargy 2017	Zinc supplementation as an adjuvant treatment in neonatal sepsis	Outcome
El-Farghali 2015	Early zinc supplementation and enhanced growth of the low-birth weight neonate	Outcome
Engebretsen 2014	Growth effects of exclusive breastfeeding promotion by peer counsellors in sub-Saharan Africa: the cluster-randomised PROMISE EBF trial	Outcome
Engstrom 2008	Effectiveness of daily and weekly iron supplementation in the prevention of anemia in infants.	Other
Ercumen 2015	Effects of source- versus household contamination of tubewell water on child diarrhea in rural bangladesh: A randomized controlled trial	Outcome
Ermis 2002	Effects of three different iron supplementations in term healthy infants after 5 months of life.	Other
Evangelista-Salazar 2004	Evaluation of the preventive effect of the intermittent provision of iron and vitamin C on the reduction of the iron and neurodevelopment in infants	Other
Evelein 2011	The association between breastfeeding and the cardiovascular system in early childhood.	Study design

© 2019 Park JJH et al. *JAMA Network Open*.

First author, year	Title	Reason for exclusion
Fabiansen 2017	Effectiveness of food supplements in increasing fat-free tissue accretion in children with moderate acute malnutrition: A randomised 2 x 2 x 3 factorial trial in burkina faso	Outcome
Fadnes 2016	Effects of an exclusive breastfeeding intervention for six months on growth patterns of 4-5 year old children in uganda: The cluster-randomised promise ebf trial	Population
Fahmida 2007	Zinc-iron, but not zinc-alone supplementation, increased linear growth of stunted infants with low haemoglobin.	Other
Fallahrokni A 2017	Zinc supplementation is an effective and feasible strategy to prevent growth retardation in 6 to 24 month children: A pragmatic randomized double blind trial	Other
Faqih 2006	Effectiveness of intermittent iron treatment of two- to six-year-old Jordanian children with iron-deficiency anemia	Population
Fardig 1980	A comparison of skin-to-skin contact and radiant heaters in promoting neonatal thermoregulation	Outcome
Fawzi 1997	Dietary vitamin a intake in relation to child growth	Study design
Fayad 1999	Comparison of soy-based formulas with lactose and with sucrose in the treatment of acute diarrhea in infants	Outcome
Feher 1989	Increasing breast<br>milk production for premature infants with a relaxation/<br>imagery audiotape	Outcome
Feliciano 1994	Seasonal and geographical variations in the growth rate of infants in china receiving increasing dosages of vitamin d supplements	Population
Ferber 2004	The effect of skin-to-skin contact (kangaroo care) shortly after birth on the neurobehavioral responses of the term newborn: a randomized, controlled trial	Outcome
Fernandeez-Rao S 2015	Multiple micronutrients and early learning interventions promote infant micronutrient status and development	Other
Fewtrell 2001a	Randomized study comparing the efficacy of a novel manual breast pump with a mini-electric breast pump in mothers of term infants	Outcome
Fewtrell 2001b	Randomized trial comparing the efficacy of a novel manual breast pump with a standard electric breast pump in mothers who delivered preterm infants	Outcome
Finch 2002	Breastfeeding education program with incentives increases exclusive breastfeeding among urban WIC participants	Outcome
Fischer Walker 2006	Zinc supplementation for the treatment of diarrhea in infants in pakistan, india and ethiopia	Outcome
Flaherman 2012	Randomised trial comparing hand expression with breast pumping for mothers of term newborns feeding poorly	Outcome
Flax 2013	Growth and micronutrient status of hiv-exposed infants in the breastfeeding, antiretrovirals, and nutrition study during the first year of life	Outcome
Flax 2014	Integrating group counseling, cell phone messaging, and participant-generated songs and dramas into a microcredit program increases Nigerian women's adherence to international breastfeeding recommendations	Outcome
Flax 2015	Provision of lipid-based nutrient supplements to honduran children increases their dietary macro- and micronutrient intake without displacing other foods	Population
Forster 2004	Two mid-pregnancy interventions to increase the initiation and duration of breastfeeding: a randomized controlled trial.	Outcome
Francis 2008	Maternal/infant lactation characteristics (MILC) study: a comparison of single electric pumping devices	Outcome
Freeman 2016	The impact of a rural sanitation programme on safe disposal of child faeces: A cluster randomised trial in odisha, india	Outcome
Friedrich 2018	Enhancing handwashing frequency and technique of primary caregivers in harare, zimbabwe: A cluster-randomized controlled trial using behavioral and microbial outcomes	Outcome
Friel 1993	Zinc supplemetation in very-low-birth-weight infants	Population
Friis 1997	The impact of zinc supplementation on growth and body composition: a randomized, controlled trial among rural Zimbabwean schoolchildren	Population
Furuhjelm 2009	Fish oil supplementation in pregnancy and lactation may decrease the risk of infant allergy	Population
Garate-Gallardo 2015	Comparing different maize supplementation strategies to improve resilience and resistance against gastrointestinal nematode infections in browsing goats	Outcome
Gardner 1998	Zinc supplementation: effects on the growth and morbidity of undernourished Jamaican children	Outcome
Gardner 2005	Zinc supplementation and psychosocial stimulation: effects on the development of undernourished Jamaican children	Population
Gargano 2018	Efficacy and tolerability outcomes of a phase ii, randomized, open-label, multicenter study of a new water-dispersible pediatric formulation of dihydroartemisinin-piperaquine for the treatment of uncomplicated plasmodium falciparum malaria in	Study design

© 2019 Park JJH et al. *JAMA Network Open*.

First author, year	Title	Reason for exclusion
	african infants	
Garmendia 2015	Effectiveness of a normative nutrition intervention (diet, physical activity and breastfeeding) on maternal nutrition and offspring growth: The chilean maternal and infant nutrition cohort study (chimincs)	Population
Garza 1982	Effects of methods of collection and storage on nutrients in human milk	Outcome
Gathwala 2008	KMC facilitates mother baby attachment in low birth weight infants.	Outcome
Gathwala 2010	KMC Facilitates Mother Baby Attachment in Low Birth Weight Infants	Population
Gavhane 2016	Long term outcomes of kangaroo mother care in very low birth weight infants	Outcome
Habib 2015	Zinc supplementation fails to increase the immunogenicity of oral poliovirus vaccine: A randomized controlled trial	Population
Gibson 1989	A growth-limiting, mild zinc-deficiency syndrome in some southern Ontario boys with low height percentiles	Other
Gibson 1997A	Effect of increasing breast milk docosahexaenoic acid on plasma and erythrocyte phospholipid fatty acids and neural indices of exclusively breast-fed infants	Population
Gibson 1998	The effects of diets rich in docosahexaenoic acid and/or gamma-linolenic acid on plasma fatty acid profiles in term infants	Study design
Gibson 2009	Safety of supplementing infant formula with long-chain polyunsaturated fatty acids and Bifidobacterium lactis in term infants: a randomised controlled trial	Study design
Gill 1997	Effect of fortification of drinking water with iron plus ascorbic acid or with ascorbic acid alone on hemoglobin values and anthropometric indicators in preschool children in day-care centers in Southeast Brazil	Outcome
Girish 2013	Impact and feasibility of breast crawl in a tertiary care hospital	Outcome
Glinz 2015	The effect of iron-fortified complementary food and intermittent preventive treatment of malaria on anaemia in 12- to 36-month-old children: A cluster-randomised controlled trial	Population
Glinz 2017	Iron fortified complementary foods containing a mixture of sodium iron edta with either ferrous fumarate or ferric pyrophosphate reduce iron deficiency anemia in 12- to 36-month-old children in a malaria endemic setting: A secondary analysis of a cluster-randomized controlled trial	Outcome
Gouchon 2010	Skin-to-skin contact after cesarean delivery: an experimental study	Outcome
Goudarzvand 2017	Comparison of conventional phototherapy and phototherapy along with kangaroo mother care on cutaneous bilirubin of neonates with physiological jaundice	Outcome
Grange 1994	Evaluation of a maize-cowpea-palm oil diet for the dietary management of Nigerian children with acute, watery diarrhea	Outcome
Grantham-McGregor 1993	The effect of nutritional supplementation and stunting on morbidity in young children: The jamaican study	Population
Greenland 2016	Multiple behaviour change intervention for diarrhoea control in lusaka, zambia: A cluster randomised trial	Population
Grillenberger 2003	Food supplements have a positive impact on weight gain and the addition of animal source foods increases lean body mass of Kenyan schoolchildren	Population
Groh-Wargo 1995	The utility of a bilateral breast pumping system for mothers of premature infants	Outcome
Guldan 2000	Culturally appropriate nutrition education improves infant feeding and growth in rural Sichuan, China.	Outcome
Gupta 1982	Effect of periodic antiascaris and antigiardia treatment on nutritional status of preschool children	Population
Gupta 2016	Vitamin d supplementation for treatment and prevention of pneumonia in under-five children: A randomized double-blind placebo controlled trial	Population
Gupta 2017	Complementary feeding at 4 versus 6 months of age for preterm infants born at less than 34 weeks of gestation: A randomised, open-label, multicentre trial	Outcome
Guzman 1958	Growth and development of Central American children: growth responses of rural Guatemalan school children to daily administration of penicillin and aureomycin	Population
Haber Ja 2016	The effect of maternal supplementation with a lipid-based nutrient supplement on infant micronutrient intake in guatemalan women and infants	Study design
Haber Ja 2016A	The short-term response of breast milk micronutrient concentrations to a lipid-based nutrient supplement in guatemalan women	Outcome
Hamadani 2001	Randomized controlled trial of the effect of zinc supplementation on the mental development of bangladeshi infants	Population
Hadi 1999	Respiratory infections reduce the growth response to vitamin a supplementation in a randomized controlled trial	Outcome
Hadi 2000	Vitamin a supplementation selectively improves the linear growth of indonesian	Outcome

© 2019 Park JJH et al. *JAMA Network Open*.

First author, year	Title	Reason for exclusion
	preschool children: Results from a randomized controlled trial	
Hadi 2004	Complex interactions with infection and diet may explain seasonal growth responses to vitamin a in preschool aged indonesian children	Outcome
Hales 1977	Defining the limits of the maternal sensitive period	Outcome
Hall 2002	A randomised trial in Mali of the effectiveness of weekly iron supplements given by teachers on the haemoglobin concentrations of schoolchildren	Population
Hall 2007	An evaluation of the impact of a school nutrition programme in Vietnam	Study design
Kumbhojkar 2016	Kangaroo Mother Care (KMC): An Alternative to Conventional Method of Care for Low Birth Weight Babies	Study design
Harding 2017	Adherence to recommendations on lipid-based nutrient supplement and iron and folic acid tablet consumption among pregnant and lactating women participating in a community health programme in northwest bangladesh	Outcome
Hartinger 2016	Improving household air, drinking water and hygiene in rural peru: A community-randomized-controlled trial of an integrated environmental home-based intervention package to improve child health	Outcome
Hartinger Sm 2012	A community randomized controlled trial of an integrated home-based intervention improving household-air pollution, drinking water quality and hygiene in rural peru	Population
Haschke 1988	Iron intake and iron nutritional status of infants fed	Population
Hauner 2009	The impact of nutritional fatty acids during pregnancy and lactation on early human adipose tissue development	Population
Heikens 1989	The kingston project. I. Growth of malnourished children during rehabilitation in the community, given a high energy supplement	Other
Heikens 1993	The kingston project. Ii. The effects of high energy supplement and metronidazole on malnourished children rehabilitated in the community: Anthropometry	Other
Heinig 1993	Intake and growth of breast-fed and formula- fed infants in relation to the timing of introduction<br>of complementary foods: the DARLING study	Study design
Heinig 2006	Zinc supplementation does not affect growth, morbidity, or motor development of US term breastfed infants at 4–10 mo of age	Population
Helland 2001	Similar effects on infants of n-3 and n-6 fatty acids supplementation to pregnant and lactating women	Population
Hemsworth 2016	Lipid-based nutrient supplements increase energy and macronutrient intakes from complementary food among malawian infants	Outcome
Heon 2011	Nursing Interventions Related to Breastfeeding in Preterm Infants	Outcome
Hess 2015	Iodine status of young burkinabe children receiving small-quantity lipid-based nutrient supplements and iodised salt: A cluster-randomised trial	Outcome
Hettiarachchi 2008	The efficacy of micronutrient supplementation in reducing the prevalence of anaemia and deficiencies of zinc and iron among adolescents in Sri Lanka	Population
Hill 1999	Breastfeeding experience and milk weight in lactating mothers pumping for preterm infants	Outcome
Hollis 2004	Vitamin D requirements during lac- tation: high-dose maternal supplementation as therapy to prevent hypovitaminosis D for both the mother and the nurs-<br>ing infant	Other
Holscher 2012	Bifidobacterium lactis Bb12 Enhances Intestinal Antibody Response in Formula-Fed Infants: A Randomized, Double-Blind, Controlled Trial	Population
Hong 1992	Growth promoting effect of zinc supplementation in infants of high-risk pregnancies.	Other
Hopkinson 2009	Maternal response to two electric breast pumps	Outcome
Hossain 2005	Increased food intake after the addition of amylase-rich flour to supplementary food for malnourished children in rural communities of bangladesh	Outcome
Hossain Mdi 2016	Home-based care with follow-up at outpatient unit or community-follow-up center with/without food supplementation and/or psychosocial stimulation of children with moderate acute malnutrition in bangladesh	Outcome
Huang 2006	Effect of very early kangaroo care on extrauterine temperature adaptation in newborn infants with hypothermia problem	Outcome
Huey S 2017	Pre-intervention characterization of nutritional status to estimate burden and potential to benefit among mothers and their children living in urban slums of mumbai, india	Study design
Huffman 1987	Nutrition and fertility in Bangladesh: breastfeeding and post partum amenorrhea	Study design
Husaini 1991	Developmental effects of short-term supplementary feeding in nutritionally-at-risk Indonesian infants.	Outcome
Husaini 1996	The effects of high energy and micronutrient supplementation on iron status in nutritionally at risk infants	Other

© 2019 Park JJH et al. *JAMA Network Open*.

First author, year	Title	Reason for exclusion
Huybregts 2012	The effect of adding ready-to-use supplementary food to a general food distribution on child nutritional status and morbidity: A cluster-randomized controlled trial	Population
Huybregts 2017	The impact of integrated prevention and treatment on child malnutrition and health: The promis project, a randomized control trial in burkina faso and mali	Outcome
Huynh Dtt 2017	Maternal nutritional adequacy and gestational weight gain in vietnamese pregnant women	Other
Hyder 2007	A multiple-micronutrient- fortified beverage affects hemoglobin, iron, and vitamin A status and growth in adolescent girls in rural Bangladesh	Population
Iannotti 2015	Early growth velocities and weight gain plasticity improve linear growth in peruvian infants	Outcome
Iannotti 2017	Eggs early in complementary feeding increase choline pathway biomarkers and dha: A randomized controlled trial in ecuador	Outcome
Ihab A 2014	The Impact of Animal Source food (ASF) on the Growth of Malnourished Children in Bachok, Kelantan: Randomized Controlled Intervention Trial	Outcome
Ijumba 2015	Effect of an integrated community-based package for maternal and newborn care on feeding patterns during the first 12 weeks of life: A cluster-randomized trial in a south african township	Outcome
Inayati 2012	Combined intensive nutrition education and micronutrient powder supplementation improved nutritional status of mildly wasted children on nias island, indonesia	Population
Innis 1996b	Blood lipid docosahexaenoic and arachidonic acid in term gestation infants fed formulas with high docosa- hexaenoic acid, low eicosapentaenoic acid fish oil	Population
Innis 1997	Visual acuity and blood lipids in term infants fed human milk or formulae	Population
Islam 2018	Study protocol for a randomized, double-blind, community-based efficacy trial of various doses of zinc in micronutrient powders or tablets in young bangladeshi children	Other
Islam Khan 2013	Effects of pre- and postnatal nutrition interventions on child growth and body composition: The minimat trial in rural bangladesh	Population
Iuliano-Burns 2003	Regional specificity of exercise and calcium during skeletal growth in girls: a randomized controlled trial	Other
Jaeggi 2015	Iron fortification adversely affects the gut microbiome, increases pathogen abundance and induces intestinal inflammation in kenyan infants	Outcome
Jakobsen 2008	Promotion of exclusive breastfeeding is not likely to be cost effective in West Africa. A randomized intervention study from Guinea-Bissau	Outcome
Jannat Kke 2015	Observed handwashing behavior during infant feeding, intervention assessment of a large randomized controlled trial (rct) in rural bangladesh	Other
Jayamala 2015	Impact of music therapy on breast milk secretion in mothers of premature newborns	Outcome
Jensen 1997	Effect of dietary linoleic/alpha-linolenic acid ratio on growth and visual function of term infants	Population
Jensen 1999	Effects of maternal docosahexaenoic acid supplementation on visual function and growth of breast-fed term infants.	Population
Jensen 2005	Effects of maternal docosahexaenoic acid intake on visual function and neurodevelopment<br>in breastfed term infants	Population
Johnson W 2016	Pre-pregnancy energy balance, gestational weight gain, and small-for-gestational age in rural gambia: The early nutrition and immune development trial (isrctn49285450)	Outcome
Jones 2001	A randomised<br>controlled trial to compare methods of milk expression after preterm delivery	Outcome
Jordan I 2015	Food based nutrition education improved complementary feeding practices and nutritional status of children below 2 years in malawi	Outcome
Jorgensen 1998	Effect of formula supplemented with docosahexaenoic acid and gamma- linolenic acid on fatty acid status and visual acuity in term infants. J. Pediatr.	Outcome
Jorgensen 2017	Lipid-based nutrient supplements during pregnancy and lactation did not affect human milk oligosaccharides and bioactive proteins in a randomized trial	Outcome
Joseph 2016	Adverse events from a randomized, multi-arm, placebo-controlled trial of mebendazole in children 12-24 months of age	Outcome
Joseph Sa 2013	Malnutrition and the critical growth window in an sthendemic area of peru: Worm infections a barrier to achieving child-related mdgs?	Other
Juliana K 2017	Bioequivalence of micronutrient powders to conventional fortification on serum zinc levels of moderately malnourished children in thika informal settlements, kenya	Outcome
Kabir 1998	Increased height gain of children fed a high-protein diet during convalescence from shigellosis: a six-month follow-up study	Outcome
Kadam 2005	Feasibility of Kangaroo Mother Care in Mumbai	Outcome
Kaestel 2005	Effects of prenatal multimicronutrient supplements on birth weight and perinatal mortality: a randomised, controlled trial in Guinea-Bissau	Population

© 2019 Park JJH et al. *JAMA Network Open*.

First author, year	Title	Reason for exclusion
Kajosaari 1983	Atopy prevention in childhood: the role of diet: prospective 5-year follow-up of high-risk infants with six months exclusive breastfeeding and solid food elimination	Study design
Kamm 2016	Is pregnancy a teachable moment to promote handwashing with soap among primiparous women in rural bangladesh? Follow-up of a randomised controlled trial	Outcome
Kang Y 2015	Effectiveness of a communitybased participatory nutrition promotion program to improve linear and ponderal growth in children 6 to 24 months of age in rural eastern ethiopia: A cluster randomized trial	Other
Kaplowitz 1983	The effect of an educational program on the decision to breastfeed	Outcome
Karanja 2010	The TOTS community intervention to prevent 117. overweight in American Indian toddlers: a feasibility and efficacy study	Population
Karimi A 2015	The effect of an interventional program based on the theory of ethology on infant breastfeeding competence	Outcome
Kaseb 2013	Efficacy of zinc supplementation on improvement of weight and height growth of healthy 9–18 year children in Iran	Population
Kastner 2005	Mother-child relationship before, during and after birth	Outcome
Keikhaei 2007	Iron-deficiency anemia among children in southwest iran	Study design
Keith 2012	The effect of music- based listening interventions on the volume, fat content, and caloric content of breast milk-produced by mothers of premature and critically ill infants	Outcome
Kellams 2016	The impact of a prenatal education video on rates of breastfeeding initiation and exclusivity during the newborn hospital stay in a low-income population	Outcome
Khademloo 2009	Comparison of the effectiveness of weekly and daily iron supplementation in 6- to 24-months-old babies in urban health centers of Sari, Iran.	Outcome
Khadivzadeh 2004	Effect of exclusive breastfeeding and complementary feeding on infant growth and morbidity	Study design
Khadivzadeh 2009	The effect of mother- infant skin to skin contact on mother's attachment.	Outcome
Khan 1984	Breastfeeding, growth and diarrhoea in rural Bangladesh children	Population
Khan 2013	Effects of exclusive breastfeeding intervention on child growth and body composition: the MINIMat trial, Bangladesh	Population
Kikafunda 1998	Effect of zinc sup- plementation on growth and body composition of Ugandan preschool children: a randomized, controlled, intervention trial	Population
Kim S 2017A	Exposure to Large-Scale Social and Behavior Change Communication Interventions Is Associated with Improvements in Infant and Young Child Feeding Practices in Ethiopia	Other
Kim S 2017B	Two-year sustained impacts of largescale social and behavior change communication interventions to improve infant and young child feeding in bangladesh	Outcome
Kimani-Murage 2017	Effectiveness of home-based nutritional counselling and support on exclusive breastfeeding in urban poor settings in nairobi: A cluster randomized controlled trial	Outcome
Kirkwood 1996	Effect of vitamin a supplementation on the growth of young children in northern ghana	Outcome
Kistin 1990	Breast-feeding rates among black urban low-income women: effect of prenatal education	Outcome
Klevor 2016	Lipid-based nutrient supplements providing approximately the recommended daily intake of vitamin a do not increase breast milk retinol concentrations among ghanaian women	Outcome
Kluka 2004	A Randomized Controlled Trial to Test the Effect of an Antenatal Educational Intervention on Breastfeeding Duration Among Primiparous Women [thesis]	Outcome
Konyole S 2017	Effect on lean mass, linear growth and iron status of improved animal source foods and micronutrients fortified complementary foods among kenyan young children: A randomized controlled trial	Other
Koo ww 2006	Posthospital discharge feeding for preterm infants: effects of standard compared with enriched milk formula on growth, bone mass, and body composition	Population
Kools 2005	A breast-feeding promotion and support program a randomized trial in The Netherlands	Outcome
Kordas 2009	The effects of iron and/or zinc supplementation on maternal reports of sleep in infants from nepal and zanzibar	Outcome
Kounnvaong 2011	Effect of daily versus weekly home fortification with multiple micronutrient powder on haemoglobin concentration of young children in a rural area, Lao People's Democratic Republic: a randomised trial.	Outcome
Kramer 2000	Short and Long Term Effects of Breast Feeding on Child Health	Outcome

© 2019 Park JJH et al. *JAMA Network Open*.

First author, year	Title	Reason for exclusion
Kramer 2007	Effects of prolonged and exclusive breastfeeding on child height, weight, adiposity, and blood pressure at age 6.5 y: evidence from a large randomized trial	Outcome
Krebs 2011	Complementary feeding: A global network cluster randomized controlled trial	Other
Kronborg 2012	Antenatal training to improve breast feeding: a randomised trial	Outcome
Kumar 2017	Supplementation of vitamin b12 or folic acid on hemoglobin concentration in children 6-36 months of age: A randomized placebo controlled trial	Outcome
Kumar T 2016	Supplementation of vitamin b12 or folic acid on hemoglobin concentration in children 6-36 months of age: A randomized placebo controlled trial	Outcome
Le Roux 2013	Outcomes of home visits for pregnant mothers and their infants: a cluster randomized controlled trial	Outcome
Kvestad 2015	Vitamin b12 and folic acid improve gross motor and problem-solving skills in young north indian children: A randomized placebo-controlled trial	Outcome
Lagrone 2010	Locally produced ready-to-use supplementary food is an effective treatment of moderate acute malnutrition in an operational setting	Population
LaGrone 2012	A novel fortified blended flour, corn-soy blend "plus-plus," is not inferior to lipid-based ready-to-use supplementary foods for the treatment of moderate acute malnutrition in malawian children	Outcome
Lampl 1978	The effects of protein supple- mentation on the growth and skeletal maturation of new Guinean school children	Population
Lamy Filho 2015	Effect of maternal skin-to-skin contact on decolonization of methicillin-oxacillin-resistant staphylococcus in neonatal intensive care units: A randomized controlled trial	Outcome
Langlois B 2017	Changes in household food insecurity between enrollment and exit from a blanket supplementary feeding program for children 6 - 23 months old in burkina faso	Other
Larnkjær 2014	Effect of milk proteins on linear growth and IGF variables in overweight adolescents	Population
Larson 2010	The added benefit of zinc supplementation after zinc treatment of acute childhood diarrhoea: a randomized, double-blind field trial	Outcome
Larson L 2017	Relation between diet, nutritional status, and stimulation and child development: A path analysis	Study design
Latham 1990	Improvements in growth following iron supplementation in young Kenyan school children	Other
Lauritzen 2004	Maternal fish oil supplementation in lactation: effect on visual acuity and n- 3 fatty acid content of infant erythrocytes	Outcome
Lavender 2005	Breastfeeding expectations versus reality: a cluster randomised controlled trial	Outcome
Lawless 1994	Iron supplementation improves appetite and growth in anemic Kenyan primary school children	Population
Lazarus 2018	The effect of probiotics and zinc supplementation on the immune response to oral rotavirus vaccine: A randomized, factorial design, placebo-controlled study among indian infants	Outcome
Leroy 2016	Tubaramure, a food-assisted integrated health and nutrition program in burundi, increases maternal and child hemoglobin concentrations and reduces anemia: A theory-based cluster-randomized controlled intervention trial	Outcome
Li 2009	Effects of maternal multimicronutrient supplementation on the mental development of infants in rural western China: follow-up evaluation of a double-blind, randomized, controlled trial	Population
Li 2015	Prenatal micronutrient supplementation is not associated with intellectual development of young school-aged children	Population
Lima 2010	Effects of vitamin a supplementation on intestinal barrier function, growth, total parasitic, and specific giardia spp infections in brazilian children: A prospective randomized, double-blind, placebo-controlled trial	Population
Lima 2014	Effects of glutamine alone or in combination with zinc and vitamin a on growth, intestinal barrier function, stress and satiety-related hormones in brazilian shantytown children	Population
Lin 2009	Effect of beta-carotene supplementation on health and growth of vitamin a deficient children in china rural villages: A randomized controlled trial	Outcome
Lira 1998	Effect of zinc supplementation on the morbidity, immune function, and growth of low-birth-weight, full-term infants in northeast Brazil.	Other
Lira 2017	Effect of rrr-alpha-tocopherol supplementation on serum of breastfeeding women up to 60 days after delivery: A randomised controlled trial	Outcome
Liu 1995	Intermittent iron supplementation in Chinese pre-schoolchildren is efficient and safe	Population
Liu 2016	Reproductive health care and family planning among women in nepal	Outcome
Lloyd 1993	Calcium supplementation and bone mineral density in adolescent girls	Population

© 2019 Park JJH et al. *JAMA Network Open*.

First author, year	Title	Reason for exclusion
Locks L 2015	Effect of zinc & multiple micronutrient supplements on growth in tanzanian children	Population
Locks 2017	The effect of daily zinc and/or multivitamin supplements on early childhood development in tanzania: Results from a randomized controlled trial	Outcome
Lonnerdal 2017	Growth, nutrition, and cytokine response of breast-fed infants and infants fed formula with added bovine osteopontin	Population
Long 2006A	A double-blind, randomized, clinical trial of the effect of vitamin A and zinc supplementation on diarrheal disease and respiratory tract infections in children in Mexico City, Mexico	Outcome
Long 2007	Supplementation with vitamin A reduces watery diarrhoea and respiratory infections in Mexican children.	Outcome
Long 2011	Vitamin a supplementation modifies the association between mucosal innate and adaptive immune responses and resolution of enteric pathogen infections	Outcome
Mridha 2016	Lipid-based nutrient supplements for pregnant women reduce newborn stunting in a cluster-randomized controlled effectiveness trial in Bangladesh	Outcome
Lopriore 2004	Spread fortified with vita- mins and minerals induces catch-up growth and eradicates severe anemia in stunted refugee children aged 3–6 y	Population
Loui 2004	Nutrition of Very Low Birth Weight Infants Fed Human Milk with or without Supplemental Trace Elements: A Randomized Controlled Trial	Outcome
Louzada 2012	Long- term effectiveness of maternal dietary counseling in a low- income population: a randomized field trial	Outcome
Luby 2004	Effect of intensive handwashing promotion on childhood diarrhoea in high-risk communities in Pakistan: a randomised controlled trial	Outcome
Luby 2006	Combining drinking water treatment and hand washing for diarrhoea prevention, a cluster randomised controlled trial	Outcome
Lucas 1996	Randomized outcome trial of human milk fortification and developmental outcome in preterm infants	Population
Lucas 1999	Efficacy and safety of long- chain polyunsaturated fatty acid supplementation of infant-formula milk: A randomised trial	Population
Lucia 2007	Does maternal docosahexaenoic acid supplementation during pregnancy and lactation lower BMI in late infancy?	Population
Lumbanraja 2016	Influence of maternal factors on growth parameters in low-birth-weight babies with kangaroo mother care	Other
Lundeen 2010	Daily use of Sprinkles micronutrient powder for 2 months reduces anemia among children 6 to 36 months of age in the Kyrgyz Republic: a cluster- randomized trial	Outcome
Luong 2015	Newly born low birthweight infants stabilise better in skin-to-skin contact than when separated from their mothers: a randomised controlled trial	Outcome
Luoto 2014	Prebiotic and probiotic supplementation prevents rhinovirus infections in preterm infants: A randomized, placebo-controlled trial	Outcome
Lussier 2015	Daily breastmilk volume in mothers of very low birth weight neonates: A repeated-measures randomized trial of hand expression versus electric breast pump expression	Outcome
Lutter 1989	Nutritional supplementation: Effects on child stunting because of diarrhea	Outcome
Ly 2006	Early shortterm infant food supplementation, maternal weight loss and duration of breast-feeding: a randomised controlled trial in rural Senegal.	Outcome
Ma 2016	The effect of iron fortification on iron (fe) status and inflammation: A randomized controlled trial	Study design
MacArthur 2009	Antenatal peer support workers and initiation of breast feeding: cluster randomised controlled trial	Outcome
Macharia-Mutie 2012	Maize porridge enriched with a micronutrient powder containing low-dose iron as NaFeEDTA but not amaranth grain flour reduces anemia and iron deficiency in Kenyan preschool children	Outcome
Mahdavi 2015	A pilot study of synbiotic supplementation on breast milk mineral concentrations and growth of exclusively breast fed infants	Outcome
Mahmood 2011	Effect of mother-infant early skin-to-skin contact on breastfeeding status: A randomized controlled trial	Other
Makrides 1000b	A critical appraisal of the role of dietary long-chain polyunsaturated fatty acids on neural indices of term infants: A randomized, controlled trial.	Intervention
Makrides 1995	Are long-chain polyunsaturated fatty acids essential nutrients in infancy?	Population
Makrides 1999	Dietary long-chain polyunsaturated fatty acids do not influence growth of term infants: A randomized clinical trial	Population
Makrides 2000a	A randomized trial of different ratios of linoleic to alpha-linolenic acid in the diet of term infants: Effects on visual function and growth	Other
Malcolm 1970	Growth retardation in a new Guinea boarding school and its response to supplementary feeding	Population

© 2019 Park JJH et al. *JAMA Network Open*.

First author, year	Title	Reason for exclusion
Maleta 2004	Supplementary feeding of underweight, stunted Malawian children with a ready-to-use food	Population
Maleta K 2013	Efficacy of reduced-cost lns formulations for infants and young children in malawi: The ilins-dose trial	Other
Malik 2013	Short-course prophylactic zinc supplementation for diarrhea morbidity in infants of 6 to 11 months	Outcome
Mangel 2015	Higher fat content in breastmilk expressed manually: a randomized trial	Outcome
Manger 2008	A micronutrient-fortified seasoning powder reduces morbidity and im- proves short-term cognitive function, but has no effect on anthropo- metric measures in primary school children in northeast Thailand: a randomized controlled trial	Population
Manno 2011	Rich micronutrient fortification of locally produced infant food does not improve mental and motor development of Zambian infants: a randomised controlled trial	Outcome
Mardones 2007	Effects of a dairy product fortified with multiple micronutrients and omega-3 fatty acids on birth weight and gestation duration in pregnant Chilean women	Outcome
Marin 2010	Randomized controlled trial of early skin-to- skin contact: effects on the mother and the newborn	Outcome
Marquis G 2017	Putting our eggs in more than one basket-lessons learned from working with multiple sectors in rural ghana	Other
Marsh 1959	Comparative Hematologic Response to Iron Fortification of a Milk Formula for Infants	Outcome
Martinez-Estevez 2016	Effects of zinc supplementation in the prevention of respiratory tract infections and diarrheal disease in colombian children: A 12-month randomised controlled trial	Outcome
Masanja 2015	Effect of neonatal vitamin a supplementation on mortality in infants in tanzania (neovita): A randomised, double-blind, placebo-controlled trial	Outcome
Mathur 2015	Zinc supplementation in preterm neonates and neurological development, a randomized controlled trial	Outcome
Mathur 2016	Assessment of adequacy of supplementation of vitamin d in very low birth weight preterm neonates: A randomized controlled trial	Intervention
Matias 2017	Home fortification during the first 1000 d improves child development in bangladesh: A cluster-randomized effectiveness trial	Outcome
Matos 2016	Prevention and treatment of anemia in infants through supplementation, assessing the effectiveness of using iron once or twice weekly	Outcome
Mattar 2007	Simple antenatal preparation to improve breastfeeding practice: a randomized controlled trial	Outcome
Maulen-Radovan 1994	Comparison of a rice-based, mixed diet versus a lactose-free, soy-protein isolate formula for young children with acute diarrhea	Outcome
Maurage 1998	Effect of two types of fish oil supplementation on plasma and erythrocyte phospholipids in formula-fed term infants.	Population
Mausezahl D 2015	Integrating environmental home-based interventions to improve the quality of household air, drinking water and hygiene in rural peru: Community-randomised controlled trial	Outcome
Mazumder 2014	Effect of implementation of Integrated Management of Neonatal and Childhood illness programme on treatment seeking practices for morbidities in infants: cluster randomised trial	Outcome
Mazumder 2015	Efficacy of early neonatal supplementation with vitamin a to reduce mortality in infancy in haryana, india (neovita): A randomised, double-blind, placebo-controlled trial	Outcome
Mazurek 1999	Influence of immediate newborn care on infant adaptation to the environment	Outcome
McClellan 1980	Effects of early mother- infant contact following cesarean birth	Outcome
McDonald 2015	Daily zinc but not multivitamin supplementation reduces diarrhea and upper respiratory infections in tanzanian infants: A randomized, double-blind, placebo-controlled clinical trial	Outcome
McGuigan 2011	A high compliance randomised controlled field trial of solar disinfection (SODIS) of drinking water and its impact on childhood diarrhoea in rural Cambodia.	Outcome
Mebrahtu 2004	Low-dose daily iron supplementation for 12 months does not increase the prevalence of malarial infection or density of parasites in young zanzibari children	Outcome
Medoua 2016	Recovery rate of children with moderate acute malnutrition treated with ready-to-use supplementary food (rusf) or improved corn-soya blend (csb+): A randomized controlled trial	Population
Mehta 2011	A randomized trial of multivitamin supplementation in children with tuberculosis in tanzania	Outcome
Meier 2008	A comparison of the efficiency, efficacy, comfort, and convenience of two hospital-grade electric breast pumps for mothers of very low birthweight infants	Outcome

© 2019 Park JJH et al. *JAMA Network Open*.

First author, year	Title	Reason for exclusion
Meier 2012	Breast<br>pump suction patterns that mimic the human infant<br>during breastfeeding: greater milk output in less time<br>spent pumping for breast pump-dependent mothers with premature infants	Outcome
Meinzen-Derr 2006	Risk of infant anemia is associated with exclusive breast-feeding and maternal anemia in a Mexican cohort.	Outcome
Menon 2007	Micronutrient Sprinkles reduce anemia among 9- to 24-mo-old children when delivered through an integrated health and nutrition program in rural Haiti.	Outcome
Menon 2016	Impacts on breastfeeding practices of at-scale strategies that combine intensive interpersonal counseling, mass media, and community mobilization: Results of cluster-randomized program evaluations in bangladesh and viet nam	Outcome
Mersmann 1993	Therapeutic Touch and Milk Letdown in Mothers of Non-nursing Preterm Infants [PhD Thesis]	Outcome
Mirnia 2017	Paternal skin-to-skin care and its effect on cortisol levels of the infants	Outcome
Mizuno 2004	Mother- infant skin-to-skin contact after delivery results in early recognition of own mother's milk odour.	Other
Mofd Ls 2016	The effect of maternal postpartum deworming on infection status, anemia and fatigue	Outcome
Mohammed H 2015	A cluster rct evaluating the effect of iodized salt on infant development in amhara region of ethiopia	Outcome
Mojibian 2015	The effects of vitamin d supplementation on maternal and neonatal outcome: A randomized clinical trial	Outcome
Moodley 2015	Single high-dose vitamin d at birth corrects vitamin d deficiency in infants in mexico	Outcome
Moore 2005	Randomized controlled trial or early mother-infant skin-to-skin contact and breastfeeding success	Other
Morley 1999	Iron fortified follow on formula from 9 to 18 months improves iron status but not development or growth: a randomised trial	Outcome
Mozaffari-Khosravi 2009	Effects of zinc supplementation on physical growth in 2–5-year-old children	Population
Muhangi 2013	Maternal hiv infection and other factors associated with growth outcomes of hiv-uninfected infants in entebbe, uganda	Study design
Muhihi 2016	Risk factors for small-for-gestational-age and preterm births among 19,269 tanzanian newborns	Outcome
Mullen 2012	The effects of micronutrient-fortified complementary/replacement food on intestinal permeability and systemic markers of inflammation among maternally hiv-exposed and unexposed zambian infants	Outcome
Muller 2001	Effect of zinc supplementation on malaria and other causes of morbidity in west African children: randomised double blind placebo controlled trial	Outcome
Muller 2003	Effect of zinc supplementation on growth in West African children: a randomized double-blind placebo-controlled trial in rural Burkina Faso	Outcome
Mustila 2013	Pragmatic controlled trial to prevent childhood obesity in maternity and child health care clinics: pregnancy and infant weight outcomes (The VACOPP Study)	Study design
Muthayya 2009	Effect of fortification with multiple micronutrients and n23 fatty acids on growth and cognitive performance in Indian schoolchildren: the CHAMPION (Children's Health and Mental Performance Influenced by Optimal Nutrition) study	Population
Mwanri 2000	Supplemental vitamin A im- proves anemia and growth in anemic school children in Tanzania	Population
Nackers 2010	Effectiveness of ready-to-use therapeutic food compared to a corn/soy-blend-based pre-mix for the treatment of childhood moderate acute malnutrition in niger	Population
Nagai 2010	Earlier versus later continuous Kangaroo Mother Care (KMC) for stable low-birth-weight infants: a randomized controlled trial	Outcome
Nahar 2015	Effect of a food supplementation and psychosocial stimulation trial for severely malnourished children on the level of maternal depressive symptoms in bangladesh	Outcome
Naheed 2009	Zinc therapy for diarrhoea improves growth among bangladeshi infants 6 to 11 months of age	Outcome
Nahidi 2011	Effect of early skin- to- skin contact of mother and newborn on mother's satisfaction.	Other
Ghavane 2012	Kangaroo Mother Care in Kangaroo ward for improving the growth and breastfeeding outcomes when reaching term gestational age in very low birth weight infants.	Population
Nakamura 1993	Mild to moderate zinc deficiency in short children: effect of zinc supplementation on linear growth velocity	Other
Namara 2017	Effects of treating helminths during pregnancy and early childhood on risk of allergy-related outcomes: Follow-up of a randomized controlled trial	Outcome
Namaste S 2017	Comparing the effectiveness and cost-effectiveness of facility-versus community-based distribution of micronutrient powders in rural uganda	Outcome

© 2019 Park JJH et al. *JAMA Network Open*.

First author, year	Title	Reason for exclusion
Nasehi 2012	The effect of early breastfeeding after cesarean section on the success of exclusive breastfeeding	Other
Navarro 2013	The double task of preventing malnutrition and overweight: a quasi-experimental community-based trial	Study design
Nazeri 2017	The effects of iodine fortified milk on the iodine status of lactating mothers and infants in an area with a successful salt iodization program: A randomized controlled trial	Outcome
Ndeezi 2010	Effect of multiple micronutrient supplementation on survival of hiv-infected children in uganda: A randomized, controlled trial	Population
Ndibazza 2012	Impact of anthelminthic treatment in pregnancy and childhood on immunisations, infections and eczema in childhood: a randomised controlled trial.	Outcome
Neu 2010	Maternal Holding of Preterm Infants<br>During the Early Weeks After Birth<br>and Dyad Interaction at Six Months	Study design
Nguyen 2002	Efficacy of daily and weekly iron supplementation for the control of iron deficiency anaemia in infants in rural Vietnam	Outcome
Nguyen 2017	Integrating nutrition interventions into an existing maternal, neonatal, and child health program increased maternal dietary diversity, micronutrient intake, and exclusive breastfeeding practices in bangladesh: Results of a cluster-randomized program evaluation	Outcome
Nguyen P 2016	Influences of early child nutritional status and home environment on child development in vietnam	Outcome
Nguyen P 2017	Feasibility and impacts of integrating nutrition interventions into an existing maternal, neonatal, and child health platform in bangladesh	Outcome
Nimbalkar 2014A	Effect of early skin-to-skin contact following normal delivery on incidence of hypothermia in neonates more than 1800 g: randomized control trial	Study design
Nimbalkar S 2016	Immediate skin to skin placement of the newborn on mothers abdomen stabilizes heart rate and rapidly normalizes oxygen saturation: A randomized controlled trial	Outcome
Ninh 1996	Zinc supplementation increases growth and circulating insulin-like growth factor I (IGF-I) in growth-retarded Vietnamese children	Outcome
Noel-Weiss 2006	Randomized controlled trial to determine effects of prenatal breastfeeding workshop on maternal breastfeeding self- efficacy and breastfeeding duration	Outcome
Nogueria (22) 2012	Use of Iron-Fortified Rice Reduces Anemia in Infants	Outcome
Nolan 2009	A pilot study of a nursing intervention protocol to minimize maternal-infant separation after Cesarean birth.	Outcome
Nopchinda 2002	Effect of bifidobacterium bb12 with or without streptococcus thermophilus supplemented formula on nutritional status	Other
Norouzi 2013	The impact of kangaroo care and music on maternal state anxiety	Other
Nowson 1997	A co-twin study of the effect of calcium supplementation on bone density during adolescence	Population
Nurko 1998	Successful use of a chicken-based diet for the treatment of severely malnourished children with persistent diarrhea: A prospective, randomized study	Outcome
Oaks 2017	Effects of a lipid-based nutrient supplement during pregnancy and lactation on maternal plasma fatty acid status and lipid profile: Results of two randomized controlled trials	Outcome
Obatolu 2003	Growth pattern of infants fed with a mixture of extruded malted maize and cowpea	Outcome
Ocansey Me 2017	Pre-and post-natal lipid-based nutrient supplements and cognitive, socioemotional and motor function in preschool-aged children in ghana	Other
Oddy 1999	Association between breast feeding and asthma in 6 year old children: findings of a prospective birth cohort study.	Outcome
Okronipa 2018	Maternal supplementation with small-quantity lipid-based nutrient supplements during pregnancy and lactation does not reduce depressive symptoms at 6 months postpartum in ghanaian women: A randomized controlled trial	Outcome
Okronipa H 2017B	Impact of exposure to lipid-based nutrient supplements in early life on sweet taste preference of ghanaian children aged 4-6 years: A non-inferiority study	Population
Okronipa Het 2016	The impact of lipid-based nutrient supplements on maternal depression at 6 mo postpartum in ghana: A randomized-controlled trial	Outcome
Olenick 2010	The effect of structured group prenatal education on breastfeeding confidence, duration, and exclusivity to 12 weeks postpartum	Outcome
Olsen 2000	The impact of iron supplementation on reinfection with intestinal helminths and Schistosoma mansoni in western Kenya	Population
Onayade 2004	THE FIRST SIX MONTH GROWTH AND ILLNESS OF EXCLUSIVELY AND NON-EXCLUSIVELY BREAST-FED INFANTS IN NIGERIA	Outcome
Osei A 2013	Using homestead food production program as a platform to deliver multiple micronutrient powders to infants and young children in nepal	Other

© 2019 Park JJH et al. *JAMA Network Open*.

First author, year	Title	Reason for exclusion
Osendarp 2002	Effect of zinc supplementation between 1 and 6 mo of life on growth and morbidity of bangladeshi infants in urban slums	Population
Osrin 2005	Effects of antenatal multiple micronutrient supplementation on birthweight and gestational duration in Nepal: double-blind, randomised controlled trial.	Population
Ostadrahimi 2017	The effect of perinatal fish oil supplementation on neurodevelopment and growth of infants: A randomized controlled trial	Population
Owino 2007	Fortified complementary foods with or without alpha-amylase treatment increase hemoglobin but do not reduce breast milk intake of 9-mo-old zambian infants	Outcome
Owino 2015	Winfood data from kenya and cambodia: Constraints on field procedures	Outcome
Paganini 2017	Prebiotic galacto-oligosaccharides mitigate the adverse effects of iron fortification on the gut microbiome: A randomised controlled study in kenyan infants	Outcome
Palmer 2018	Impact of biofortified maize consumption on serum carotenoid concentrations in zambian children	Outcome
Paragaroufalis 2014	A randomized double blind controlled safety trial evaluating d-lactic acid production in healthy infants fed a lactobacillus reuteri-containing formula	Outcome
Parker 2012	Effect of early breast milk expression on milk volume and timing of lactogenesis stage II among mothers of very low birth weight infants: A pilot study	Outcome
Partty 2013	Effects of early prebiotic and probiotic supplementation on development of gut microbiota and fussing and crying in preterm infants: A randomized, double-blind, placebo-controlled trial	Outcome
Pate 1999	EFFECTIVENESS OF WEB-BASED PROGRAMS IN IMPROVING BREASTFEEDING SELF-EFFICACY	Outcome
Paul 1996	Manual and pump methods of expression of breast milk	Outcome
Payne 2007	Benefit of vitamin a supplementation on ascaris reinfection is less evident in stunted children	Outcome
Pearson 2017	Effectiveness of household lockable pesticide storage to reduce pesticide self-poisoning in rural asia: A community-based, cluster-randomised controlled trial	Outcome
Penny 2004	Randomized controlled trial of the effect of daily supplementation with zinc or multiple micronutrients on the morbidity, growth, and micronutrient status of young Peruvian children	Outcome
Penny 2005	Effectiveness of an educational intervention delivered through the health services to improve nutrition in young children: a cluster-randomised controlled trial.	Outcome
Pereira 1969	Lysine-supplemented wheat and growth of preschool children	Other
Pereira 1973	Feeding trials with lysine- and threonine-fortified rice	Other
Pereira 1979	A preliminary simulated iron fortification trial in South Indian preschool children	Other
Perkin 2016	Randomized trial of introduction of allergenic foods in breast-fed infants	Population
Perumal 2017	Prenatal vitamin d supplementation and infant vitamin d status in bangladesh	Outcome
Pessoto 2010	Is a high quality expressed breast milk, according to expression method, possible for the premature baby in a third world country?	Outcome
Pettifor 1981	The effect of dietary calcium supplementation on serum calcium, phosphorus, and alkaline phosphatase concentrations in a rural black population	Population
Pinelli 2001	Randomized trial of breastfeeding support in very low-birth-weight infants	Outcome
Pinnock 1988	Vitamin A status of children with a history of respiratory syncytial virus infection in infancy.	Other
Pisacane 1995	Iron status in breast-fed infants	Other
Pitchik H 2017	Prenatal nutrition, stimulation, and exposure to punishment are associated with early child motor, cognitive, and socioemotional development in dar es salaam, tanzania	Other
Pittard 1991	Bacterial contamination of human milk: container type and method of expression	Outcome
Pollitt 2000	Effects of an energy and micronutrient supplement on growth and development in undernourished children in indonesia: Methods	Outcome
Pollitt 2000a	Effect of an energy and micro-nutrient supplement on growth and development in undernourished children in Indonesia: methods	Outcome
Ponder 1992	Docosahexaenoic acid status of term infants fed breast milk or infant formula containing soy oil or corn oil	Population
Pontes 2016	Cow's milk-based beverage consumption in 1- to 4-year-olds and allergic manifestations: An rct	Outcome
Prado 2012	Maternal multiple micronutrient supplements and child cognition: a randomized trial in Indonesia	Population
Prado 2016A	Effects of maternal and child lipid-based nutrient supplements on infant development: A randomized trial in malawi	Outcome

© 2019 Park JJH et al. *JAMA Network Open*.

First author, year	Title	Reason for exclusion
Prado 2016B	Effects of pre- and post-natal lipid-based nutrient supplements on infant development in a randomized trial in ghana	Outcome
Prado 2016C	Lipid-based nutrient supplements plus malaria and diarrhea treatment increase infant development scores in a cluster-randomized trial in burkina faso	Outcome
Prado 2016D	Provision of lipid-based nutrient supplements from age 6 to 18 months does not affect infant development scores in a randomized trial in malawi	Outcome
Prasad 2016	Impact of Sorghum supplementation on growth and micronutrient status of school going children in Southern India — a randomized trial	Population
Prendergast 2011	Improved growth and anemia in HIV-infected African children taking cotrimoxazole prophylaxis	Population
Prime 2010	The effect of breastshield size and anatomy on milk removal in women	Outcome
Prudhon 2017	Effect of ready-to-use foods for preventing child undernutrition in niger: Analysis of a prospective intervention study over 15months of follow-up	Study design
Pulakka 2015	Effect of 12-month intervention with lipid-based nutrient supplements on physical activity of 18-month-old malawian children: A randomised, controlled trial	Outcome
Pulakka 2017	Effect of 12-month intervention with lipid-based nutrient supplement on the physical activity of malawian toddlers: A randomised, controlled trial	Outcome
Punthmatharith 2001	Randomized Controlled Trial of early Kangaroo Care (Skin-to-Skin) Care: Effects on Maternal Feelings, Maternal-Infant Interaction and Breastfeeding Success in Thailand dissertation]	Outcome
Radhakrishna 2013	Effectiveness of zinc supplementation to full term normal infants: A community based double blind, randomized, controlled, clinical trial	Population
Raeisi 2014	A single center study of the effects of trained fathers' participation in constant breastfeeding	Outcome
Rahman 1999	Long-term supplementation with iron does not enhance growth in malnourished bangladeshi children	Population
Rahman 2001	Simultaneous zinc and vitamin A supplementation in Bangladeshi children: randomised double blind controlled trial	Outcome
Rahman 2002	Short-term supplementation with zinc and vitamin a has no significant effect on the growth of undernourished bangladeshi children	Population
Ramakrishnan 1995	Vitamin a supplementation does not improve growth of preschool children: A randomized, double-blind field trial in south india	Other
Ramakrishnan 2003	Multiple micronutrient supplementation during pregnancy does not lead to greater infant birth size than does iron-only supplementation: a randomized controlled trial in a semirural community in Mexico	Population
Ramakrishnan 2004	Multimicronutrient interventions but not vitamin a or iron interventions alone improve child growth: Results of 3 meta-analyses	Study design
Ramanathan 2001	Kangaroo Mother Care in Very Low Birth Weight Infants	Study design
Ramirez-Luzuriaga 2016	A food transfer program without a formal education component modifies complementary feeding practices in poor rural mexican communities	Outcome
Rao 1992	Prolonged breast-feeding and malnutrition among rural Indian children below 3 years of age.	Outcome
Rasmussen 2011	Interventions to increase the duration of breastfeeding in obese mothers: the Bassett Improving Breastfeeding Study	Outcome
Rawat R 2015	Sale of micronutrient powders (mnps) by frontline workers (flws) enables high reach, but low uptake limits impact on anemia and iron status: A cluster randomized study in bangladesh	Other
Reinbott 2016	Nutrition education linked to agricultural interventions improved child dietary diversity in rural cambodia	Study design
Rivera 1991	Effect of supplementary feeding on recovery from mild to moderate wasting in preschool children	Outcome
Rivera 1995	Nutritional supplementation during the preschool years influences body size and composition of guatemalan adolescents	Outcome
Rivera 1996	The recovery of guatemalan children with mild to moderate wasting: Factors enhancing the impact of supplementary feeding	Outcome
Roberfroid 2008	Effects of maternal multiple micronutrient supplementation on fetal growth: a double-blind randomized controlled trial in rural Burkina Faso	Population
Roberfroid 2012	Impact of prenatal multiple micronutrients on survival and growth during infancy: a randomized controlled trial.	Outcome
Roberts 2000	A Comparison of Kangaroo Mother Care and Conventional Cuddling Care	Outcome
Rojas 2003	Somatic Growth of Preterm Infants During Skin-to-Skin Care Versus Traditional Holding: A Randomized, Controlled Trial	Population
Ronaghy 1969	Controlled zinc supplementation for malnourished school boys: a pilot experiment	Population
Ronaghy 1974	Zinc supplementation of malnourished schoolboys in Iran: increased growth and	Population

© 2019 Park JJH et al. *JAMA Network Open*.

First author, year	Title	Reason for exclusion
	other effects	
Rosado 1997	Zinc supplementation reduced morbidity, but neither zinc nor iron supplementation affected growth or body composition of Mexican preschoolers	Population
Rosado 1999	Separate and joint effects of micronutrient deficiencies on linear growth	Population
Rosado 2009	Interaction of zinc or vitamin a supplementation and specific parasite infections on mexican infants' growth: A randomized clinical trial	Population
Rosado 2011	Effectiveness of the nutritional supplement used in the mexican oportunidades programme on growth, anaemia, morbidity and cognitive development in children aged 12-24 months	Population
Rossiter	The effect of a culture-specific education program to promote breastfeeding among Vietnamese women in Sydney	Outcome
Rotheram-Borus 2014	A cluster randomised controlled effectiveness trial evaluating perinatal home visiting among South African mothers/infants	Outcome
Rothman 2015	Acceptability of novel small-quantity lipid-based nutrient supplements for complementary feeding in a peri-urban south african community	Outcome
Roy 1999	Impact of zinc supplementation on subsequent growth and morbidity in bangladeshi children with acute diarrhoea	Outcome
Roy 2007	Prevention of malnutrition among young children in rural Bangladesh by a food-health-care educational intervention: a randomized, controlled trial.	Outcome
Ruel 1997	Impact of zinc supplementation on morbidity from diarrhea and respiratory infections among rural Guatemalan children	Outcome
Ruz 1997	A 14-mo zinc-supplementation trial in apparently healthy Chilean pre- school children	Population
Ryan 2014	Zinc or albendazole attenuates the progression of environmental enteropathy: A randomized controlled trial	Outcome
Ryser 2004	Breastfeeding attitudes, intention, and initiation in low-income women: the effect of the best start program.	Outcome
Saadi 2009	Effect of combined maternal and infant vitamin D supplementation on vitamin D status of exclusively breastfed infants	Outcome
Santos 2001	Nutrition counseling increases weight gain among Brazilian children.	Outcome
Santosham 1990	A comparison of rice-based oral rehydration solution and 'early feeding' for the treatment of acute diarrhea in infants	Outcome
Sarkar 2017	Study design and baseline results of an open-label cluster randomized community-intervention trial to assess the effectiveness of a modified mass deworming program in reducing hookworm infection in a tribal population in southern india	Outcome
Sarma 2006	Effect of micronutrient supplementation on health and nutritional status of schoolchildren: growth and morbidity	Population
Sato W 2017	A macro and micronutrient fortified complementary food supplement enhances plasma branched-chain amino acid levels in ghanaian infants	Other
Sattar 2012	Efficacy of a high-dose in addition to daily low-dose vitamin a in children suffering from severe acute malnutrition with other illnesses	Study design
Savilahti 1987	Prolonged exclusive breast feeding and heredity as determinants in infantile atopy	Outcome
Sayeg 2000	Linear growth and zinc supplementation in children with short stature	Other
Sazawal 2006	Zinc supplementation reduces the incidence of persistent diarrhea and dysentery among low socioeconomic children in India	Outcome
Scalabrin 2009	Growth and Tolerance of Healthy Term Infants Receiving Hydrolyzed Infant Formulas Supplemented With Lactobacillus rhamnosus GG: Randomized, Double-Blind, Controlled Trial	Population
Schlegelmilch 2016	Evaluation of water, sanitation and hygiene program outcomes shows knowledge-behavior gaps in coast province, kenya	Outcome
Schlickau 2005	Development and testing of a prenatal breastfeeding education intervention for Hispanic women	Outcome
Schroeder 1995	Age differences in the impact of nutritional supplementation on growth	Outcome
Schwartz 2014	Medium-term impact of a pro-breastfeeding and healthy complementary feeding intervention on growth and prevalence of overweight in children: a randomized clinical trial with adolescent mothers and grandmothers	Outcome
Scott 1998	Formula supplementation with long-chain polyunsaturated fatty acids: Are there developmental benefits?	Population
Scrimshaw 1953	Effect of dietary supplements and the administration of vitamin B12 and aureomycin on the growth of school children	Population
Semba 1995	Reduced seroconversion to measles in infants given vitamin A with measles vaccination	Outcome
Semba 2005	Effect of periodic vitamin a supplementation on mortality and morbidity of human	Outcome

© 2019 Park JJH et al. *JAMA Network Open*.

First author, year	Title	Reason for exclusion
	immunodeficiency virus-infected children in uganda: A controlled clinical trial	
Sempértegui 1996	Effects of short- term zinc supplementation on cellular immunity, respiratory symp-toms, and growth of malnourished Equadorian children	Other
Sempertegui 1999	The beneficial effects of weekly low-dose vitamin A supplementation on acute lower respiratory infections and diarrhea in Ecuadorian children.	Outcome
Serwint 1996	A randomized controlled trial of prenatal pediatric visits for urban, low-income families	Population
Shafique S 2016	Mineral- and vitamin-enhanced micronutrient powder reduces stunting in full-term low-birth-weight infants receiving nutrition, health, and hygiene education: a 2 3 2 factorial, cluster-randomized trial in Bangladesh	Other
Shankar 2008	Effect of maternal multiple micronutrient supplementation on fetal loss and infant death in Indonesia: a double-blind cluster-randomised trial	Population
Sharieff 2006	Short- term daily or weekly administration of micronutrient Sprinkles has high compliance and does not cause iron overload in Chinese schoolchildren: a cluster-randomised trial	Outcome
Sharma 2016A	Efficacy of early skin-to-skin contact on the rate of exclusive breastfeeding in term neonates: A randomized controlled trial	Outcome
Sharma 2016B	The effect of kangaroo ward care in comparison with "intermediate intensive care" on the growth velocity in preterm infant with birth weight <1100 g: Randomized control trial	Intervention
Sharma 2016C	To compare cost effectiveness of 'kangaroo ward care' with 'intermediate intensive care' in stable very low birth weight infants (birth weight<1100 grams): A randomized control trial	Outcome
Sharma 2017	Study comparing "kangaroo ward care" with "intermediate intensive care" for improving the growth outcome and cost effectiveness: Randomized control trial	Other
Shatrugna 2006	Effect of micronutrient supplement on health and nutritional status of schoolchildren: bone health and body composition	Population
Sheikh 2017	The efficacy of early iron supplementation on postpartum depression, a randomized double-blind placebo-controlled trial	Outcome
Shiau 1997	Randomized controlled trial of kangaroo care with full term infants: effects on breastmilk maturation, breast engorgement, and breastfeeding status	Outcome
Siddiqua 2016	Vitamin b12 supplementation during pregnancy and postpartum improves b12 status of both mothers and infants but vaccine response in mothers only: A randomized clinical trial in bangladesh	Outcome
Sikander 2015	Cognitive-behavioral counseling for exclusive breastfeeding in rural pediatrics: A cluster rct	Outcome
Silva 2008	Growth in non-anemic infants supplemented with different prophylactic iron doses	Other
Simondon 1996	Effect of early, short-term supplementation on weight and linear growth of 4-7-mo-old infants in developing countries: A four-country randomized trial	Population
Simondon 1997	Age at introduction of complementary food and physical growth from 2 to 9 months in rural Senegal	Outcome
Simondon 1997a	Lactational amenorrhea is associated with child age at the time of introduction of complementary food: a prospective<br>cohort study in rural Senegal,West Africa.	Outcome
Singh H 2017	Daily supplementation with 400 iu vitamin d in term breast fed infants from 0-6 months and changes in total and bone specific alkaline phosphatase-a rct	Outcome
Skau Jkh 2014	The use of linear programming to determine whether a formulated complementary food product can ensure adequate nutrients for 6- to 11-month-old Cambodian infants	Study design
Slayton 2016	A cluster randomized controlled evaluation of the health impact of a novel antimicrobial hand towel on the health of children under 2 years old in rural communities in nyanza province, kenya	Outcome
Sloan 1994	Kangaroo mother metthod: randomised controlled trial of an alternative method of care for stabilised low-birthweight infants	Outcome
Slusher 2007	Electric breast pump use increases maternal milk volume in African nurseries	Outcome
Smith 2014	Multiple micronutrient supplementation transiently ameliorates environmental enteropathy in malawian children aged 12-35 months in a randomized controlled clinical trial	Outcome
Smith 2016	The effect of neonatal vitamin a supplementation on morbidity and mortality at 12 months: A randomized trial	Outcome
Sneed 1981	The effects of ascorbic acid, vitamin B6, vitamin B12, and folic acid supplementation on the breast milk and maternal nutritional status of low socioeconomic lactating women	Population
Solon 2003	Effect of a multiple-micronutrient- fortified fruit powder beverage on the nutrition status, physical fitness, and cognitive performance of schoolchildren in the	Population

© 2019 Park JJH et al. *JAMA Network Open*.

First author, year	Title	Reason for exclusion
	Philippines	
Some 2015	Effect of zinc added to a daily small-quantity lipid-based nutrient supplement on diarrhoea, malaria, fever and respiratory infections in young children in rural burkina faso: A cluster-randomised trial	Outcome
Somnath 2017	Therapeutic effect of vitamin d in acute lower respiratory infection: A randomized controlled trial	Outcome
Soofi 2017	Evaluation of the uptake and impact of neonatal vitamin a supplementation delivered through the lady health worker programme on neonatal and infant morbidity and mortality in rural pakistan: An effectiveness trial	Outcome
Sosa 1976	The effect of early mother-infant contact on breastfeeding, infection and growth	Outcome
Specker 2003	Randomized trial of physical activity and calcium supplementation on bone mineral content in 3- to 5-year-old children	Other
Srinivasan 2016	Household and personal factors are sources of heterogenity in intestinal parasite clearance among mexican children 6-15 months of age supplemented with vitamin a and zinc	Outcome
Srivastava 2014	Effect of very early skin to skin contact on success at breastfeeding and preventing early hypothermia in neonates	Study design
Stabell 1995	No evidence of fontanelle-bulging episodes after vitamin A supplementation of 6- and 9-month-old infants in Guinea Bissau	Other
Stellwagen 2010	Hand expression combined with pumping does not change the nutrient content of human milk from mothers of very low birthweight infants	Outcome
Stewart 2017A	Biological pathways through which eggs may influence child growth and development	Other
Stewart 2017B	The impact of maternal diet fortification with lipid-based nutrient supplements on postpartum depression in rural malawi: A randomised-controlled trial	Outcome
Stewart C 2017	Factorial trial of lipid-based nutrient supplements with infant and young child feeding counseling with or without improved wash in kenya: Effects on anemia, iron, vitamin a, vitamin b12 and folate	Other
Stobaugh 2016	Including whey protein and whey permeate in ready-to-use supplementary food improves recovery rates in children with moderate acute malnutrition: A randomized, double-blind clinical trial	Outcome
Stobaugh 2017	Effect of a package of health and nutrition services on sustained recovery in children after moderate acute malnutrition and factors related to sustaining recovery: A cluster-randomized trial	Outcome
Stoltzfus 2001	Effects of iron supplementation and anthelmintic treatment on motor and language development of preschool children in Zanzibar: double blind, placebo controlled study	Outcome
Stoltzfus 2004	Low dose daily iron supplementation improves iron status and appetite but not anemia, whereas quarterly anthelminthic treatment improves growth, appetite and anemia in zanzibari preschool children	Population
Strand 2015	Vitamin b-12, folic acid, and growth in 6- to 30-month-old children: A randomized controlled trial	Outcome
Stutte 1988	The effects of breast massage on volume and fat content of human milk	Other
Suchdev 2010	Monitoring the marketing, distribution, and use of Sprinkles micronutrient powders in rural western Kenya. Food & Nutrition Bulletin 2010, 31(Supplement 2):	Outcome
Suchdev 2012	Selling sprinkles micronutrient powder reduces anemia, iron deficiency, and vitamin a deficiency in young children in western kenya: A cluster-randomized controlled trial	Outcome
Suchdev 2016	Effects of community-based sales of micronutrient powders on morbidity episodes in preschool children in western kenya	Outcome
Sudfeld 2015	Malnutrition and its determinants are associated with suboptimal cognitive, communication, and motor development in tanzanian children	Study design
Suman 2008	Kangaroo mother care for low birth weight infants: a randomized controlled trial	Outcome
Sung 2014	Treating infant colic with the probiotic Lactobacillus reuteri: Double blind, placebo controlled randomised trial	Outcome
Sungthong 2002	Once weekly is superior to daily iron supplementation on height gain but not on hematological improvement among schoolchildren in Thailand	Population
Sur 2003	Impact of zinc supplementation on diarrheal morbidity and growth pattern of low birth weight infants in kolkata, India: a randomized, double-blind, placebo-controlled, community-based study.	Outcome
Surkan 2015	The role of zinc and iron-folic acid supplementation on early child temperament and eating behaviors in rural nepal: A randomized controlled trial	Population
Svefors 2018	Cost-effectiveness of prenatal food and micronutrient interventions on under-five mortality and stunting: Analysis of data from the minimat randomized trial, bangladesh	Study design

© 2019 Park JJH et al. *JAMA Network Open*.

First author, year	Title	Reason for exclusion
Svejda 1980	Mother-infant bonding: failure to generalize. Child Development	Population
Swarnkar 2016	Effect of kangaroo mother care on growth and morbidity pattern in low birth weight infants.	Study design
Syfrett 1993	Very Early and Virtually Continuous Kangaroo Care for 34-36 week Gestation Preterm Infants: Effects on Temperature, Breastfeeding, Supplementation and Weight [thesis]	Outcome
Tahan 2007	A randomized double-blind clinical trial of the effect of non-absorbable oral polymyxin on infants with severe infectious diarrhea	Outcome
Talukder A 2017	The role of small scale aquaculture and enhanced homestead food production in improving household food security and nutrition	Other
Tanchoco 2007	Diet supplemented with mct oil in the management of childhood diarrhea	Outcome
Tang 2017	Iron in micronutrient powder promotes an unfavorable gut microbiota in kenyan infants	Outcome
Tariku 2015	Application of the health belief model to teach complementary feeding messages in ethiopia	Outcome
Tavil 2003	Effect of twice weekly versus daily iron treatment in Turkish children with iron deficiency anemia.	Outcome
Tchum 2006	Evaluation of vitamin a supplementation regimens in ghanaian postpartum mothers with the use of the modified-relative-dose-response test	Outcome
Tergestina 2016	A randomized double-blind controlled trial comparing two regimens of vitamin d supplementation in preterm neonates	Outcome
Teshome 2018	Adherence to home fortification with micronutrient powders in kenyan pre-school children: Self-reporting and sachet counts compared to an electronic monitoring device	Outcome
Thakur 2012	Effect of nutrition education on exclusive breastfeeding for nutritional outcome of low birth weight babies	Outcome
Thomson 1979	The importance of immediate postnatal contact: its effect on breastfeeding	Outcome
Thukral 2012	Early skin-to-skin contact and breast-feeding behavior in term neonates: a randomized controlled trial	Outcome
Timby 2014	Neurodevelopment, nutrition, and growth until 12 mo of age in infants fed a low-energy, low-protein formula supplemented with bovine milk fat globule membranes: A randomized controlled trial	Outcome
Tofall 2008	Effects of prenatal food and micronutrient supplementation on infant development: a randomized trial from the Maternal and Infant Nutrition Interventions, Matlab (MINIMat) study	Population
Tomiya 2017	The effect of vitamin a supplementation with 400 000 iu vs 200 000 iu on retinol concentrations in the breast milk: A randomized clinical trial	Outcome
Tomlinson 2011	An effectiveness study of an integrated, community-based package for maternal, newborn, child and HIV care in South Africa: study protocol for a randomized controlled trial	Outcome
Tomlinson 2014	Goodstart: a cluster randomised effectiveness trial of an integrated, community-based package for maternal and newborn care, with prevention of mother-to-child transmission of HIV in a South African township	Outcome
Torabi 2014	The effect of calcium and phosphorus supplementation on metabolic bone disorders in premature infants	Outcome
Torrejon 2004	Zinc and Iron Nutrition in Chilean Children Fed Fortified Milk Provided by the Complementary National Food Program	Outcome
Trehan 2009	A randomized, double-blind, placebo-controlled trial of rifaximin, a nonabsorbable antibiotic, in the treatment of tropical enteropathy	Population
Trehan 2013	Antibiotics as part of the management of severe acute malnutrition	Population
Tuthill 2002	Randomized double-blind controlled trial on the effects on iron status in the rst year between a no added iron and standard infant formula received for three months*	Outcome
Tylleskar 2011	Exclusive breastfeeding promotion by peer counsellors in sub-Saharan Africa (PROMISE-EBF): a cluster-randomised trial.	Outcome
Ullah B 2017	Effect of pre-and postnatal nutritional supplements on childhood illnesses in bangladesh: A cluster-randomized effectiveness trial	Other
Unger 2017	Impact of fortified versus unfortified lipid-based supplements on morbidity and nutritional status: A randomised double-blind placebo-controlled trial in ill gambian children	Outcome
Urban 2008	Growth of infants born to HIV-infected women when fed a biologically acidi ed starter formula with and without probiotics	Population
Vahdati 2017	Effect of kangaroo care combined with music on the mother-premature neonate	Outcome

© 2019 Park JJH et al. *JAMA Network Open*.

First author, year	Title	Reason for exclusion
	attachment: A randomized controlled trial	
Vaidya 2005	Effect of early mother-baby close contact over the duration of exclusive breastfeeding	Outcome
Vaidya 2008	Effects of antenatal multiple micronutrient supplementation on children's weight and size at 2 years of age in nepal: Follow-up of a double-blind randomised controlled trial	population
van Goor 2010	Supplementation of DHA but not DHA with arachidonic acid during pregnancy and lactation influences general movement quality in 12-week- old term infants	Outcome
Vasan 2004	Maternal milk volume (MMV) and milk transfer (MT) for low birth weight (LBW: <2500G) infants in Ecuador: comparison of electric breast pump and hand expression	Population
Vazir 2013	Cluster-randomized trial on complementary and responsive feeding education to caregivers found improved dietary intake, growth, and development among rural Indian toddlers.	Population
Vaziri 2016	Vitamin d supplementation during pregnancy on infant anthropometric measurements and bone mass of mother-infant pairs: A randomized placebo clinical trial	Outcome
Veenemans 2011	Effect of supplementation with zinc and other micronutrients on malaria in tanzanian children: A randomised trial	Outcome
Veenemans 2012	Effect of preventive supplementation with zinc and other micronutrients on non-malarial morbidity in tanzanian pre-school children: A randomized trial	Outcome
Velaphi 2008	Growth and metabolism of infants born to women infected with human immunodeficiency virus and fed acidified whey-adapted starter formulas	Population
Venkatarao 1996	Effect of vitamin A supplementation to mother and infant on morbidity in infancy.	Outcome
Verhoef 2002	Malarial anemia leads to adequately increased erythropoiesis in asymptomatic Kenyan children.	Outcome
Verna M 2012	Parma pap project: A ready to use therapeutic food for moderately malnourished children in sierra leone	Other
Villalon 1992	Effect of early skin-to-skin contact on temperature regulation, heart rate, and respiratory rate in healthy, full-term newborns [Contacto precoz piel a piel: efecto sobre los parametros fisiologicos en las cuatro horas posteriores al parto en recien nacidos de termino sanos]	Study design
Villamor 2005	Vitamin supplementation of hiv-infected women improves postnatal child growth	population
Vinod 2006	Impact of a multiple-micronutrient food supplement on the nutritional status of schoolchildren	Population
Wagner 2006	High-dose vitamin D3 supplementation in a cohort of breast- feeding mothers and their infants: a 6-month follow-up pilot study.	Population
Walker 1991	Nutritional supplementation, psychosocial stimulation, and growth of stunted children: the Jamaican study.	Outcome
Walravens 1983	Linear growth of low income pre- school children receiving a zinc supplement	Other
Walravens Pa 1992	Zinc supplements in breastfed infants	Outcome
Walter 1993	Effectiveness of Iron-Fortified Infant Cereal in Prevention of Iron Deficiency Anemia	Outcome
Wammes 2016	Community deworming alleviates geohelminth-induced immune hyporesponsiveness	Outcome
Wang 2005	Feeding practices in 105 counties in rural China.	Study design
Wang 2012	No effect of maternal micronutrient supplementation on early childhood growth in rural western China: 30 month follow-up evaluation of a double blind, cluster randomized controlled trial	Population
Wang 2017A	A combined intervention of zinc, multiple micronutrients, and albendazole does not ameliorate environmental enteric dysfunction or stunting in rural malawian children in a double-blind randomized controlled trial	Outcome
Wang 2017B	Effectiveness of community-based complementary food supplement (yingyangbao) distribution in children aged 6-23 months in poor areas in china	Study design
Warthon-Medina 2015	The long term impact of micronutrient supplementation during infancy on cognition and executive function performance in pre-school children	Outcome
Warthon-Medina M 2015	The long term impact of multiple micronutrient supplementation during infancy on cognition and executive function performance in pre-school children	Study design
Wegmuller 2016	Efficacy and safety of hepcidin-based screen-and-treat approaches using two different doses versus a standard universal approach of iron supplementation in young children in rural gambia: A double-blind randomised controlled trial	Outcome
Weizman 2006	Effect of a probiotic infant formula on infections in child care centers: comparison of two probiotic agents	Population
Wen 2012	Effectiveness of home based early intervention on children’s BMI at age 2:	Outcome

© 2019 Park JJH et al. *JAMA Network Open*.

First author, year	Title	Reason for exclusion
	randomised controlled trial	
West 1997	Effects of vitamin a on growth of vitamin a-deficient children: Field studies in nepal	Population
West 2014	Effect of maternal multiple micronutrient vs iron-folic acid supplementation on infant mortality and adverse birth outcomes in rural Bangladesh: the JiVitA-3 randomized trial	Population
West 2014	Effect of maternal multiple micronutrient vs iron-folic acid supplementation on infant mortality and adverse birth outcomes in rural Bangladesh: the JiVitA-3 randomized trial.	Outcome
West Kp 2015	Effect of maternal multiple micronutrient vs iron-folic acid supplementation on infant mortality and adverse birth outcomes in rural bangladesh: The jivita-3 randomized trial	Population
Whitelaw 1988	Skin to skin contact for very low birth weight infants and their mothers	Outcome
Whiting S 2017	Feasibility study of an intervention of gifting chickens for young children in ethiopia for provision of egg and eggshell	Other
WHO 1994	An Evaluation of Infant Growth	Outcome
WHO 1997	Effects of timing of complementary foods on post-natal growth	Outcome
Widen 2015	Antiretroviral treatment is associated with iron deficiency in hiv-infected malawian women that is mitigated with supplementation, but is not associated with infant iron deficiency during 24 weeks of exclusive breastfeeding	Outcome
Willatts 1998a	Influence of long-chain polyunsaturated fatty acids on infant cognitive function	Population
Willatts 1998b	Effect of long-chain polyunsaturated fatty acids in infant formula on problem solving at 10 months of age	Population
Wiria 2013	The effect of three-monthly albendazole treatment on malarial parasitemia and allergy: a household-based cluster-randomized, double-blind, placebo-controlled trial.	Population
Wolfberg 2004	Dads as breastfeeding advocates: results from a randomized controlled trial of an educational intervention	Outcome
Wolfsdorf 1973	Trimethoprim-sulphonamide mixture in the treatment of infantile gastro-enteritis	Outcome
Wong 2014	Antenatal education to increase exclusive breastfeeding. A randomized controlled trial	Outcome
Worku 2005	Kangaroo Mother Care: A Randomized Controlled Trial on Effectiveness of Early Kangaroo Mother Care for the Low Birthweight Infants in Addis Ababa, Ethiopia	Outcome
Wright 2016	Subsidized sachet water to reduce diarrheal disease in young children: A feasibility study in accra, ghana	Outcome
Wuehler 2008	Dose-response trial of prophylactic zinc supplements, with or without copper, in young Ecuadorian children at risk of zinc deficiency	Outcome
Yang 2002	Effect of micronutrient supplementation on the growth of preschool children in China	Other
Yang 2004	Effect of daily or once weekly iron supplementation on growth and iron status of preschool children	Population
Yi it 2012	Does warming the breasts affect the amount of breastmilk production?	Outcome
Young M 2017	Role of preconception nutrition in offspring growth and risk of stunting across the first 1000 days in vietnam	Other
Yousafzai 2014	Effect of integrated responsive stimulation and nutrition interventions in the lady health worker programme in pakistan on child development, growth, and health outcomes: A cluster-randomised factorial effectiveness trial	Outcome
Yousefichaijan 2015	Oral zinc sulfate as adjuvant treatment in children with nephrolithiasis: A randomized, double-blind, placebo-controlled clinical trial	Outcome
Yurdakok 2004	Efficacy of daily and weekly iron supplementation on iron status in exclusively breast-fed infants	Outcome
Zadik 2004	Vitamin A and iron supplemen- tation is as efficient as hormonal therapy in constitutionally delayed children	Population
Zadik 2010	“Functional food” for acceleration of growth in short children born small for gestational age	Other
Zaman 2008	Training in complementary feeding counselling of healthcare workers and its influence on maternal behaviours and child growth: a cluster-randomized controlled trial in Lahore, Pakistan.	Outcome
Zeng 2008	Impact of micronutrient supplementation during pregnancy on birth weight, duration of gestation, and perinatal mortality in rural western China: double blind cluster randomised controlled trial	Population
Zhang 2016	Effectiveness of complementary food supplements and dietary counselling on anaemia and stunting in children aged 6-23 months in poor areas of qinghai province, china: A controlled interventional study	Outcome

© 2019 Park JJH et al. *JAMA Network Open*.

First author, year	Title	Reason for exclusion
Ziegler 2009	Iron status of breastfed infants is improved equally by medicinal iron and iron-fortified cereal	Population
Zimmermann 2007	Treatment of iodine deficiency in school-age children increases insulin-like growth factor (IGF)-I and IGF binding protein-3 concentrations and improves somatic growth	Population
Zinaman 1992	Acute prolactin and oxytocin responses and milk yield to infant suckling and artificial methods of expression in lactating women	Outcome
Zlotkin 2013	Effect of iron fortification on malaria incidence in infants and young children in ghana: A randomized trial	Outcome

© 2019 Park JJH et al. *JAMA Network Open*.

Details of the evidence base for each life stage

eTable 14. Trial characteristics for pregnancy life stage

Trial ID	Intervention domains	Unit of randomization	Blinding	Continents	Countries	Trimester at randomization:	Mean gestational age (weeks)	Follow-up and treatment duration
Adu-Afarwuah 2015	Balanced energy protein	Individual	Double blind	Africa	Ghana	1st/2nd	16.3	Until delivery
Ahmad 2016	Micronutrients	Individual	Double blind	South East Asia	Bangladesh	1st	13.35	Until 6 months of postpartum
Alizadeh 2016	Micronutrients	Individual	Double blind	Middle East	Iran	1st/2nd	18	Until delivery (mean 12 weeks)
Asci 2016	Lifestyle intervention	Individual	Single (subject) blind	Other	Turkey	1st	7.3	Until 6 weeks of postpartum
Asemi 2012	Micronutrients	Individual	Single blind	South East Asia	Pakistan	3rd	25	Until delivery (9 weeks)
Asemi 2013	Micronutrients	Individual	Double blind	Middle East	Iran	2nd	25	Until delivery (9 weeks)
Asemi 2015	Micronutrients	Individual	NR	Middle East	Iran	NR	NR	NR
Asemi 2016	Micronutrients	Individual	NR	Middle East	Iran	NR	NR	NR
Ashorn 2015A	Micronutrient and Balanced energy protein	Individual	Single (outcome assessor) blind	Africa	Malawi	1st/2nd	16.8	Until 6 months of postpartum
AViDD	Micronutrients	Individual	Double blind	South East Asia	Bangladesh	3rd	27.75	Until delivery
Belizan 1991	Micronutrients	Individual	Double blind	South America	Argentina	2nd	20.8	Until delivery
Bhutta 2009a	Micronutrients	Cluster	Single (outcome assessor) blind	South East Asia	Pakistan	1st/2nd	12.2	Until delivery
Callaghan-Gillespie 2017	Balanced energy protein	Individual	Single (outcome assessor) blind	Africa	Malawi	Not reported	NR	Until delivery (mean 12 weeks)
CARING trial	Community intervention	Cluster	Single (outcome assessor) blind	South East Asia	India	3rd	NR	Until 6 months of postpartum
Castillo-Duran 2001	Micronutrients	Individual	Double blind	South America	Chile	1st/2nd	12.65	Until delivery
Caulfield 1999	Micronutrients	Individual	Double blind	South America	Peru	1st/2nd	16	Until 4 weeks of postpartum
Ceesay 1997	Balanced energy protein	Individual	NR	Africa	Gambia	2nd	20	Until delivery
Christian 2003	Micronutrients	Cluster	Double blind	South East Asia	Nepal	1st	11.4	Until 12 weeks of postpartum
Cox 2005	Micronutrients	Individual	Double blind	Africa	Ghana	1st/2nd	16	Until 6 weeks of postpartum
Dandamrongrak 2016	Micronutrients	Individual	NR	South East Asia	Thailand	1st/2nd	14.8	Until delivery
Danesh 2010	Micronutrients	Individual	Double blind	Middle East	Iran	1st/2nd	14	Until delivery
Devi 2017	Micronutrient and Balanced energy protein	Individual	Open label	South East Asia	India	1st	11	Until delivery
Dijkhuizen 2004 BC	Micronutrients	Individual	Double blind	South East Asia	Indonesia	1st/2nd	16.325	Until delivery
Diogenes 2013	Micronutrients	Individual	Single (subject) blind	South America	Brazil	2nd	26	Until delivery
ENID trial	Balanced energy protein	Individual	Single (outcome assessor) blind	Africa	Gambia	2nd	13.8	Until delivery
Etheredge 2015	Micronutrients	Individual	Double blind	Africa	Tanzania	2nd/3rd	18.2	Until delivery
Fawzi 2007	Micronutrients	Individual	Double blind	Africa	Tanzania	1st/2nd	21.3	Until 6 weeks of postpartum
Fleming 1968	Micronutrients	Individual	NR	Africa	Nigeria	NR	NR	NR
Friss 2004	Micronutrients	Individual	Double blind	Africa	Zimbabwe	2nd/3rd	29	Until delivery
Goldberg 2013	Micronutrients	Individual	Double blind	Africa	Gambia	2nd	20.35	Until delivery
Gonzalez-Casanova 2015	Micronutrients	Individual	Double blind	Other	Mexico	2nd	20.5	Until delivery
Gowachirapant 2017	Micronutrients	Individual	Double blind	South East Asia	India, Thailand	1st	10.8	Until delivery

© 2019 Park JJH et al. *JAMA Network Open*.

Trial ID	Intervention domains	Unit of randomization	Blinding	Continents	Countries	Trimester at randomization:	Mean gestational age (weeks)	Follow-up and treatment duration
Hafeez 2005	Micronutrients	Individual	Double blind	South East Asia	Pakistan	1st/2nd	13	Until delivery
Hanieh 2017	Micronutrients	Cluster	Triple blind	South East Asia	Vietnam	1st	12	Until 12 weeks of postpartum
Hashemipour 2014	Micronutrients	Individual	Open label	Middle East	Iran	2nd	25	Until delivery (8 weeks)
Hossain 2014	Micronutrients	Individual	Open label	South East Asia	Pakistan	2nd	20	Until delivery
Huybregts 2009	Balanced energy protein	Individual	Open label	Africa	Burkina Faso	All trimesters	16.15	Until delivery
Iyengar 1975	Micronutrients	Individual	NR	South East Asia	India	1st/2nd	15	NR
Janmohamed 2016	Balanced energy protein	Cluster	Single (outcome assessor) blind	South East Asia	Cambodia	1st	8.3	Until delivery (6-8 months)
JiVitA-3	Micronutrients	Cluster	Double blind	South East Asia	Bangladesh	1st/2nd	9	Until 12 weeks of postpartum
Kaestel 2005	Micronutrients	Individual	Double blind	Africa	Guinea-Bissau	2nd/3rd	22	Until delivery (mean 17 weeks)
Kardjati 1988	Balanced energy protein	Individual	Open label	South East Asia	Indonesia	3rd	NR	Until delivery
Katz 2000	Micronutrients	Cluster	Double blind	South East Asia	Nepal	1st	3	Until delivery
Kumar 2009	Micronutrients	Individual	Double blind	South East Asia	India	1st/2nd	17.9	Until delivery
Kumwenda 2002	Micronutrients	Individual	Double blind	Africa	Malwi	2nd/3rd	23.25	Until delivery
Larocque 2006	Micronutrients and Deworming	Individual	Double blind	South America	Peru	2nd	20.4	Until delivery for micronutrients and single dose for deworming
Lopez-Jaramillo 1989	Micronutrients	Individual	Double blind	South America	Ecuador	2nd	24	Until delivery
Lopez-Jaramillo 1997	Micronutrients	Individual	Double blind	South America	Ecuador	2nd	20	Until delivery
Lui 2013	Micronutrients	Individual	Double blind	Other	China	1st/2nd	12	Until delivery
Mardones 2007	Balanced energy protein	Individual	Open label	South America	Chile	1st/2nd	11	Until delivery
Marya 1988	Micronutrients	Individual	NR	South East Asia	India	3rd	NR	Until delivery
Menedez 1994	Micronutrients	Individual	Double blind	Africa	Gambia	All trimesters	33	Until delivery
Merialdi 2004	Micronutrients	Individual	Double blind	South America	Peru	1st/2nd	13.4	Until 4 weeks of postpartum
MINIMAT	Micronutrient and Balanced energy protein	Individual	Double blind	South East Asia	Bangladesh	1st	9.4	Until 8 weeks of postpartum
Mohammad-Alizadeh-Charandabi 2015	Micronutrients	Individual	Triple blind	South East Asia	India	2nd/3rd	27.5	Until delivery
Mojibian 2015	Micronutrients	Individual	Open label	South East Asia	India	1st/2nd	14	Until delivery
Mwangi 2015	Micronutrients	Individual	Double blind	Africa	Kenya	2nd	17.5	Until 4 weeks of postpartum
Ndibazza 2011	Micronutrients and Deworming	Individual	Double blind	Africa	Uganda	2nd/3rd	26.6	Single dose
Ndyomugyenyi 2008	Micronutrients and Deworming	Individual	Open label	Africa	Uganda	2nd	23	Single dose
ObaapaVitA	Micronutrients	Cluster	Double blind	Africa	Ghana	All trimesters	NR	Until delivery
Osendarp 2000	Micronutrients	Individual	Double blind	South East Asia	Bangladesh	1st/2nd	14	Until delivery
Osrin 2005	Micronutrients	Individual	Double blind	South East Asia	Nepal	1st/2nd	16.1	Until delivery
Owens 2015	Micronutrients	Individual	Double blind	Africa	Gambia	1st	0	Until delivery
PRECONCEPT	Micronutrients	Individual	Double blind	South East Asia	Vietnam	1st	0	Until delivery

© 2019 Park JJH et al. *JAMA Network Open*.

Trial ID	Intervention domains	Unit of randomization	Blinding	Continents	Countries	Trimester at randomization:	Mean gestational age (weeks)	Follow-up and treatment duration
PROFEG	Micronutrients	Individual	Open label	Africa	Mozambique	1st/2nd	21.6	Until delivery
Purwar 1996	Micronutrients	Individual	Double blind	South East Asia	India	2nd	20	Until delivery
Radhika 2003	Micronutrients	Individual	Double blind	South East Asia	India	2nd/3rd	27	Until delivery (8 weeks)
Ramakrishnan 2003	Micronutrients	Individual	Double blind	Other	Mexico	1st	12	Until delivery
Rang-Din Nutrition study	Micronutrient and Balanced energy protein	Cluster	Single (outcome assessor) blind	South East Asia	Bangladesh	1st/2nd	12.8	Until delivery
Roberfroid 2008	Micronutrients	Individual	Double blind	Africa	Burkina Faso	All trimesters	17.35	Until delivery (mean 13 weeks)
Ruangvutilert 2017	Micronutrients	Individual	NR	South East Asia	Thailand	3rd	33	Until delivery
Saaka 2009	Micronutrients	Individual	Double blind	Africa	Ghana	1st/2nd	13.75	Until delivery
Sablok 2015	Micronutrients	Individual	NR	South East Asia	India	2nd	17	Until delivery
Satya Deepti 2015	Deworming	Individual	Double blind	Africa	Uganda	2nd/3rd	26.6	Single dose
Sayyah-Melli 2016	Micronutrients	Individual	Open label	Middle East	Iran	NR	14	Until delivery
SUMMIT 2008	Micronutrients	Cluster	Double blind	South East Asia	Indonesia	All trimesters	NR	Until 12 weeks of postpartum
Sunawang 2009	Micronutrients	Cluster	Double blind	South East Asia	Indonesia	All trimesters	14.4	Until 4 weeks of postpartum
Taherian 2002	Micronutrients	Individual	Double blind	Middle East	Iran	1st/2nd	22.55	Until delivery
Villar 2006	Micronutrients	Individual	Double blind	Asia and South America	Argentina; Egypt; India; Peru; South Africa;, Vietnam	1st/2nd	15.1	Until delivery
Wanchu 2001	Micronutrients	Individual	Open label	South East Asia	India	1st/2nd	20	Until delivery
Whitfield 2016	Micronutrients	Individual	Double blind	South East Asia	Cambodia	2nd/3rd	23.3	Until delivery
Yu 2008	Micronutrients	Individual	Open label	South East Asia	India	3rd	27	Until delivery
Zagre 2007	Micronutrients	Cluster	Double blind	Africa	Niger	1st	11	Until delivery
Zahiri Sorouri 2016	Micronutrients	Individual	Single (outcome assessor) blind	Middle East	Iran	2nd	16	Until delivery
Zeng 2008	Micronutrients	Cluster	Double blind	Other	China	All trimesters	13.8	Until delivery
Zhao 2015	Micronutrients	Individual	Triple blind	Other	China	2nd/3rd	15.85	Until delivery
Ziaei 2007	Micronutrients	Individual	Double blind	Middle East	Iran	2nd	13.4	Until delivery

© 2019 Park JJH et al. *JAMA Network Open*.

eTable 15. Trial characteristics for principal systematic review, exclusive breastfeeding life stage

Trial ID	Intervention domains	Unit of randomization	Blinding	Continents	Countries	Population intervened	Intervention duration (weeks)
Acharya 2014[178]	Kangaroo care	Individual	Open label	South East Asia	Nepal	Child	2
Adu-Afarwuah 2016[71,179]	Micronutrients and food supplements	Individual	Single blind (data analyst)	Africa	Ghana	Caregiver	24
Ashorn 2015A[180,181]	Micronutrients and food supplements	Individual	Single blind (outcome assessor)	Africa	Malawi	Caregiver	24
Boo 2007[182]	Kangaroo care	Individual	Open label	South East Asia	Malaysia	Child	2
CARING trial[87]	Home visit group counseling	Cluster	Not reported	South East Asia	India	Caregiver	24
Feliciano 1994[183]	Micronutrient	Individual	Open label	Other	China	Child	24
Gathwala 2010[184]	Kangaroo care	Individual	Open label	South East Asia	India	Child	1
Goodstart[185,186]	CHW education	Cluster	Single blind (outcome assessor)	Africa	South Africa	Caregiver	8
Habib 2015[187]	Micronutrient	Individual	Double blind, placebo controlled	South East Asia	Pakistan	Child	18
Hamadani 2001[188]	Micronutrient	Individual	Double blind, placebo controlled	South East Asia	Bangladesh	Child	20
JiVitA-3[189,190]	Micronutrient	Cluster	Double blind, placebo controlled	South East Asia	Bangladesh	Caregiver	12
Kumbhojkar 2016[191]	Kangaroo care	Individual	Open label	South East Asia	India	Child	1
Le Roux 2013[192,193]	CHW education	Cluster	Single blind (outcome assessor)	Africa	South Africa	Caregiver	8
Locks 2016[194,195]	Micronutrient	Individual	Double blind	Africa	Tanzania	Child	18
Lonnerdal 2017[196]	Food supplement	Individual	Double blind	Other	China	Child	24
LUCOMAI[197]	Maternal nutrition counseling	Cluster	Open label	Africa	Burkina Faso	Caregiver	24
Osendarp 2002[198]	Micronutrient	Individual	Double blind	South East Asia	Bangladesh	Child	20
Ostadrahimi 2017[199]	Food supplement	Individual	Triple blind	Other	Iran	Caregiver	8
PROMISE EBF[200-202]	Peer education on exclusive breastfeeding	Cluster	Open label	Africa	Burkina Faso; South Africa; Uganda	Caregiver	24
RDNS[203,204]	Micronutrients and food supplements	Cluster	Single blind (outcome assessor)	South East Asia	Bangladesh	Caregiver	24
Shafique 2016[205]	WASH	Cluster	Triple blind	South East Asia	Bangladesh	Caregiver	24
Simondon 1996[206]	Food supplement	Individual	Open label	Africa and South America	Congo, Senegal, Bolivia, New Caledonia	Child	12
Suman 2008[207]	Kangaroo care	Individual	Open label	South East Asia	India	Child	1
Urban 2008[208]	Food supplement	Individual	Double blind	Africa	South Africa	Child	24
Vazir 2013[209]	Maternal education	Cluster	Single blind (outcome assessor)	South East Asia	India	Caregiver	12
Velaphi 2008[210]	Food supplement	Individual	Double blind	Africa	South Africa	Child	24

© 2019 Park JJH et al. *JAMA Network Open*.

eTable 16. Trial characteristics for principal systematic literature review, complementary feeding stage

Trial ID	Intervention domains	Unit of randomization	Blinding	Continents	Countries	Intervention duration (months)
Adu-Afarwuah 2007	Micronutrients	Individual	NR	Africa	Kenya	6 months
Alarcon 1991	Lactose-adapted formula	Individual	NR	South America	Peru	6 days
Alarcon 1992	Balanced energy protein	Individual	NR	South America	Peru	6 days
Ashorn 2015	Micronutrients	Individual	Double blind	Africa	Malawi	12 months
Bhandari 2001	Food supplement	Individual	NR	South East Asia	India	8 months
Black 2004A	Micronutrients	Individual	Double blind	South East Asia	Bangladesh	6 months
Brooks 2005	Micronutrients	Individual	Double blind	South East Asia	Bangladesh	52 months
Brown 2007	Micronutrients	Individual	Double blind	South America	Peru	6 months
CARING trial	Maternal education	Cluster	NR	South East Asia	India	12 months
Castillo-Duran 1995	Micronutrients	Individual	Double blind	South America	Chile	6 months
Castillo-Duran 2001	Micronutrients	Individual	Double blind	South America	Chile	12 months
CIGNIS trial	Micronutrients	Individual	Double-blind	Africa	Zambia	12 months
Cohen 1994A	Balanced energy proteins	Individual		North America	Honduras	8 months
Dewey 2002	Micronutrients	Individual	Double-blind	North America, other	Honduras and Sweden	5 months
Dijkhuizen 2001	Micronutrients	Individual	Double blind	Asia	Vietnam	6 months
Dijkhuizen 2001	Micronutrients	Individual	Double blind	Asia	Indonesia	6 months
Dijkhuizen 2001	Micronutrients	Individual	Double blind	Asia	Thailand	6 months
Dijkhuizen 2001	Micronutrients	Individual	Double blind	Asia	Indonesia	6 months
Dijkhuizen 2001	Micronutrients	Individual	Double blind	Asia	Indonesia	6 months
Doherty 1998	Micronutrients	Individual	Double-blind	South East Asia	Bangladesh	30 days
Duggan 2003 - Trial 1	Micronutrients	Individual	Double-blind	South America	Peru	6 months
Duggan 2003 - Trial 2	Micronutrients	Individual	Double-blind	South America	Peru	6 months
Fahmida 2007	Micronutrients	Individual	Double-blind	Asia	Indonesia	6 months
Fink 2017	Maternal education	Cluster	Single-blind	Africa	Zambia	10 months
Gardner 1998	Micronutrients	Individual	Double blind	North America	Jamaica	12 weeks
Giovannini 2006	Micronutrients	Individual	Double blind	Asia	Cambodia	12 months
Goto 2009	Deworming	Individual	Double blind	South East Asia	Bangladesh	36 months
Guldan 2000	Maternal education	Cluster	NR	Asia	China	12 months
Helmizar 2017	Maternal education and micronutrients	Cluster	NR	Asia	Indonesia	6 months
Hess 2015	Micronutrients	Cluster	Partial-blinding	Africa	Burkina Faso	18 months
Husaini 1991	Micronutrients	Cluster	NR	Asia	Indonesia	3 months
Iannotti 2014	Micronutrients	Individual	Double blind	North America	Haiti	6 months
Idjradinata 1993	Micronutrients	Individual	Double blind	Asia	Indonesia	4 months
Idjradinata 1994	Micronutrients	Individual	NR	Asia	Indonesia	4 months
Jack 2012	Micronutrients	Cluster	NR	Asia	Cambodia	18 months
Jones 2015	Micronutrients	Individual	Partial blinding	Africa	Kenya	3 months
Joseph 2015A	Deworming	Individual	Double-blind	South America	Peru	18 months
Krebs 2012	Micronutrients	Cluster	Not blinded	Africa, North America, Asia	Democratic Republic of	12 months

© 2019 Park JJH et al. *JAMA Network Open*.

Trial ID	Intervention domains	Unit of randomization	Blinding	Continents	Countries	Intervention duration (months)
					Congo, Zambia, Guatemala, and Pakistan	
Kupka 2013	Micronutrients	Individual	Non-blinded	Africa	Tanzania	23 months
Kuusipalo 2006	Micronutrients	Individual	Single blind	Africa	Malawi	12 weeks
Lartey 1999	Micronutrients	Individual	Non-blinded	Africa	Ghana	6 months
LCNI-5	Micronutrients	Individual	Single-blind	Africa	Malawi	12 months
Lin 2008	Micronutrients	Individual	Non-blinded	Africa	Malawi	12 months
Lind 2004	Micronutrients	Individual	Double blind	Asia	Indonesia	6 months
Locks 2016	Micronutrients	Double-blind, randomized, placebo-controlled trial	Double-blind	Africa	Tanzania.	18 months
LUCOMA	Maternal education	Cluster	Non-blinded	Africa	Burkina Faso	12 months
Maleta 2015	Micronutrients	Individual	Single blind (participants)	Africa	Malawi	12 months
Mamiro 2004	Micronutrients	Double-blind, randomized, placebo-controlled trial	Double-blind	Africa	Tanzania.	6 months
Maulen-Radovan 1994	Lactose-adapted formula	Individual	NR	North America	Mexico	6 days
Mazariegos 2010	Micronutrients	Individual	Double blind	North America	Guatemala	6 months
Mda 2013	Micronutrients	Individual	Double-blind	Africa	Sub-Saharan Africa	6 months
Medeiros 2015	Micronutrients	Individual	Double-blind	South America	Brazil	3 months
Muhoozi 2017	Maternal education	Cluster	blinded	Africa	Uganda	14 months
Newton 2016	Micronutrients	Individual	Double-blind	Africa	Ghana	5 months
Nurko 1998	Balanced energy protein	Individual	Double-blind	South America	Mexico	16 days
Obatolu 2003	Balanced energy protein	Individual	Single-	Africa	Nigeria	14 monhs
Oelofse 2003	Micronutrients	Individual	NR	Africa	South Africa	6 months
Olney 2006	Micronutrients	Individual	Double blind	Africa	Tanzania	6 months
Osei 2015	Micronutrients	Cluster	NR	Asia	Nepal	11 months
Osendarp 2002	Micronutrients	Individual	Single blind	South East Asia	Bangladesh	12 weeks
Penny 2005	Maternal education	Cluster	Single-blind	South America	Peru	Not reported
Pham 2012	Micronutrients	Cluster	non-blinded	Asia	Vietnam	6 months
Phuka 2009B	Micronutrients	Individual	Single-blinded	Africa	Malawi	3 months
Phuka 2012	Micronutrients	Individual	Single-blinded	Africa	Malawi	12 months
Ramakrishnan 2009	Micronutrients	Individual	Double blind	North America	Mexico	21 months
RDNS	Micronutrients	Cluster	Single-blind	South East Asia	Bangladesh	18 months
Rivera 1998	Micronutrients	Individual	Double blind	North America	Guatemala	6.9 months (4 - 8 months)
Rivera 2001	Micronutrients	Individual	Double-blind	North America	Mexico	12 months
Roy 2007	Maternal education	Cluster	NR	South East Asia	Bangladesh	6 months
Saleem 2014	Maternal education	Cluster	NR	Asia	Pakistan	30 weeks
Santos 2001	Maternal education	Cluster	Single-blind	South America	Brazil	6 months
Santosham 1990	Lactose-adapted formula	Individual	NR	Africa	Egypt	7 days
SEAMTIZI	Micronutrients	Individual	Double-blind	Asia	Thailand, Vietnam, and Indonesia	6 months
Shafique 2016	Micronutrients	Cluster	Single-blind	South East Asia	Bangladesh	6 months

© 2019 Park JJH et al. *JAMA Network Open*.

Trial ID	Intervention domains	Unit of randomization	Blinding	Continents	Countries	Intervention duration (months)
Simondon 1996	Micronutrients	Individual	Non-blinded	Africa, Oceania, South America	Senegal, New Caledonia, Bolivia, Congo	3 months
Skau 2015	Balanced energy protein	Individual	Single blind	Asia	Cambodia	9 months
Smuts 2005A	Micronutrients	Individual	Double-blind	Africa	South Africa	6 months
Sur 2003	Micronutrients	Individual	Double blind	South East Asia	India	12 months
Taneja 2010	Micronutrients	Individual	Double-blind	South East Asia	India	4 months
Tang 2014	Balanced energy protein	Cluster	Single blind	Asia	China	6 months
Thakwalakwa 2012	Micronutrients	Individual	Double-blind	Africa	Malawi	3 months
The JiVitA-4-trial	Micronutrients	Cluster	Non-blinded	South East Asia	Bangladesh	12 months
Thu 1999	Micronutrients	Individual	Double blind	Asia	Vietnam	12 weeks
Umeta 2000	Micronutrients	Individual	Double blind	Africa	Ethiopia	6 months
Untoro 2005	Micronutrients	Individual	Double-blind	South East Asia	Indonesia	5.75 months
Walker 1991	Micronutrients	Individual	NR	North America	Jamaica	12 months
Wasantwisut 2006	Micronutrients	Individual	Double blind	Asia	Thailand	6 months
Williams 2007	Micronutrients	Individual	Double-blind	Africa	Gambia	5 months
Zaman 2008	Maternal education	Cluster	Single-blind	Asia	Pakistan	6 months
Zlotkin 2003	Micronutrients	Randomized trial	Double-blind	Africa	Ghana	2 months

© 2019 Park JJH et al. *JAMA Network Open*.

eTable 17. Patient characteristics for pregnancy life stage review

Trial ID	Country	Intervention domains	Mean gestational age (weeks)
**Preconception**			
Owens 2015	Gambia	Micronutrients	0
PRECONCEPT	Vietnam	Micronutrients	0
**First trimester**			
Katz 2000	Nepal	Micronutrients	3
Asci 2016	Turkey	Lifestyle intervention	7.3
Janmohamed 2016	Cambodia	Balanced energy protein	8.3
JiVitA-3	Bangladesh	Micronutrients	9
MINIMAT	Bangladesh	Micronutrient and Balanced energy protein	9.4
Gowachirapant 2017	India, Thailand	Micronutrients	10.8
Devi 2017	India	Micronutrient and Balanced energy protein	11
Mardones 2007	Chile	Balanced energy protein	11
Zagre 2007	Niger	Micronutrients	11
Christian 2003	Nepal	Micronutrients	11.4
Hanieh 2017	Vietnam	Micronutrients	12
Lui 2013	China	Micronutrients	12
Ramakrishnan 2003	Mexico	Micronutrients	12
Bhutta 2009a	Pakistan	Micronutrients	12.2
Castillo-Duran 2001	Chile	Micronutrients	12.65
Rang-Din Nutrition study	Bangladesh	Micronutrient and Balanced energy protein	12.8
Hafeez 2005	Pakistan	Micronutrients	13
Ahmad 2016	Bangladesh	Micronutrients	13.35
Merialdi 2004	Peru	Micronutrients	13.4
Ziaei 2007	Iran	Micronutrients	13.4
Saaka 2009	Ghana	Micronutrients	13.75
ENID trial	Gambia	Balanced energy protein	13.8
Zeng 2008	China	Micronutrients	13.8
**Second trimester**			
Danesh 2010	Iran	Micronutrients	14
Mojibian 2015	India	Micronutrients	14
Osendarp 2000	Bangladesh	Micronutrients	14
Sayyah-Melli 2016	Iran	Micronutrients	14
Sunawang 2009	Indonesia	Micronutrients	14.4
Dandamrongrak 2016	Thailand	Micronutrients	14.8
Iyengar 1975	India	Micronutrients	15
Villar 2006	Argentina; Egypt; India; Peru; South Africa; Vietnam	Micronutrients	15.1
Zhao 2015	China	Micronutrients	15.85
Caulfield 1999	Peru	Micronutrients	16
Cox 2005	Ghana	Micronutrients	16

© 2019 Park JJH et al. *JAMA Network Open*.

Trial ID	Country	Intervention domains	Mean gestational age (weeks)
Zahiri Sorouri 2016	Iran	Micronutrients	16
Osrin 2005	Nepal	Micronutrients	16.1
Huybregts 2009	Burkina Faso	Balanced energy protein	16.15
Adu-Afarwuah 2015	Ghana	Balanced energy protein	16.3
Dijkhuizen 2004 BC	Indonesia	Micronutrients	16.325
Ashorn 2015A	Malwi	Micronutrient and Balanced energy protein	16.8
Sablok 2015	India	Micronutrients	17
Roberfroid 2008	Burkina Faso	Micronutrients	17.35
Mwangi 2015	Kenya	Micronutrients	17.5
Kumar 2009	India	Micronutrients	17.9
Alizadeh 2016	Iran	Micronutrients	18
Etheredge 2015	Tanzania	Micronutrients	18.2
Ceesay 1997	Gambia	Balanced energy protein	20
Hossain 2014	Pakistan	Micronutrients	20
Lopez-Jaramillo 1997	Ecuador	Micronutrients	20
Purwar 1996	India	Micronutrients	20
Wanchu 2001	India	Micronutrients	20
Goldberg 2013	Gambia	Micronutrients	20.35
Larocque 2006	Peru	Micronutrients and Deworming	20.4
Gonzalez-Casanova 2015	Mexico	Micronutrients	20.5
Belizan 1991	Argentina	Micronutrients	20.8
Fawzi 2007	Tanzania	Micronutrients	21.3
PROFEG	Mozambique	Micronutrients	21.6
Kaestel 2005	Guinea-Bissau	Micronutrients	22
Taherian 2002	Iran	Micronutrients	22.55
Ndyomugyenyi 2008	Uganda	Micronutrients and Deworming	23
Kumwenda 2002	Malwi	Micronutrients	23.25
Whitfield 2016	Cambodia	Micronutrients	23.3
Lopez-Jaramillo 1989	Ecuador	Micronutrients	24
Asemi 2012	Pakistan	Micronutrients	25
Asemi 2013	Iran	Micronutrients	25
Hashemipour 2014	Iran	Micronutrients	25
Diogenes 2013	Brazil	Micronutrients	26
Ndibazza 2011	Uganda	Micronutrients and Deworming	26.6
Satya Deepti 2015	Uganda	Deworming	26.6
Radhika 2003	India	Micronutrients	27
Yu 2008	India	Micronutrients	27
MIDG	Bangladesh	Micronutrients	20
Mohammad-Alizadeh-Charandabi 2015	India	Micronutrients	27.5
AViDD	Bangladesh	Micronutrients	27.75
**Third trimester**			
Friss 2004	Zimbabwe	Micronutrients	29
Menedez 1994	Gambia	Micronutrients	33
Ruangvutilert 2017	Thailand	Micronutrients	33
**Gestational age not reported**			

© 2019 Park JJH et al. *JAMA Network Open*.

Trial ID	Country	Intervention domains	Mean gestational age (weeks)
Asemi 2015	Iran	Micronutrients	NR
Asemi 2016	Iran	Micronutrients	NR
Callaghan-Gillespie 2017	Malawi	Balanced energy protein	NR
CARING trial	India	Community intervention	NR
Fleming 1968	Nigeria	Micronutrients	NR
Kardjati 1988	Indonesia	Balanced energy protein	NR
Marya 1988	India	Micronutrients	NR
ObaapaVitA	Ghana	Micronutrients	NR
SUMMIT 2008	Indonesia	Micronutrients	NR

© 2019 Park JJH et al. *JAMA Network Open*.

eTable 18. Patient characteristics for exclusive breastfeeding life stage review

Trial ID	Average Gestational Age at Enrolment (weeks)	Average Age of Mother (years)	Average proportion of boys	N of children completed the trial
**Adu-Afarwuah 2016**	16.3	26.6	NR	1197
**Ashorn 2015**	NR	25	NR	678
**JiVitA-3**	NR	NR	NR	26935
**RDNS**	31.15	22	NR	3379
**PROMISE EBF Burki Faso**	NR	25.3	0.5	2299
**Feliciano 1994**	NR	NR	NR	219
**Habib 2015**	NR	NR	NR	386
**Hamadani 2001**	NR	NR	0.45	212
**Le Roux 2013**	25.9	25.9	NR	1082
**Locks 2016**	NR	26.4	0.5	2328
**Lonnerdal 2017**	39	29.1	NR	251
**CARING trial**	NR	NR	NR	2683
**LUCOMA**	NR	NR	0.5	1162
**Osendarp 2002**	NR	23.6	0.45	270
**Ostadrahimi 2017**	39.3	26.4	0.67	146
**Simondon 1996**	NR	NR	NR	894
**Goodstart**	NR	23	NR	3494
**Urban 2008**	39.3	27.3	0.52	86
**Vazir 2013**	NR	22.2	0.49	483
**Velaphi 2008**	39.3	NR	0.5	137

© 2019 Park JJH et al. *JAMA Network Open*.

eTable 19. Patient characteristics for complementary feeding life stage review

Trial ID	Age Group of Children Recruited (months)*	Average Child Age (months)**	Average % of Boys
Adu-Afarwuah 2007	6	6	0.5
Alarcon 1991	5 to 24	11.9	1
Alarcon 1992	6 to 24	11.5	1
Ashorn 2015	6	6	NR
Bhan 1988	3 to 24	9.1	0.68
Bhandari 2001	4	16.2	0.47
Black 2004A	6	6.3	0.48
Brooks 2005	2 to 12	5.3	0.52
Brown 1991	3 to 24	12.4	1
Brown 2007	6 to 8	7.5	0.48
CARING trial	6	6	NR
Castillo-Duran 1995	0	0	0.47
Castillo-Duran 2001	0	0	0.51
Chew 1993	15 days to 6	3.7	1
CIGNIS trial	6	6	0.48
Cohen 1994A	4	4	0.44
Dewey 2002	4	4	NR
Doherty 1998	6 to 36	15.5	0.49
Duggan 2003 - Trial 1	6 to12	8.9	0.49
Duggan 2003 - Trial 2	6 to12	8.6	NR
Fahmida 2007	3 to 6	5	0.52
Fayad 1999	3 to 18	8.6	1
Fink 2017	6 to 24	5	0.47
Gardner 1998	6 to 24	13.9	0.43
Giovannini 2006	6	14.1	NR
Goto 2009	<11	6	0.45
Grange 1994	6 to 24	10.3	1
Guldan 2000	4 to 12	NR	NR
Helmizar 2017	6 to 9	6.7	0.54
Hess 2015	9 to 18	NR	NR
Husaini 1991	6 to 20	NR	NR
Iannotti 2014	6 to 11	7.3	0.45
Idjradinata 1993	12 to 18	14.5	0.43
Idjradinata 1994	12 to 18	14.5	0.43
Jack 2012	6	13.2	0.53
Jones 2015	6 to 50	16	0.55
Joseph 2015A	12	12.5	0.52
Krebs 2012	6	6	0.49
Kupka 2013	5 to 7 weeks	1.5	0.54
Kuusipalo 2006	6 to17	13.5	0.35
Lartey 1999	<= 1	6	NR
LCNI-5	6	6	0.5
Lin 2008	6	6	0.47

© 2019 Park JJH et al. *JAMA Network Open*.

Trial ID	Age Group of Children Recruited (months)*	Average Child Age (months)**	Average % of Boys
Lind 2004	<6	6.2	0.52
Locks 2016	5–7 weeks	1.5	0.5
LUCOMA	6	6	NR
Maleta 2015	6	5.9	0.5
Mamiro 2004	6	6	0.5
Maulen-Radovan 1994	5 to 36	11.6	1
Mazariegos 2010	6 to 12	NR	0.53
Mda 2013	4 to 24	12.7	NR
Medeiros 2015	6 to 24	14	0.5
Muhoozi 2017	6 to 8	7.4	NR
Newton 2016	7 to 9	8.3	NR
Nurko 1998	3 to 36	6.4	0.54
Obatolu 2003	4	4	0.5
Oelofse 2003	6	6.2	NR
Olney 2006	5	8.8	0.51
Osei 2015	6 to 9	7.2	0.53
Osendarp 2002	3 to 5 weeks	0.9	0.45
Penny 2005	0	7.3	NR
Pham 2012	5 to 11	5	NR
Phuka 2009B	6 to 18	11.7	0.53
Phuka 2012	18	5.9	0.5
Ramakrishnan 2009	3	3	0.55
RDNS	6	6	NR
Rivera 1998	6 to 9	7.6	0.57
Rivera 2001	8 to 14	12.3	0.5
Roy 2007	6 to 9	NR	NR
Saleem 2014	10 to 20 weeks	3.6	0.62
Santos 2001	<18	NR	NR
Santosham 1990	3 to 18	7.8	0.91
SEAMTIZI	4 to 6	5.2	0.43
Shafique 2016	6	6	NR
Simondon 1996	4	NR	NR
Skau 2015	6	5.3	0.53
Smuts 2005A	6 to 12	8.5	0.5
Sur 2003	0	0	NR
Taneja 2010	6 to 30	15.3	0.52
Tang 2014	6 to 18	6	NR
Thakwalakwa 2012	6 to 18	10.9	0.5
The JiVitA-4-trial	6	6	0.5
Thu 1999	6 to 24	14.2	NR
Umeta 2000	6 to 12	9.4	0.5
Untoro 2005	6 to 12	9.2	0.51
Walker 1991	9 to 24	NR	NR
Wasantwisut 2006	4 to 6	4.5	0.51
Williams 2007	4 to 10	7.9	0.45
Zaman 2008	6 to 24	NR	NR

© 2019 Park JJH et al. *JAMA Network Open*.

Trial ID	Age Group of Children Recruited (months)*	Average Child Age (months)**	Average % of Boys
Zlotkin 2003	6 to 18	10.3	NR

© 2019 Park JJH et al. *JAMA Network Open*.

Risk of bias assessment

eTable 20. Risk of bias assessment for the included studies

Author	Random sequence generation (selection bias)	Allocation concealment (selection bias)	Blinding of participants and personnel (performance bias)	Blinding of outcome assessment (detection bias)	Incomplete outcome data (attrition bias)	Selective reporting (reporting bias)	Other sources of bias
**Pregnancy**							
Adu-Afarwuah 2015	Low	Low	High	Low	Low	Low	Unclear
Ahmad 2016	Unclear	Unclear	Unclear	Unclear	Unclear	Low	Low
Alizadeh 2016	Low	Unclear	Low	Low	High	Low	Low
Asci 2016	Low	Unclear	Unclear	Unclear	Low	Low	Unclear
Asemi 2012	Unclear	Unclear	Unclear	High	Low	Low	Unclear
Asemi 2013	Low	Low	Low	Low	Low	Low	Low
Asemi 2015	Unclear	Unclear	Low	Low	Low	Unclear	Unclear
Asemi 2016	Low	Unclear	Low	Low	Low	Low	Low
Ashorn 2015A	Low	Low	Unclear	Unclear	Low	Low	Unclear
Belizan 1991	Low	Low	Low	Low	Low	Low	Low
Bhutta 2009a	Low	Low	Low	Low	Low	Low	Low
Callaghan-Gillespie 2017	Low	Unclear	Low	Low	Unclear	Low	Unclear
Castillo-Duran 2001	Unclear	Unclear	Unclear	Unclear	Unclear	High	Low
Caulfield 1999	Unclear	Unclear	High	Unclear	High	Unclear	High
Ceesay 1997	Unclear	Unclear	Unclear	Unclear	Unclear	Low	Unclear
Christian 2003	Low	Unclear	Low	Low	Low	Low	Low
Cox 2005	Low	Low	Low	Low	Low	Low	Low
Dandamrongrak 2016	Low	Unclear	Unclear	Unclear	Unclear	Low	Low
Danesh 2010	Unclear	Unclear	Low	Low	Unclear	Low	Unclear
Devi 2017	Unclear	Unclear	High	Unclear	Unclear	Low	Low
Dijkhuizen 2004 BC	Unclear	Unclear	Low	Low	Unclear	Low	Low
Diogenes 2013	Low	High	Unclear	Unclear	Low	Low	Low
Etheredge 2015	Low	Low	Low	Low	Low	Low	Low
Fawzi 2007	Low	Low	Low	Low	Low	Low	Low
Fleming 1968	Unclear	Unclear	Low	Low	Low	Low	Low
Friss 2004	Low	Low	High	Low	Low	Low	Low
Goldberg 2013	Unclear	Unclear	Low	Low	Low	Low	Low
Gonzalez-Casanova 2015	Unclear	Unclear	Low	Low	Low	Low	Unclear
Gowachirapant 2017	Low	Low	Low	Low	Low	Low	Low
Hafeez 2005	Low	Unclear	Low	Low	Unclear	Low	Low
Hanieh 2017	Unclear	Unclear	Low	Low	Unclear	Low	Unclear
Hashemipour 2014	Low	Low	High	High	Unclear	Low	Low
Hemminki 2016	Low	Low	Unclear	Unclear	Low	Low	Low
Hossain 2014	Low	Unclear	Low	Low	Low	Low	Low
Huybregts 2009	Low	Low	High	Low	Low	Low	Unclear
Iyengar 1975	High	Unclear	Unclear	Low	Low	Low	Unclear
Janmohamed 2016	Low	Low	Unclear	Low	Low	Low	Unclear
Johnson 2017	Unclear	Unclear	High	Low	Unclear	Unclear	Unclear
Kaestel 2005	Low	Unclear	Low	Low	Low	Low	Low
Kardjati 1988	Low	Unclear	High	High	Unclear	Low	Low

© 2019 Park JJH et al. *JAMA Network Open*.

Author	Random sequence generation (selection bias)	Allocation concealment (selection bias)	Blinding of participants and personnel (performance bias)	Blinding of outcome assessment (detection bias)	Incomplete outcome data (attrition bias)	Selective reporting (reporting bias)	Other sources of bias
Katz 2000	Unclear	Unclear	Unclear	Low	Low	Low	Unclear
Kirkwood 2010	Low	Low	Low	Low	Low	Low	Unclear
Kumar 2009	Low	Low	Low	Low	Low	Low	Low
Kumwenda 2002	Low	Low	Unclear	Unclear	Unclear	Low	Low
Larocque 2006	Low	Unclear	Low	Low	Low	Low	Low
Lopez-Jaramillo 1989	Low	Low	Low	Low	Unclear	Unclear	Low
Lopez-Jaramillo 1997	Low	Low	Low	Low	Low	Low	Low
Lui 2013	Low	Low	Low	Low	Low	Low	Unclear
Mardones 2007	Unclear	Unclear	High	High	Low	Low	Low
Marya 1988	Unclear	Unclear	Unclear	Unclear	Low	Low	Low
Menedez 1994	Unclear	High	High	Low	Unclear	Unclear	Unclear
Merialdi 2004	Low	Low	Low	Low	Low	Low	Low
Mohammad-Alizadeh-Charandabi 2015	Low	Low	Low	Low	Unclear	Low	Unclear
Mojibian 2015	Low	Unclear	High	High	Unclear	Low	Low
Mridha 2016	Low	Unclear	High	Low	Low	Low	Unclear
Mwangi 2015	Low	Low	Low	Low	Low	Low	Low
Ndibazza 2011	Low	Low	Low	Low	Low	Low	Low
Ndyomugyenyi 2008	Low	Unclear	Unclear	Unclear	Unclear	Low	Unclear
Osendarp 2000	Low	Unclear	Low	Unclear	Unclear	Low	Low
Osrin 2005	Low	Low	High	Low	Low	Low	Low
Owens 2015	Low	Unclear	Low	Low	Low	Low	Low
Persson 2012	Low	Unclear	Unclear	Low	Low	Low	Low
Purwar 1996	Low	Low	Low	Low	Low	Unclear	Low
Radhika 2003	Unclear	Unclear	Low	Low	Low	Low	Low
Ramakrishnan 2003	Low	Low	Low	Low	Low	Low	Unclear
Ramakrishnan 2016A	Low	Low	Low	Low	Low	Low	Low
Roberfroid 2008	Low	Low	Low	Low	Unclear	Low	Low
Roth 2010	Low	Low	Low	Low	Low	Low	Low
Ruangvutilert 2017	Low	Low	Unclear	Unclear	Unclear	Low	Low
Saaka 2009	Low	Low	Low	Unclear	Unclear	Low	Low
Sablok 2015	Low	Unclear	Unclear	Unclear	Unclear	Unclear	Low
Satya Deepti 2015	Low	Low	Low	Low	Low	Low	Low
Sayyah-Melli 2016	Low	Low	High	Low	Low	Unclear	Low
SUMMIT 2008	Low	Low	Low	Low	Low	Low	Unclear
Sunawang 2009	Unclear	Unclear	Low	Low	Low	Unclear	Unclear
Taherian 2002	Low	Unclear	High	High	Low	Low	Low
Villar 2006	Low	Low	Low	Low	Low	Low	Low
Wanchu 2001	Unclear	Unclear	High	High	High	Unclear	Low
West 2014	Low	Unclear	Unclear	Low	Low	Low	Unclear
Whitfield 2016	Low	Unclear	Low	Low	Low	Low	Unclear
Yu 2008	Low	High	High	Unclear	Low	Low	Low
Zagre 2007	Unclear	Unclear	Unclear	Low	Low	Low	Low
Zahiri Sorouri 2016	Low	Unclear	Unclear	Low	Low	Low	Low
Zeng 2008	Low	Low	Low	Low	Low	Low	Low

© 2019 Park JJH et al. *JAMA Network Open*.

Author	Random sequence generation (selection bias)	Allocation concealment (selection bias)	Blinding of participants and personnel (performance bias)	Blinding of outcome assessment (detection bias)	Incomplete outcome data (attrition bias)	Selective reporting (reporting bias)	Other sources of bias
Zhao 2015	Low	Unclear	Low	Unclear	Low	Low	Low
Ziaei 2007	Low	Unclear	Low	Low	Unclear	Low	Low
**Exclusive breastfeeding period: 0-6 months**							
Acharya 2014	Low	Unclear	High	Unclear	Low	Low	Low
Adu-Afarwuah 2016	Low	Low	High	Low	Low	High	Unclear
Boo 2007	Low	Low	High	Unclear	High	Low	Low
Christian 2016	Low	High	Low	Low	Low	Low	Unclear
Engebretsen 2014	Unclear	Unclear	Unclear	Unclear	Low	Low	Unclear
Feliciano 1994	Unclear	Unclear	Unclear	Unclear	Unclear	Unclear	Unclear
Gathwala 2010	Low	Unclear	High	High	Low	Low	High
Habib 2015	Low	Low	Low	Low	Low	Low	Low
Hamadani 2001	Unclear	Unclear	Low	Low	Unclear	Low	Low
Kumbhojkar 2016	Low	Low	High	Unclear	High	Low	Low
Le Roux 2013	Low	Low	Unclear	Low	Low	Unclear	Unclear
Lonnerdal 2017	Unclear	Unclear	Low	Low	Unclear	Unclear	Unclear
Osendarp 2002	Low	Unclear	Unclear	Low	Low	Low	Unclear
Ostadrahimi 2017	Low	Low	Low	Low	Low	Low	Low
Roth 2018	Low	Low	Low	Low	Low	Low	Unclear
Simondon 1996	Low	Unclear	Unclear	Low	Low	Low	Unclear
Suman 2008	Low	Low	High	Unclear	High	Low	Unclear
Tomlinson 2014	Low	Unclear	Unclear	Unclear	Low	Low	Unclear
Urban 2008	Low	Unclear	Low	Low	Low	Low	Unclear
Vazir 2013	Low	Unclear	Unclear	Low	Low	Low	Unclear
Velaphi 2008	Unclear	Unclear	Low	Low	Unclear	Low	Low
**Complementary feeding period: 6-24 months old**							
Adu-Afarwuah 2007	Unclear	Low	High	Low	Low	Low	Unclear
Black 2004A	High	High	Low	Low	High	Low	Unclear
Brown 2007	Low	Low	Low	Low	Low	Unclear	Low
Castillo-Duran 1995	Unclear	Unclear	Low	Low	Unclear	High	Low
Chilenje Infant Growth 2010	Low	Low	Low	Low	Low	Low	Unclear
Christian 2015	Low	High	High	High	Low	Low	Unclear
Cohen 1994A	Unclear	Unclear	Unclear	Unclear	Unclear	Low	Unclear
Dewey 2002	Unclear	Unclear	Low	Low	Low	Low	Unclear
Dewey 2017	Unclear	Unclear	High	Low	Low	Low	High
Dijkhuizen 2001	Low	Low	Low	Low	High	Low	Unclear
Dijkhuizen 2008	Low	Unclear	Low	Low	Unclear	Low	Low
Doherty 1998	Low	Low	Low	Low	Unclear	Unclear	Unclear
Duggan 2003	Low	Low	Unclear	Unclear	Low	Low	Low
Fahmida 2007	Low	Low	Low	Low	Low	Low	Low
Fink 2017	Unclear	Unclear	High	High	Unclear	Low	Unclear
Giovannini 2006	Low	Low	Low	Low	Low	Unclear	Low
Goto 2009	Low	Unclear	Low	Low	Low	Low	Unclear
Helmizar 2017	Low	Unclear	Unclear	Unclear	Low	Unclear	Unclear
Hess 2015	Low	Unclear	High	High	Low	Low	Unclear

© 2019 Park JJH et al. *JAMA Network Open*.

Author	Random sequence generation (selection bias)	Allocation concealment (selection bias)	Blinding of participants and personnel (performance bias)	Blinding of outcome assessment (detection bias)	Incomplete outcome data (attrition bias)	Selective reporting (reporting bias)	Other sources of bias
Iannotti 2014	Low	Low	Unclear	Unclear	Low	Low	Unclear
Idjradinata 1993	Unclear	Unclear	Unclear	Unclear	Unclear	Low	Low
Idjradinata 1994	Low	Unclear	Unclear	Unclear	Unclear	Unclear	Unclear
Jack 2012	Unclear	Unclear	Unclear	Unclear	Unclear	Low	Unclear
Jones 2015	Unclear	Unclear	High	High	Low	Low	Low
Joseph 2015A	Low	Low	Low	Low	Low	Low	Low
Krebs 2012	Low	Unclear	High	High	Low	Low	Unclear
Kupka 2013	Low	Low	Low	Low	Low	Unclear	Unclear
Kuusipalo 2006	Low	Unclear	Unclear	Low	Low	Unclear	Unclear
Lartey 1999	Low	Low	Unclear	Unclear	Low	Low	Unclear
Lin 2008	Unclear	Unclear	Unclear	Unclear	Low	High	Unclear
Lind 2004	Unclear	Low	Low	Low	Low	Low	Low
Locks 2016	Low	Low	Low	Low	Unclear	High	Unclear
Luby 2018	Low	Unclear	High	Low	Low	Low	Unclear
Maleta 2015	Low	Low	High	Low	Low	Low	Low
Mamiro 2004	Low	Unclear	Low	Low	Low	Low	Low
Mangani 2015	Low	Low	High	Low	Low	Low	Low
Mazariegos 2010	Low	Low	Low	Low	Low	High	Low
Mda 2013	Low	Low	Low	Low	Low	Low	Unclear
Medeiros 2015	Low	Unclear	Low	Low	Low	Low	Unclear
Muhoozi 2017	Low	Unclear	Unclear	Low	Low	Low	Unclear
Nair 2017	High	High	High	Low	Low	High	High
Newton 2016	Low	Unclear	Low	Low	Low	Low	Low
Nikiema 2017	Unclear	High	High	Low	Low	Low	Unclear
Null 2018	Low	High	High	Low	Low	Low	Unclear
Oelofse 2003	Low	Unclear	Unclear	Unclear	Unclear	Low	Unclear
Olney 2006	Unclear	Unclear	Low	Low	Low	Low	Unclear
Osei 2015	Low	Unclear	High	Low	Low	Low	Unclear
Pham 2012	Unclear	Unclear	Unclear	Unclear	Unclear	Low	Unclear
Phuka 2009A	Low	Low	Unclear	Low	Low	Low	Unclear
Phuka 2012	Unclear	Unclear	Unclear	Unclear	Unclear	Unclear	Low
Ramakrishnan 2009	Low	Unclear	Low	Low	Low	Low	Unclear
Rivera 1998	Unclear	Unclear	Low	Unclear	Unclear	Low	Unclear
Rivera 2001	Low	Unclear	Low	Unclear	Unclear	Unclear	Unclear
Saleem 2014	Low	Unclear	Unclear	Unclear	Unclear	Low	Unclear
Shafique 2016	Low	High	High	Low	Low	Low	Unclear
Skau 2015	Low	Unclear	Unclear	Low	Low	Low	Unclear
Smuts 2005A	Unclear	Unclear	Low	Unclear	Unclear	Low	Low
Taneja 2010	Unclear	Unclear	Low	Unclear	Unclear	Unclear	Low
Tang 2014	Unclear	Unclear	Unclear	Unclear	Unclear	Low	Unclear
Thakwalakwa 2012	Low	Low	High	High	Low	Low	Unclear
Thu 1999	Low	Low	Unclear	Unclear	Low	Unclear	Low
Umeta 2000	Unclear	Unclear	Low	Low	Low	High	Low
Untoro 2005	Low	Unclear	Unclear	Unclear	Low	Low	Unclear

© 2019 Park JJH et al. *JAMA Network Open*.

Author	Random sequence generation (selection bias)	Allocation concealment (selection bias)	Blinding of participants and personnel (performance bias)	Blinding of outcome assessment (detection bias)	Incomplete outcome data (attrition bias)	Selective reporting (reporting bias)	Other sources of bias
Wasantwisut 2006	Low	Low	Low	Low	Low	Low	Low
Williams 2007	Low	Unclear	Low	Unclear	Unclear	Low	Unclear
Zlotkin 2003	Low	Unclear	High	Unclear	Low	Low	Unclear

© 2019 Park JJH et al. *JAMA Network Open*.

Markov chain Monte Carlo summary statistics

eTable 21. MCMC summary for preterm birth NMA

Parameter	mean	sd	2.50%	25%	50%	75%	97.50%	Rhat	n.eff
Folic Acid trt. effect	-0.5	0.33	-1.22	-0.71	-0.49	-0.28	0.12	1	4000
Calcium trt. effect	-0.28	0.14	-0.57	-0.37	-0.28	-0.19	-0.02	1	4700
Calcium+VitD trt. effect	0.31	0.31	-0.29	0.1	0.31	0.52	0.92	1	10000
Iron trt. effect	-0.61	0.27	-1.18	-0.78	-0.6	-0.42	-0.11	1	1100
VitA trt. effect	-0.61	0.36	-1.38	-0.84	-0.59	-0.36	0.06	1	2500
VitD trt. effect	-0.38	0.29	-0.96	-0.56	-0.37	-0.18	0.17	1	10000
Zinc trt. effect	-0.65	0.31	-1.28	-0.85	-0.63	-0.44	-0.08	1	1200
Folic Acid+VitA trt. effect	-0.6	0.39	-1.4	-0.85	-0.59	-0.34	0.12	1	3300
Iron+Zinc trt. effect	-0.59	0.34	-1.27	-0.81	-0.58	-0.36	0.05	1	1800
IFA trt. effect	-0.53	0.32	-1.22	-0.73	-0.52	-0.32	0.06	1	4100
IFA+VitA trt. effect	-0.63	0.39	-1.43	-0.88	-0.62	-0.37	0.11	1	3700
IFA+Zinc trt. effect	-0.15	0.42	-1	-0.43	-0.15	0.13	0.67	1	10000
IFA+Zinc+VitA trt. effect	-0.6	0.39	-1.4	-0.85	-0.59	-0.34	0.14	1	3100
MMN trt. effect	-0.62	0.32	-1.3	-0.82	-0.61	-0.41	-0.03	1	2700
Deworming 1dose trt. effect	-0.17	0.7	-1.57	-0.63	-0.16	0.3	1.17	1	1500
Deworming 1dose+Iron trt. effect	-0.86	0.49	-1.88	-1.18	-0.85	-0.53	0.07	1	4800
Deworming 2dose trt. effect	-0.07	0.82	-1.79	-0.59	-0.03	0.5	1.43	1.01	480
Iron+Calcium+VitD trt. effect	-1.64	1.01	-3.56	-2.36	-1.63	-0.93	0.34	1	600
Iron+Calcium trt. effect	-1.85	0.9	-3.62	-2.49	-1.83	-1.21	-0.13	1	490
Flour 750kcal fort trt. effect	-1.41	0.99	-3.42	-2.07	-1.39	-0.73	0.5	1	8000
Milk 261-575kcal trt. effect	0.08	1.12	-2.12	-0.67	0.09	0.84	2.29	1	3300
Milk 261-575kcal+VitB12 trt. effect	-1.29	1.29	-3.65	-2.25	-1.29	-0.39	1.27	1	990
LNS20 trt. effect	-0.56	0.36	-1.32	-0.8	-0.55	-0.32	0.13	1	3800
LNS20 fort trt. effect	-0.59	0.39	-1.38	-0.84	-0.58	-0.34	0.15	1	7500
LNS72 fort trt. effect	-0.46	0.38	-1.24	-0.71	-0.45	-0.2	0.28	1	5100
LNS135 trt. effect	-1.47	0.86	-3.32	-2.03	-1.42	-0.86	0.09	1	2300
LNS135 fort trt. effect	-0.72	0.69	-2.15	-1.18	-0.69	-0.24	0.54	1	3200
Iodine trt. effect	-0.33	0.39	-1.11	-0.58	-0.33	-0.06	0.44	1	10000
Iodine+Iron trt. effect	-0.75	0.58	-1.88	-1.13	-0.74	-0.36	0.37	1	2200
Regression coefficient	0.42	0.26	-0.05	0.25	0.41	0.59	0.97	1	1700
Precision	93.26	94.76	16.74	40.07	66.35	111	338.2	1	1700

© 2019 Park JJH et al. *JAMA Network Open*.

eTable 22. MCMC summary for mean birthweight NMA

Parameter	mean	sd	2.50%	25%	50%	75%	97.50%	Rhat	n.eff
Folic Acid trt. effect	0.05	0.04	-0.03	0.02	0.05	0.08	0.14	1	1200
Calcium trt. effect	0.03	0.02	-0.01	0.02	0.03	0.04	0.07	1	10000
Calcium+VitD trt. effect	0.11	0.04	0.04	0.09	0.11	0.14	0.18	1	8900
Iron trt. effect	0.09	0.03	0.03	0.07	0.09	0.11	0.15	1	890
VitA trt. effect	0.01	0.05	-0.09	-0.02	0.01	0.04	0.1	1	1100
VitB1 trt. effect	-0.02	0.13	-0.28	-0.11	-0.02	0.06	0.23	1	1300
VitD trt. effect	-0.01	0.03	-0.06	-0.02	-0.01	0.01	0.05	1	10000
Zinc trt. effect	0.12	0.03	0.06	0.1	0.12	0.14	0.18	1	3200
Folic Acid+VitA trt. effect	0.01	0.06	-0.1	-0.03	0.01	0.05	0.13	1	840
Iron+Zinc trt. effect	0.08	0.05	0	0.05	0.08	0.11	0.17	1	890
IFA trt. effect	0.05	0.04	-0.04	0.02	0.05	0.07	0.13	1	1100
IFA+VitA trt. effect	0.11	0.05	0.01	0.08	0.11	0.15	0.21	1	1700
IFA+Zinc trt. effect	0.03	0.05	-0.08	-0.01	0.03	0.06	0.13	1	1400
IFA+Zinc+VitA trt. effect	0	0.06	-0.11	-0.04	0	0.04	0.11	1	790
MMN trt. effect	0.08	0.04	0	0.06	0.08	0.11	0.17	1	910
MMN+VitD trt. effect	-0.09	0.08	-0.24	-0.14	-0.08	-0.03	0.06	1	1900
Deworming 1dose trt. effect	0.02	0.02	-0.02	0.01	0.02	0.04	0.07	1	10000
Deworming 1dose+Iron trt. effect	0.1	0.05	0	0.07	0.1	0.13	0.2	1	1500
Deworming 2dose trt. effect	-0.02	0.05	-0.12	-0.05	-0.02	0.01	0.07	1	9000
Iron+Calcium+VitD trt. effect	0.09	0.1	-0.11	0.02	0.09	0.16	0.29	1	1300
Iron+Calcium trt. effect	0.15	0.07	0.02	0.11	0.15	0.19	0.28	1	1100
Flour 750kcal fort trt. effect	0.05	0.07	-0.07	0.01	0.05	0.1	0.18	1	10000
Milk 261-575kcal fort trt. effect	0.03	0.06	-0.09	-0.01	0.03	0.06	0.14	1	9800
Milk 261-575kcal trt. effect	-0.04	0.04	-0.12	-0.07	-0.04	-0.01	0.04	1	4400
Milk 261-575kcal+VitB12 trt. effect	-0.19	0.09	-0.36	-0.25	-0.19	-0.13	-0.02	1	3800
Local food 597kcal-850kcal fort trt. effect	0.08	0.05	-0.03	0.04	0.08	0.11	0.18	1	1300
LNS20 trt. effect	0.11	0.05	0.02	0.08	0.11	0.15	0.21	1	1200
LNS20 fort trt. effect	0.09	0.06	-0.02	0.05	0.09	0.12	0.2	1	1600
LNS72 fort trt. effect	0.1	0.06	-0.02	0.06	0.1	0.14	0.21	1	2100
Maternal Education trt. effect	0.04	0.08	-0.12	-0.02	0.04	0.1	0.2	1	7200
Iodine+Iron trt. effect	0.06	0.07	-0.08	0.01	0.05	0.1	0.19	1	2500
DHA trt. effect	0.05	0.04	-0.03	0.03	0.05	0.08	0.13	1	10000
Home Visits+Peer Counseling trt. effect	-0.06	0.05	-0.15	-0.09	-0.06	-0.03	0.03	1	6400
Regression coefficient	-0.19	0.05	-0.28	-0.22	-0.19	-0.15	-0.09	1	2800
Precision	1667.54	1086.45	563.43	1026	1415	1990.75	4187.82	1	3300

© 2019 Park JJH et al. *JAMA Network Open*.

eTable 23. MCMC summary for length-for-age (LAZ) NMA

Parameter	mean	sd	2.50%	25%	50%	75%	97.50%	Rhat	n.eff
MMN M trt. effect	-0.02	0.08	-0.18	-0.07	-0.02	0.02	0.14	1	10000
MMN C trt. effect	0.2	0.08	0.03	0.15	0.2	0.24	0.35	1	8200
LNS 20 M trt. effect	-0.01	0.08	-0.17	-0.05	-0.01	0.04	0.16	1	10000
IFA M trt. effect	0.04	0.1	-0.15	-0.01	0.05	0.1	0.23	1	10000
IFA+LNS 20 M trt. effect	0.08	0.1	-0.13	0.03	0.08	0.14	0.29	1	10000
Zinc 5mg C trt. effect	0.12	0.07	-0.03	0.08	0.12	0.16	0.24	1	3300
Formula 335kcal C trt. effect	0.05	0.13	-0.2	-0.03	0.05	0.13	0.3	1	10000
Formula+OPN 65 C trt. effect	-0.13	0.12	-0.37	-0.21	-0.13	-0.05	0.11	1	10000
Formula+OPN 130 C trt. effect	-0.06	0.12	-0.3	-0.14	-0.06	0.02	0.17	1	5300
Maternal education M trt. effect	0.05	0.09	-0.12	0	0.05	0.1	0.23	1	4800
Peer counseling M trt. effect	-0.05	0.07	-0.18	-0.09	-0.05	-0.01	0.07	1	9400
Individual counseling M trt. effect	0.06	0.1	-0.14	0	0.06	0.12	0.26	1	10000
VitD M trt. effect	0.08	0.09	-0.1	0.02	0.08	0.14	0.27	1	10000
Precision	681.86	1023.75	37.76	147.9	318.45	761.97	3632.72	1	5100

© 2019 Park JJH et al. *JAMA Network Open*.

eTable 24. MCMC summary for height-for-age (HAZ) NMA

Parameter	mean	sd	2.50%	25%	50%	75%	97.50%	Rhat	n.eff
IFA trt. effect	0.18	0.06	0.05	0.14	0.18	0.22	0.3	1	10000
IFA+Zinc trt. effect	0.11	0.09	-0.07	0.05	0.11	0.17	0.29	1	10000
Iron trt. effect	0.05	0.05	-0.04	0.02	0.05	0.08	0.15	1	4700
Iron+VitA trt. effect	0.11	0.08	-0.06	0.06	0.11	0.16	0.26	1	3300
Iron+VitB2 trt. effect	0.33	0.25	-0.17	0.15	0.32	0.49	0.81	1	4600
Iron+Zinc trt. effect	-0.04	0.05	-0.13	-0.08	-0.04	-0.01	0.05	1	3000
Iron+Zinc+VitA trt. effect	-0.01	0.12	-0.24	-0.09	-0.01	0.07	0.23	1	10000
Iron+Zinc+VitB2 trt. effect	0.13	0.26	-0.38	-0.05	0.13	0.31	0.65	1	1200
Zinc trt. effect	-0.02	0.03	-0.08	-0.04	-0.02	0	0.04	1	10000
Zinc+VitB2 trt. effect	-0.07	0.26	-0.58	-0.24	-0.07	0.1	0.42	1	10000
Zinc+VitBcomplex+C+E trt. effect	0.05	0.06	-0.09	0.01	0.05	0.09	0.16	1	10000
VitB2 trt. effect	0.33	0.25	-0.16	0.16	0.33	0.49	0.81	1	2800
VitBcomplex+C+E trt. effect	-0.04	0.06	-0.17	-0.08	-0.04	0	0.07	1	1800
MMN weekly trt. effect	0.09	0.14	-0.19	0	0.09	0.19	0.37	1	700
MMN trt. effect	0.14	0.06	0.02	0.1	0.14	0.18	0.25	1	1600
Maternal education trt. effect	-0.11	0.07	-0.26	-0.15	-0.1	-0.06	0.03	1	10000
Antibiotics+Deworming 1dose trt. effect	-0.04	0.13	-0.28	-0.12	-0.04	0.05	0.21	1	10000
Deworming 1dose trt. effect	-0.01	0.06	-0.13	-0.05	-0.01	0.02	0.1	1	10000
Deworming 2doses trt. effect	0.01	0.06	-0.12	-0.03	0.01	0.05	0.13	1	8300
Porridge 185-340kcal trt. effect	-0.02	0.08	-0.18	-0.07	-0.02	0.03	0.14	1	3500
Porridge 185-340kcal fort trt. effect	0.05	0.07	-0.09	0.01	0.05	0.1	0.19	1	9000
Flour 270-340kcal trt. effect	0.05	0.07	-0.08	0.01	0.05	0.1	0.19	1	1500
Flour 270-340kcal fort trt. effect	0.19	0.26	-0.33	0.01	0.19	0.36	0.69	1	5100
LNS 23-55kcal fort trt. effect	0.05	0.07	-0.09	0.01	0.05	0.1	0.19	1	10000
LNS 118-130kcal fort trt. effect	-0.03	0.04	-0.11	-0.06	-0.03	-0.01	0.04	1	10000
LNS 118kcal trt. effect	-0.04	0.08	-0.19	-0.08	-0.04	0.01	0.11	1	8000
LNS 220-285kcal fort trt. effect	-0.01	0.04	-0.09	-0.04	-0.01	0.02	0.07	1	2800
Local food 185-260kcal trt. effect	0.04	0.07	-0.1	-0.01	0.04	0.08	0.18	1	10000
Water treatment trt. effect	-0.1	0.06	-0.22	-0.14	-0.09	-0.05	0.03	1	10000
Toilet facilities trt. effect	-0.07	0.06	-0.2	-0.11	-0.07	-0.03	0.05	1	7400
Handwashing trt. effect	-0.05	0.06	-0.18	-0.09	-0.05	-0.01	0.07	1	10000
WASH combined trt. effect	-0.07	0.06	-0.19	-0.11	-0.06	-0.02	0.06	1	10000
WASH combined+LNS 118kcal fort trt. effect	0.02	0.06	-0.1	-0.02	0.02	0.06	0.14	1	10000
Precision	20907757.9	1578167094	123.4	343.2	766.8	2642.75	255984.98	1.01	340
Regression coefficient	0.03	0.02	-0.01	0.01	0.02	0.04	0.08	1	1300

© 2019 Park JJH et al. *JAMA Network Open*.

© 2019 Park JJH et al. *JAMA Network Open*.

eReference 1:

eReference 1 includes all of references cited in eTables.

1. Imdad A, Bhutta ZA. Effect of balanced protein energy supplementation during pregnancy on birth outcomes. *BMC Public Health* 2011; **11 Suppl 3**: S17.

2. Imdad A, Bhutta ZA. Maternal nutrition and birth outcomes: effect of balanced protein-energy supplementation. *Paediatr Perinat Epidemiol* 2012; **26 Suppl 1**: 178-90.

3. Liberato SC, Singh G, Mulholland K. Effects of protein energy supplementation during pregnancy on fetal growth: a review of the literature focusing on contextual factors. *Food Nutr Res* 2013; **57**.

4. Stevens B, Buettner P, Watt K, Clough A, Brimblecombe J, Judd J. The effect of balanced protein energy supplementation in undernourished pregnant women and child physical growth in low- and middle-income countries: a systematic review and meta-analysis. *Matern Child Nutr* 2015; **11**(4): 415-32.

5. Buppasiri P, Lumbiganon P, Thinkhamrop J, Ngamjarus C, Laopaiboon M, Medley N. Calcium supplementation (other than for preventing or treating hypertension) for improving pregnancy and infant outcomes. *The Cochrane Library* 2015.

6. Hofmeyr GJ, Lawrie TA, Atallah AN, Duley L, Torloni MR. Calcium supplementation during pregnancy for preventing hypertensive disorders and related problems. *Cochrane Database Syst Rev* 2014; (6): CD001059.

7. Bassani DG, Arora P, Wazny K, Gaffey MF, Lenters L, Bhutta ZA. Financial incentives and coverage of child health interventions: a systematic review and meta-analysis. *BMC Public Health* 2013; **13 Suppl 3**: S30.

8. Glassman A, Duran D, Fleisher L, et al. Impact of conditional cash transfers on maternal and newborn health. *J Health Popul Nutr* 2013; **31**(4 Suppl 2): 48-66.

9. Salam RA, Haider BA, Humayun Q, Bhutta ZA. Effect of administration of antihelminthics for soil-transmitted helminths during pregnancy. *Cochrane Database Syst Rev* 2015; (6): CD005547.

10. Lassi ZS, Salam RA, Haider BA, Bhutta ZA. Folic acid supplementation during pregnancy for maternal health and pregnancy outcomes. *Cochrane Database Syst Rev* 2013; (3): CD006896.

11. Yang Z, Huffman SL. Review of fortified food and beverage products for pregnant and lactating women and their impact on nutritional status. *Maternal & child nutrition* 2011; **7**: 19-43.

© 2019 Park JJH et al. *JAMA Network Open*.

12. Bratton KN, Wardle MT, Orenstein WA, Omer SB. Maternal influenza immunization and birth outcomes of stillbirth and spontaneous abortion: a systematic review and meta-analysis. *Clin Infect Dis* 2015; **60**(5): e11-9.

13. Nunes MC, Aqil AR, Omer SB, Madhi SA. The Effects of Influenza Vaccination during Pregnancy on Birth Outcomes: A Systematic Review and Meta-Analysis. *Am J Perinatol* 2016; **33**(11): 1104-14.

14. Pena-Rosas JP, Viteri FE. Effects and safety of preventive oral iron or iron+folic acid supplementation for women during pregnancy. *Cochrane Database Syst Rev* 2009; (4): CD004736.

15. Suchdev P, Peña-Rosas J, De-Regil L. Multiple micronutrient powders for home (point-of-use) fortification of foods in pregnant women. *Cochrane Database of Systematic Reviews* 2014; **6**.

16. Haider BA, Bhutta ZA. Multiple-micronutrient supplementation for women during pregnancy. *Cochrane Database Syst Rev* 2017; **4**: CD004905.

17. Imhoff-Kunsch B, Briggs V, Goldenberg T, Ramakrishnan U. Effect of n-3 long-chain polyunsaturated fatty acid intake during pregnancy on maternal, infant, and child health outcomes: a systematic review. *Paediatr Perinat Epidemiol* 2012; **26 Suppl 1**: 91-107.

18. Thorne-Lyman AL, Fawzi WW. Vitamin A and carotenoids during pregnancy and maternal, neonatal and infant health outcomes: a systematic review and meta-analysis. *Paediatr Perinat Epidemiol* 2012; **26 Suppl 1**: 36-54.

19. De-Regil LM, Palacios C, Lombardo LK, Pena-Rosas JP. Vitamin D supplementation for women during pregnancy. *Cochrane Database Syst Rev* 2016; (1): CD008873.

20. Perez-Lopez FR, Pasupuleti V, Mezones-Holguin E, et al. Effect of vitamin D supplementation during pregnancy on maternal and neonatal outcomes: a systematic review and meta-analysis of randomized controlled trials. *Fertil Steril* 2015; **103**(5): 1278-88 e4.

21. Thorne-Lyman A, Fawzi WW. Vitamin D during pregnancy and maternal, neonatal and infant health outcomes: a systematic review and meta-analysis. *Paediatr Perinat Epidemiol* 2012; **26 Suppl 1**: 75-90.

22. Ota E, Mori R, Middleton P, et al. Zinc supplementation for improving pregnancy and infant outcome. *Cochrane Database Syst Rev* 2015; (2): CD000230.

© 2019 Park JJH et al. *JAMA Network Open*.

23. Kramer MS, Kakuma R. Optimal duration of exclusive breastfeeding. *Cochrane Database Syst Rev* 2012; (8): CD003517.

24. Lumbiganon P, Martis R, Laopaiboon M, Festin MR, Ho JJ, Hakimi M. Antenatal breastfeeding education for increasing breastfeeding duration. *Cochrane Database Syst Rev* 2016; **12**: CD006425.

25. Haroon S, Das JK, Salam RA, Imdad A, Bhutta ZA. Breastfeeding promotion interventions and breastfeeding practices: a systematic review. *BMC Public Health* 2013; **13 Suppl 3**: S20.

26. Balogun OO, O'Sullivan EJ, McFadden A, et al. Interventions for promoting the initiation of breastfeeding. *Cochrane Database Syst Rev* 2016; **11**: CD001688.

27. Giugliani ER, Horta BL, Loret de Mola C, Lisboa BO, Victora CG. Effect of breastfeeding promotion interventions on child growth: a systematic review and meta-analysis. *Acta Paediatr* 2015; **104**(467): 20-9.

28. Abe SK, Balogun OO, Ota E, Takahashi K, Mori R. Supplementation with multiple micronutrients for breastfeeding women for improving outcomes for the mother and baby. *Cochrane Database Syst Rev* 2016; **2**: CD010647.

29. Ndikom CM, Fawole B, Ilesanmi RE. Extra fluids for breastfeeding mothers for increasing milk production. *Cochrane Database Syst Rev* 2014; (6): CD008758.

30. Martin CR, Ling PR, Blackburn GL. Review of Infant Feeding: Key Features of Breast Milk and Infant Formula. *Nutrients* 2016; **8**(5).

31. Fleith M, Clandinin MT. Dietary PUFA for Preterm and Term Infants: Review of Clinical Studies. *Critical Reviews in Food Science and Nutrition* 2005; **45**(3): 205-29.

32. Conde-Agudelo A, Diaz-Rossello JL. Kangaroo mother care to reduce morbidity and mortality in low birthweight infants. *Cochrane Database Syst Rev* 2016; (8): CD002771.

33. Moore ER, Bergman N, Anderson GC, Medley N. Early skin-to-skin contact for mothers and their healthy newborn infants. *Cochrane Database Syst Rev* 2016; **11**: CD003519.

34. Delgado-Noguera MF, Calvache JA, Bonfill Cosp X, Kotanidou EP, Galli-Tsinopoulou A. Supplementation with long chain polyunsaturated fatty acids (LCPUFA) to breastfeeding mothers for improving child growth and development. *Cochrane Database Syst Rev* 2015; (7): CD007901.

© 2019 Park JJH et al. *JAMA Network Open*.

35. Thiele DK, Senti JL, Anderson CM. Maternal Vitamin D Supplementation to Meet the Needs of the Breastfed Infant. *Journal of Human Lactation* 2013; **29**(2): 163-70.

36. Becker GE, Smith HA, Cooney F. Methods of milk expression for lactating women. *Cochrane Database Syst Rev* 2016; **9**: CD006170.

37. Dangour AD, Watson L, Cumming O, et al. Interventions to improve water quality and supply, sanitation and hygiene practices, and their effects on the nutritional status of children. *Cochrane Database Syst Rev* 2013; (8): CD009382.

38. Darlow BA, Graham PJ, Rojas-Reyes MX. Vitamin A supplementation to prevent mortality and short- and long-term morbidity in very low birth weight infants. *Cochrane Database Syst Rev* 2016; (8): CD000501.

39. Das JK, Salam RA, Kumar R, Bhutta ZA. Micronutrient fortification of food and its impact on woman and child health: a systematic review. *Syst Rev* 2013; **2**: 67.

40. De-Regil LM, Jefferds ME, Sylvetsky AC, Dowswell T. Intermittent iron supplementation for improving nutrition and development in children under 12 years of age. *Cochrane Database Syst Rev* 2011; (12): CD009085.

41. De-Regil LM, Suchdev PS, Vist GE, Walleser S, Peña-Rosas JP. Home fortification of foods with multiple micronutrient powders for health and nutrition in children under two years of age (Review). *Evidence-Based Child Health: A Cochrane Review Journal* 2013; **8**(1): 112-201.

42. Devakumar D, Fall CH, Sachdev HS, et al. Maternal antenatal multiple micronutrient supplementation for long-term health benefits in children: a systematic review and meta-analysis. *BMC Med* 2016; **14**: 90.

43. Gaffey Mea. Dietary management of childhood diarrhea in low- and middle-income countries: a systematic review. *BMC Public Health* 2013; **13**.

44. Gough EK, Moodie EE, Prendergast AJ, et al. The impact of antibiotics on growth in children in low and middle income countries: systematic review and meta-analysis of randomised controlled trials. *BMJ* 2014; **348**: g2267.

© 2019 Park JJH et al. *JAMA Network Open*.

45. Imdad A, Bhutta ZA. Effect of preventive zinc supplementation on linear growth in children under 5 years of age in developing countries: a meta-analysis of studies for input to the lives saved tool. *BMC Public Health* 2011; **11 Suppl 3**: S22.

46. Imdad A, Mayo-Wilson E, Herzer K, Bhutta ZA. Vitamin A supplementation for preventing morbidity and mortality in children from six months to five years of age. *Cochrane Database Syst Rev* 2017; **3**: CD008524.

47. Kristjansson E, Francis DK, Liberato S, et al. Food supplementation for improving the physical and psychosocial health of socio-economically disadvantaged children aged three months to five years. *Cochrane Database Syst Rev* 2015; (3): CD009924.

48. Lassi Zea. Impact of education and provision of complementary feeding on growth and morbidity in children less than 2 years of age in developing countries: a systematic review. *BMC Public Health* 2013; **13**.

49. Matsungo TM, Kruger HS, Smuts CM, Faber M. Lipid-based nutrient supplements and linear growth in children under 2 years: a review. *Proc Nutr Soc* 2017; **76**(4): 580-8.

50. Mayo-Wilson E, Junior JA, Imdad A, et al. Zinc supplementation for preventing mortality, morbidity, and growth failure in children aged 6 months to 12 years of age. *Cochrane Database Syst Rev* 2014; (5): CD009384.

51. Pasricha S-R, Hayes E, Kalumba K, Biggs B-A. Effect of daily iron supplementation on health in children aged 4–23 months: a systematic review and meta-analysis of randomised controlled trials. *The Lancet Global Health* 2013; **1**(2): e77-e86.

52. Petry N, Olofin I, Boy E, Donahue Angel M, Rohner F. The Effect of Low Dose Iron and Zinc Intake on Child Micronutrient Status and Development during the First 1000 Days of Life: A Systematic Review and Meta-Analysis. *Nutrients* 2016; **8**(12).

53. Salam RA. Effectiveness of Micronutrient Powders (MNP) in women and children. *BMC Public Health* 2013; **13**.

54. Sguassero Y, de Onis M, Bonotti AM, Carroli G. Community-based supplementary feeding for promoting the growth of children under five years of age in low and middle income countries. *Cochrane Database Syst Rev* 2012; (6): CD005039.

© 2019 Park JJH et al. *JAMA Network Open*.

55. Taylor-Robinson DC, Maayan N, Soares-Weiser K, Donegan S, Garner P. Deworming drugs for soil-transmitted intestinal worms in children: effects on nutritional indicators, haemoglobin, and school performance. *Cochrane Database Syst Rev* 2015; (7): CD000371.

56. Dias S, Sutton AJ, Ades AE, Welton NJ. Evidence synthesis for decision making 2: a generalized linear modeling framework for pairwise and network meta-analysis of randomized controlled trials. *Medical decision making : an international journal of the Society for Medical Decision Making* 2013; **33**(5): 607-17.

57. Thorlund K, Thabane L, Mills EJ. Modelling heterogeneity variances in multiple treatment comparison meta-analysis – Are informative priors the better solution? *BMC Medical Research Methodology* 2013; **13**: 2-.

58. Turner RM, Davey J, Clarke MJ, Thompson SG, Higgins JP. Predicting the extent of heterogeneity in meta-analysis, using empirical data from the Cochrane Database of Systematic Reviews. *Int J Epidemiol* 2012; **41**(3): 818-27.

59. Rhodes KM, Turner RM, Higgins JPT. Predictive distributions were developed for the extent of heterogeneity in meta-analyses of continuous outcome data. *Journal of Clinical Epidemiology* 2015; **68**(1): 52-60.

60. R: A Language Environment for Statistical Computing. 2017. https://www.R-project.org/.

61. Sturtz S, Ligges U, Gelman A. R2WinBUGS: A Package for Running WinBUGS from R. *Journal of Statistical Software* 2005; **12**(3): 1-16.

62. Dias S, Welton NJ, Sutton AJ, Caldwell DM, Lu G, Ades AE. Evidence Synthesis for Decision Making 4: Inconsistency in Networks of Evidence Based on Randomized Controlled Trials. *Medical Decision Making* 2013; **33**(5): 641-56.

63. Luce BR, Claxton K. Redefining the analytical approach to pharmacoeconomics. *Health Economics* 1999; **8**(3): 187-9.

64. Spiegelhalter DJ, Abrams KR, Myles JP. Bayesian approaches to clinical trials and health-care evaluation. Chichester: John Wiley & Sons; 2004.

65. Sutton AJ, Abrams KR. Bayesian methods in meta-analysis and evidence synthesis. *Statistical methods in medical research* 2001; **10**(4): 277-303.

© 2019 Park JJH et al. *JAMA Network Open*.

66. Goodman S. Toward Evidence-Based Medical Statistics. 1: The P Value Fallacy. *Ann Internal Medicine* 1999; **130**(12): 995.

67. Rhodes KM, Turner RM, White IR, Jackson D, Spiegelhalter DJ, Higgins JP. Implementing informative priors for heterogeneity in meta-analysis using meta-regression and pseudo data. *Statistics in Medicine* 2016; **35**(29): 5495-511.

68. Dempster AP. The direct use of likelihood for significance testing. *Statistics and Computing* 1997; **7**(4): 247-52.

69. Brooks SP, Gelman A. General methods for monitoring convergence of iterative simulations. *Journal of Computational and Graphical Statistics* 1997; **7**(434-455).

70. Adu-Afarwuah S, Lartey A, Okronipa H, et al. Lipid-based nutrient supplement increases the birth size of infants of primiparous women in Ghana. *Am J Clin Nutr* 2015; **101**(4): 835-46.

71. Adu-Afarwuah S, Lartey A, Okronipa H, et al. Small-quantity, lipid-based nutrient supplements provided to women during pregnancy and 6 mo postpartum and to their infants from 6 mo of age increase the mean attained length of 18-mo-old children in semi-urban Ghana: a randomized controlled trial. *Am J Clin Nutr* 2016; **104**(3): 797-808.

72. Prado EL, Adu-Afarwuah S, Lartey A, et al. Effects of pre- and post-natal lipid-based nutrient supplements on infant development in a randomized trial in Ghana. *Early Human Development* 2016; **99**: 43-51.

73. Ahmad SM, Hossain MB, Monirujjaman M, et al. Maternal zinc supplementation improves hepatitis B antibody responses in infants but decreases plasma zinc level. *Eur J Nutr* 2016; **55**(5): 1823-9.

74. Alizadeh L, Salehi L. Is Routine Iron Supplementation Necessary in Pregnant Women With High Hemoglobin? *Iranian Red Crescent Medical Journal* 2016; **18**(1): e22761.

75. Aşcı Ö, Rathfisch G. Effect of lifestyle interventions of pregnant women on their dietary habits, lifestyle behaviors, and weight gain: a randomized controlled trial. *Journal of Health, Population and Nutrition* 2016; **35**(1): 7.

76. Asemi Z. Effect of Calcium-vitamin D Supplementation on Metabolic Profiles in Pregnant Women at Risk for Pre-eclampsia: A Randomized Placebo-controlled trial. *Pakistan Journal of Biological Sciences* 2012; **15**(7): 316-24.

77. Asemi Z, Samimi M, Tabassi Z, Shakeri H, Esmaillzadeh A. Vitamin D supplementation affects serum high-sensitivity C-reactive protein, insulin resistance, and biomarkers of oxidative stress in pregnant women. *J Nutr* 2013; **143**(9): 1432-8.

78. Asemi Z, Esmaillzadeh A. The Effect of Multi mineral-Vitamin D Supplementation on Pregnancy Outcomes in Pregnant Women at Risk for Pre-eclampsia. *International Journal of Preventive Medicine* 2015; **6**: 62.

79. Asemi Z, Samimi M, Siavashani MA, et al. Calcium-Vitamin D Co-supplementation Affects Metabolic Profiles, but not Pregnancy Outcomes, in Healthy Pregnant Women. *International Journal of Preventive Medicine* 2016; **7**: 49.

80. Ashorn P, Alho L, Ashorn U, et al. Supplementation of Maternal Diets during Pregnancy and for 6 Months Postpartum and Infant Diets Thereafter with Small-Quantity Lipid-Based Nutrient Supplements Does Not Promote Child Growth by 18 Months of Age in Rural Malawi: A Randomized Controlled Trial. *The Journal of Nutrition* 2015; **145**(6): 1345-53.

81. Ashorn P, Alho L, Ashorn U, et al. The impact of lipid-based nutrient supplement provision to pregnant women on newborn size in rural Malawi: a randomized controlled trial. *The American Journal of Clinical Nutrition* 2015; **101**(2): 387-97.

82. Stewart CP, Oaks BM, Laugero KD, et al. Maternal cortisol and stress are associated with birth outcomes, but are not affected by lipid-based nutrient supplements during pregnancy: an analysis of data from a randomized controlled trial in rural Malawi. *BMC Pregnancy and Childbirth* 2015; **15**(1): 346.

83. Roth D. Randomized placebo-controlled trial of high-dose prenatal third-trimester vitamin D3 supplementation in Bangladesh: the AViDD trial. *Nutr J* 2013; **12**.

84. Belizan Jea. Calcium Supplemetation to Prevent Hypertensive Disorders of Pregnancy. *New England Journal of Medicine* 1991; **325**(20): 1399-405.

85. Bhutta ZAea. A comparative evaluation of multiple micronutrient and iron–folic acid supplementation during pregnancy in Pakistan: Impact on pregnancy outcomes. *Food and Nutrition Bulletin* 2009; **30**(4): S496-S505.

© 2019 Park JJH et al. *JAMA Network Open*.

86. Callaghan-Gillespie M, Schaffner AA, Garcia P, et al. Trial of ready-to-use supplemental food and corn-soy blend in pregnant Malawian women with moderate malnutrition: a randomized controlled clinical trial. *The American Journal of Clinical Nutrition* 2017; **106**(4): 1062-9.

87. Nair N, Tripathy P, Sachdev H, et al. Effect of participatory women's groups and counselling through home visits on children's linear growth in rural eastern India (CARING trial): a cluster-randomised controlled trial. *The Lancet Global Health* 2017; **5**(10): e1004-e16.

88. Castillo-Durán C, Perales CG, Hertrampf ED, Marín VB, Rivera FA, Icaza G. Effect of zinc supplementation on development and growth of Chilean infants. *The Journal of Pediatrics* 2001; **138**(2): 229-35.

89. Caulfield LEea. Adding zinc to prenatal iron and folate supplements improves
maternal and neonatal zinc status in a Peruvian population1. *The American Journal of Clinical Nutrition* 1999; **69**: 1257-63.

90. Ceesay S. Effects on birth weight and perinatal mortality of maternal dietary supplements in rural Gambia: 5 year randomised controlled trial. *BMJ* 1997; **315**: 786-90.

91. Christian P, Khatry SK, Katz J, et al. Effects of alternative maternal micronutrient supplements on low birth weight in rural Nepal: double blind randomised community trial. *BMJ* 2003; **326**(7389): 571.

92. Kozuki N, Katz J, LeClerq SC, Khatry SK, West KP, Christian P. Risk factors and neonatal/infant mortality risk of small-for-gestational-age and preterm birth in rural Nepal. *The Journal of Maternal-Fetal & Neonatal Medicine* 2014; **28**(9): 1019-25.

93. Cox SE, Staalsoe T, Arthur P, et al. Maternal vitamin A supplementation and immunity to malaria in pregnancy in Ghanaian primigravids. *Trop Med Int Health* 2005; **10**(12): 1286-97.

94. Dandamrongrak Pea. Correlation between Iodine Supplement in Pregnancy
and Neonatal TSH Level. *J Med Assoc Thai* 2016; **99**(12).

95. Danesh A, Janghorbani M, Mohammadi B. Effects of zinc supplementation during pregnancy on pregnancy outcome in women with history of preterm delivery: A double-blind randomized, placebo-controlled trial. *The Journal of Maternal-Fetal & Neonatal Medicine* 2010; **23**(5): 403-8.

© 2019 Park JJH et al. *JAMA Network Open*.

96. Devi S, Mukhopadhyay A, Dwarkanath P, et al. Combined Vitamin B-12 and Balanced Protein-Energy Supplementation Affect Homocysteine Remethylation in the Methionine Cycle in Pregnant South Indian Women of Low Vitamin B-12 Status. *The Journal of Nutrition* 2017; **147**(6): 1094-103.

97. Dijkhuizen Mea. Zinc plus B-carotene supplementation of pregnant women is superior to B-carotene supplementation alone in improving vitamin A status in both mothers and infants. *Am J Clin Nutr* 2004; **80**: 1299-307.

98. Diogenes ME, Bezerra FF, Rezende EP, Taveira MF, Pinhal I, Donangelo CM. Effect of calcium plus vitamin D supplementation during pregnancy in Brazilian adolescent mothers: a randomized, placebo-controlled trial. *Am J Clin Nutr* 2013; **98**(1): 82-91.

99. Diogenes MEL, Bezerra FF, Rezende EP, Donangelo CM. Calcium Plus Vitamin D Supplementation During the Third Trimester of Pregnancy in Adolescents Accustomed to Low Calcium Diets Does Not Affect Infant Bone Mass at Early Lactation in a Randomized Controlled Trial. *The Journal of Nutrition* 2015; **145**(7): 1515-23.

100. Johnson W, Darboe MK, Sosseh F, Nshe P, Prentice AM, Moore SE. Association of prenatal lipid-based nutritional supplementation with fetal growth in rural Gambia. *Maternal & Child Nutrition* 2016; **13**(2): e12367.

101. Jobarteh ML, McArdle HJ, Holtrop G, Sise EA, Prentice AM, Moore SE. mRNA Levels of Placental Iron and Zinc Transporter Genes Are Upregulated in Gambian Women with Low Iron and Zinc Status. *The Journal of Nutrition* 2017; **147**(7): 1401-9.

102. Etheredge AJ, Premji Z, Gunaratna NS, et al. Iron supplementation in iron-replete and nonanemic pregnant women in tanzania: A randomized clinical trial. *JAMA Pediatrics* 2015; **169**(10): 947-55.

103. Fawzi WW. Vitamins and Perinatal Outcomes among HIV-Negative Women in Tanzania. *New England Journal of Medicine* 2007; **356**: 1423-31.

104. Fleming AF, De V. Hendrickse JP, Allan NC. THE PREVENTION OF MEGALOBLASTIC ANAEMIA IN PREGNANCY IN NIGERIA. *BJOG: An International Journal of Obstetrics & Gynaecology* 1968; **75**(4): 425-32.

© 2019 Park JJH et al. *JAMA Network Open*.

105. Friis H. Effect of multimicronutrient supplementation on gestational length and birth size: a randomized, placebo-controlled, double-blind effectiveness trial in Zimbabwe. *Am J Clin Nutr* 2004; **80**: 178-84.

106. Goldberg GR, Jarjou LM, Cole TJ, Prentice A. Randomized, placebo-controlled, calcium supplementation trial in pregnant Gambian women accustomed to a low calcium intake: effects on maternal blood pressure and infant growth. *Am J Clin Nutr* 2013; **98**(4): 972-82.

107. Gonzalez-Casanova I, Stein AD, Hao W, et al. Prenatal Supplementation with Docosahexaenoic Acid Has No Effect on Growth through 60 Months of Age. *The Journal of Nutrition* 2015; **145**(6): 1330-4.

108. Gutierrez-Gomez Y, Stein AD, Ramakrishnan U, et al. Prenatal Docosahexaenoic Acid Supplementation Does Not Affect Nonfasting Serum Lipid and Glucose Concentrations of Offspring at 4 Years of Age in a Follow-Up of a Randomized Controlled Clinical Trial in Mexico. *The Journal of Nutrition* 2017; **147**(2): 242-7.

109. Ramakrishnan U, Gonzalez-Casanova I, Schnaas L, et al. Prenatal supplementation with DHA improves attention at 5 y of age: a randomized controlled trial. *The American Journal of Clinical Nutrition* 2016; **104**(4): 1075-82.

110. Gowachirapant S, Jaiswal N, Melse-Boonstra A, et al. Effect of iodine supplementation in pregnant women on child neurodevelopment: a randomised, double-blind, placebo-controlled trial. *The Lancet Diabetes & Endocrinology* 2017; **5**(11): 853-63.

111. Hafeez A, Mehmood G, Mazhar F. Oral zinc supplementation in pregnant women and its effect on birth weight: a randomised controlled trial. *Arch Dis Child Fetal Neonatal Ed* 2005; **90**(2): F170-1.

112. Hanieh S, Ha TT, Simpson JA, et al. Effect of low-dose versus higher-dose antenatal iron supplementation on child health outcomes at 36 months of age in Viet Nam: longitudinal follow-up of a cluster randomised controlled trial. *BMJ Global Health* 2017; **2**(3).

113. Hashemipour S, Ziaee A, Javadi A, et al. Effect of treatment of vitamin D deficiency and insufficiency during pregnancy on fetal growth indices and maternal weight gain: a randomized clinical trial. *Eur J Obstet Gynecol Reprod Biol* 2014; **172**: 15-9.

© 2019 Park JJH et al. *JAMA Network Open*.

114. Hossain N, Kanani FH, Ramzan S, et al. Obstetric and neonatal outcomes of maternal vitamin D supplementation: results of an open-label, randomized controlled trial of antenatal vitamin D supplementation in Pakistani women. *J Clin Endocrinol Metab* 2014; **99**(7): 2448-55.

115. Huybregts Lieven, Roberfroid Dominique, Lanou Hermann, et al. Prenatal food supplementation fortified with multiple micronutrients increases birth length: a randomized controlled trial in rural Burkina Faso–. *The American journal of clinical nutrition* 2009; **90**(6): 1593-600.

116. Toe LC, Bouckaert KP, De Beuf K, et al. Seasonality Modifies the Effect of a Lipid-Based Nutrient Supplement for Pregnant Rural Women on Birth Length. *The Journal of Nutrition* 2015; **145**(3): 634-9.

117. Iyengar L, Rajalakshmi K. Effect of folic acid supplement on birth weights of infants. *American Journal of Obstetrics & Gynecology* 1975; **122**(3): 332-6.

118. Janmohamed A, Karakochuk CD, Boungnasiri S, et al. Prenatal supplementation with Corn Soya Blend Plus reduces the risk of maternal anemia in late gestation and lowers the rate of preterm birth but does not significantly improve maternal weight gain and birth anthropometric measurements in rural Cambodian women: a randomized trial1. *The American Journal of Clinical Nutrition* 2016; **103**(2): 559-66.

119. West KP, Jr., Shamim AA, Mehra S, et al. Effect of maternal multiple micronutrient vs iron-folic acid supplementation on infant mortality and adverse birth outcomes in rural Bangladesh: the JiVitA-3 randomized trial. *JAMA* 2014; **312**(24): 2649-58.

120. Christian P, Kim J, Mehra S, et al. Effects of prenatal multiple micronutrient supplementation on growth and cognition through 2 y of age in rural Bangladesh: the JiVitA-3 Trial. *The American Journal of Clinical Nutrition* 2016; **104**(4): 1175-82.

121. Kaestel P, Michaelsen KF, Aaby P, Friis H. Effects of prenatal multimicronutrient supplements on birth weight and perinatal mortality: a randomised, controlled trial in Guinea-Bissau. *Eur J Clin Nutr* 2005; **59**(9): 1081-9.

122. Kardjati SRI, Kusin JA, With C. Energy supplementation in the last trimester of pregnancy in East Java: I. Effect on birthweight. *BJOG: An International Journal of Obstetrics & Gynaecology* 1988; **95**(8): 783-94.

123. Katz J. Maternal low-dose vitamin A or !-carotene supplementation has no effect on fetal loss and early infant mortality: a randomized cluster trial in Nepal. *Am J Clin Nutr* 2000; **71**: 1570-6.

© 2019 Park JJH et al. *JAMA Network Open*.

124. Kumar A, Devi SG, Batra S, Singh C, Shukla DK. Calcium supplementation for the prevention of pre-eclampsia. *Int J Gynaecol Obstet* 2009; **104**(1): 32-6.

125. Kumwenda Nea. Antenatal Vitamin A Supplementation Increases Birth Weight and Decreases Anemia among Infants Born to Human Immunodeficiency Virus–Infected Women in Malawi. *HIV/AIDS* 2002; **35**: 618-24.

126. Larocque R, Casapia M, Gotuzzo E, et al. A double-blind randomized controlled trial of antenatal mebendazole to reduce low birthweight in a hookworm-endemic area of Peru. *Trop Med Int Health* 2006; **11**(10): 1485-95.

127. Lopez-Jaramillo Pea. Calcium Supplementation reduces the risk of pregnancy-induced hypertension in an Andes population. *British Journal of Obstetrics and Gynaecology* 1989; **96**: 648-55.

128. Lopez-Jaramillo P, Narvaez M, Felix C, Lopez A. Dietary calcium supplementation and prevention of pregnancy hypertension. *The Lancet* 1990; **335**(8684): 293.

129. Lopez-Jaramillo Pea. Calcium Supplementation and the Risk of Preeclampsia in Ecuadorian Pregnant Teenagers. *Obstetrics and Gynecology* 1997; **90**(2).

130. Liu JM, Mei Z, Ye R, Serdula MK, Ren A, Cogswell ME. Micronutrient supplementation and pregnancy outcomes: double-blind randomized controlled trial in China. *JAMA Intern Med* 2013; **173**(4): 276-82.

131. Wang L, Mei Z, Li H, Zhang Y, Liu J, Serdula MK. Modifying effects of maternal Hb concentration on infant birth weight in women receiving prenatal iron-containing supplements: a randomised controlled trial. *British Journal of Nutrition* 2015; **115**(4): 644-9.

132. Mardones F, Urrutia MT, Villarroel L, et al. Effects of a dairy product fortified with multiple micronutrients and omega-3 fatty acids on birth weight and gestation duration in pregnant Chilean women. *Public Health Nutr* 2008; **11**(1): 30-40.

133. Marya R, Rathee S, Dua V, Sangwan K. Effect of vitamin D supplementation during pregnancy on foetal growth. *The Indian journal of medical research* 1988; **88**: 488.

134. Menendez Cea. The effects of iron supplementation during pregnancy, given by traditional birth attendants, on the prevelence of anaemia and malaria. *Transactions of the Royal Society of Tropical Medicine and Hygiene* 1994; **88**: 590-3.

© 2019 Park JJH et al. *JAMA Network Open*.

135. Merialdi M, Caulfield LE, Zavaleta N, Figueroa A, Dominici F, Dipietro JA. Randomized controlled trial of prenatal zinc supplementation and the development of fetal heart rate. *Am J Obstet Gynecol* 2004; **190**(4): 1106-12.

136. Persson L, Arifeen S, Ekström E, et al. Effects of prenatal micronutrient and early food supplementation on maternal hemoglobin, birth weight, and infant mortality among children in bangladesh: The minimat randomized trial. *JAMA* 2012; **307**(19): 2050-9.

137. Frith AL, Naved RT, Persson LA, Frongillo EA. Early prenatal food supplementation ameliorates the negative association of maternal stress with birth size in a randomised trial. *Maternal & Child Nutrition* 2013; **11**(4): 537-49.

138. Khan AI, Kabir I, Hawkesworth S, et al. Early invitation to food and/or multiple micronutrient supplementation in pregnancy does not affect body composition in offspring at 54 months: follow-up of the MINIMat randomised trial, Bangladesh. *Maternal & Child Nutrition* 2012; **11**(3): 385-97.

139. Kallioinen M, Ekström E-C, Khan AI, et al. Prenatal early food and multiple micronutrient supplementation trial reduced infant mortality in Bangladesh, but did not influence morbidity. *Acta Paediatrica* 2017; **106**(12): 1979-86.

140. Khan AI, Kabir I, Eneroth H, et al. Effect of a randomised exclusive breastfeeding counselling intervention nested into the MINIMat prenatal nutrition trial in Bangladesh. *Acta Paediatrica* 2016; **106**(1): 49-54.

141. Ekström E-C, Lindström E, Raqib R, et al. Effects of prenatal micronutrient and early food supplementation on metabolic status of the offspring at 4.5 years of age. The MINIMat randomized trial in rural Bangladesh. *International Journal of Epidemiology* 2016; **45**(5): 1656-67.

142. Roth DE, Morris SK, Zlotkin S, et al. Vitamin D Supplementation in Pregnancy and Lactation and Infant Growth. *N Engl J Med* 2018; **379**(6): 535-46.

143. Mojibian M, Soheilykhah S, Fallah Zadeh MA, Jannati Moghadam M. The effects of vitamin D supplementation on maternal and neonatal outcome: A randomized clinical trial. *Iranian Journal of Reproductive Medicine* 2015; **13**(11): 687-96.

144. Mwangi MN, Roth JM, Smit MR, et al. Effect of daily antenatal iron supplementation on plasmodium infection in kenyan women: A randomized clinical trial. *JAMA* 2015; **314**(10): 1009-20.

© 2019 Park JJH et al. *JAMA Network Open*.

145. Ndibazza J, Muhangi L, Akishule D, et al. Effects of deworming during pregnancy on maternal and perinatal outcomes in Entebbe, Uganda: a randomized controlled trial. *Clin Infect Dis* 2010; **50**(4): 531-40.

146. Ndyomugyenyi Rea. Efficacy of Ivermectin and Albendazole Alone and in Combination for Treatment of
Soil-Transmitted Helminths in Pregnancy and Adverse Events: A Randomized Open
Label Controlled Intervention Trial in Masindi District, Western Uganda. *Am J Trop Med Hyg* 2008.

147. Kirkwood BRea. Effect of vitamin A supplementation in women of reproductive age on maternal survival in Ghana (ObaapaVitA): a cluster-randomised, placebo-controlled trial. *The Lancet* 2010; **375**(375): 1640-9.

148. Osendarp S. A randomized, placebo-controlled trial of the effect of zinc supplementation during pregnancy on pregnancy outcome in Bangladeshi urban poor. *The American Journal of Clinical Nutrition* 2000; **71**: 114-9.

149. Osrin D, Vaidya A, Shrestha Y, et al. Effects of antenatal multiple micronutrient supplementation on birthweight and gestational duration in Nepal: double-blind, randomised controlled trial. *The Lancet* 2005; **365**(9463): 955-62.

150. Devakumar D, Stocks J, Ayres JG, et al. Effects of antenatal multiple micronutrient supplementation on lung function in mid-childhood: follow-up of a double-blind randomised controlled trial in Nepal. *European Respiratory Journal* 2015.

151. Owens S, Gulati R, Fulford AJ, et al. Periconceptional multiple-micronutrient supplementation and placental function in rural Gambian women: a double-blind, randomized, placebo-controlled trial. *Am J Clin Nutr* 2015; **102**(6): 1450-9.

152. Ramakrishnan U, Nguyen PH, Gonzalez-Casanova I, et al. Neither Preconceptional Weekly Multiple Micronutrient nor Iron–Folic Acid Supplements Affect Birth Size and Gestational Age Compared with a Folic Acid Supplement Alone in Rural Vietnamese Women: A Randomized Controlled Trial–4. *The Journal of nutrition* 2016; **146**(7): 1445S-52S.

153. Hemminki E, Nwaru BI, Salomé G, et al. Is selective prenatal iron prophylaxis better than routine prophylaxis: final results of a trial (PROFEG) in Maputo, Mozambique. *BMJ Open* 2016; **6**(6).

© 2019 Park JJH et al. *JAMA Network Open*.

154. Nwaru BI, Parkkali S, Abacassamo F, et al. A pragmatic randomised controlled trial on routine iron prophylaxis during pregnancy in Maputo, Mozambique (PROFEG): rationale, design, and success. *Maternal & Child Nutrition* 2012; **11**(2): 146-63.

155. Purwar Mea. Calcium Supplementation and Prevention of Pregnancy Induced Hypertension. *J Obstet Gynaecol* 1996; **22**(5): 425-30.

156. Radhika MS, Bhaskaram P, Balakrishna N, Ramalakshmi BA. Red Palm Oil Supplementation: A Feasible Diet-Based Approach to Improve the Vitamin A Status of Pregnant Women and Their Infants. *Food and Nutrition Bulletin* 2003; **24**(2): 208-17.

157. Ramakrishnan Uea. Multiple micronutrient supplementation during pregnancy does not lead to greater infant birth size than does iron-only supplementation: a randomized controlled trial in a semirural community in Mexico. *Am J Clin Nutr* 2003; **77**: 720-5.

158. Mridha MK, Matias SL, Chaparro CM, et al. Lipid-based nutrient supplements for pregnant women reduce newborn stunting in a cluster-randomized controlled effectiveness trial in Bangladesh. *The American Journal of Clinical Nutrition* 2016; **103**(1): 236-49.

159. Mridha MK, Matias SL, Paul RR, et al. Prenatal Lipid-Based Nutrient Supplements Do Not Affect Pregnancy or Childbirth Complications or Cesarean Delivery in Bangladesh: A Cluster-Randomized Controlled Effectiveness Trial. *The Journal of Nutrition* 2017; **147**(9): 1776-84.

160. Matias SL, Mridha MK, Paul RR, et al. Prenatal Lipid-Based Nutrient Supplements Affect Maternal Anthropometric Indicators Only in Certain Subgroups of Rural Bangladeshi Women. *The Journal of Nutrition* 2016; **146**(9): 1775-82.

161. Roberfroid D, Huybregts L, Lanou H, et al. Effects of maternal multiple micronutrient supplementation on fetal growth: a double-blind randomized controlled trial in rural Burkina Faso. *The American Journal of Clinical Nutrition* 2008; **88**(5): 1330-40.

162. Ruangvutilert P. Low-Dose Weekly Intravenous Iron Sucrose versus
Daily Oral Iron for Iron Deficiency Anemia in Late
Pregnancy: A Randomized Controlled Trial. *J Med Assoc Thai* 2017; **100**(5).

163. Saaka Mea. Effect of Prenatal Zinc Supplementation on Birthweight. *J Health Popul Nutr* 2009; **5**(6).

© 2019 Park JJH et al. *JAMA Network Open*.

164. Sablok A, Batra A, Thariani K, et al. Supplementation of vitamin D in pregnancy and its correlation with feto-maternal outcome. *Clinical Endocrinology* 2015; **83**(4): 536-41.

165. Sayyah-Melli M, Ghorbanihaghjo A, Alizadeh M, Kazemi-Shishvan M, Ghojazadeh M, Bidadi S. The Effect of High Dose Folic Acid throughout Pregnancy on Homocysteine (Hcy) Concentration and Pre-Eclampsia: A Randomized Clinical Trial. *PLOS ONE* 2016; **11**(5): e0154400.

166. Effect of maternal multiple micronutrient supplementation on fetal loss and infant death in Indonesia: a double-blind cluster-randomised trial. *The Lancet* 2008; **371**(9608): 215-27.

167. Sunawang. Preventing low birthweight through maternal multiple
micronutrient supplementation: A cluster-randomized,
controlled trial in Indramayu, West Java. *Food and Nutrition Bulletin* 2009; **30**(4).

168. Taherian Aea. Prevention of Preeclampsia with Low-Dose Aspirin or Calcium Supplementaion. 2002.

169. Villar J, Abdel-Aleem H, Merialdi M, et al. World Health Organization randomized trial of calcium supplementation among low calcium intake pregnant women. *American Journal of Obstetrics and Gynecology* 2006; **194**(3): 639-49.

170. Wanchu M, Malhotra S, Khullar M. Calcium supplementation in pre-eclampsia. *J Assoc Physicians India* 2001; **49**: 795-8.

171. Whitfield KC, Karakochuk CD, Kroeun H, et al. Perinatal consumption of thiamine-fortified fish sauce in rural cambodia: A randomized clinical trial. *JAMA Pediatrics* 2016; **170**(10): e162065.

172. Yu CK, Sykes L, Sethi M, Teoh TG, Robinson S. Vitamin D deficiency and supplementation during pregnancy. *Clin Endocrinol (Oxf)* 2009; **70**(5): 685-90.

173. Zagre Nea. Prenatal multiple micronutrient supplementation has greater impact on birthweight than supplementation with iron and folic acid: A cluster-randomized, doubleblind, controlled programmatic study in rural Niger. *Food and Nutrition Bulletin* 2007; **28**(3).

174. Zahiri sorouri Z, Sadeghi H, Pourmarzi D. The effect of zinc supplementation on pregnancy outcome: a randomized controlled trial. *The Journal of Maternal-Fetal & Neonatal Medicine* 2016; **29**(13): 2194-8.

© 2019 Park JJH et al. *JAMA Network Open*.

175. Zeng L, Dibley MJ, Cheng Y, et al. Impact of micronutrient supplementation during pregnancy on birth weight, duration of gestation, and perinatal mortality in rural western China: double blind cluster randomised controlled trial. *BMJ* 2008; **337**: a2001.

176. Zhao G, Xu G, Zhou M, et al. Prenatal Iron Supplementation Reduces Maternal Anemia, Iron Deficiency, and Iron Deficiency Anemia in a Randomized Clinical Trial in Rural China, but Iron Deficiency Remains Widespread in Mothers and Neonates. *The Journal of Nutrition* 2015; **145**(8): 1916-23.

177. Ziaei S, Mehrnia M, Faghihzadeh S. Iron status markers in nonanemic pregnant women with and without iron supplementation. *Int J Gynaecol Obstet* 2008; **100**(2): 130-2.

178. Acharya N, Singh RR, Bhatta NK, Poudel P. Randomized Control Trial of Kangaroo Mother Care in Low Birth Weight Babies at a Tertiary Level Hospital. *Journal of Nepal Paediatric Society* 2014; **34**(1).

179. Adu-Afarwuah S, Lartey A, Okronipa H, et al. Maternal Supplementation with Small-Quantity Lipid-Based Nutrient Supplements Compared with Multiple Micronutrients, but Not with Iron and Folic Acid, Reduces the Prevalence of Low Gestational Weight Gain in Semi-Urban Ghana: A Randomized Controlled Trial–3. *The Journal of nutrition* 2017; **147**(4): 697-705.

180. Ashorn P, Alho L, Ashorn U, et al. Supplementation of Maternal Diets during Pregnancy and for 6 Months Postpartum and Infant Diets Thereafter with Small-Quantity Lipid-Based Nutrient Supplements Does Not Promote Child Growth by 18 Months of Age in Rural Malawi: A Randomized Controlled Trial. *J Nutr* 2015; **145**(6): 1345-53.

181. Ashorn P, Alho L, Ashorn U, et al. The impact of lipid-based nutrient supplement provision to pregnant women on newborn size in rural Malawi: a randomized controlled trial. *Am J Clin Nutr* 2015; **101**(2): 387-97.

182. Boo NY, Jamli FM. Short duration of skin-to-skin contact: effects on growth and breastfeeding. *J Paediatr Child Health* 2007; **43**(12): 831-6.

183. Feliciano Eea. Seasonal and Geographical Variations in the Growth Rate of Infants in China Receiving Increasing Dosages of Vitamin D Supplements. *Journal of Tropical Pediatrics* 1994; **40**: 162-5.

184. Gathwala G, Singh B, Singh J. Effect of Kangaroo Mother Care on physical growth, breastfeeding and its acceptability. *Trop Doct* 2010; **40**(4): 199-202.

© 2019 Park JJH et al. *JAMA Network Open*.

185. Tomlinson M, Doherty T, Jackson D, et al. An effectiveness study of an integrated, community-based package for maternal, newborn, child and HIV care in South Africa: study protocol for a randomized controlled trial. *Trials* 2011; **12**: 236.

186. Tomlinson M, Doherty T, Ijumba P, et al. Goodstart: a cluster randomised effectiveness trial of an integrated, community-based package for maternal and newborn care, with prevention of mother-to-child transmission of HIV in a South African township. *Trop Med Int Health* 2014; **19**(3): 256-66.

187. Habib MA, Soofi S, Sheraz A, et al. Zinc supplementation fails to increase the immunogenicity of oral poliovirus vaccine: a randomized controlled trial. *Vaccine* 2015; **33**(6): 819-25.

188. Hamadani Jea. Randomized controlled trial of the effect of zinc supplementation on the mental development of Bangladeshi infants. *Am J Clin Nutr* 2001; **74**.

189. Christian P, Kim J, Mehra S, et al. Effects of prenatal multiple micronutrient supplementation on growth and cognition through 2 y of age in rural Bangladesh: the JiVitA-3 Trial. *Am J Clin Nutr* 2016; **104**(4): 1175-82.

190. West KP, Shamim AA, Mehra S, et al. Effect of Maternal Multiple Micronutrient vs Iron–Folic Acid Supplementation on Infant Mortality and Adverse Birth Outcomes in Rural Bangladesh. *Jama* 2014; **312**(24): 2649.

191. Kumbhojkar Sea. Kangaroo Mother Care (KMC): An Alternative to Conventional Method of Care for Low Birth Weight Babies. *International Journal of Health Sciences and Research* 2016; **6**(3): 36-42.

192. le Roux IM, Tomlinson M, Harwood JM, et al. Outcomes of home visits for pregnant mothers and their infants: a cluster randomised controlled trial. *AIDS (London, England)* 2013; **27**(9): 1461.

193. Rotheram-Borus MJ, Tomlinson M, le Roux IM, et al. A cluster randomised controlled effectiveness trial evaluating perinatal home visiting among South African mothers/infants. *PLoS One* 2014; **9**(10): e105934.

194. Locks LM, Manji KP, McDonald CM, et al. Effect of zinc and multivitamin supplementation on the growth of Tanzanian children aged 6-84 wk: a randomized, placebo-controlled, double-blind trial. *Am J Clin Nutr* 2016; **103**(3): 910-8.

195. Locks L, Manji K, McDonald C, et al. Effect of Zinc & Multiple Micronutrient Supplements on Growth in Tanzanian Children. *The FASEB Journal* 2015; **29**(1_supplement): 729.1.

© 2019 Park JJH et al. *JAMA Network Open*.

196. Lonnerdal B, Kvistgaard AS, Peerson JM, Donovan SM, Peng YM. Growth, Nutrition, and Cytokine Response of Breast-fed Infants and Infants Fed Formula With Added Bovine Osteopontin. *J Pediatr Gastroenterol Nutr* 2016; **62**(4): 650-7.

197. Nikiema L, Huybregts L, Martin-Prevel Y, et al. Effectiveness of facility-based personalized maternal nutrition counseling in improving child growth and morbidity up to 18 months: A cluster-randomized controlled trial in rural Burkina Faso. *PLoS One* 2017; **12**(5): e0177839.

198. Osendarp S. Effect of zinc supplementation between 1 and 6 mo of life on growth and morbidity of Bangladeshi infants in urban slums. *Am J Clin Nutr* 2002; **76**: 1401-8.

199. Ostadrahimi A, Salehi-Pourmehr H, Mohammad-Alizadeh-Charandabi S, Heidarabady S, Farshbaf-Khalili A. The effect of perinatal fish oil supplementation on neurodevelopment and growth of infants: a randomized controlled trial. *Eur J Nutr* 2017.

200. Engebretsen IM. Growth effects of exclusive breastfeeding promotion by peer counsellors in sub-Saharan Africa: the cluster-randomised PROMISE EBF trial. *BMC Public Health* 2014; **14**.

201. Fadnes LT, Nankabirwa V, Engebretsen IM, et al. Effects of an exclusive breastfeeding intervention for six months on growth patterns of 4–5 year old children in Uganda: the cluster-randomised PROMISE EBF trial. *BMC Public Health* 2016; **16**(1).

202. Tylleskar T, Jackson D, Meda N, et al. Exclusive breastfeeding promotion by peer counsellors in sub-Saharan Africa (PROMISE-EBF): a cluster-randomised trial. *Lancet* 2011; **378**(9789): 420-7.

203. Dewey KG, Mridha MK, Matias SL, et al. Lipid-based nutrient supplementation in the first 1000 d improves child growth in Bangladesh: a cluster-randomized effectiveness trial. *Am J Clin Nutr* 2017; **105**(4): 944-57.

204. Mridha MK, Matias SL, Chaparro CM, et al. Lipid-based nutrient supplements for pregnant women reduce newborn stunting in a cluster-randomized controlled effectiveness trial in Bangladesh. *Am J Clin Nutr* 2016; **103**(1): 236-49.

205. Shafique S, Sellen DW, Lou W, Jalal CS, Jolly SP, Zlotkin SH. Mineral- and vitamin-enhanced micronutrient powder reduces stunting in full-term low-birth-weight infants receiving nutrition, health, and hygiene education: a 2 x 2 factorial, cluster-randomized trial in Bangladesh. *Am J Clin Nutr* 2016; **103**(5): 1357-69.

© 2019 Park JJH et al. *JAMA Network Open*.

206. Simondon KB, Gartner A, Berger J, et al. Effect of early, short-term supplementation on weight and linear growth of 4-7-mo-old infants in developing countries: a four-country randomized trial. *Am J Clin Nutr* 1996; **64**(4): 537-45.

207. Suman RP, Udani R, Nanavati R. Kangaroo mother care for low birth weight infants: a randomized controlled trial. *Indian Pediatr* 2008; **45**(1): 17-23.

208. Urban M. Growth of infants born to HIV-infected women when fed a biologically acidified starter formula with and without probiotics. *A Afr J Clin Nutr* 2008; **21**(1): 28-32.

209. Vazir S, Engle P, Balakrishna N, et al. Cluster-randomized trial on complementary and responsive feeding education to caregivers found improved dietary intake, growth and development among rural Indian toddlers. *Matern Child Nutr* 2013; **9**(1): 99-117.

210. Velaphi SC, Cooper PA, Bolton KD, et al. Growth and metabolism of infants born to women infected with human immunodeficiency virus and fed acidified whey-adapted starter formulas. *Nutrition* 2008; **24**(3): 203-11.

211. Adu-Afarwuah S. Randomized comparison of 3 types of micronutrient supplements for home fortification of complementary foods in Ghana: effects on growth and motor development. *Am J Clin Nutr* 2007; **86**: 412-20.

212. Black Mea. Cognitive and Motor Development Among Small-for-Gestational- Age Infants: Impact of Zinc Supplementation, Birth Weight, and Caregiving Practices. *Pediatrics* 2004; **113**(5).

213. Fischer Walker CL, Baqui AH, Ahmed S, et al. Low-dose weekly supplementation of iron and/or zinc does not affect growth among Bangladeshi infants. *Eur J Clin Nutr* 2009; **63**(1): 87-92.

214. Brown KH. Comparison of the effects of zinc delivered in a fortified food or a liquid supplement on the growth, morbidity, and plasma zinc concentrations of young Peruvian children. *Am J Clin Nutr* 2007; **85**: 538-47.

215. Castillo-Durán C, Rodríguez A, Venegas G, Alvarez P, Icaza G. Zinc supplementation and growth of infants born small for gestational age. *The Journal of Pediatrics* 1995; **127**(2): 206-11.

216. The Chilenje Infant G, Nutrition, Infection Study T. Micronutrient Fortification to Improve Growth and Health of Maternally HIV-Unexposed and Exposed Zambian Infants: A Randomised Controlled Trial. *PLOS ONE* 2010; **5**(6): e11165.

© 2019 Park JJH et al. *JAMA Network Open*.

217. Filteau S, Baisley K, Chisenga M, Kasonka L, Gibson RS, Team tCS. Provision of Micronutrient-Fortified Food From 6 Months of Age Does Not Permit HIV-Exposed Uninfected Zambian Children to Catch Up in Growth to HIV-Unexposed Children: A Randomized Controlled Trial. *JAIDS Journal of Acquired Immune Deficiency Syndromes* 2011; **56**(2): 166-75.

218. Cohen Rea. Determinants of Growth From Birth to 12 Months Among Breast-Fed Honduran Infants in Relation to Age of Introduction of Complementary Foods. *Pediatrics* 1995; **96**: 504-10.

219. Dewey KG, Domellöf M, Cohen RJ, Landa Rivera L, Hernell O, Lönnerdal B. Iron Supplementation Affects Growth and Morbidity of Breast-Fed Infants: Results of a Randomized Trial in Sweden and Honduras. *The Journal of Nutrition* 2002; **132**(11): 3249-55.

220. Dijkhuizen Mea. Effects of Iron and Zinc Supplementation in Indonesian Infants on Micronutrient Status and Growth. *J Nutr* 2001; **131**: 2960-865.

221. Doherty CP, Sarkar MA, Shakur MS, Ling SC, Elton RA, Cutting WA. Zinc and rehabilitation from severe protein-energy malnutrition: higher-dose regimens are associated with increased mortality. *The American Journal of Clinical Nutrition* 1998; **68**(3): 742-8.

222. Duggan C, Penny ME, Hibberd P, et al. Oligofructose-supplemented infant cereal: 2 randomized, blinded, community-based trials in Peruvian infants. *The American Journal of Clinical Nutrition* 2003; **77**(4): 937-42.

223. Fahmida U. Zinc-iron, but not zinc-alone supplementation, increased linear growth of stunted infants with low haemoglobin. *Asia Pac J Clin Nutr* 2007; **12**(2): 301-9.

224. Fink G, Levenson R, Tembo S, Rockers PC. Home- and community-based growth monitoring to reduce early life growth faltering: an open-label, cluster-randomized controlled trial. *The American Journal of Clinical Nutrition* 2017; **106**(4): 1070-7.

225. Giovannini Mea. Double-Blind, Placebo-Controlled Trial Comparing Effects of Supplementation with Two Different Combinations of Micronutrients Delivered as Sprinkles on Growth, Anemia, and Iron Deficiency in Cambodian Infants. *Journal of Pedriatric Gastroentology and Nutrition* 2006; **42**: 306-12.

226. Goto R, Mascie-Taylor CG, Lunn PG. Impact of anti-Giardia and anthelminthic treatment on infant growth and intestinal permeability in rural Bangladesh: a randomised double-blind controlled study. *Trans R Soc Trop Med Hyg* 2009; **103**(5): 520-9.

227. Helmizar Hea. Local food supplementation and psychosocial stimulation improve linear growth and cognitive development among Indonesian infants aged 6 to 9 months. *Asia Pac J Clin Nutr* 2017; **26**(1).

228. Hess SY, Abbeddou S, Jimenez EY, et al. Small-quantity lipid-based nutrient supplements, regardless of their zinc content, increase growth and reduce the prevalence of stunting and wasting in young burkinabe children: a cluster-randomized trial. *PLoS One* 2015; **10**(3): e0122242.

229. Iannotti LL, Dulience SJ, Green J, et al. Linear growth increased in young children in an urban slum of Haiti: a randomized controlled trial of a lipid-based nutrient supplement. *Am J Clin Nutr* 2014; **99**(1): 198-208.

230. Idjradinata P. Reversal of developmental delays in iron-deficient anaemic infants treated with iron. *The Lancet* 1993; **341**(8836).

231. Idjradinata P, Watkins WE, Pollitt E. Adverse effect of iron supplementation on weight gain of iron-replete young children. *The Lancet* 1994; **343**(8908): 1252-4.

232. Jack SJ, Ou K, Chea M, et al. Effect of micronutrient sprinkles on reducing anemia: a cluster-randomized effectiveness trial. *Arch Pediatr Adolesc Med* 2012; **166**(9): 842-50.

233. Jones KDJ, Ali R, Khasira MA, et al. Ready-to-use therapeutic food with elevated n-3 polyunsaturated fatty acid content, with or without fish oil, to treat severe acute malnutrition: a randomized controlled trial. *BMC Medicine* 2015; **13**(1): 93.

234. Joseph SA, Casapía M, Montresor A, et al. The Effect of Deworming on Growth in One-Year-Old Children Living in a Soil-Transmitted Helminth-Endemic Area of Peru: A Randomized Controlled Trial. *PLOS Neglected Tropical Diseases* 2015; **9**(10): e0004020.

235. Joseph SA, Casapía M, Lazarte F, et al. The effect of deworming on early childhood development in Peru: A randomized controlled trial. *SSM - Population Health* 2015; **1**: 32-9.

© 2019 Park JJH et al. *JAMA Network Open*.

236. Krebs NF, Mazariegos M, Chomba E, et al. Randomized controlled trial of meat compared with multimicronutrient-fortified cereal in infants and toddlers with high stunting rates in diverse settings. *The American Journal of Clinical Nutrition* 2012; **96**(4): 840-7.

237. Kupka R, Manji KP, Bosch RJ, et al. Multivitamin Supplements Have No Effect on Growth of Tanzanian Children Born to HIV-Infected Mothers. *The Journal of Nutrition* 2013; **143**(5): 722-7.

238. Kuusipalo Hea. Growth and Change in Blood Haemoglobin Concentration Among Underweight Malawian Infants Receiving Fortified Spreads for 12 Weeks: A Preliminary Trial. *Journal of Pedriatric Gastroentology and Nutrition* 2006; **43**: 525-32.

239. Lartey A. A randomized, community-based trial of the effects of improved, centrally processed complementary foods on growth and micronutrient status of Ghanaian infants from 6 to 12 mo of age. *Am J Clin Nutr* 1999; **70**: 391-404.

240. Mangani C, Maleta K, Phuka J, et al. Effect of complementary feeding with lipid-based nutrient supplements and corn-soy blend on the incidence of stunting and linear growth among 6- to 18-month-old infants and children in rural Malawi. *Matern Child Nutr* 2015; **11 Suppl 4**: 132-43.

241. Mangani C, Cheung YB, Maleta K, et al. Providing lipid-based nutrient supplements does not affect developmental milestones among Malawian children. *Acta Paediatrica* 2013; **103**(1): e17-e26.

242. Lin CA, Manary MJ, Maleta K, Briend A, Ashorn P. An Energy-Dense Complementary Food Is Associated with a Modest Increase in Weight Gain When Compared with a Fortified Porridge in Malawian Children Aged 6–18 Months. *The Journal of Nutrition* 2008; **138**(3): 593-8.

243. Lind Tea. A community-based randomized controlled trial of iron and zinc supplementation in Indonesian infants: effects on growth and development. *Am J Clin Nutr* 2004; **80**: 729-36.

244. Locks LM, Manji KP, McDonald CM, et al. Effect of zinc and multivitamin supplementation on the growth of Tanzanian children aged 6–84 wk: a randomized, placebo-controlled, double-blind trial. *The American Journal of Clinical Nutrition* 2016; **103**(3): 910-8.

245. Maleta KM, Phuka J, Alho L, et al. Provision of 10-40 g/d Lipid-Based Nutrient Supplements from 6 to 18 Months of Age Does Not Prevent Linear Growth Faltering in Malawi. *J Nutr* 2015; **145**(8): 1909-15.

© 2019 Park JJH et al. *JAMA Network Open*.

246. Mamiro PS, Kolsteren PW, van Camp JH, Roberfroid DA, Tatala S, Opsomer AS. Processed complementary food does not improve growth or hemoglobin status of rural Tanzanian infants from 6–12 months of age in Kilosa district, Tanzania. *The Journal of nutrition* 2004; **134**(5): 1084-90.

247. Mazariegos M, Hambidge KM, Westcott JE, et al. Neither a zinc supplement nor phytate-reduced maize nor their combination enhance growth of 6- to 12-month-old Guatemalan infants. *J Nutr* 2010; **140**(5): 1041-8.

248. Mda S, van Raaij JMA, de Villiers FPR, Kok FJ. Impact of Multi-Micronutrient Supplementation on Growth and Morbidity of HIV-Infected South African Children. *Nutrients* 2013; **5**(10): 4079-92.

249. Medeiros DA, Hadler MCCM, Sugai A, Torres VM. The effect of folic acid supplementation with ferrous sulfate on the linear and ponderal growth of children aged 6–24 months: a randomized controlled trial. *European Journal Of Clinical Nutrition* 2014; **69**: 198.

250. Muhoozi GK, Atukunda P, Diep LM, et al. Nutrition, hygiene, and stimulation education to improve growth, cognitive, language, and motor development among infants in Uganda: A cluster-randomized trial. *Maternal & child nutrition* 2018; **14**(2): e12527.

251. Newton S, Owusu-Agyei S, Asante KP, et al. Vitamin A status and body pool size of infants before and after consuming fortified home-based complementary foods. *Archives of Public Health* 2016; **74**: 10.

252. Oelofse A, Van Raaij JM, Benade AJ, Dhansay MA, Tolboom JJ, Hautvast JG. The effect of a micronutrient-fortified complementary food on micronutrient status, growth and development of 6- to 12-month-old disadvantaged urban South African infants. *Int J Food Sci Nutr* 2003; **54**(5): 399-407.

253. Olney DK. Combined Iron and Folic Acid Supplementation with or without Zinc Reduces Time to Walking
Unassisted among Zanzibari Infants 5- to 11-mo old. *The Journal of Nutrition* 2006: 2427-34.

254. Osei AK, Pandey P, Spiro D, et al. Adding multiple micronutrient powders to a homestead food production programme yields marginally significant benefit on anaemia reduction among young children in N epal. *Maternal & child nutrition* 2015; **11**: 188-202.

255. Phu PV, Hoan NV, Salvignol B, et al. A Six-Month Intervention with Two Different Types of Micronutrient-Fortified Complementary Foods Had Distinct Short- and Long-Term Effects on Linear and Ponderal Growth of Vietnamese Infants. *The Journal of Nutrition* 2012; **142**(9): 1735-40.

256. Phuka J, Thakwalakwa C, Maleta K, et al. Supplementary feeding with fortified spread among moderately underweight 6–18-month-old rural Malawian children. *Maternal & Child Nutrition* 2009; **5**(2): 159-70.

257. Phuka JC, Gladstone M, Maleta K, et al. Developmental outcomes among 18-month-old Malawians after a year of complementary feeding with lipid-based nutrient supplements or corn-soy flour. *Maternal & child nutrition* 2012; **8**(2): 239-48.

258. Phuka JC, Maleta K, Thakwalakwa C, et al. Postintervention growth of Malawian children who received 12-mo dietary complementation with a lipid-based nutrient supplement or maize-soy flour. *Am J Clin Nutr* 2009; **89**(1): 382-90.

259. Ramakrishnan U, Neufeld LM, Flores R, Rivera J, Martorell R. Multiple micronutrient supplementation during early childhood increases child size at 2 y of age only among high compliers. *Am J Clin Nutr* 2009; **89**(4): 1125-31.

260. Dewey KG, Mridha MK, Matias SL, et al. Lipid-based nutrient supplementation in the first 1000 d improves child growth in Bangladesh: a cluster-randomized effectiveness trial–3. *The American journal of clinical nutrition* 2017; **105**(4): 944-57.

261. Matias SL, Vargas-Vásquez A, Bado Pérez R, et al. Effects of lipid-based nutrient supplements v. micronutrient powders on nutritional and developmental outcomes among Peruvian infants. *Public Health Nutrition* 2017; **20**(16): 2998-3007.

262. Rivera J. Zinc Supplementation Improves the Growth of Stunted Rural Guatemalan Infants. *The Journal of Nutrition* 1998; **128**: 556-62.

263. Rivera Jea. Multiple micronutrient supplementation increases the growth of Mexican infants. *Am J Clin Nutr* 2001; **74**: 657-63.

264. Saleem AF, Mahmud S, Baig-Ansari N, Zaidi AK. Impact of maternal education about complementary feeding on their infants' nutritional outcomes in low-and middle-income households: a

© 2019 Park JJH et al. *JAMA Network Open*.

community-based randomized interventional study in Karachi, Pakistan. *Journal of health, population, and nutrition* 2014; **32**(4): 623.

265. Dijkhuizen Mea. Zinc Supplementation Improved Length Growth Only in Anemic Infants in a Multi-Country Trial
of Iron and Zinc Supplementation in South-East Asia. *The Journal of Nutrition* 2008; **138**.

266. Berger J, Ninh NX, Khan NC, et al. Efficacy of combined iron and zinc supplementation on micronutrient status and growth in Vietnamese infants. *Eur J Clin Nutr* 2006; **60**(4): 443-54.

267. Walravens P. Zinc Supplementation in Infants With a Nutritional Pattern of Failure to Thrive: A Double Blind, Controlled Study. *Pediatrics* 1989; **83**(4).

268. Wieringa FT, Dijkhuizen MA, West CE, Thurnham DI, Muhilal, Van der Meer JWM. Redistribution of vitamin A after iron supplementation in Indonesian infants. *The American Journal of Clinical Nutrition* 2003; **77**(3): 651-7.

269. Shafique S. Prevention of linear growth faltering among low birth weight infants in rural bangladesh: A community-based cluster randomized trial. 2013.

270. Singla DR, Shafique S, Zlotkin SH, Aboud FE. A 22-Element Micronutrient Powder Benefits Language but Not Cognition in Bangladeshi Full-Term Low-Birth-Weight Children. *The Journal of Nutrition* 2014; **144**(11): 1803-10.

271. Skau JK, Touch B, Chhoun C, et al. Effects of animal source food and micronutrient fortification in complementary food products on body composition, iron status, and linear growth: a randomized trial in Cambodia. *Am J Clin Nutr* 2015; **101**(4): 742-51.

272. International Research on Infant Supplementation Study G. Efficacy of a Foodlet-Based Multiple Micronutrient Supplement for Preventing Growth Faltering, Anemia, and Micronutrient Deficiency of Infants: The Four Country IRIS Trial Pooled Data Analysis. *The Journal of Nutrition* 2005; **135**(3): 631S-8S.

273. Smuts CM, Dhansay MA, Faber M, et al. Efficacy of multiple micronutrient supplementation for improving anemia, micronutrient status, and growth in South African infants. *J Nutr* 2005; **135**(3): 653S-9S.

© 2019 Park JJH et al. *JAMA Network Open*.

274. Taneja S, Strand TA, Sommerfelt H, Bahl R, Bhandari N. Zinc Supplementation for Four Months Does Not Affect Growth in Young North Indian Children. *The Journal of Nutrition* 2010; **140**(3): 630-4.

275. Tang M, Sheng X-Y, Krebs NF, Hambidge KM. Meat as Complementary Food for Older Breastfed Infants and Toddlers: A Randomized, Controlled Trial in Rural China. *Food and Nutrition Bulletin* 2014; **35**(4_suppl3): S188-S92.

276. Thakwalakwa CM, Ashorn P, Jawati M, Phuka JC, Cheung YB, Maleta KM. An effectiveness trial showed lipid-based nutrient supplementation but not corn–soya blend offered a modest benefit in weight gain among 6- to 18-month-old underweight children in rural Malawi. *Public Health Nutrition* 2012; **15**(9): 1755-62.

277. Thakwalakwa C, Ashorn P, Phuka J, et al. A lipid-based nutrient supplement but not corn-soy blend modestly increases weight gain among 6- to 18-month-old moderately underweight children in rural Malawi. *J Nutr* 2010; **140**(11): 2008-13.

278. Christian Pea. Effect of fortified complementary food supplementation on child growth in rural Bangladesh: a cluster-randomized trial. *International Journal of Epidemiology* 2015: 1-15.

279. Thu Bea. Effect of daily and weekly micronutrient supplementation on micronutrient deficiencies and growth in young Vietnamese children. *Am J Clin Nutr* 1999; **69**: 80-6.

280. Umeta M, West CE, Haidar J, Deurenberg P, Hautvast JGAJ. Zinc supplementation and stunted infants in Ethiopia: a randomised controlled trial. *The Lancet* 2000; **355**(9220): 2021-6.

281. Untoro J. Multiple Micronutrient Supplements Improve Micronutrient Status and Anemia But Not Growth and Morbidity of Indonesian Infants: A Randomized, Double-Blind, Placebo-Controlled Trial. *J Nutr* 2005; **135**.

282. Wasanatwisut Eea. Iron and Zinc Supplementation Improved Iron and Zinc Status, but Not Physical Growth, of Apparently Healthy, Breast-Fed Infants in Rural Communities of Northeast Thailand. *J Nutr* 2006; **136**: 2405-11.

283. Luby SP, Rahman M, Arnold BF, et al. Effects of water quality, sanitation, handwashing, and nutritional interventions on diarrhoea and child growth in rural Bangladesh: a cluster randomised controlled trial. *Lancet Glob Health* 2018; **6**(3): e302-e15.

284. Null C, Stewart CP, Pickering AJ, et al. Effects of water quality, sanitation, handwashing, and nutritional interventions on diarrhoea and child growth in rural Kenya: a cluster-randomised controlled trial. *Lancet Glob Health* 2018; **6**(3): e316-e29.

285. Williams EA, Elia M, Lunn PG. A double-blind, placebo-controlled, glutamine-supplementation trial in growth-faltering Gambian infants. *The American Journal of Clinical Nutrition* 2007; **86**(2): 421-7.

286. Zlotkin S, Arthur P, Schauer C, Antwi KY, Yeung G, Piekarz A. Home-Fortification with Iron and Zinc Sprinkles or Iron Sprinkles Alone Successfully Treats Anemia in Infants and Young Children. *The Journal of Nutrition* 2003; **133**(4): 1075-80.

© 2019 Park JJH et al. *JAMA Network Open*.

Literature search and flow diagram

eFigure 1. An overview of the literature review strategy

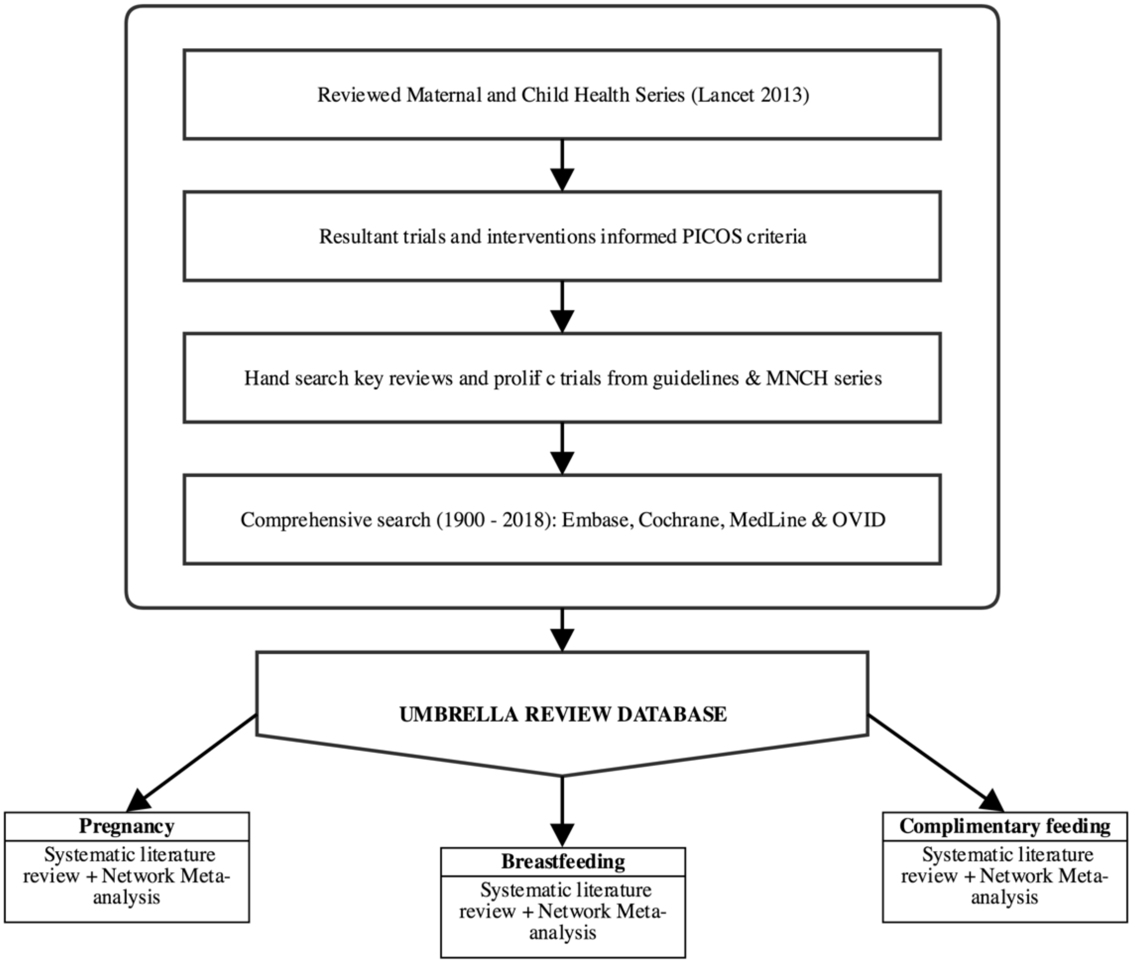

© 2019 Park JJH et al. *JAMA Network Open*.

eFigure 2. Study flow diagram

Identification

22449 records identified through database searching

290 additional records identified through other sources

Screening

22738 records screened

21666 records excluded

Eligibility

1,072 full-text articles assessed for eligibility

Included

215 papers, reporting 189 trials included

Pregnancy
109 papers, reporting
87 trials included

Exclusive Breastfeeding
38 papers, reporting
27 trials included

Complementary Feeding
82 papers, reporting
68 trials included

© 2019 Park JJH et al. *JAMA Network Open*.

Forest plots and model diagnostics of primary analysis, cluster and non-cluster trials

eFigure 3A. Preterm birth forest plot (odds ratio) for pregnancy, primary analysis (cluster and non-cluster trials)

vs. Standard of care	OR (95% interval)
Folic Acid	0.61 (0.30, 1.13)
Calcium	* 0.76 (0.56, 0.98)
Calcium+VitD	1.37 (0.75, 2.52)
Iron	* 0.55 (0.31, 0.90)
VitA	0.56 (0.25, 1.06)
VitD	0.69 (0.38, 1.19)
Zinc	* 0.53 (0.28, 0.93)
Folic Acid+VitA	0.55 (0.25, 1.13)
Iron+Zinc	0.56 (0.28, 1.05)
IFA	0.59 (0.29, 1.07)
IFA+VitA	0.54 (0.24, 1.12)
IFA+Zinc	0.86 (0.37, 1.95)
IFA+Zinc+VitA	0.55 (0.25, 1.15)
MMN	* 0.54 (0.27, 0.97)
Deworming 1dose	0.85 (0.21, 3.23)
Deworming 1dose+Iron	0.43 (0.15, 1.07)
Deworming 2dose	0.97 (0.17, 4.16)
Iron+Calcium+VitD	0.20 (0.03, 1.41)
Iron+Calcium	* 0.16 (0.03, 0.87)
Flour 750kcal fort	0.25 (0.03, 1.66)
Milk 261–575kcal	1.09 (0.12, 9.93)
Milk 261–575kcal+VitB12	0.27 (0.03, 3.56)
LNS 118kcal	0.58 (0.27, 1.14)
LNS 118kcal fort	0.56 (0.25, 1.16)
LNS 372kcal fort	0.64 (0.29, 1.32)
LNS 746kcal	0.24 (0.04, 1.09)
LNS 746kcal fort	0.50 (0.12, 1.72)
Iodine	0.72 (0.33, 1.56)
Iodine+Iron	0.48 (0.15, 1.45)

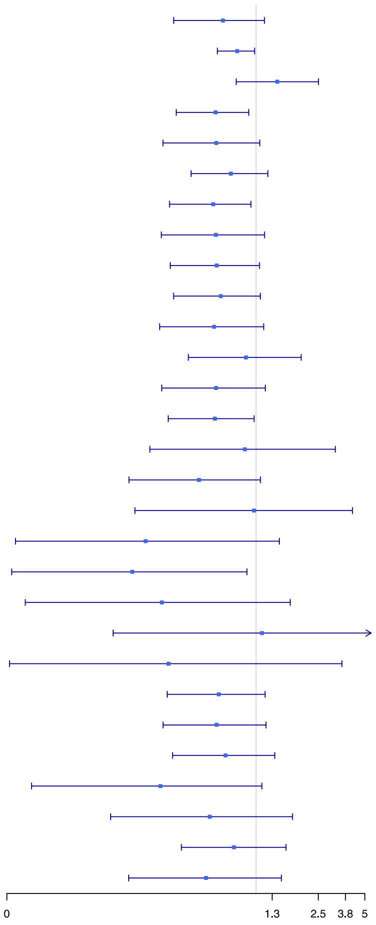

© 2019 Park JJH et al. *JAMA Network Open*.

eFigure 3B. Birthweight forest plot (mean difference) for pregnancy, primary analysis (cluster and non-cluster trials)

vs. Standard of care	Mean Diff. (95% interval)
Folic Acid	0.05 (−0.03, 0.14)
Calcium	0.03 (−0.01, 0.07)
Calcium+VitD	* 0.11 (0.04, 0.18)
Iron	* 0.09 (0.03, 0.15)
VitA	0.01 (−0.09, 0.10)
VitB1	−0.02 (−0.28, 0.23)
VitD	−0.01 (−0.06, 0.05)
Zinc	* 0.12 (0.06, 0.18)
Folic Acid+VitA	0.01 (−0.10, 0.13)
Iron+Zinc	0.08 (0.00, 0.17)
IFA	0.05 (−0.04, 0.13)
IFA+VitA	* 0.11 (0.01, 0.21)
IFA+Zinc	0.03 (−0.08, 0.13)
IFA+Zinc+VitA	0.00 (−0.11, 0.11)
MMN	* 0.08 (0.00, 0.17)
MMN+VitD	−0.08 (−0.24, 0.06)
Deworming 1dose	0.02 (−0.02, 0.07)
Deworming 1dose+Iron	* 0.10 (0.00, 0.20)
Deworming 2dose	−0.02 (−0.12, 0.07)
Iron+Calcium+VitD	0.09 (−0.11, 0.29)
Iron+Calcium	* 0.15 (0.02, 0.28)
Flour 750kcal fort	0.05 (−0.07, 0.18)
Milk 261−575kcal fort	0.03 (−0.09, 0.14)
Milk 261−575kcal	−0.04 (−0.12, 0.04)
Milk 261−575kcal+VitB12	* −0.19 (−0.36, −0.02)
Local food 597kcal−850kcal fort	0.08 (−0.03, 0.18)
LNS20	* 0.11 (0.02, 0.21)
LNS20 fort	0.09 (−0.02, 0.20)
LNS72 fort	0.10 (−0.02, 0.21)
Maternal Education	0.04 (−0.12, 0.20)
Iodine+Iron	0.05 (−0.08, 0.19)
DHA	0.05 (−0.03, 0.13)
Home Visits+Peer Counseling	−0.06 (−0.15, 0.03)

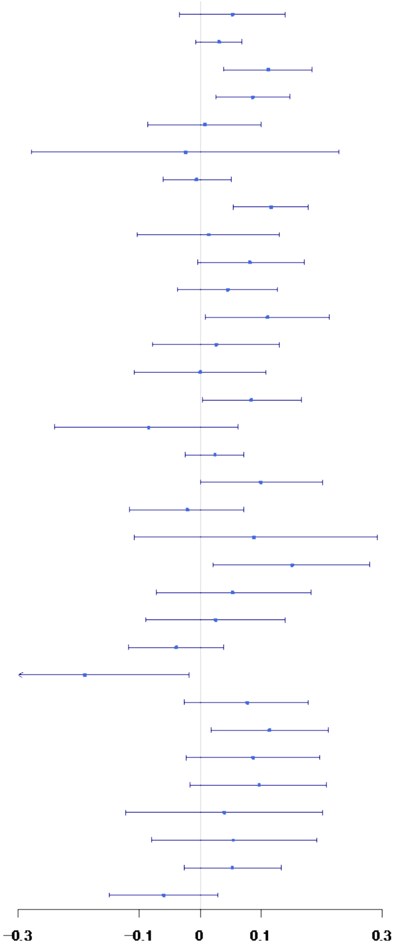

© 2019 Park JJH et al. *JAMA Network Open*.

eFigure 3C. Leverage plot of preterm birth NMA (pregnancy), primary analysis (cluster and non-cluster trials)

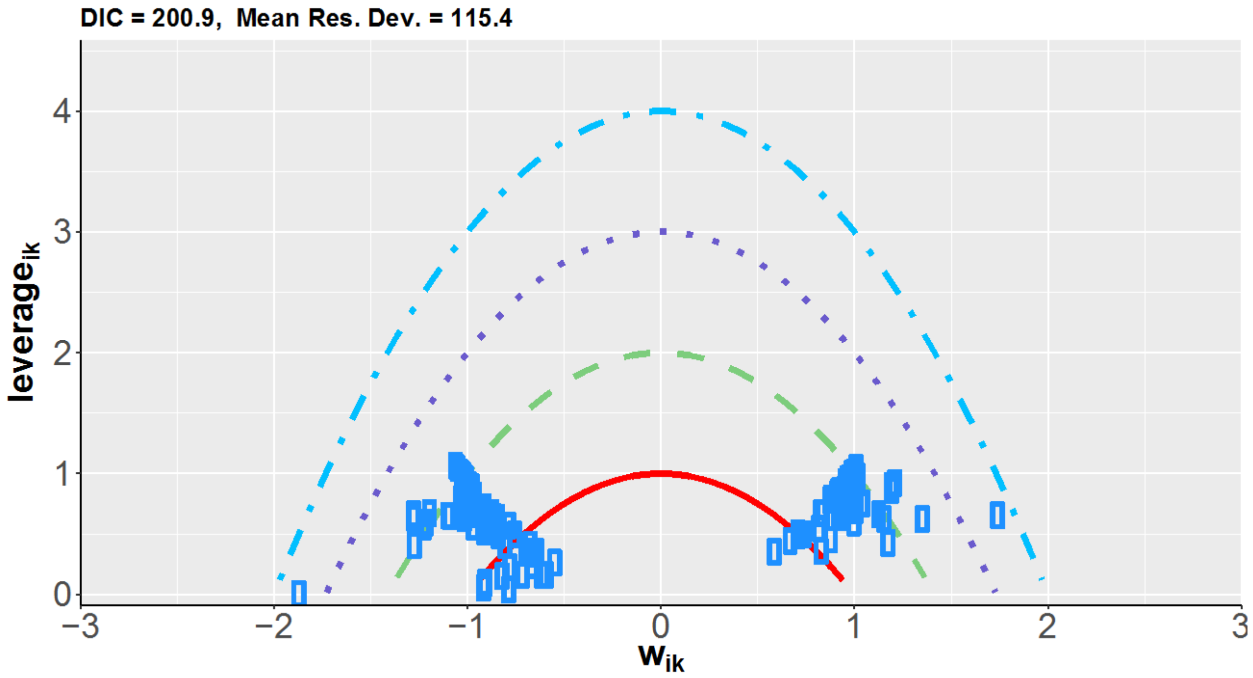

Each point represents a study's arm and its contribution to the DIC is noted in the x-axis (W Ik). Points that are further away from the origin contribute more to the DIC value and are therefore more susceptible to being outliers.

© 2019 Park JJH et al. *JAMA Network Open*.

eFigure 3D. Consistency check of preterm birth NMA (pregnancy), primary analysis (cluster and non-cluster trials)

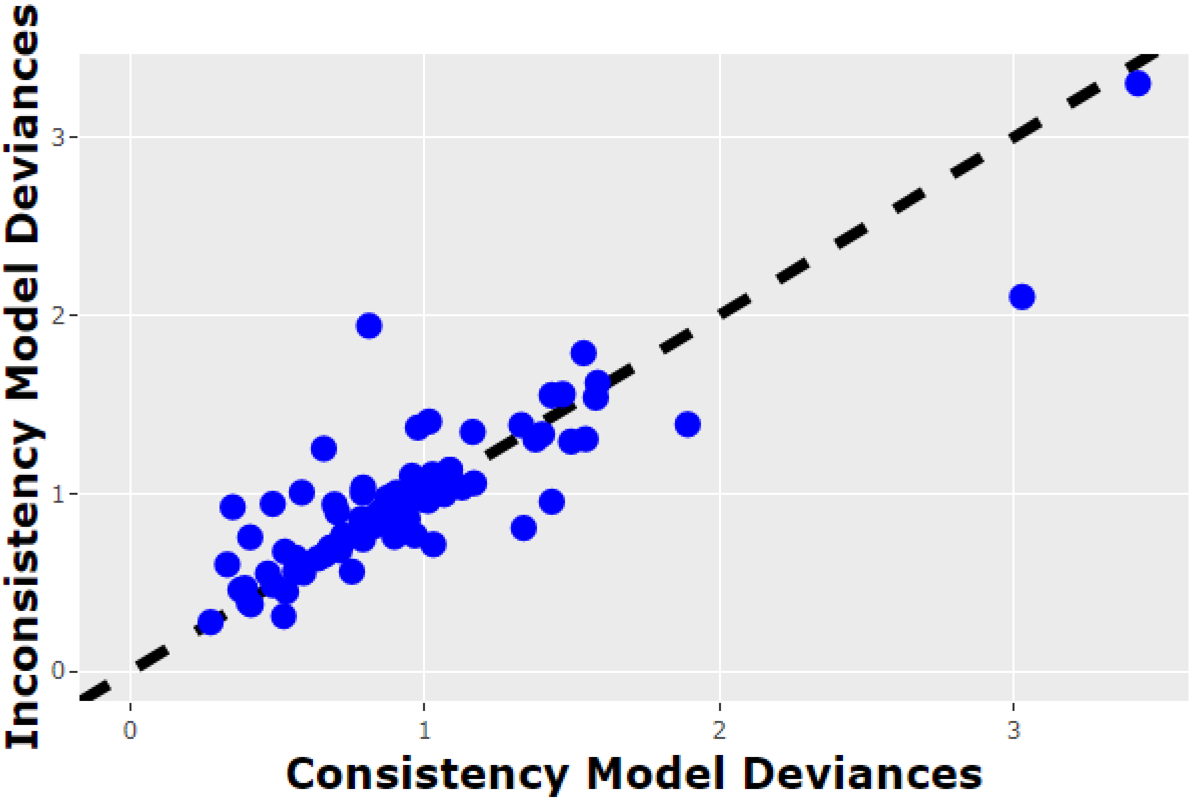

This figure illustrates the residual deviances of the inconsistency model (the NMA model fitted with no consistency constraints) versus those of standard NMA models under the consistency constraints. Study arms represented by points well below the y=x line may point to potential inconsistencies; study arms represented by points above the y=x line favor the consistency model.

© 2019 Park JJH et al. *JAMA Network Open*.

eFigure 3E. Leverage plot of mean birthweight NMA (pregnancy), primary analysis (cluster and non-cluster trials)

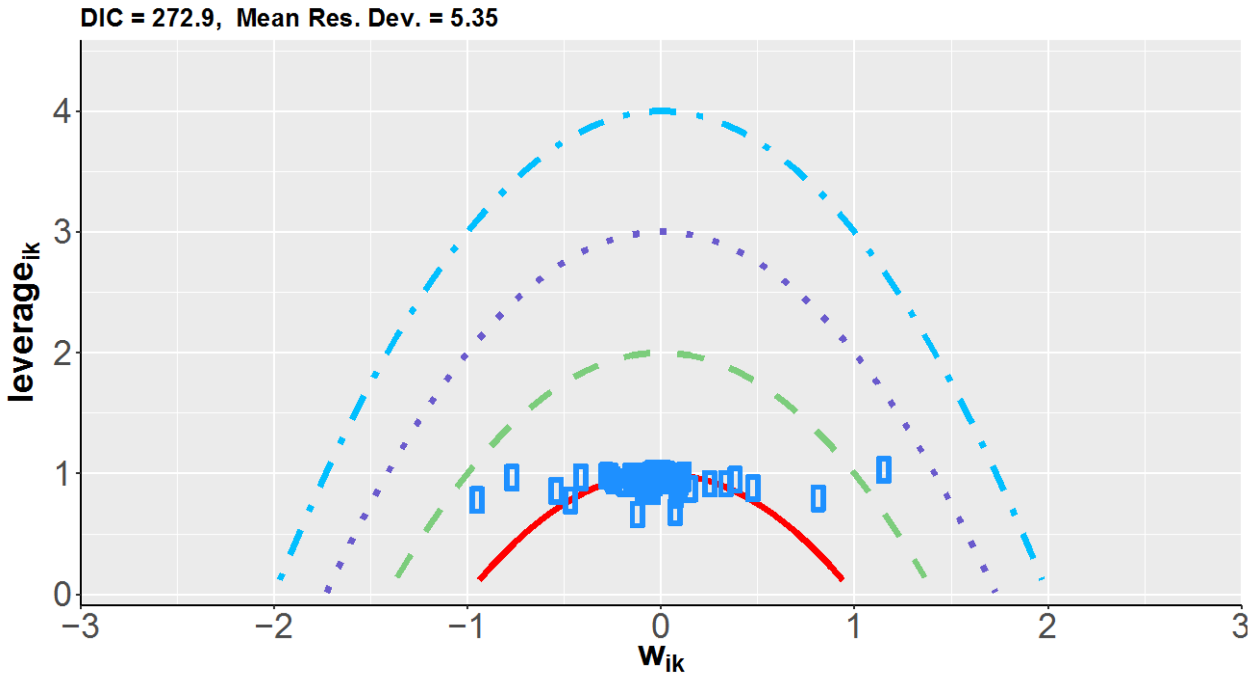

Each point represents a study's arm and its contribution to the DIC is noted in the x-axis (W Ik). Points that are further away from the origin contribute more to the DIC value and are therefore more susceptible to being outliers.

© 2019 Park JJH et al. *JAMA Network Open*.

eFigure 3F. Consistency check of mean birthweight NMA (pregnancy), primary analysis (cluster and non-cluster trials)

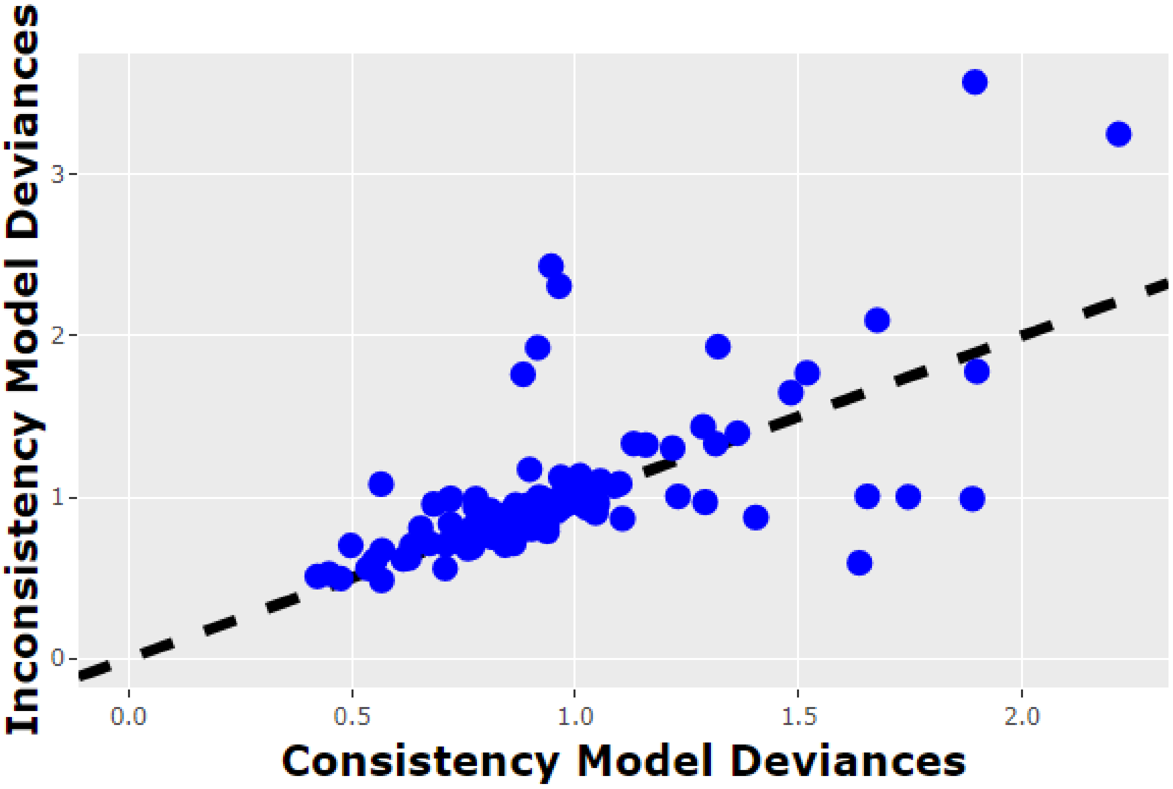

	Consistency	Inconsistency
Deviance	134.3	141.2
pD	111.4	114.9
DIC	245.8	256.1

This figure illustrates the residual deviances of the inconsistency model (the NMA model fitted with no consistency constraints) versus those of standard NMA models under the consistency constraints. Study arms represented by points well below the y=x line may point to potential inconsistencies; study arms represented by points above the y=x line favor the consistency model.

© 2019 Park JJH et al. *JAMA Network Open*.

eFigure 3G. LAZ forest plot (mean difference) for exclusive breastfeeding period, primary analysis (cluster and non-cluster trials)

vs. Standard of Care	Mean Diff. (95% interval)
MMN M	−0.02 (−0.18, 0.14)
MMN C	* 0.20 (0.03, 0.35)
LNS 118kcal (20g) M	−0.01 (−0.17, 0.16)
IFA M	0.05 (−0.15, 0.23)
IFA+LNS 118kcal (20g) M	0.08 (−0.13, 0.29)
Zinc 5mg C	0.12 (−0.03, 0.24)
Formula 335kcal C	0.05 (−0.20, 0.30)
Formula+OPN 65 C	−0.13 (−0.37, 0.11)
Formula+OPN 130 C	−0.06 (−0.30, 0.17)
Maternal education M	0.05 (−0.12, 0.23)
Peer counseling M	−0.05 (−0.18, 0.07)
Individual counseling M	0.06 (−0.14, 0.26)
VitD M	0.08 (−0.10, 0.27)

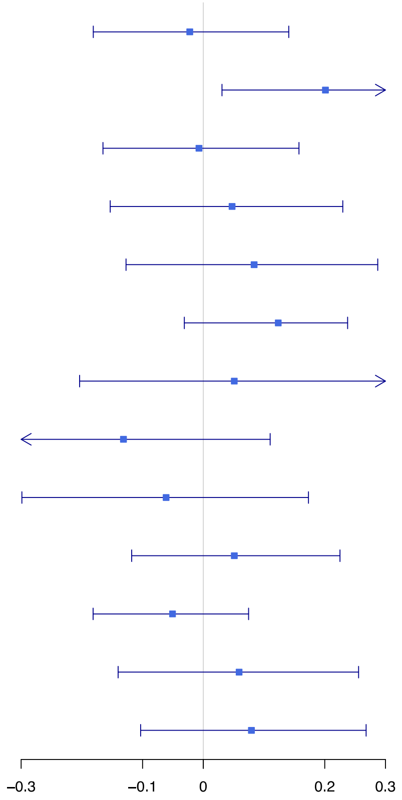

© 2019 Park JJH et al. *JAMA Network Open*.

eFigure 3H. Leverage plot of LAZ NMA (exclusive breastfeeding period), primary analysis (cluster and non-cluster trials)

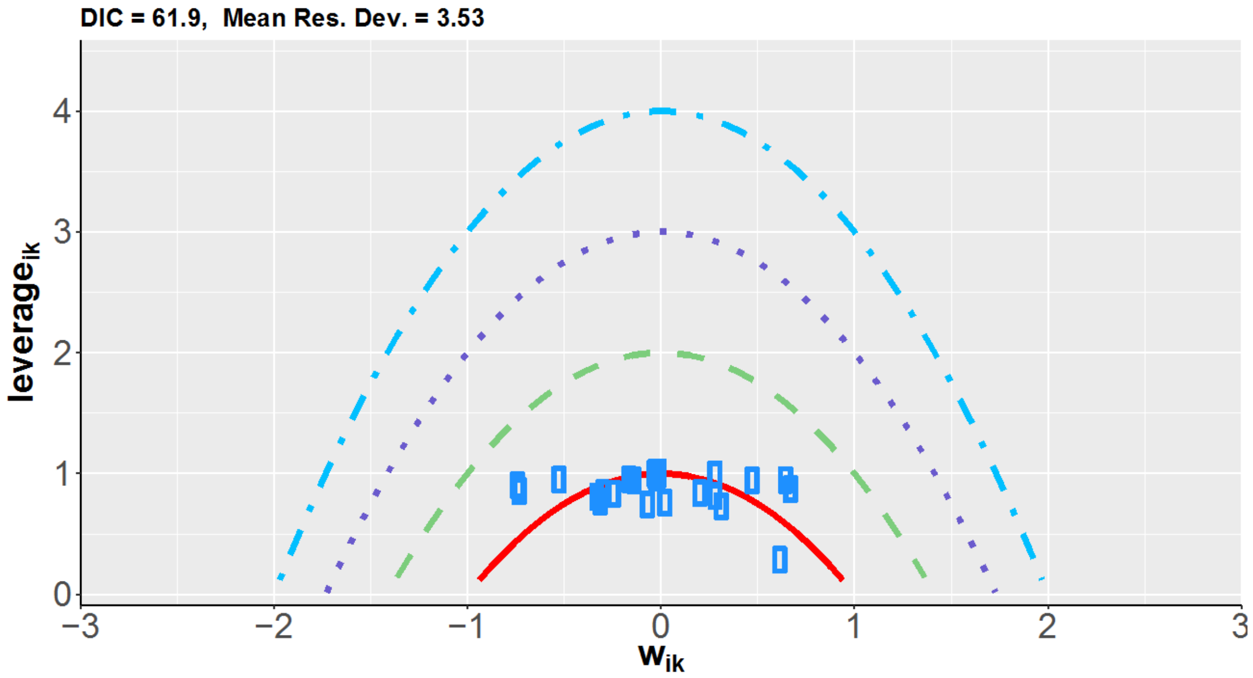

Each point represents a study's arm and its contribution to the DIC is noted in the x-axis (W Ik). Points that are further away from the origin contribute more to the DIC value and are therefore more susceptible to being outliers.

© 2019 Park JJH et al. *JAMA Network Open*.

eFigure 3I. Consistency check of LAZ NMA (exclusive breastfeeding period), primary analysis (cluster and non-cluster trials)

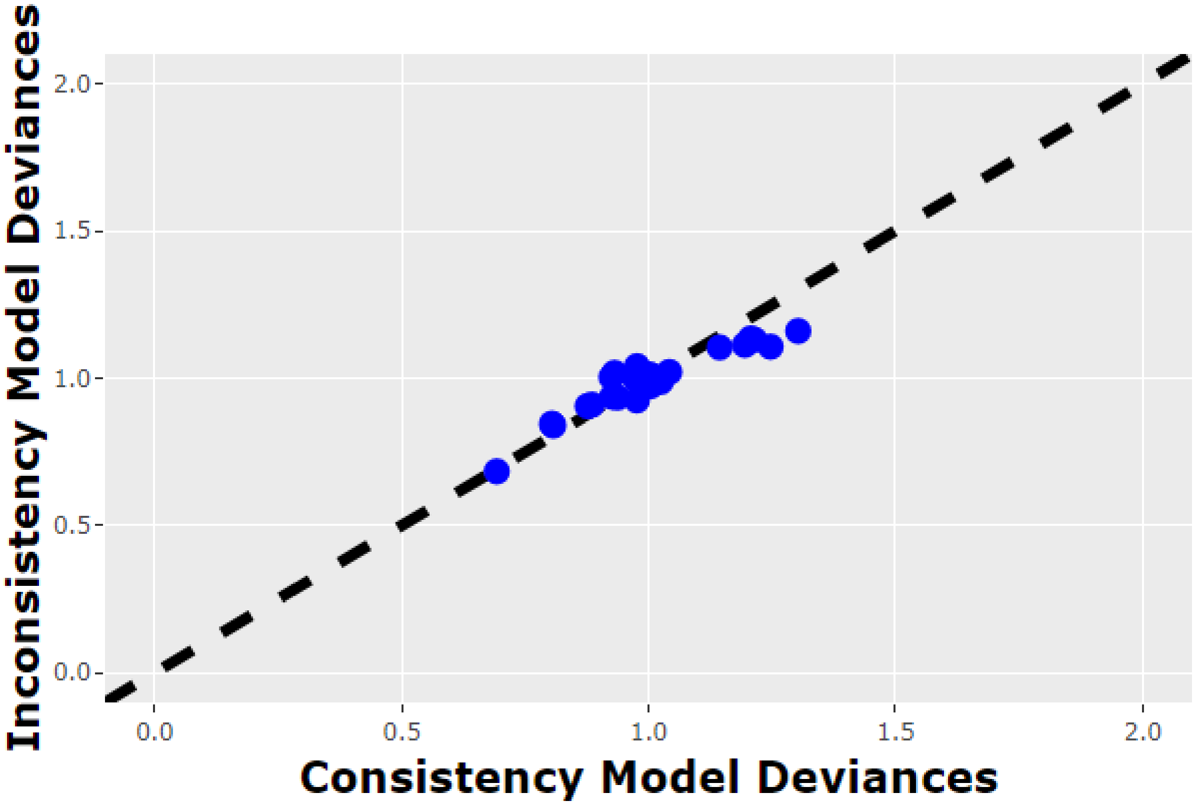

	Consistency	Inconsistency
Deviance	32.1	31.8
pD	29.9	30.5
DIC	61.9	62.2

This figure illustrates the residual deviances of the inconsistency model (the NMA model fitted with no consistency constraints) versus those of standard NMA models under the consistency constraints. Study arms represented by points well below the y=x line may point to potential inconsistencies; study arms represented by points above the y=x line favor the consistency model.

© 2019 Park JJH et al. *JAMA Network Open*.

eFigure 3J. HAZ forest plot (mean difference) for complementary feeding period, primary analysis (cluster and non-cluster trials)

vs. Standard of care	Mean Diff. (95% interval)
IFA	* 0.18 (0.05, 0.30)
IFA+Zinc	0.11 (−0.07, 0.29)
Iron	0.05 (−0.04, 0.15)
Iron+VitA	0.11 (−0.06, 0.26)
Iron+VitB2	0.32 (−0.17, 0.81)
Iron+Zinc	−0.04 (−0.13, 0.05)
Iron+Zinc+VitA	−0.01 (−0.24, 0.23)
Iron+Zinc+VitB2	0.13 (−0.38, 0.65)
Zinc	−0.02 (−0.08, 0.04)
Zinc+VitB2	−0.07 (−0.58, 0.42)
Zinc+VitBcomplex+C+E	0.05 (−0.09, 0.16)
VitB2	0.33 (−0.16, 0.81)
VitBcomplex+C+E	−0.04 (−0.17, 0.07)
MMN weekly	0.09 (−0.19, 0.37)
MMN	* 0.14 (0.02, 0.25)
Maternal education	−0.10 (−0.26, 0.03)
Antibiotics+Deworming 1dose	−0.04 (−0.28, 0.21)
Deworming 1dose	−0.01 (−0.13, 0.10)
Deworming 2doses	0.01 (−0.12, 0.13)
Porridge 185−340kcal	−0.02 (−0.18, 0.14)
Porridge 185−340kcal fort	0.05 (−0.09, 0.19)
Flour 270−340kcal	0.05 (−0.08, 0.19)
Flour 270−340kcal fort	0.19 (−0.33, 0.69)
LNS 23−55kcal fort	0.05 (−0.09, 0.19)
LNS 118−130kcal fort	−0.03 (−0.11, 0.04)
LNS 118kcal	−0.04 (−0.19, 0.11)
LNS 220−285kcal fort	−0.01 (−0.09, 0.07)
Local food 185−260kcal	0.04 (−0.10, 0.18)
Water treatment	−0.09 (−0.22, 0.03)
Toilet facilities	−0.07 (−0.20, 0.05)
Handwashing	−0.05 (−0.18, 0.07)
WASH combined	−0.06 (−0.19, 0.06)
WASH combined+LNS 118kcal fort	0.02 (−0.10, 0.14)

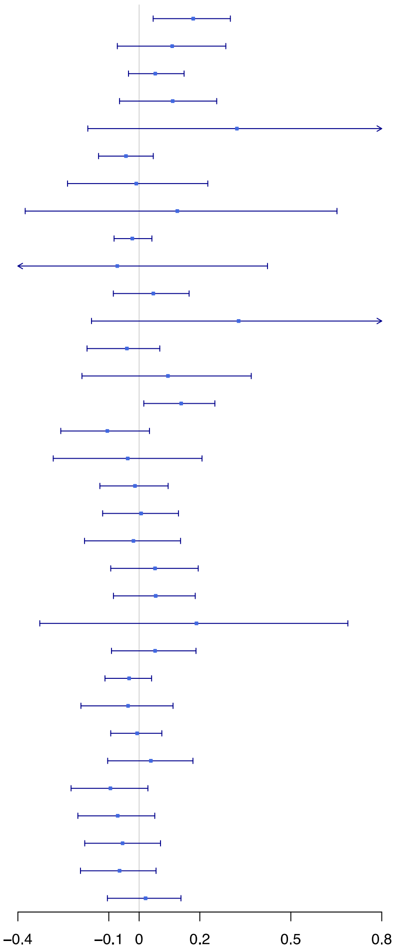

© 2019 Park JJH et al. *JAMA Network Open*.

eFigure 3K. Leverage plot of HAZ NMA (complementary feeding period), primary analysis (cluster and non-cluster trials)

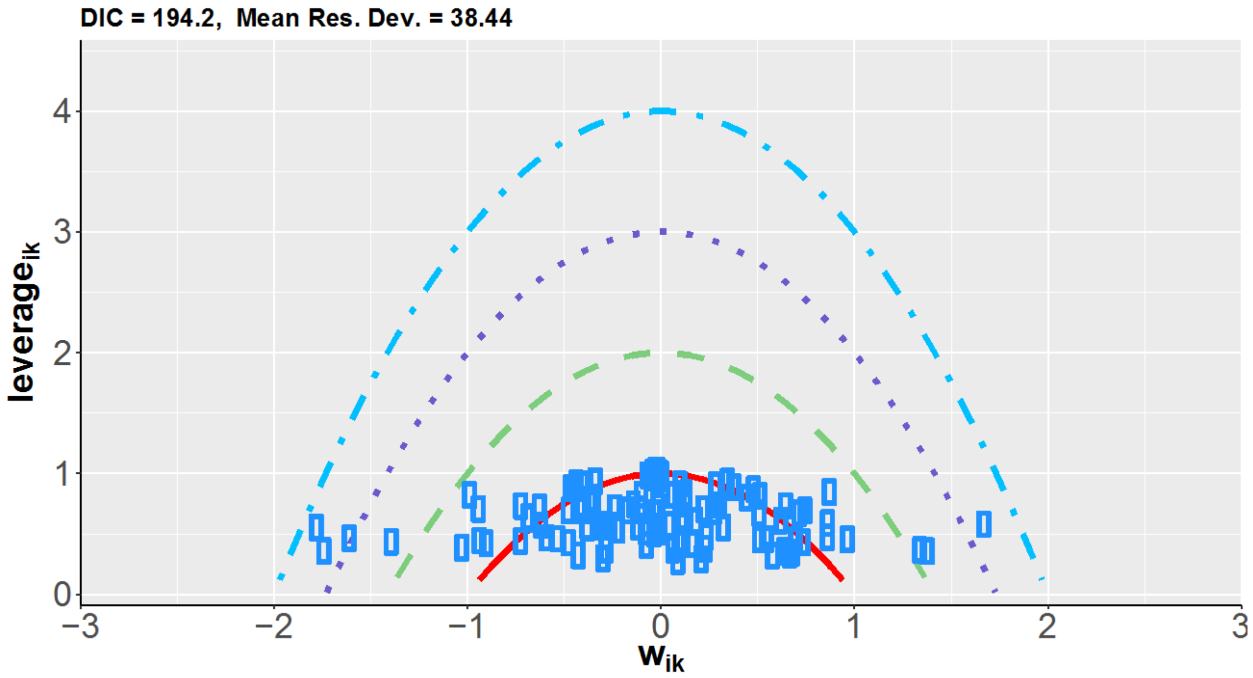

Each point represents a study's arm and its contribution to the DIC is noted in the x-axis (W Ik). Points that are further away from the origin contribute more to the DIC value and are therefore more susceptible to being outliers.

© 2019 Park JJH et al. *JAMA Network Open*.

eFigure 3L. Consistency check of HAZ NMA (complementary feeding period), primary analysis (cluster and non-cluster trials)

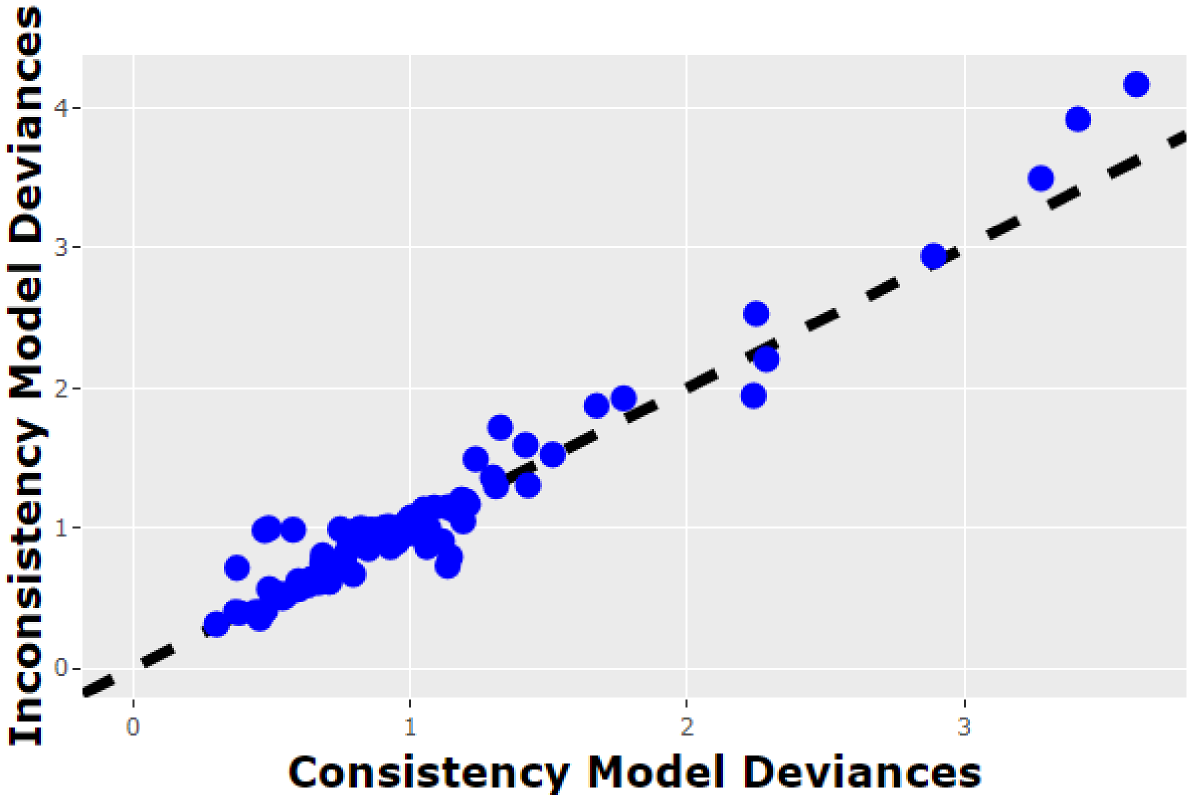

	Consistency	Inconsistency
Deviance	115.7	119.7
pD	77.8	80.4
DIC	193.6	200.1

This figure illustrates the residual deviances of the inconsistency model (the NMA model fitted with no consistency constraints) versus those of standard NMA models under the consistency constraints. Study arms represented by points well below the y=x line may point to potential inconsistencies; study arms represented by points above the y=x line favor the consistency model.

© 2019 Park JJH et al. *JAMA Network Open*.

Forest plots of sensitivity analysis I, non-cluster trials only

eFigure 4A. Preterm birth forest plot (odds ratio) for pregnancy, sensitivity analysis (non-cluster trials only)

vs. Standard of care	OR (95% interval)
Folic Acid	0.59 (0.27, 1.16)
Calcium	0.76 (0.53, 1.01)
Calcium+VitD	1.34 (0.72, 2.39)
Iron	0.60 (0.30, 1.01)
VitA	0.55 (0.15, 1.70)
VitD	0.72 (0.38, 1.21)
Zinc	0.60 (0.28, 1.07)
Iron+Zinc	0.60 (0.28, 1.18)
IFA	0.58 (0.28, 1.11)
IFA+VitA	0.20 (0.02, 1.53)
IFA+Zinc	0.81 (0.32, 1.93)
IFA+Zinc+VitA	0.24 (0.03, 1.89)
MMN	* 0.52 (0.25, 0.99)
Deworming 1dose	0.67 (0.16, 2.69)
Deworming 1dose+Iron	0.47 (0.16, 1.26)
Deworming 2dose	0.76 (0.14, 3.41)
Iron+Calcium+VitD	0.20 (0.03, 1.50)
Iron+Calcium	* 0.16 (0.03, 0.91)
Milk 261−575kcal	1.07 (0.13, 7.86)
Milk 261−575kcal+VitB12	0.27 (0.03, 3.00)
LNS 118kcal	0.56 (0.25, 1.18)
LNS 372kcal fort	0.61 (0.26, 1.35)
LNS 746kcal	0.24 (0.03, 1.05)
LNS 746kcal fort	0.49 (0.11, 1.75)
Iodine	0.73 (0.34, 1.56)
Iodine+Iron	0.52 (0.16, 1.67)

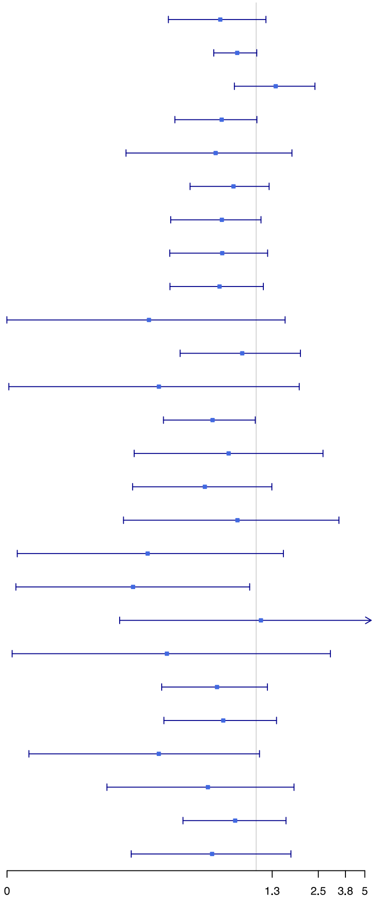

© 2019 Park JJH et al. *JAMA Network Open*.

eFigure 4B. Birthweight forest plot (mean difference) for pregnancy, sensitivity analysis (non-cluster trials only)

vs. Standard of care	Mean Diff. (95% interval)
Folic Acid	0.07 (−0.04, 0.18)
Calcium	0.03 (−0.01, 0.07)
Calcium+VitD	* 0.11 (0.03, 0.19)
Iron	* 0.09 (0.02, 0.16)
VitA	−0.01 (−0.14, 0.12)
VitB1	−0.02 (−0.27, 0.23)
VitD	−0.01 (−0.07, 0.05)
Zinc	* 0.11 (0.05, 0.18)
Iron+Zinc	0.09 (−0.01, 0.19)
IFA	0.06 (−0.05, 0.16)
IFA+VitA	* 0.16 (0.02, 0.31)
IFA+Zinc	0.03 (−0.09, 0.16)
IFA+Zinc+VitA	−0.11 (−0.30, 0.10)
MMN	0.09 (−0.01, 0.20)
MMN+VitD	−0.08 (−0.24, 0.09)
Deworming 1dose	0.02 (−0.03, 0.07)
Deworming 1dose+Iron	0.10 (0.00, 0.22)
Deworming 2dose	−0.02 (−0.12, 0.07)
Iron+Calcium+VitD	0.10 (−0.11, 0.32)
Iron+Calcium	* 0.16 (0.02, 0.31)
Milk 261−575kcal fort	0.02 (−0.09, 0.15)
Milk 261−575kcal	−0.04 (−0.12, 0.04)
Milk 261−575kcal+VitB12	* −0.19 (−0.37, −0.02)
Local food 597kcal–850kcal fort	0.09 (−0.03, 0.21)
LNS 118kcal	* 0.12 (0.01, 0.24)
LNS 372kcal fort	0.10 (−0.03, 0.24)
Maternal Education	0.04 (−0.13, 0.20)
Iodine+Iron	0.06 (−0.08, 0.20)
DHA	0.05 (−0.03, 0.14)

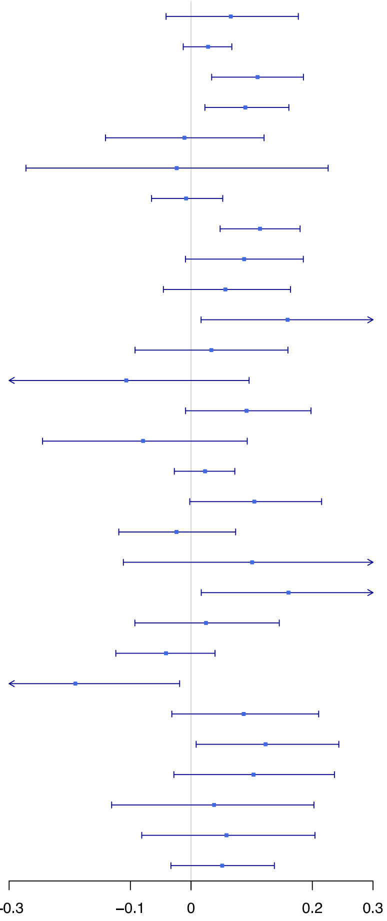

© 2019 Park JJH et al. *JAMA Network Open*.

eFigure 4C. LAZ forest plot (mean difference) for exclusive breastfeeding period, sensitivity analysis (non-cluster trials only)

vs. Standard of Care	Mean Diff. (95% interval)
MMN M	−0.04 (−0.38, 0.31)
MMN C	0.20 (−0.14, 0.52)
LNS 118kcal (20g) M	0.01 (−0.34, 0.36)
Zinc 5mg C	0.12 (−0.16, 0.31)
Formula 335kcal C	0.05 (−0.34, 0.43)
Formula+OPN 65 C	−0.13 (−0.53, 0.24)
Formula+OPN 130 C	−0.06 (−0.45, 0.32)
VitD M	0.08 (−0.28, 0.45)

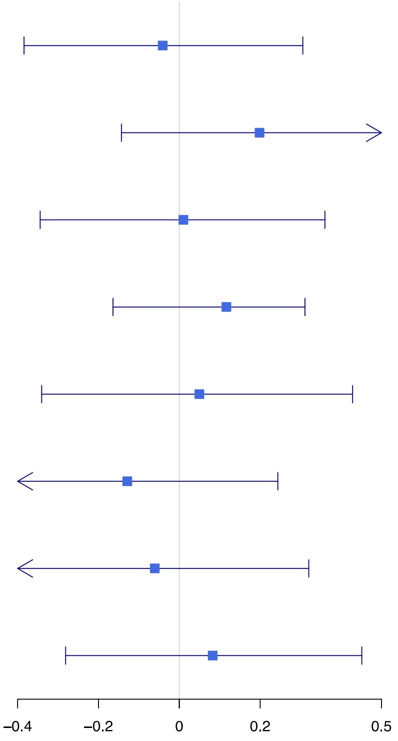

© 2019 Park JJH et al. *JAMA Network Open*.

eFigure 4D. HAZ forest plot (mean difference) for complementary feeding period, sensitivity analysis (non-cluster trials only)

vs. Standard of care	Mean Diff. (95% interval)
IFA	* 0.18 (0.04, 0.31)
IFA+Zinc	0.10 (−0.08, 0.29)
Iron	0.05 (−0.05, 0.15)
Iron+VitA	0.13 (−0.05, 0.30)
Iron+VitB2	0.35 (−0.21, 0.86)
Iron+Zinc	−0.05 (−0.15, 0.05)
Iron+Zinc+VitA	−0.01 (−0.26, 0.23)
Iron+Zinc+VitB2	0.15 (−0.42, 0.70)
Zinc	−0.02 (−0.09, 0.05)
Zinc+VitB2	−0.05 (−0.61, 0.49)
Zinc+VitBcomplex+C+E	0.08 (−0.04, 0.20)
VitB2	0.35 (−0.19, 0.86)
VitBcomplex+C+E	0.02 (−0.10, 0.14)
MMN weekly	0.08 (−0.22, 0.38)
MMN	* 0.16 (0.04, 0.28)
Antibiotics+Deworming 1dose	−0.04 (−0.31, 0.23)
Deworming 1dose	0.00 (−0.12, 0.11)
Deworming 2doses	0.02 (−0.11, 0.15)
Porridge 185−340kcal	−0.07 (−0.26, 0.13)
Porridge 185−340kcal fort	−0.03 (−0.32, 0.26)
Flour 270−340kcal	0.07 (−0.07, 0.22)
LNS 5−10g 23−55kcal fort	0.03 (−0.12, 0.17)
LNS20−25g 118−130kcal fort	−0.06 (−0.16, 0.04)
LNS40−55g 220−285kcal fort	−0.03 (−0.13, 0.07)
LNS75g 575kcal fort	0.28 (−0.43, 0.99)

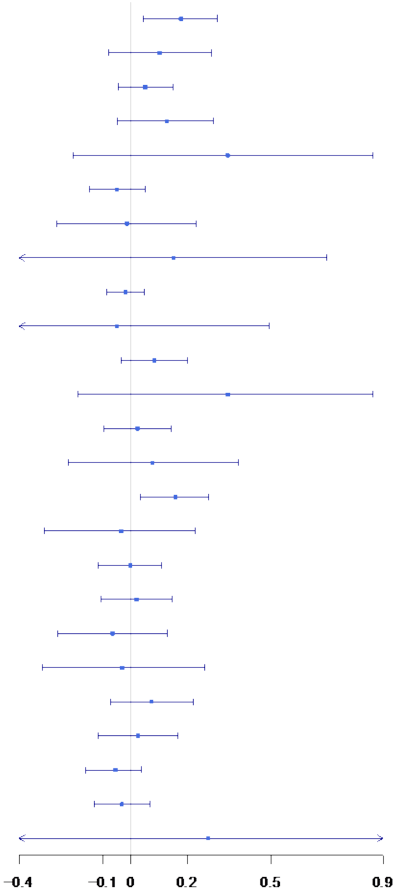

© 2019 Park JJH et al. *JAMA Network Open*.

MCID plots of primary analysis, cluster and non-cluster trials

eFigure 5A. Probability of being superior to SOC by at least the MCID for pregnancy, preterm birth, primary analysis (cluster and non-cluster trials)

MCID = 15% relative risk reduction

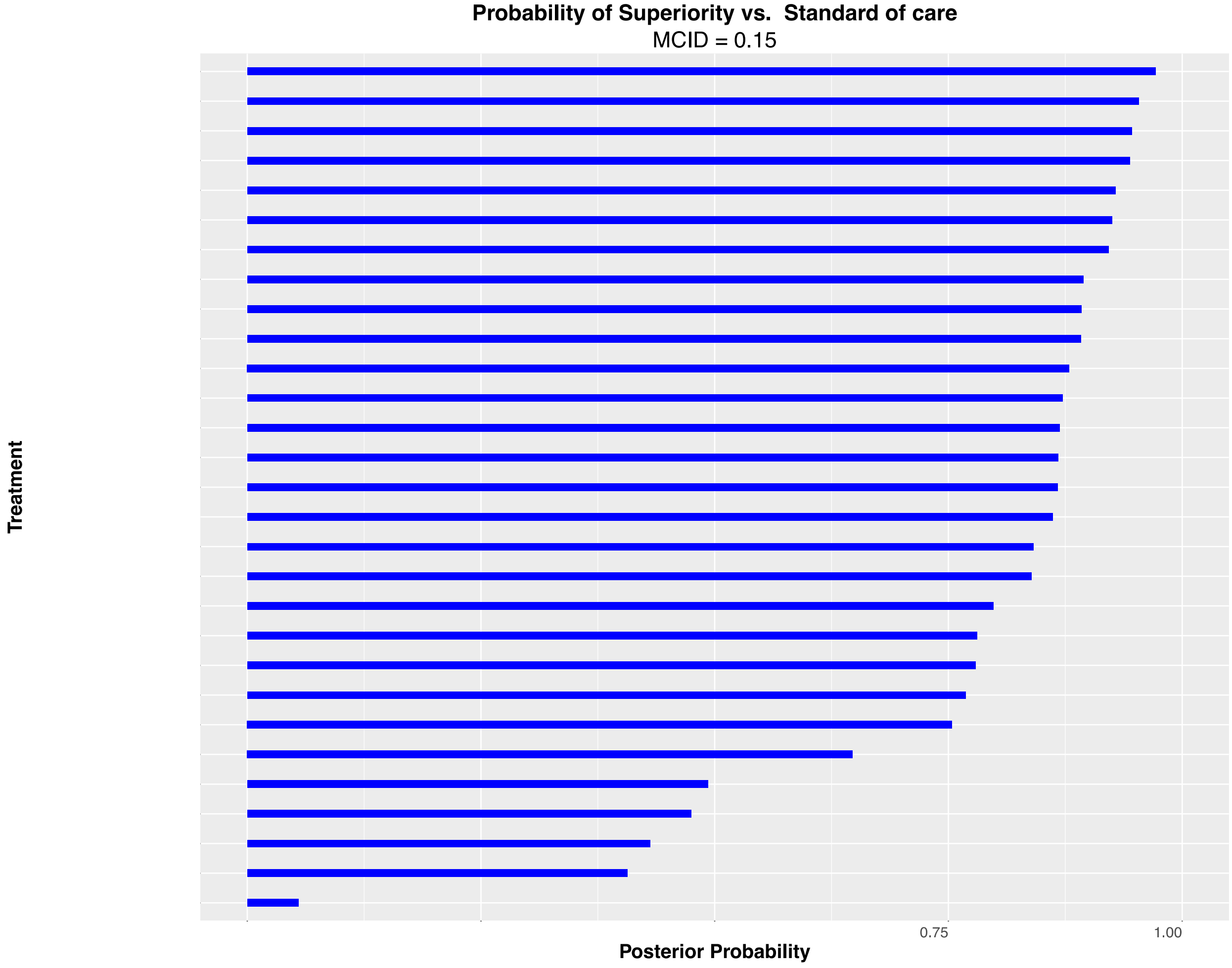

© 2019 Park JJH et al. *JAMA Network Open*.

eFigure 5B. Probability of being superior to SOC by at least the MCID for pregnancy, mean birthweight, primary analysis (cluster and non-cluster trials)

MCID = 71g of mean difference (0.15SD)

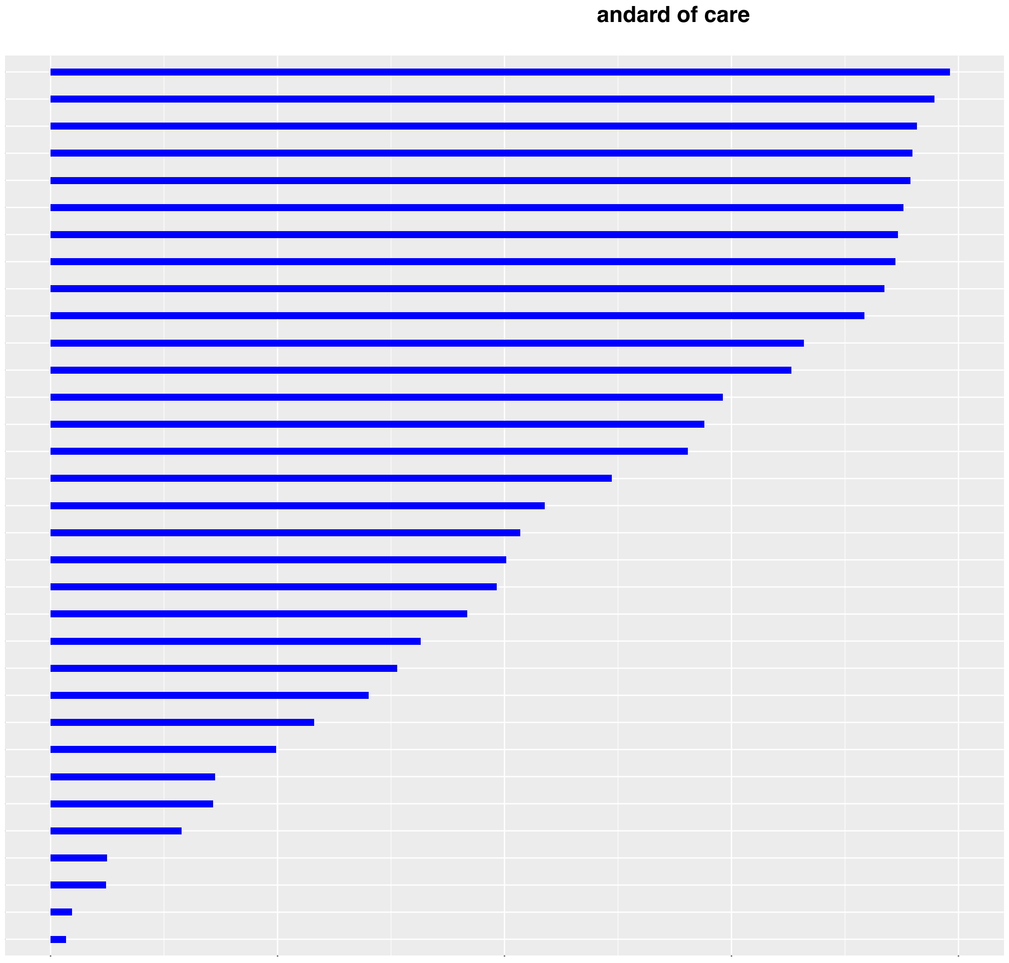

© 2019 Park JJH et al. *JAMA Network Open*.

eFigure 5C. Probability of being superior to SOC by at least the MCID for EBF, LAZ, primary analysis (cluster and non-cluster trials)

MCID = 0.15 (0.15 SD)

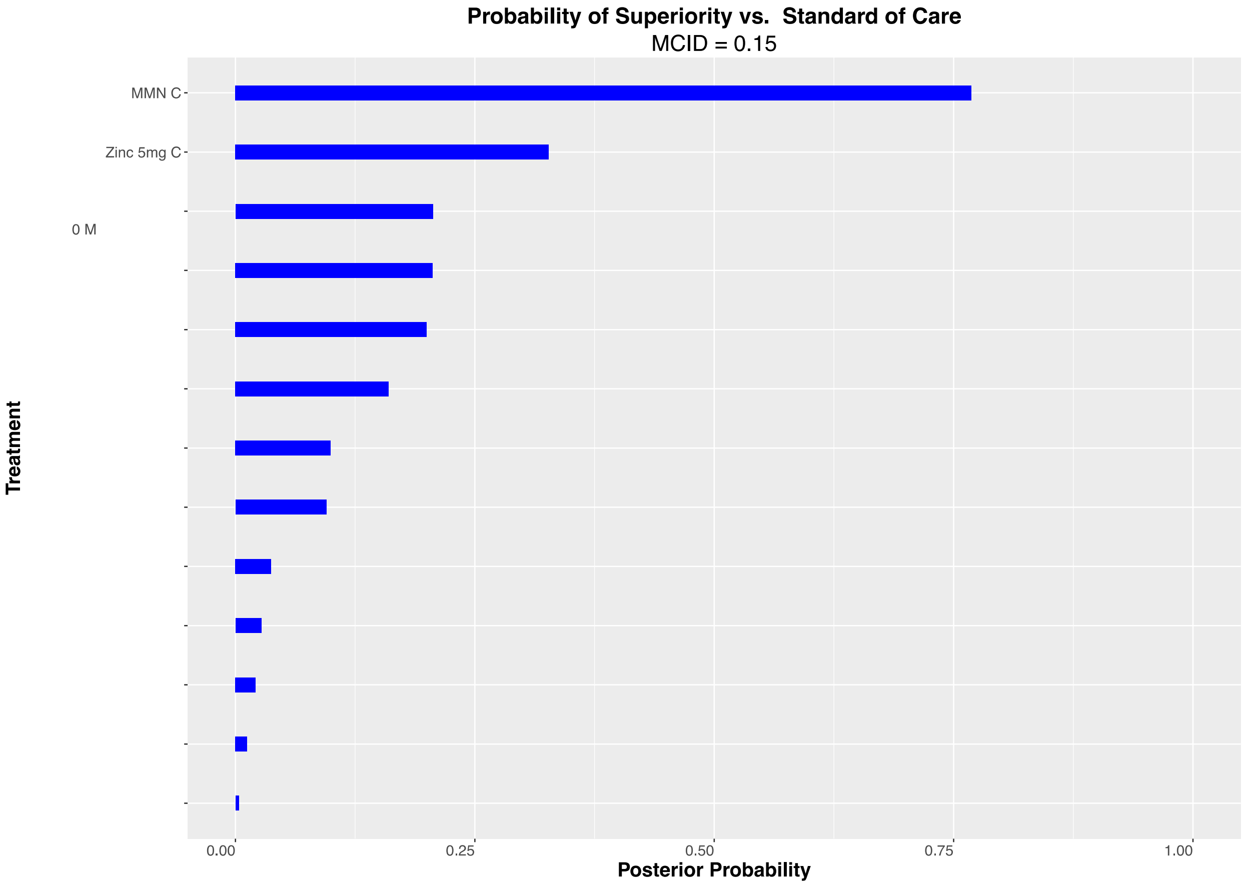

© 2019 Park JJH et al. *JAMA Network Open*.

eFigure 5D. Probability of being superior to SOC by at least the MCID for CFP, HAZ, primary analysis (cluster and non-cluster trials)

MCID = 0.15 (0.15 SD)

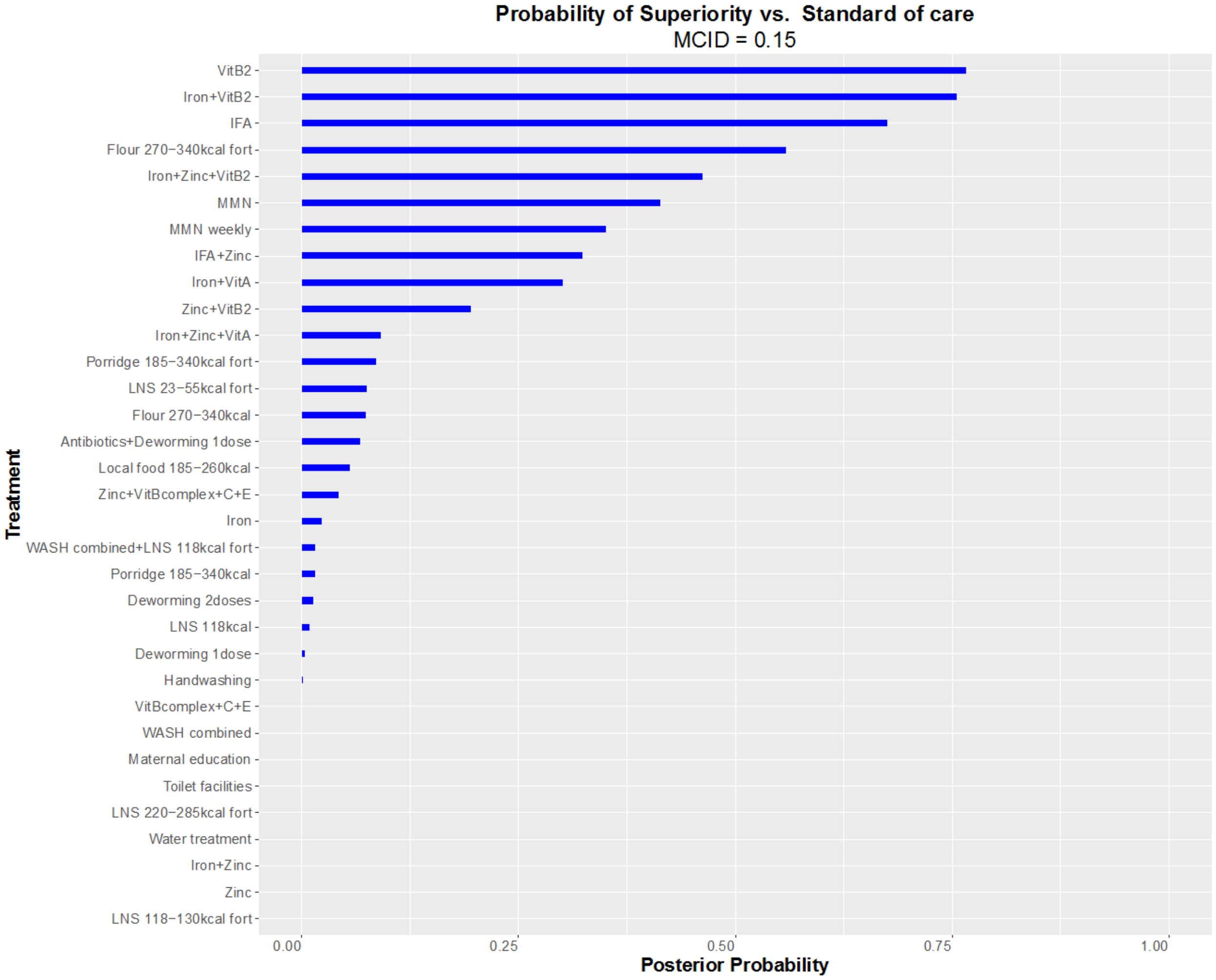

© 2019 Park JJH et al. *JAMA Network Open*.

MCID plots of sensitivity analysis I, non-cluster trials only

eFigure 6A. Probability of being superior to SOC by at least the MCID for pregnancy, preterm birth, sensitivity analysis (non-cluster trials only)

MCID = 15% relative risk reduction

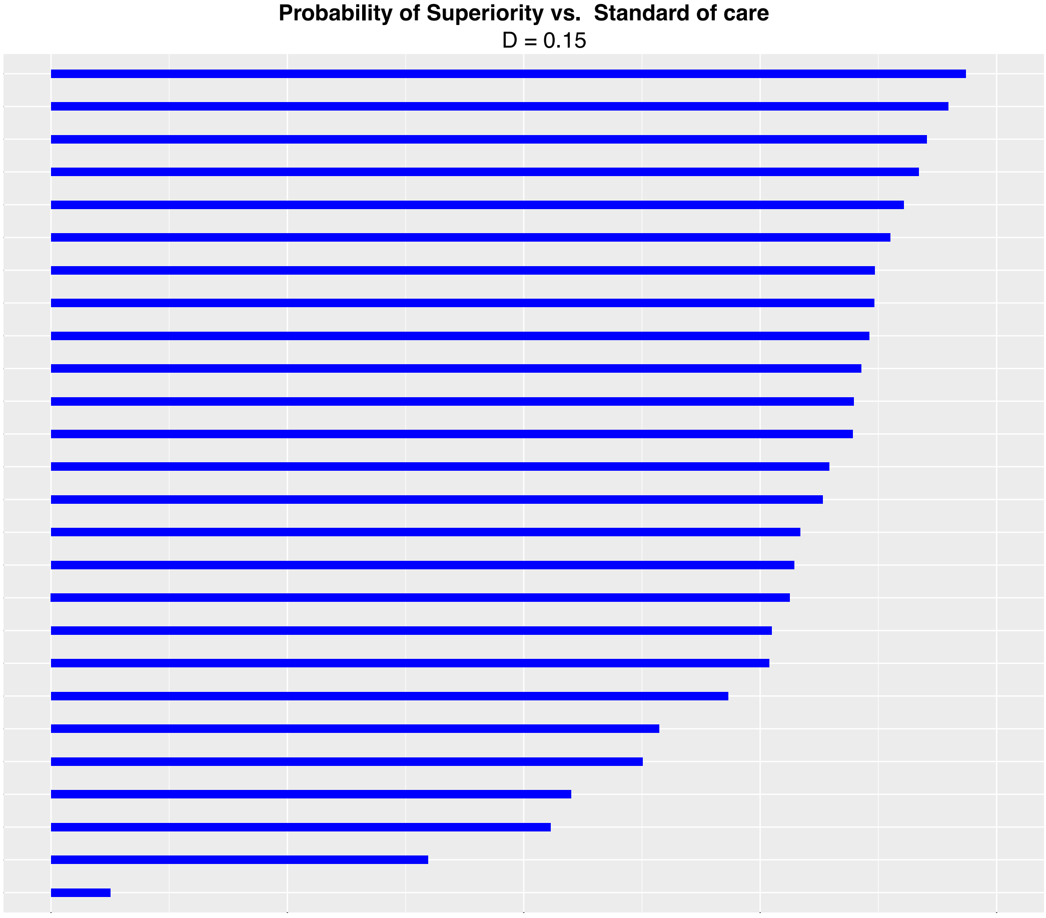

© 2019 Park JJH et al. *JAMA Network Open*.

eFigure 6B. Probability of being superior to SOC by at least the MCID for pregnancy, mean birthweight, sensitivity analysis (non-cluster trials only)

MCID = 71g (0.15 SD)

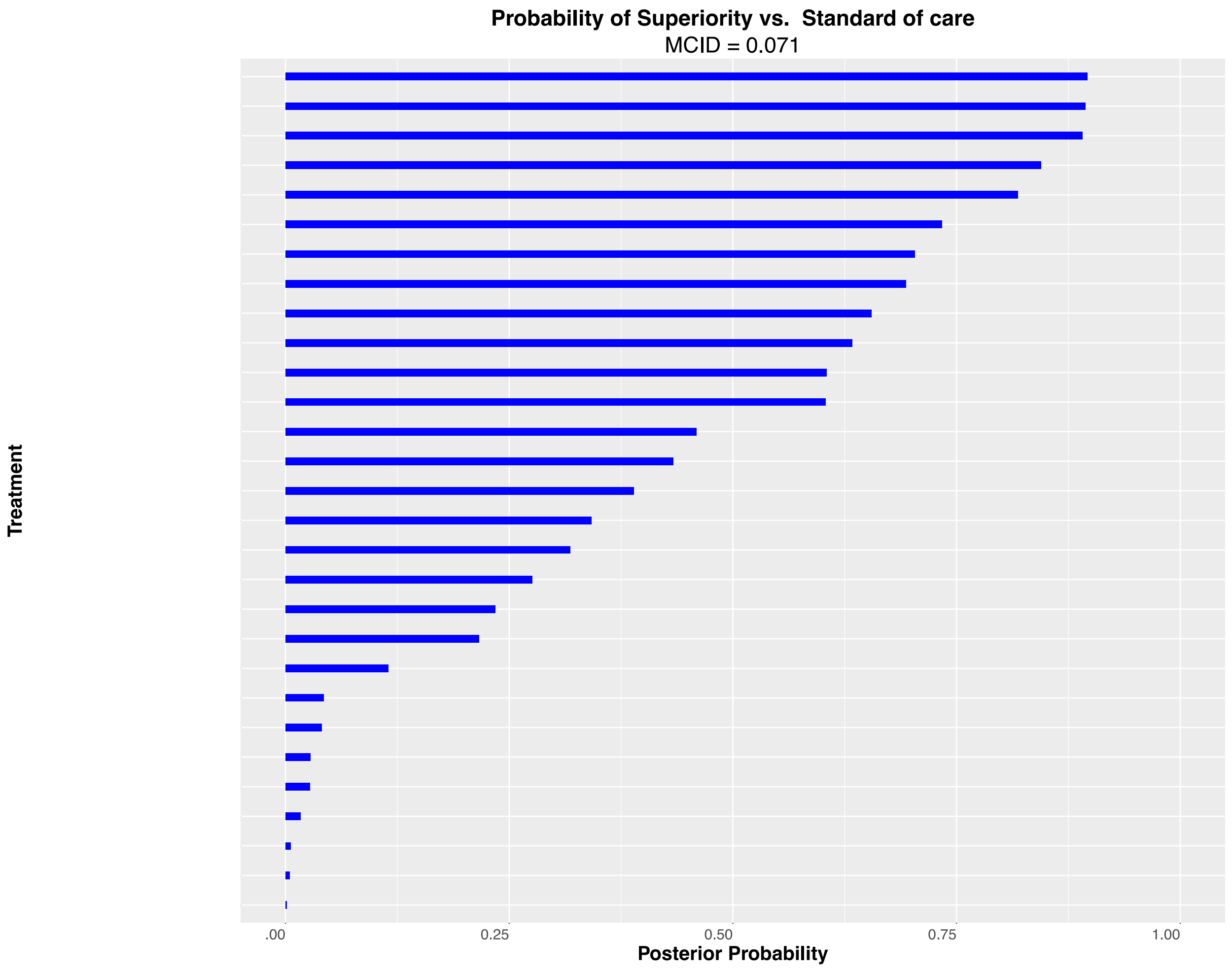

© 2019 Park JJH et al. *JAMA Network Open*.

eFigure 6C. Probability of being superior to SOC by at least the MCID for EBF, LAZ, sensitivity analysis (non-cluster trials only)

MCID = 0.15 (0.15SD)

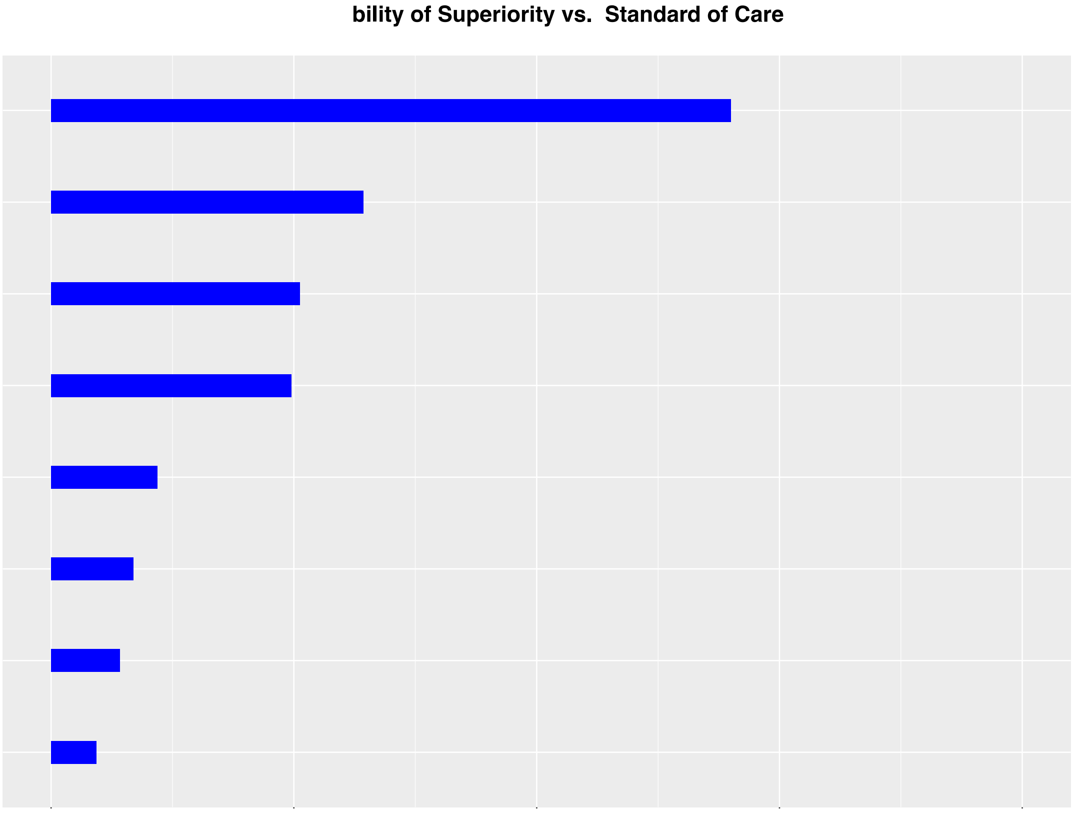

© 2019 Park JJH et al. *JAMA Network Open*.

eFigure 6D. Probability of being superior to SOC by at least the MCID for CFP, HAZ, sensitivity analysis (non-cluster trials only)

MCID = 0.15 (0.15 SD)

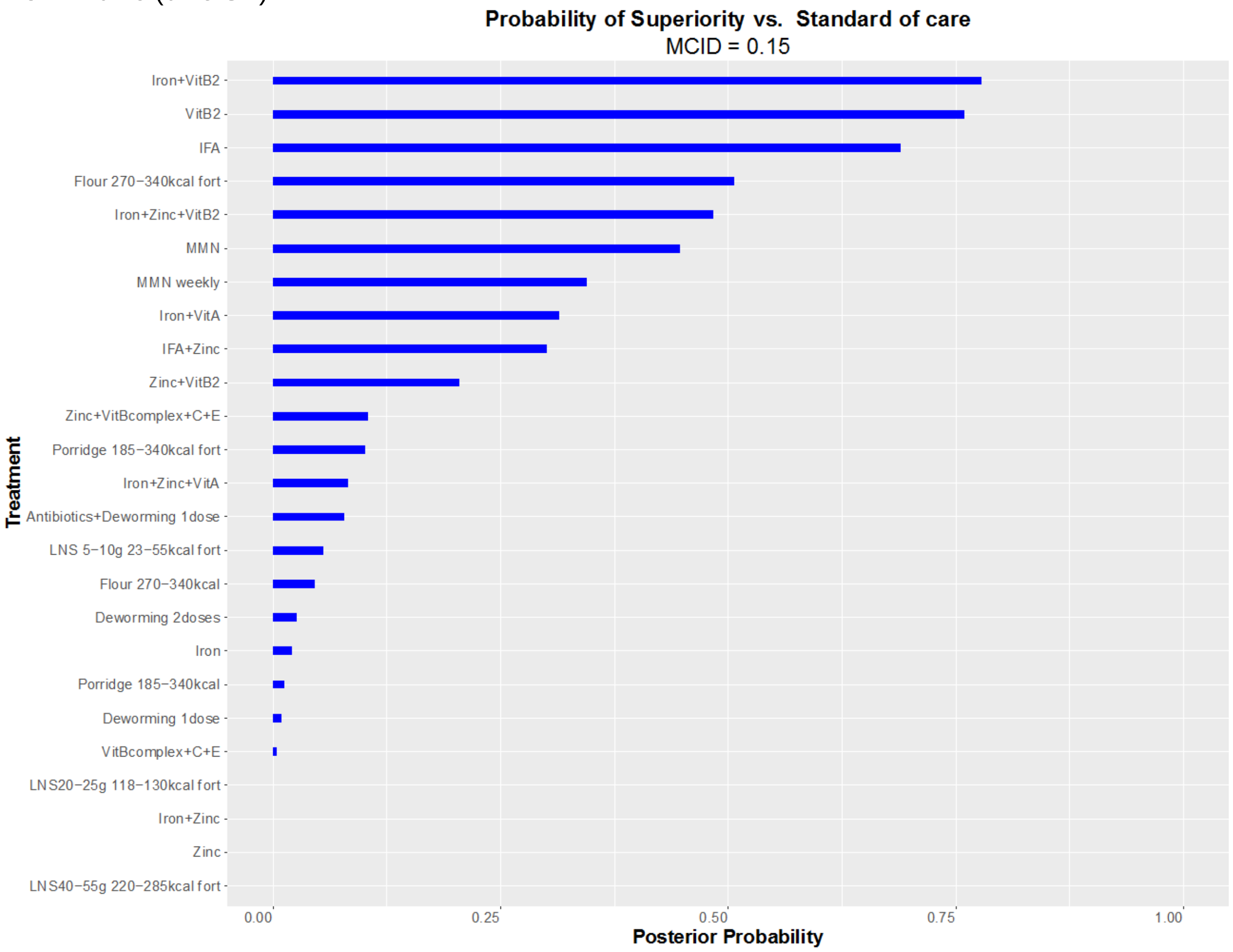

© 2019 Park JJH et al. *JAMA Network Open*.

MCID plots of sensitivity analysis II, differing MCID threshold

eFigure 7A. Probability of being superior to SOC by at least the lower MCID, the preterm birth NMA with cluster and non-cluster trials

MCID = 10% relative risk reduction

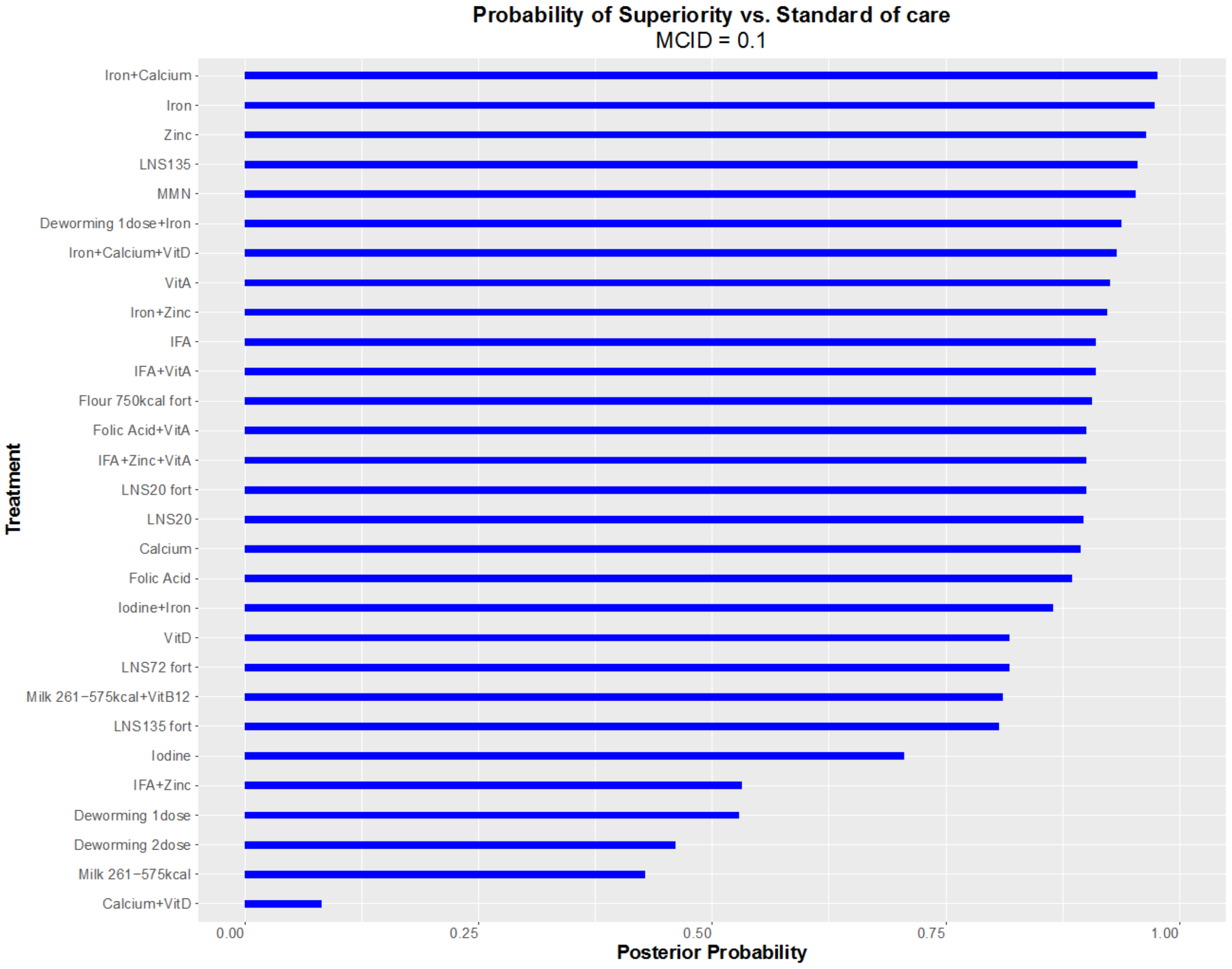

© 2019 Park JJH et al. *JAMA Network Open*.

eFigure 7B. Probability of being superior to SOC by at least the higher MCID, the preterm birth NMA with cluster and non-cluster trials

MCID = 20% relative risk reduction

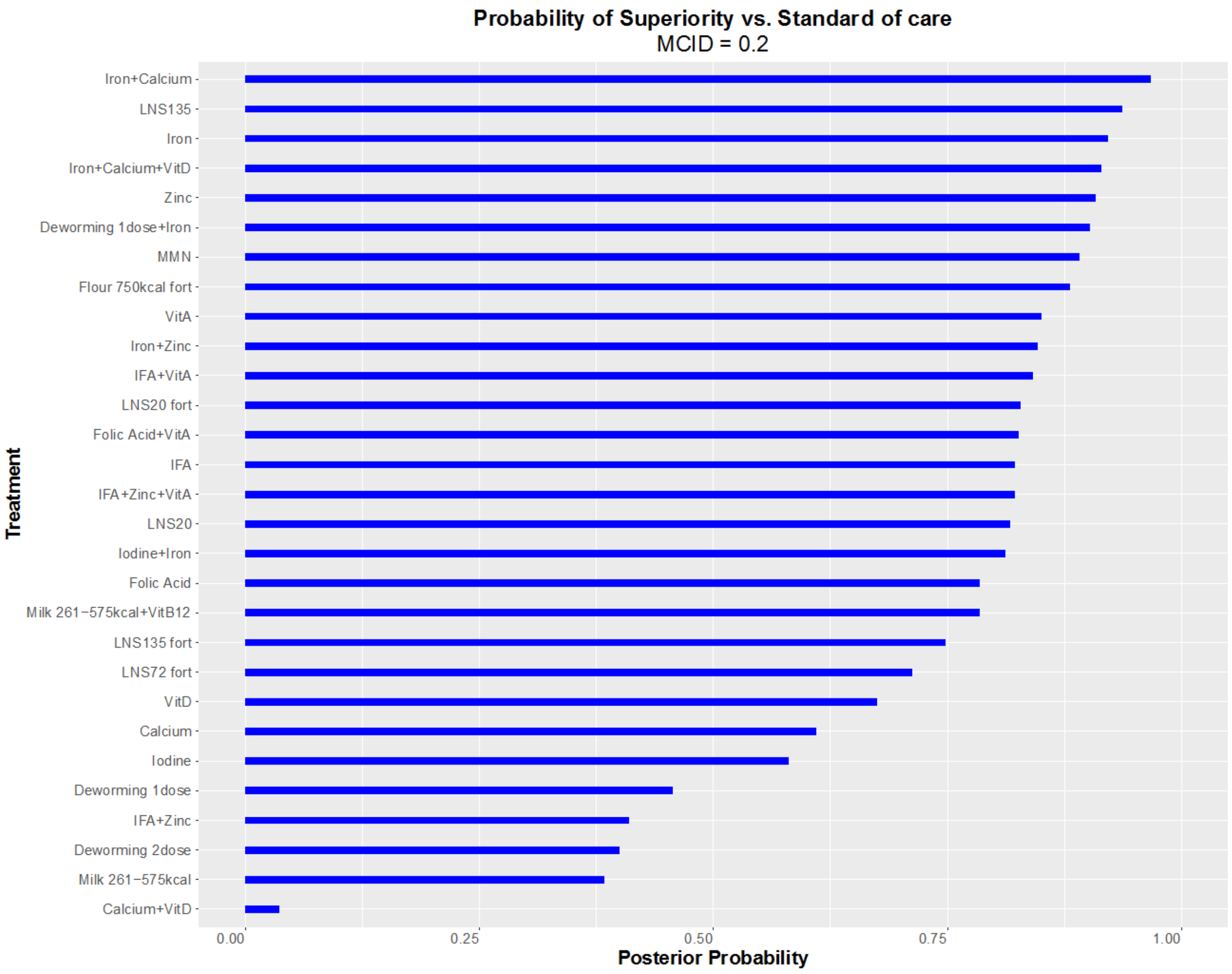

© 2019 Park JJH et al. *JAMA Network Open*.

eFigure 7C. Probability of being superior to SOC by at least the lower MCID, the mean birthweight NMA with cluster and non-cluster trials

MCID = 47.4g of mean difference (0.10 SD)

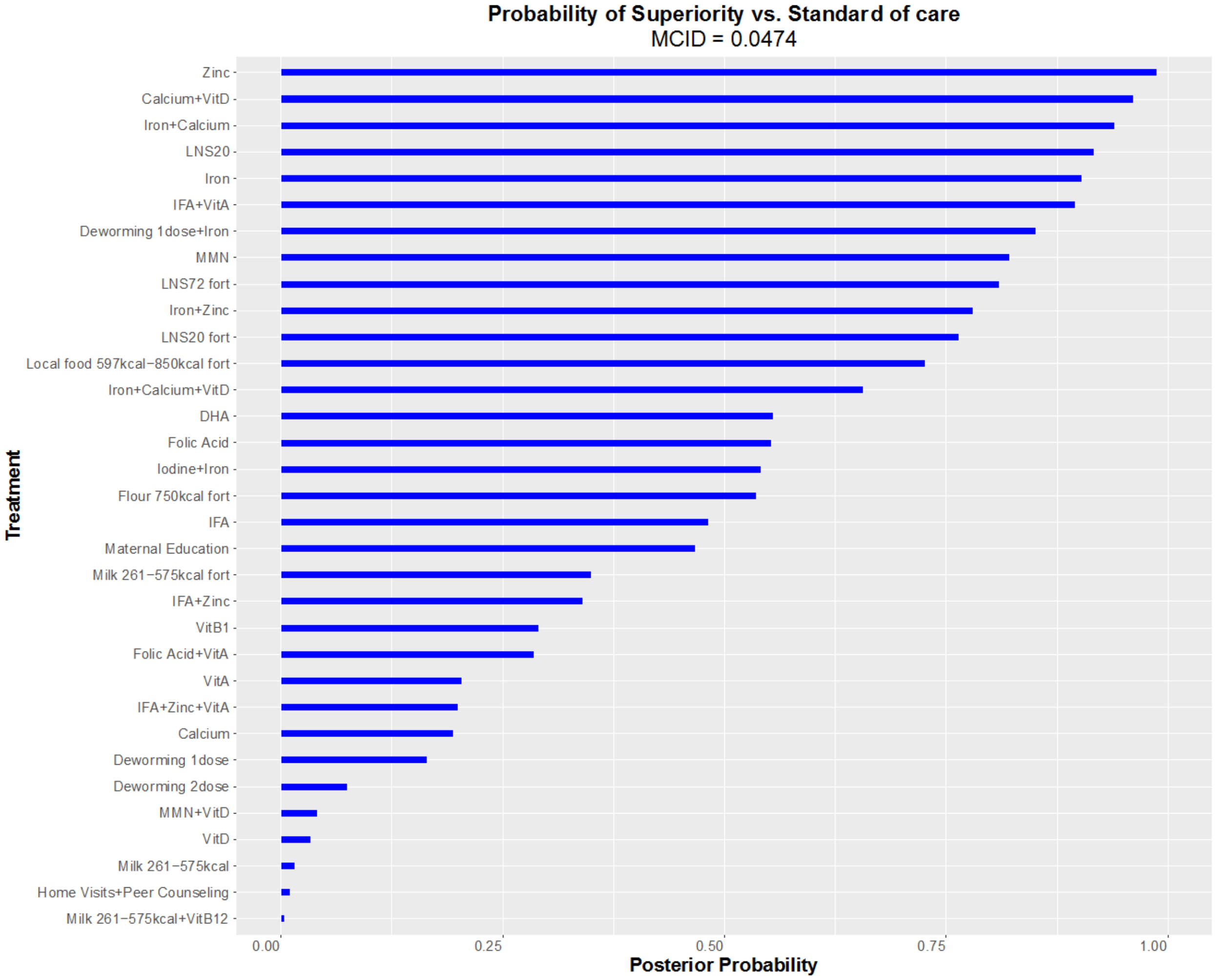

© 2019 Park JJH et al. *JAMA Network Open*.

eFigure 7D. Probability of being superior to SOC by at least the higher MCID, the mean birthweight NMA with cluster and non-cluster trials

MCID = 94.8g of mean difference (0.20 SD)

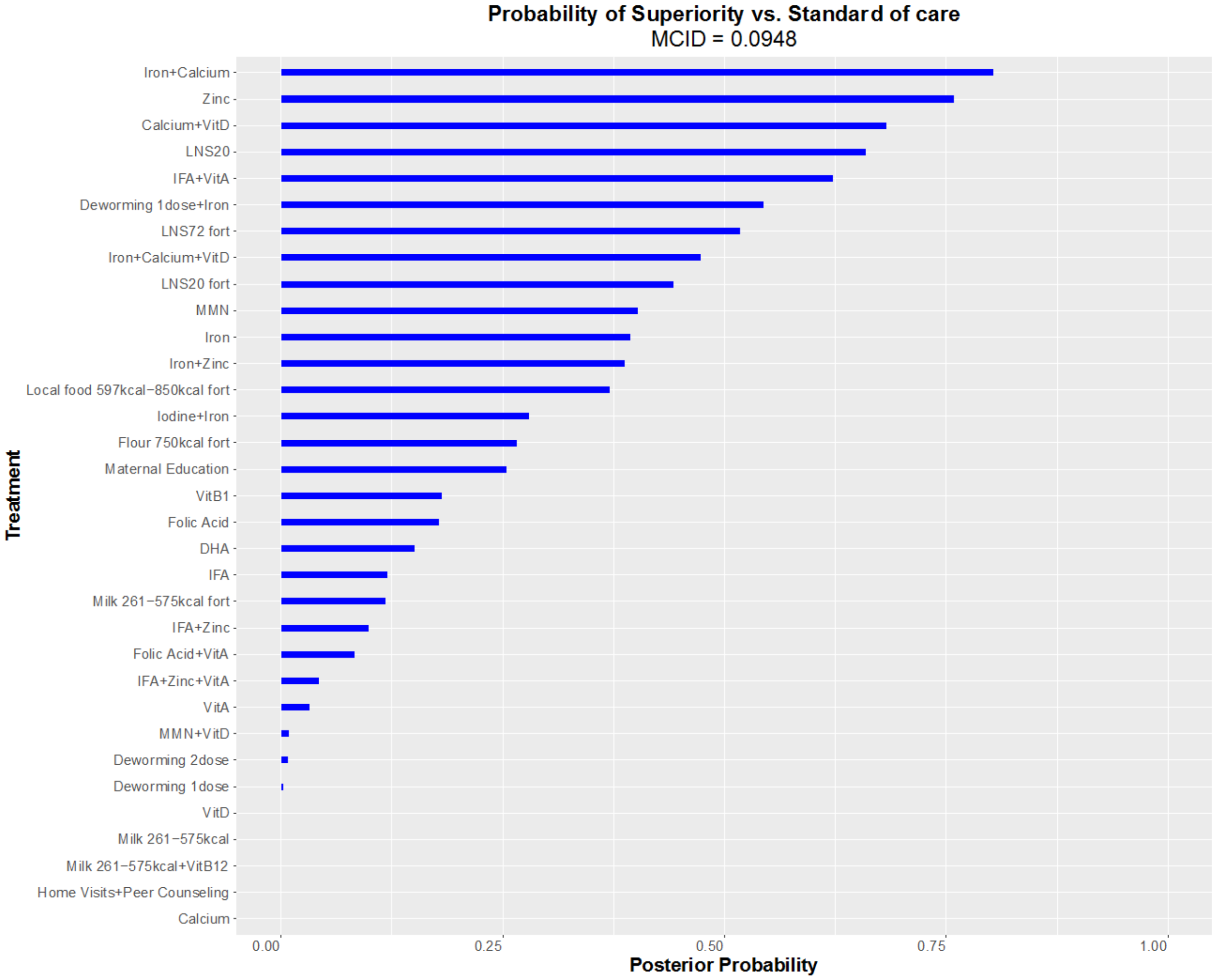

© 2019 Park JJH et al. *JAMA Network Open*.

eFigure 7E. Probability of being superior to SOC by at least the lower MCID, the LAZ NMA with cluster and non-cluster trials

MCID = 0.10 (0.10 SD)

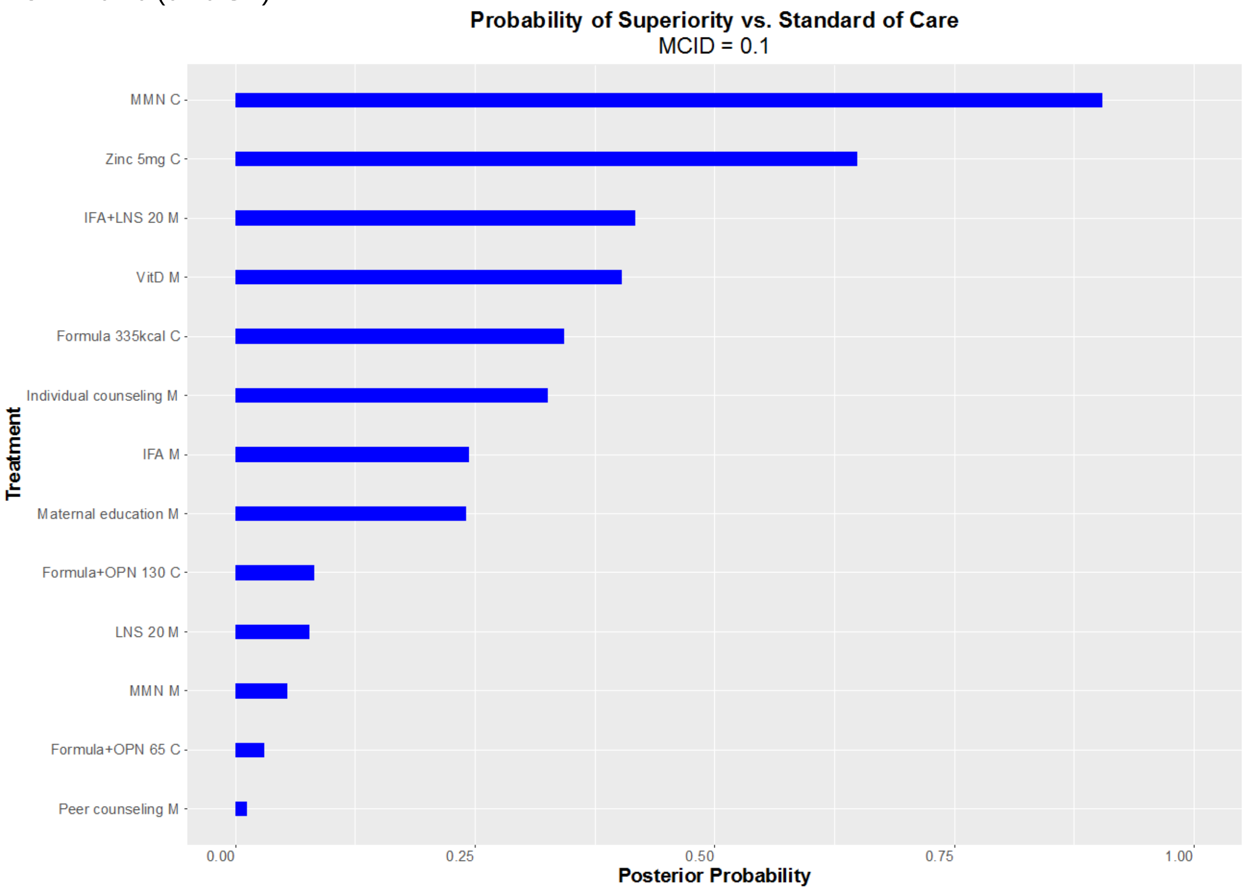

© 2019 Park JJH et al. *JAMA Network Open*.

eFigure 7F. Probability of being superior to SOC by at least the higher MCID, the LAZ NMA with cluster and non-cluster trials

MCID = 0.20 (0.20 SD)

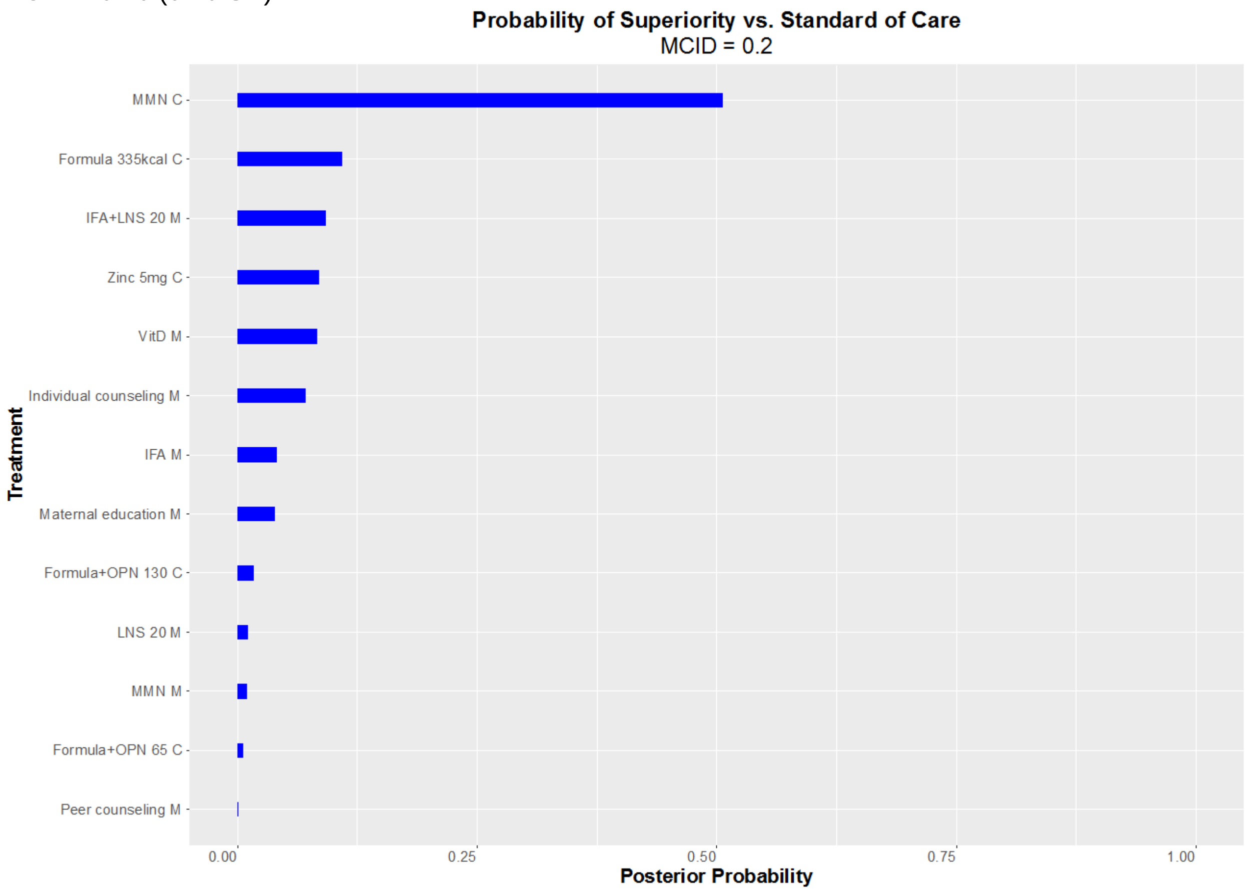

© 2019 Park JJH et al. *JAMA Network Open*.

eFigure 7G. Probability of being superior to SOC by at least the lower MCID, the HAZ NMA with cluster and non-cluster trials

MCID = 0.10 (0.10 SD)

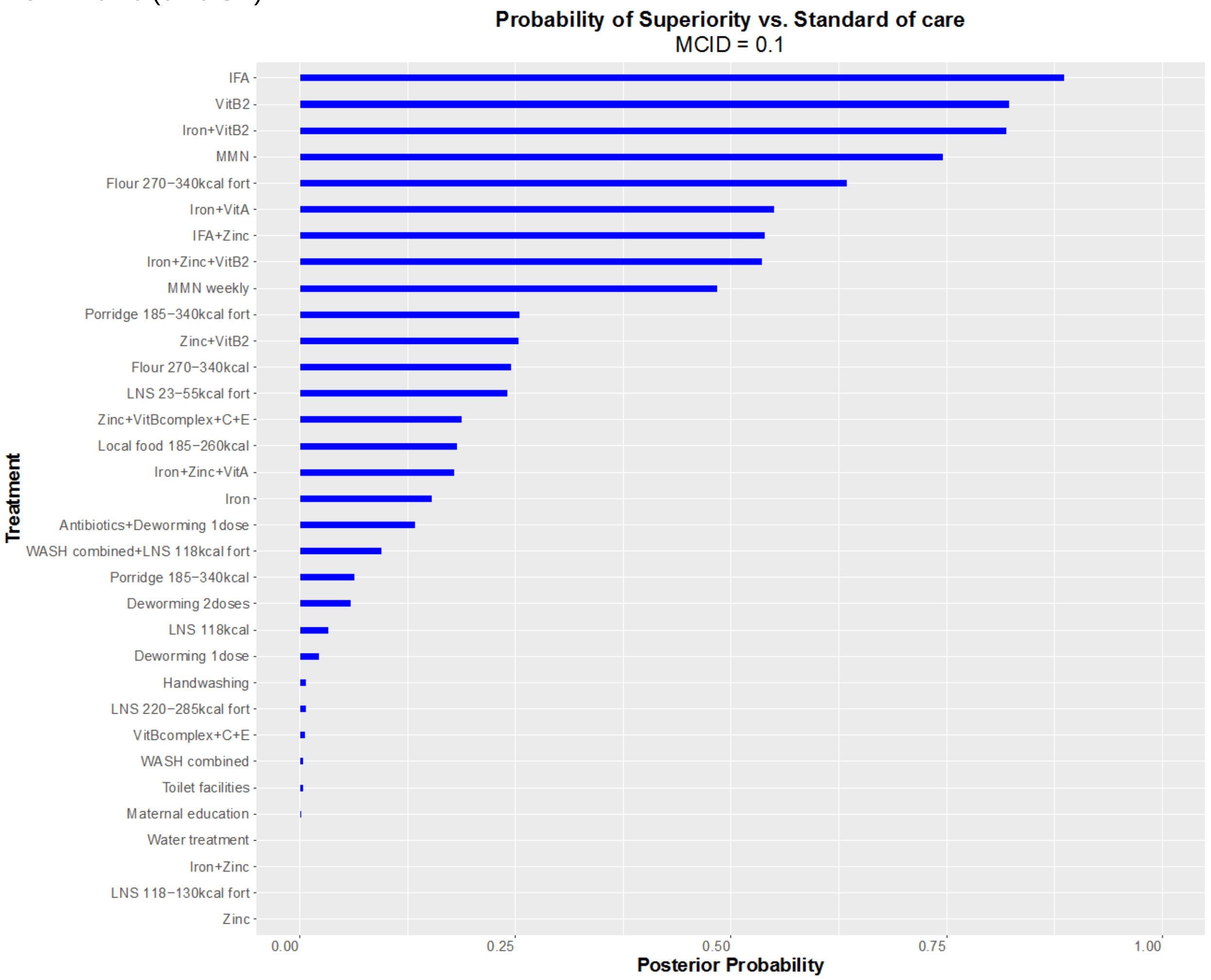

© 2019 Park JJH et al. *JAMA Network Open*.

eFigure 7H. Probability of being superior to SOC by at least the higher MCID, the HAZ NMA with cluster and non-cluster trials

MCID = 0.20 (0.20 SD)

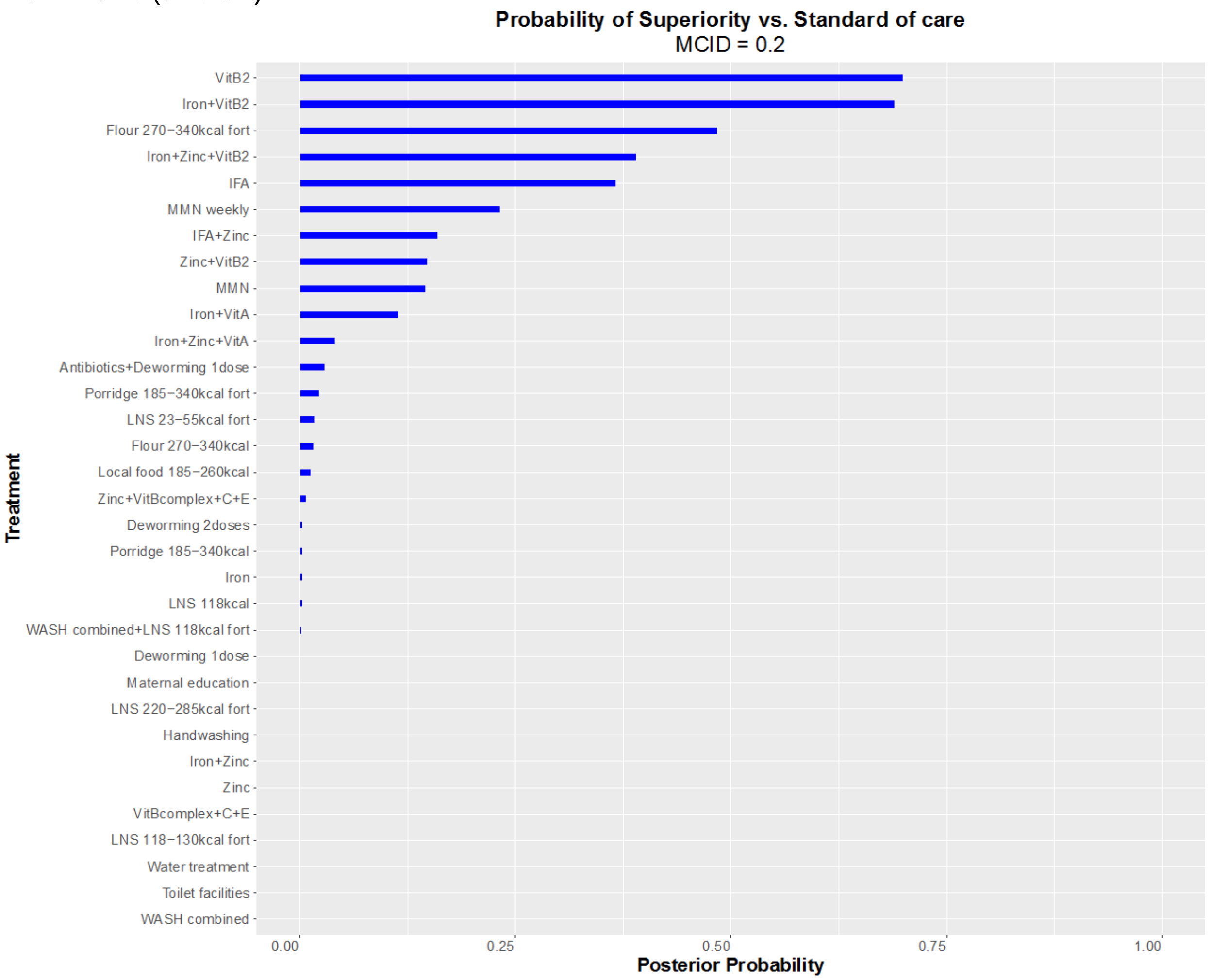

© 2019 Park JJH et al. *JAMA Network Open*.

© 2019 Park JJH et al. *JAMA Network Open*.

eAppendix. Details to our feasibility assessment and statistical analyses

Feasibility assessment of network meta-analyses

Feasibility assessment for network meta-analysis was done after systematic literature reviews for each of the life periods were completed.

We first compared the trial characteristics of included studies in terms of unit of randomization (individual versus cluster-based), blinding, geographic locations (i.e. continents and countries), organized by and across the intervention domains. We also compared the participant characteristics of all included trials. For the pregnancy NMA, we compared the participant characteristics in terms of trimester and mean gestational age at randomization, and follow-up and treatment duration. For the NMA for the exclusive breastfeeding period, we considered mothers' age at enrolment and gestational age at birth, and sex in terms of proportion of boys. For the complementary feeding NMA, we considered age of children and sex also in terms of proportion of boys recruited. The risk of bias was assessed using Cochrane risk of bias assessment tool in all of the included trials across the three life periods, without specific associations for the pre-specified outcomes.

Networks of evidence were then assessed for all of the outcomes in order to assess the connectivity of all included trials. The trials that could not be connected into the network were excluded from the analyses.

Data conversions

General conversions for continuous change from baseline data

For continuous outcomes, the effect measure was the mean difference between the change from baseline (CFB) of one treatment arm versus another treatment arm. Thus, the data input to the statistical model were the mean change from baseline for each arm and their associated standard errors. When trials do not report CFB, this can still be calculated for a specific time point by subtracting the endpoint value from the value at baseline. In this case, the associated standard error can only be approximated by assuming some correlation between the endpoint and baseline value. As a base case, we assumed a correlation of 0.8, but tested scenarios where 0.5 and 0.9 are assumed. The approximated standard error of the CFB was obtained via the basic formula:

$$\mathrm{SE(CFB)} = \sqrt{\mathrm{Var(Endpoint)} + \mathrm{Var(Baseline)} - 2 * \mathrm{Corr(Endpoint, Baseline)} * \mathrm{SE(Endpoint)} * \mathrm{SE(Baseline)}}$$

Where Var(X) denotes the variance of X, SE(X) the standard error of X, and Corr(X,Y) the correlation between X and Y.

Statistical models and effect measures

The treatments of interest were compared using Bayesian indirect comparisons following the Bayesian models recommended in the NICE TSD2.[1] Effect measures employed were relative risk for binary outcomes and CFB for continuous outcomes. To this end, in the Bayesian framework logistic regression were used for binary outcomes and linear regression were used for continuous outcomes. All approaches were employed under the random-effects framework. For the Bayesian models, the random-effects models recommended by NICE TSD2 were employed, however, due to the limited number of studies per comparison, a random-effects model with an empirically informed prior on the heterogeneity parameter were also employed.[2-4]

Model fit was assessed by comparing the Deviance Information Criterion (DIC) of the fixed-effect and random-effects models, as well as their respective leverage plots. All Bayesian models were performed in *R* using the *R2WinBUGS* package.[5,6]

Three separate chains and sufficiently many iterations (as indicated by Markov Chain Monte Carlo diagnostics) were used to confirm proper burn-in, and an additional number of iterations will be used to

© 2019 Park JJH et al. *JAMA Network Open*.

construct posterior distributions for all parameters. Longer chains were used should convergence diagnostics be deemed unsatisfactory. The median and 2.5th and 97.5th percentiles from the posterior distributions constituted the best estimate of effect and 95% credible interval for each of the comparative effects and other relevant parameters (e.g., the between-study heterogeneity).

Inclusion of cluster trials

When including data from a cluster randomized trial with estimated intraclaster correlation (ICC) ρ and average cluster size m in a network meta-analysis that also includes (non-cluster) randomized clinical trials, we make the following adjustments, as recommended by Uhlmann et al 2017.[7]

- the design effect $DEff = 1 + (m - 1)\rho$ is evaluated
- The effective sample size is calculated. The effective sample size of an intervention arm of size N is $N_{Eff} = N/DEff$.
- Similarly, when the outcome of interest is a dichotomous one, the number of cases is also adjusted, namely: $r_{Eff} = r/DEff$.
- Standard errors of mean differences are now adjusted to the effective sample size, that is: $SE_{Eff} = SE\sqrt{DEff}$.

Planned analyses

Primary outcomes

As a first round of indirect comparisons, the above models and effect measures were utilized to establish inference of comparative efficacy and safety of the interventions included in the networks.

Meta-regressions

When there are enough studies for a given network, it may be possible to perform a meta-regression analysis where the relative treatment effect of each study is a function of not only a treatment comparison of that study but also an effect modifier. In other words, with a meta-regression model we estimated the pooled relative treatment effect for a certain comparison based on the available studies, adjusted for differences in the level of the effect modifier between studies. Meta-regression analysis can help explain between-study heterogeneity and minimize (bias) in indirect comparisons due to transitivity violations. We may then model:

$$\theta_{jk} = \begin{cases} \mu_{jb} & if\ k = b,\ b \in A, B, C \\ \mu_{jb+}\, d_{bk} + \beta X_j & if\ k \succ b = \mathrm{A} \\ \mu_{jb+}\, d_{bk} & if\ k \succ b \succ \mathrm{A} \end{cases} \qquad (7)$$

for fixed effects, where β reflects the impact of study level covariate X_j. The random effect modification is as usual,

$$\theta_{jk} = \begin{cases} \mu_{jb} & if\ k = b, \quad b \in A, B, C \\ \mu_{jb+\delta_{jbk}+}\, \beta X_j & if\ k \succ b = \mathrm{A} \\ \mu_{jb+}\, \delta_{jbk} & if\ k \succ b \succ \mathrm{A} \end{cases}$$
$$\delta_{jbk} \sim \mathcal{N}(d_{bk}, \sigma^2) = \mathcal{N}(d_{Ak} - d_{Ab}, \sigma^2)$$

© 2019 Park JJH et al. *JAMA Network Open*.

$$d_{AA} = 0$$

Other examples where meta-regression may be useful include incorporating baseline risk (as a continuous covariate) in multiple treatment comparison with binary outcomes, or equivalently, a mean baseline continuous outcome.

Assessment of inconsistency

Where closed loops exist in an evidence network, it is important to check that the results from the direct evidence are consistent with the results of the indirect evidence (i.e., that consistency assumption is not violated). To the extent the consistency assumption is violated, results from a network meta-analysis may not be valid. Our assessment of inconsistency contained the following approaches:

First, data were be modelled using an independent means model, which contrary to the convention NMA model does not impose the assumption of consistency.[8] The DIC model fit statistics and comparative effect estimates of the two models were compared. If the independent means model appeared to provide a better fit, there was evidence of meaningful inconsistency. Secondly, the methods commonly known as edge-splitting were employed.[8] With this approach, a full network less a single pair wise comparison was modelled and results of the full NMA were compared to the direct evidence of the single pair wise comparison. This step was repeated until such a comparison has been made between every single pair wise comparison involved in at least one closed loop. In other words, the edge-splitting approach allowed for each pair wise direct evidence to be compared to the full body of indirect evidence.

For networks where closed loops exist, the above inconsistency tests were employed (where applicable). If evidence of inconsistency was identified, further statistical analyses were planned to explore the sources of inconsistency or increase consistency in the network (e.g., by meta-regression adjustments or subgroup analyses).

Analytical models to synthesize direct and indirect evidence

Network meta-analysis models to synthesize direct and indirect evidence

Fixed and random effect meta-analysis

At the center of the NMA lies a generalized linear model with a *likelihood* and a *link* function that are chosen to reflect the nature of the data at hand. The likelihood was defined in terms of an unknown parameter γ_{jk}, for treatment k in study j (e.g., a proportion of success within a binomial process). A link function $g(\cdot)$ was used to transform the parameter on a scale of all real numbers (such as a log of the odds or *logit* in the case of a proportion): $\theta_{jk} = g(\gamma_{jk})$. The transformed parameter was in turn modelled using a model that takes the following form:

$$\theta_{jk} = \begin{cases} \mu_j & k = A \\ \mu_j + d & k = B \end{cases} \qquad (1)$$

where μ_j represents this (transformed) outcome in trial *j* with comparator treatment *A*. *d* is the underlying *treatment effect* of B versus A on a normal scale that is the same for each study, j. With the random-effects meta-analysis model, δ_j is the trial-specific relative treatment effect of *B* relative to *A*. These trial-specific relative effects were drawn from a random effects distribution: $\delta_j \sim \mathcal{N}(d, \sigma^2)$:

$$\theta_{jk} = \begin{cases} \mu_j & k = A \\ \mu_j + \delta_j & k = B \end{cases} \qquad (2)$$

The basic NMA model

When the available evidence consisted of a network of multiple pairwise comparisons (i.e. AB-trials, AC-trials, BC-trials, etc.) the standard fixed effects model for NMA can be specified as follows:

$$\theta_{jk} = \begin{cases} \mu_{jb} & if\ \ k = b,\ \ b \in A, B, C \\ \mu_{jb} + \ d_{bk} = \mu_{jb} + \ d_{Ak} - d_{Ab} & \text{if } k \succ \text{b} \end{cases} \qquad (3)$$

$$d_{AA} = 0$$

There are *k* treatments labelled as *A, B, C,* etc., and treatment *A* was taken to be the reference treatment for the analysis. μ_{jb} was the (transformed) outcome in study j on 'baseline' treatment *b* which will vary across studies. d_{bk} was the fixed effect of treatment *k* relative to 'baseline treatment' *b.* d_{bk} were identified by expressing them in terms of the reference treatment A: $d_{bk} = \ d_{Ak} - d_{Ab}$ with $d_{AA} = 0$.

When the aggregate-level data set contained multiple direct comparisons of some of the pairs, assessment of the between-study treatment effect heterogeneity became possible. If there was evidence of strong heterogeneity (typically assessed by pair-comparisons forest plot) in which case a *random effect* model may be preferred. Here, in a similar manner to the fixed effect model, we assume that

$$\theta_{jk} = \begin{cases} \mu_{jb} & if\ k = b\ , b \in A, B, C \\ \mu_{jb} + \ \delta_{jbk} & \text{if } k \succ \text{b} \end{cases}, \qquad (4)$$

where the treatment effects δ_{jbk} are now assumed to be random and *exchangeable*, i.e.

$$\delta_{jbk} \sim \mathcal{N}(d_{bk},\ \sigma^2) = \ \mathcal{N}(d_{Ak} - d_{Ab},\ \sigma^2)$$
$$d_{AA} = 0$$

δ_{jbk} is the trial-specific treatment effect of *k* relative to treatment *b*. These trial-specific effects are drawn from a random effects distribution: $\delta_{jbk} \sim \mathcal{N}(d_{bk}, \sigma^2)$. Again, the pooled effects, d_{bk}, are identified by expressing them in terms of the reference treatment A. The heterogeneity σ^2 is assumed constant for all treatment comparisons (note that fixed effect model is obtained if σ^2 equals zero).
This random effect model treats multiple-arm trials (>2 treatments) without taking account of the correlations between the trial-specific $\delta's$ that they estimate. Bayesian random effects models with a heterogeneity parameter for d_{Ak} can be easily extended to fit trials with 3 or more treatment arms by decomposing a multivariate normal distribution as a series of conditional univariate distributions.[16]

$$\begin{pmatrix} \delta_{jbk_1} \\ \vdots \\ \delta_{jbk_p} \end{pmatrix} \sim \mathcal{N}\left(\begin{pmatrix} d_{bk_1} \\ \vdots \\ d_{bk_p} \end{pmatrix}, \begin{pmatrix} \sigma^2 & \cdots & \sigma^2/2 \\ \vdots & \ddots & \vdots \\ \sigma^2/2 & \cdots & \sigma^2 \end{pmatrix} \right) \qquad (5)$$

Then the conditional univariate distributions for arm i given the previous $1, \dots, (i-1)$ arms are:

$$\delta_{jbk_i} \mid \begin{pmatrix} \delta_{jbk_1} \\ \vdots \\ \delta_{jbk_{i-1}} \end{pmatrix} \sim \mathcal{N}\left(d_{bk_i} + \frac{1}{i} \sum_{j=1}^{i-1} \left(\delta_{jbk_j} - \ d_{bk_j} \right), \frac{(i-1)}{2i}\ \sigma^2 \right) \qquad (6)$$

Inconsistency

In the presence of a closed loop, violations of the transitivity assumption will show as systematic differences between direct and indirect estimates for comparisons of interventions that are part of the loop. Combining these may be inappropriate.

To help identify inconsistency it was informative to perform a meta-analysis of the relative treatment effects based on only the direct evidence, as well as a synthesis of only indirect evidence, before performing the network meta-analysis where we combine direct and indirect evidence.

A synthesis of only direct evidence can be performed by repeatedly performing traditional pairwise meta-analysis for each different direct comparison in the network. A more efficient approach is the use of

© 2019 Park JJH et al. *JAMA Network Open*.

independent-means models where we simultaneously obtain pooled estimates for all the different direct comparisons.[8]

The independent-means model (also referred to as the *inconsistency model*) simply dropped the consistency assumption on the mean treatment affects. For example, in a network consisting of treatments A, B, C and D, the usual (consistency model) estimates 3 basic parameters d_{AB}, d_{AC} and d_{AD}, while the remaining treatment effects are obtained from the consistency equations, namely

$$d_{BC} = d_{AC} - d_{AB},$$
$$d_{BD} = d_{AD} - d_{AB},$$

and

$$d_{CD} = d_{AD} - d_{AC}.$$

The inconsistency model (be it fixed or random effect) assumed that d_{AB}, d_{AC}, d_{AD}, d_{BC}, d_{BD} and d_{CD} are 6 unrelated parameters requiring estimation, having distinctive prior distributions.

Fitting the inconsistency model to the data was then followed up by comparing the results to the standard (consistency) model in terms point and interval estimates and DIC values. Plotting posterior mean deviance of individual data points for both models (consistency and inconsistency) against one another, provides a useful means for visual inspection of large discrepancies between the two models, that may point to loops in which inconsistency is present (i.e. conflicting direct and indirect evidences).

Continuous outcomes

Here it is common practice to assume

$$y_{jk} \sim \mathcal{N}(\theta_{jk}, \mathrm{se}_{jk}^2),$$

where y_{jk} is the observed average of treatment k in study j and se_{jk} the corresponding standard error. If all group sample sizes are reasonably large, this assumption is supported by the Central Limit Theorem.

Implicitly, the identity link is used for the mean parameter in this model.

Binary outcomes

When the outcome of interest is dichotomous, the binomial likelihood is used, namely

$$r_{jk} \sim b\mathrm{inom}(n_{jk}, p_{jk}),$$

where n_{jk} and r_{jk} are the number of people at risk and the number of cases observed in the kth treatment group of study j, and p_{jk} is the (unknown) probability of an individual in the group contracting the disease. The most common link function used is the *logit* link, whereby

$$\theta_{jk} = \log \frac{p_{jk}}{1-p_{jk}},$$

although other possibilities (such as the *probit* link) exist.

Bayesian approach

A network meta-analysis can be performed within a frequentist or Bayesian framework. Bayesian methods involve formal combination of a prior probability distribution (that reflects a prior belief of the possible values of the model parameters) with a (likelihood) distribution based on the observed data to obtain a posterior probability distribution of model parameters.[17] The likelihood informs us about the extent to which different values for the parameter of interest are supported by the data.

A major advantage of the Bayesian approach is that the method naturally leads into a decision framework. [9-11] [8-10] [63-65] [63-65] [63-6516-18] The posterior distribution can be interpreted in terms of probabilities

(e.g. "There is an x% probability that treatment A results in a greater response than treatment B"); frequentist approaches do not allow for such interpretation[11,12]. For a NMA, an additional advantage of a Bayesian approach is that it allows straightforward calculation of rank-probabilities[11].

Prior distributions

Popular choices of prior distributions include:

1. A $\mathcal{N}(\mu_0, \tau_\mu^2)$ prior for the baseline study-level means, μ_{jb}. Typically, we would take τ_μ^2 to be large, so that the prior is as little informative as possible.
2. A $\mathcal{N}(0, \tau_d^2)$ prior for the basic difference parameters, d_{Ak}, again with large τ_d^2.
3. A $\mathcal{N}(0, \tau_\beta^2)$ prior for the meta-regression coefficient β, with large τ_β^2.
4. Traditionally, a uniform prior distribution with a wide support has been used for the study heterogeneity standard deviation, σ. However, this choice often results in rather diffuse posterior distributions, as the between-study heterogeneity completely overshadows any within-study differences. This is especially true when the number of direct comparisons is small. Recent works[2,4,13] have explored the Cochrane Database of Systematic Reviews, and arrived at informative prior distribution for the between-study variance. For meta-analyses comparing interventions on binary outcomes, the $\log-\text{normal}(-2.34, 1.62^2)$ distribution was used.

Goodness of fit: the Deviance Information Criterion (DIC)

To help identify the most appropriate model given the evidence base, the goodness-of-fit of model predictions to the observed data can be measured by calculating the posterior mean residual deviance, $\bar{D}$.[14] The deviance information criterion (DIC) was used to compare competing models and provides a measure of model fit that penalizes models in a manner similar to the Akaike Information Criterion (AIC) used in frequentist scenarios; a smaller DIC indicates better fit, therefore the model with the smallest DIC be considered the most parsimonious given the data available.[10]

However, we do not recommend blindly choosing the model with the smallest DIC without further examination of diagnostics (e.g. residual plots) and results (e.g. forest plots, cross-tables). Generally, between two models with similar DIC values, we tend to pick the more "parsimonious" one (fixed effect over random effect etc.).

Markov Chain Monte Carlo sampling and convergence diagnostics

Posterior distributions leading to decisions were represented by *Monte Carlo samples,* drawn from said distributions through application of Markov Chain Monte Carlo (MCMC) methods. Basic NMA models have been implemented in the form of hierarchical Bayesian models in WinBUGS,[1] using Gibbs sampling.

When performed carefully, the Markov chain underlying the MCMC algorithm of choice is an *ergodic* one, and must thus converge to its stationary distribution, that is the joint posterior distribution of the model parameters, hence: if we sample for "long enough" and discard an early "burn-in period", the resultant sample should be a valid sample from the posterior distribution. Inference can then be based on Bayesian *credible intervals* for the different parameters or their functions, using sample quantiles.

Note that accurate quantile estimation requires a large number of independent samples for the *law of large numbers* to take place. Since the resultant MCMC sample was typically a (positively) autocorrelated one, the *effective sample size* (that is: the number of effectively independent draws within the sample) were considered.

© 2019 Park JJH et al. *JAMA Network Open*.

Assessing the convergence of the chain involves examination of diagnostic plots such as trace plots and autocorrelation plots for the different parameters. In addition, running multiple parallel chains allows for calculation of the Brooks-Gelman-Rubin statistic.[15] Using the above methods, we will begin each analysis with 100,000 MCMC iterations with a burn-in period of 90,000 and a thinning rate of 1. Note that depending on convergence and autocorrelation diagnostics, these parameters were subject to significant change according to the observed results of our analysis.

© 2019 Park JJH et al. *JAMA Network Open*.

eReference 2

eReference e includes all of references cited in eAppendix.

1. Dias S, Sutton AJ, Ades AE, Welton NJ. Evidence synthesis for decision making 2: a generalized linear modeling framework for pairwise and network meta-analysis of randomized controlled trials. *Medical decision making : an international journal of the Society for Medical Decision Making* 2013; **33**(5): 607-17.

2. Thorlund K, Thabane L, Mills EJ. Modelling heterogeneity variances in multiple treatment comparison meta-analysis – Are informative priors the better solution? *BMC Medical Research Methodology* 2013; **13**: 2-.

3. Turner RM, Davey J, Clarke MJ, Thompson SG, Higgins JP. Predicting the extent of heterogeneity in meta-analysis, using empirical data from the Cochrane Database of Systematic Reviews. *Int J Epidemiol* 2012; **41**(3): 818-27.

4. Rhodes KM, Turner RM, Higgins JPT. Predictive distributions were developed for the extent of heterogeneity in meta-analyses of continuous outcome data. *Journal of Clinical Epidemiology* 2015; **68**(1): 52-60.

5. R: A Language Environment for Statistical Computing. 2017. https://www.R-project.org/.

6. Sturtz S, Ligges U, Gelman A. R2WinBUGS: A Package for Running WinBUGS from R. *Journal of Statistical Software* 2005; **12**(3): 1-16.

7. Uhlmann L, Jensen K, Kieser M. Bayesian network meta-analysis for cluster randomized trials with binary outcomes. *Research synthesis methods* 2017; **8**(2): 236-50.

8. Dias S, Welton NJ, Sutton AJ, Caldwell DM, Lu G, Ades AE. Evidence Synthesis for Decision Making 4: Inconsistency in Networks of Evidence Based on Randomized Controlled Trials. *Medical Decision Making* 2013; **33**(5): 641-56.

9. Luce BR, Claxton K. Redefining the analytical approach to pharmacoeconomics. *Health Economics* 1999; **8**(3): 187-9.

10. Spiegelhalter DJ, Abrams KR, Myles JP. Bayesian approaches to clinical trials and health-care evaluation. Chichester: John Wiley & Sons; 2004.

© 2019 Park JJH et al. *JAMA Network Open*.

11. Sutton AJ, Abrams KR. Bayesian methods in meta-analysis and evidence synthesis. *Statistical methods in medical research* 2001; **10**(4): 277-303.

12. Goodman S. Toward Evidence-Based Medical Statistics. 1: The P Value Fallacy. *Ann Internal Medicine* 1999; **130**(12): 995.

13. Rhodes KM, Turner RM, White IR, Jackson D, Spiegelhalter DJ, Higgins JP. Implementing informative priors for heterogeneity in meta-analysis using meta-regression and pseudo data. *Statistics in Medicine* 2016; **35**(29): 5495-511.

14. Dempster AP. The direct use of likelihood for significance testing. *Statistics and Computing* 1997; **7**(4): 247-52.

15. Brooks SP, Gelman A. General methods for monitoring convergence of iterative simulations. *Journal of Computational and Graphical Statistics* 1997; **7**(434-455).

© 2019 Park JJH et al. *JAMA Network Open*.
